# Bidirectional Interaction Between PGE_2_-Preconditioned Mesenchymal Stem Cells and Myofibroblasts Mediates Anti-Fibrotic Effects: A Proteomic Investigation into Equine Endometrial Fibrosis Reversal

**DOI:** 10.3390/proteomes13030041

**Published:** 2025-09-08

**Authors:** Lidice Méndez-Pérez, Yat Sen Wong, Belén O. Ibáñez, Ioanna Martinez-Hormaza, Lleretny Rodríguez-Álvarez, Fidel Ovidio Castro

**Affiliations:** 1 Laboratory of Animal Biotechnology, Department of Animal Science, Faculty of Veterinary Sciences, Universidad de Concepción, Chillán 3780000, Chile; lmendez@udec.cl (L.M.-P.); ywong@udec.cl (Y.S.W.); belenibanez@udec.cl (B.O.I.); iomartinez@udec.cl (I.M.-H.); 2 Vascular Physiology Laboratory, Group of Research and Innovation in Vascular Health, Basic Sciences Department, Faculty of Sciences, Universidad del Bio-Bio, Chillán 378000, Chile

**Keywords:** endometrosis, equine mesenshymal stem cells, mare endometrial, precondition with PGE_2_, SILAC, myofibloblasts-equine mesenshymal stem cells interaction

## Abstract

Background: Endometrosis is a prevalent fibrotic condition in mares that impairs reproductive efficiency by inducing transdifferentiation of endometrial stromal cells into myofibroblasts, leading to excessive ECM deposition. Methods: To elucidate the molecular mechanisms underlying fibrosis resolution, this study employed comprehensive proteomic techniques, including LC-MS/MS and SILAC, to analyze the interaction between myofibroblasts and mesenchymal stem cells derived from the endometrium (ET-eMSCs) preconditioned with PGE_2_. An in vitro co-culture system was used, with samples collected at baseline and after 48 h. Results: Proteomic analysis identified significant alterations in proteins associated with ECM remodeling, immune regulation, and cellular stress response. Notably, proteins involved in collagen degradation, antioxidant defense, and growth factor signaling pathways were differentially abundant. Network analyses demonstrated robust interactions among these proteins, suggesting coordinated modulatory effects. The data indicate that PGE_2_-primed ET-eMSCs induce a shift in myofibroblast secretory profiles, promoting a reduction in ECM stiffness, tissue reorganization, and activation of resolution pathways. Data are available via ProteomeXchange with identifier PXD067551. Conclusions: These findings reinforce the therapeutic potential of mesenchymal stem cell-based interventions for fibrotic diseases of the endometrium, opening avenues for regenerative strategies to restore reproductive function in mares.

## 1. Introduction

Endometrosis is widely recognized as one of the most critical reproductive disorders affecting mares characterized by the fibrotic remodeling of the endometrium. Endometrosis compromises early pregnancy maintenance by disrupting endometrial structure and function during the critical peri-implantation period [1,2,3]. The earliest detectable alterations involve the atypical differentiation of stromal cells, which adopt an elongated and polygonal morphology accompanied by increased metabolic activity, a hallmark of myofibroblast-like activation [4,5,6].

The activation of myofibroblasts is characterized by the production of contractile proteins, such as α-smooth muscle actin (α-SMA), and a pronounced accumulation of ECM components, notably collagen types I and III [7,8,9]. Despite the profound impact of endometrosis on reproductive performance, no effective treatment currently exists [10]. Recent approaches have focused on modulating local inflammation and promoting cellular regeneration as novel therapeutic strategies.

In this context, mesenchymal stem cell (MSC) therapies have emerged as a promising alternative due to their antifibrotic properties and their role in maintaining tissue homeostasis [11,12]. These therapeutic effects were attributed to the MSC secretome, which was enriched in lipids, growth factors, microRNAs, and cytokines that were shown to inhibit apoptosis and fibrosis, enhance angiogenesis, drive progenitor cell proliferation and differentiation, and modulate immune responses [13]. Extracellular vesicles were also released into the conditioned medium and recognized as key mediators of intercellular communication [14].

We investigated equine MSCs (eMSCs) and their secretome as potential regenerative therapy for endometrosis and endometritis [15,16,17,18]. In previous assays, myofibroblasts were co-cultured with equine endometrial-derived MSCs (ET-eMSCs) that had been preconditioned with PGE_2_ in a transwell system, and a statistically significant reduction was observed in the relative expression of fibrosis-related genes (α-SMA, COL1A1, COL3A1, MMP-9, and MMP-2), all of which are known to contribute to fibrotic progression. However, no transcriptional changes were detected when the myofibroblasts were exposed solely to the conditioned medium of these cells [19].

These observations led us to hypothesize that the interaction between ET-eMSCs and myofibroblasts plays a bidirectional role in modulating the fibrotic response. Specifically, this crosstalk appears to enrich the ET-eMSC secretome with antifibrotic molecules, thereby exerting a “healing” effect on myofibroblasts, while myofibroblasts, in turn, provide stimulatory feedback to the ET-eMSCs via paracrine mechanisms. To explore this hypothesis, we employed label-free quantitative proteomics to identify differentially abundant proteins (DAPs) in the proteome of endometrial myofibroblasts co-cultured with ET-eMSCs that had been preconditioned with PGE_2_. Based on our previous work, we challenged ET-eMSC with PGE_2_ as a trigger of antifibrotic activity [16,19]. Next, a quantitative proteomics assay using SILAC was conducted on the co-culture experiment, enabling proteins in the culture supernatant to be traced back to their source cell isolates.

By integrating proteomic and functional analyses, this study aims to elucidate the molecular mechanisms underlying the antifibrotic effects mediated by MSC-derived secretome. Our findings are expected to contribute to a better understanding of the therapeutic potential of these cellular therapies in the treatment of endometrosis, advancing the field of regenerative medicine

## 2. Materials and Methods

The animal study was approved by the Ethics Committee of the Faculty of Veterinary Sciences, University of Concepción, Chile (CEBB 907-2021). Sample collection was carried out during the reproductive season in the Southern Hemisphere (August to January). All procedures complied with local legislation and institutional guidelines.

Endometrial tissues used in this study were obtained from clinically healthy Chilean Thoroughbred mares (*n* = 6) during routine slaughter at an authorized facility. Prior to tissue collection, all animals were examined by a licensed veterinarian to confirm their reproductive and systemic health. Only mares clearly identified in the follicular phase of the estrous cycle were selected for further processing, as determined by ovarian morphology. Uteri were incised longitudinally to access the uterine cavity, and endometrial tissue was excised in longitudinal strips from the horn region, strictly excluding the myometrial layer by the methodology reported by [20]. Tissue integrity was verified macroscopically before processing, and samples were subjected to histopathological assessment of endometrosis according to the scale of Kenney and Doig [21]. Samples were processed immediately upon extraction; if endometrosis was detected after, the samples were discarded, and no cell culture was conducted for those particular samples.

Samples were individually processed using a standardized enzymatic dissociation protocol. After two washes in PBS (1X) containing a double concentration of Antibiotic-Antimycotic Solution (Corning™ CI, NY, USA), 15 g of endometrial tissue per mare were finely minced into approximately 1 mm^2^ fragments. The resulting material was incubated in high-glucose DMEM (Sigma-Aldrich™, Saint Louis, MO, USA) supplemented with 1 mg/mL of Collagenase Type I (Gibco™, Thermo Fisher Scientific Inc., Waltham, MA, USA) for 2 h at 38 °C under continuous gentle agitation. Digested suspensions were mechanically homogenized and filtered through 40 µm strainers (Corning™ 4), followed by centrifugation at 1000 rpm for 10 min at room temperature.

Cell pellets were resuspended in complete culture medium (DMEM high glucose + 10% FBS [Sigma-Aldrich™ F2442] + 1X AAM) and seeded in 100 mm sterile Petri dishes. Cells were maintained at 38 °C with 5% CO_2_, and the medium was changed every 2–3 days until cultures reached 90% confluence. Expanded cells were transferred to T175 cm^2^ flasks (SPL Life Sciences 71175, Gyeonggi-do, Republic of Korea) and cryopreserved at passage 2 (P2). To ensure culture uniformity, ET-eMSC isolates were pooled to minimize inter-sample variability in growth kinetics and cell–cell signaling dynamics. Given that ET-eMSCs exhibit a population doubling time ranging from 24 to 34 h and demonstrate contact-dependent proliferation, each isolate was seeded at an equivalent initial cell density. This approach facilitated synchronized progression toward the target confluence required for the assay, reducing discrepancies in proliferation-associated variables and optimizing experimental consistency under limited sample conditions.

These equine endometrial mesenchymal stem cells exhibited a surface marker profile consistent with mesenchymal lineage, characterized by positive expression of CD90 and CD44, and absence of CD45 and MHCII, following established characterization criteria [22,23].

An equine endometrial fibroblast was previously isolated and characterized in our laboratory as described by [24] and further validated following the methodology reported by [17]. All cellular derivation from animal samples was performed under the mentioned permit (CEBB 907-2021; Universidad de Concepción). No commercial cell lines were used in this study.

### 2.1. Experimental Design

#### 2.1.1. Experiment 1: Proteomic Analysis of the Interaction of Endometrial Myofibroblasts with PGE_2_-Conditioned Equine Mesenchymal Stem Cells Derived from Endometrial Tissue (ET-eMSCs) for the Study of Endometrosis

This experiment evaluated the combined effect of soluble mediators and indirect cell–cell interactions mediated by PGE_2_-preconditioned ET-eMSCs on myofibroblast behavior. Myofibroblasts were co-cultured with preconditioned ET-eMSCs using a permeable membrane system, allowing only paracrine signaling. ET-eMSC isolates were combined into a single pooled culture and subjected to triplicate experimental replicates.

#### 2.1.2. Induction of Myofibroblasts from Endometrial Fibroblasts

**Figure 1:**
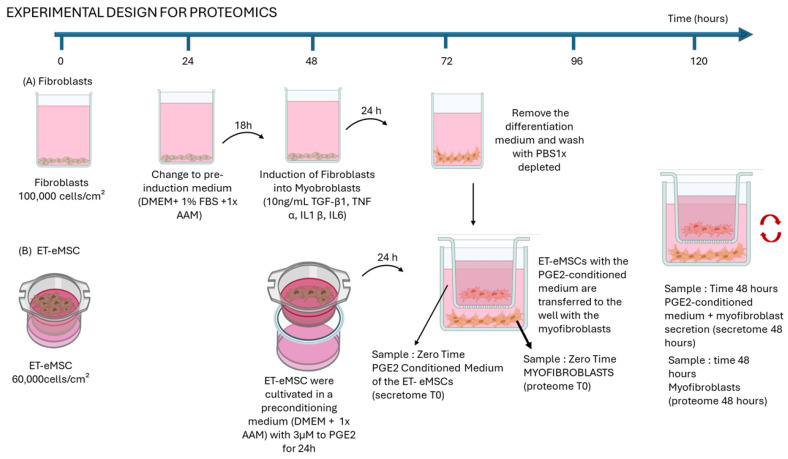
Schematic representation of experimental design for proteomics analysis. Myofibroblasts were induced for 24 h using a cytokine cocktail (TGF-β1, TNF-α, IL-1β, and IL-6) before co-culture with ET-eMSCs preconditioned with PGE_2_ (3 μM) in a transwell system. This setup enabled bidirectional exchange of soluble factors without direct cell contact. Samples were collected at two points: (i) time zero, including the conditioned medium from ET-eMSCs (secretome T0) and total proteins extracts from myofibroblasts (proteome T0), and (ii) after 48 h of co-culture, collecting the PGE_2_-conditioned medium containing myofibroblast secretions (secretome 48 h) and total protein extracts from myofibroblasts (proteome 48 h). Each sample was analyzed in triplicate. (Figure created with BioRender.com, accessed on 21 August 2024).

The fibroblasts were seeded at a density of 1 × 10^5^ cell/cm^2^ in 12-well flat-bottom dish in high-glucose DMEM (Sigma-Aldrich™ D6429) supplemented with 10% Fetal Bovine Serum (FSB) (Sigma-Aldrich™ F2442) and 1× Antibiotic-Antimycotic Solution (AAM) (Corning™ 30-004-CI). They were incubated for 24 h at 38 °C with 5% CO_2_, and after this time, the fibroblasts were washed with 1× PBS and transferred to a preinduction medium (high-glucose DMEM supplemented with 0.5% FBS and 1× AAM). The fibroblasts were then incubated for an additional 18 h at 38 °C with 5% CO_2_. After this period, 10 ng/mL each of TGF-β1 (code: 100-21-10UG), TNF-α (code: 300-01A-50UG), IL-6 (code: AF-200-06-20UG), and IL-1β (code: 200-01B-10UG) (all, Gibco™, Thermo Fisher Scientific Inc.) were added, and the fibroblasts were further incubated for 24 h at 38 °C with 5% CO_2_ (48 h in Figure 1). This incubation time induces the differentiation into myofibroblasts, according to previously published work of Wong et al., 2023 [24].

#### 2.1.3. Culture to ET-eMSC

ET-eMSCs were seeded in the upper chamber of a transwell system (0.4 μm pore size; SPLInsert™ Hanging 37012, SPL Life Sciences Co., Ltd., Pocheon-si, Republic of Korea) at a density of 6 × 10^4^ cells/cm^2^ in 2 mL of high-glucose DMEM supplemented with 10% FBS and 1× AAM. The cells were incubated under these culture conditions at 38 °C in a humidified atmosphere containing 5% CO_2_ for 48 h.

After 48 h, ET-eMSCs were transferred to a medium conditioned with 3 μM PGE_2_ (high-glucose DMEM supplemented with 1× AAM and 3 μM PGE_2_ (Cayman Chemical 14010, Ann Harbor, MI, USA) and incubated for 24 h (72 h in Figure 1).

#### 2.1.4. Co-Culture of Myofibroblasts and ET-eMSCs Preconditioned with PGE_2_

On day 4 (72 h in Figure 1), the induction medium was removed from the myofibroblast plates, and the myofibroblasts were washed twice with 1× PBS to eliminate any residual medium. The co-culture of myofibroblasts and ET-eMSCs preconditioned with 3 μM PGE_2_ was conducted using a transwell system, which allowed bidirectional exchange between cells seeded in the upper chamber and those in the lower chamber without direct physical contact. For the co-culture, the ET-eMSCs in the upper chamber were transferred to the well where the myofibroblasts were located at the bottom of the plate (lower chamber). One milliliter of PGE_2_-conditioned medium from ET-eMSCs was added to both chambers. Myofibroblast samples (obtained 24 h post-induction with the cytokine cocktail) and culture supernatants from ET-eMSCs (preconditioned for 24 h with PGE_2_) were collected at the start of the co-culture (proteome T0 and secretome T0, respectively) and again after 48 h (proteome 48 h and secretome 48 h, respectively). Each sampling was performed in triplicate.

The culture supernatant samples for secretome analysis were collected and supplemented with a Protease Inhibitor Cocktail (Cell Signaling Technology™, Boston, MA, USA) at a final concentration of 1×. Samples were rapidly frozen by immersion in liquid nitrogen before being stored at −80 °C until further processing. For the myofibroblast culture samples, the cell monolayer was harvested using cell scrapers, resuspended in 100 µL of commercial 1× PBS (Gibco 10010023, Thermo Fisher Scientific Inc.), supplemented with Protease Inhibitor Cocktail at a final concentration of 1×, snap frozen in liquid nitrogen and stored at −80 °C until processing. Figure 1 summarizes the experimental conditions.

### 2.2. Experiment 2: Canonical Protein Matching Databases and Relative Quantification of the Interaction Between Endometrial Myofibroblasts and PGE_2_-Conditioned ET-eMSCs Were Conducted Using the SILAC Protein Quantitation Kit (Trypsin)

**Figure 2:**
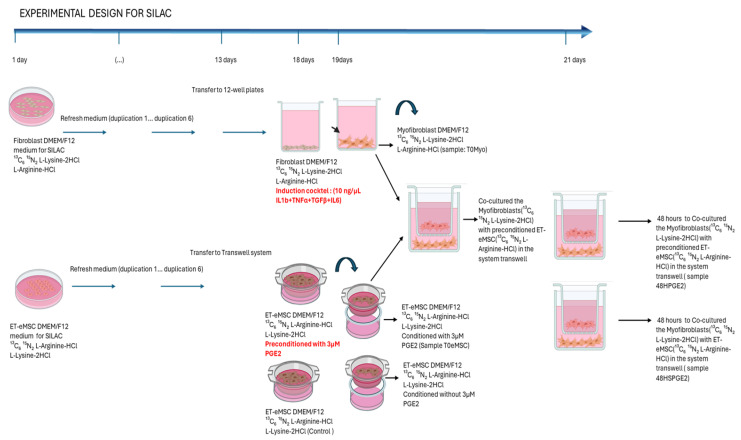
Schematic representation of experimental design employed SILAC in Co-culture Experiment. Co-cultures were maintained in conditioned SILAC medium until Day 21 to permit bidirectional molecular exchange. Samples were collected at two time points: (i) time zero, including the conditioned medium from ET-eMSCs (T0MSC) and supernatant from myofibroblasts (T0Myo), and (ii) after 48 h of co-culture, collecting the PGE_2_-conditioned medium containing myofibroblast and ET-eMSC secretions(48H-PGE_2_) and control group (48H-SPGE_2_). Each sample was analyzed in triplicate. (Figure created with BioRender.com, accessed on 14 November 2024).

This assay aimed to determine, within the co-culture supernatant of myofibroblasts and ET-eMSC (similar to the previous experiment), the specific proteins contributed by each cell isolates type. Using the SILAC Protein Quantitation Kit (Trypsin), two cell populations were cultured in media containing either light or heavy isotopic amino acids [20] (Figure 2).

For this assay, the SILAC Protein Quantitation Kit (Trypsin)—DMEM was used, which includes DMEM/F12 for SILAC; 13C_6 15N_2 L-Lysine-2HCl; L-Lysine-2HCl; 13C_6 15N_4 L-Arginine-HCl; L-Arginine-HCl and Dialyzed Fetal Bovine Serum(code: A33972 SILAC Protein Quantitation Kit (Trypsin)—DMEM; Pierce Biotechnology, Thermo Fisher Scientific Inc., Waltham, MA, USA). The SILAC medium was supplemented with 10% dialyzed FBS (supplied with the kit), 1× Antibiotic-Antimycotic Solution (AAM) (code: 30-004-CI; Corning™), and the manufacturer’s instructions for cell culture and protein labeling were followed. Fibroblasts were labeled with heavy isotopologues of lysine (13C_6 15N_2 L-Lysine-2HCl), and ET-eMSCs were labeled with heavy isotopologues of arginine (13C_6 15N_4 L-Arginine-HCl) (SILAC Protein Quantitation Kit (Trypsin)—DMEM A33972; Pierce Biotechnology, Thermo Fisher Scientific Inc.). Both cell populations were subcultured for seven population doublings, with the culture medium replaced every 48 h. Cell seeding density was adjusted to maintain active proliferation within the logarithmic growth phase, achieving approximately 90% confluence in 100 mm cell culture dishes.

On day thirteen, ET-eMSCs were seeded in the upper chamber of a transwell system (0.4 μm pore size; SPLInsert™ Hanging 37012; SPL Life Sciences Co., Ltd.) at a density of 6 × 10^4^ cells/cm^2^ in 2 mL of DMEM/F12 supplemented with 13C_6 15N_4 L-Arginine-HCl; 10% dialyzed FBS, and 1× AAM(in triplicate). The cells were incubated under these culture conditions at 38 °C in a humidified atmosphere containing 5% CO_2_ for 48 h.

The fibroblasts were then seeded at 1 × 10^5^ cells/cm^2^ in 12-well plates (in triplicate) in 1 mL of DMEM/F12 supplemented with 13C_6 15N_2 L-Lysine-2HCl; 10% dialyzed FBS, and 1× AAM. The cells were incubated under these culture conditions at 38 °C in a humidified atmosphere containing 5% CO_2_ for 48 h.

#### 2.2.1. Induction of Myofibroblasts from Endometrial Fibroblasts

After 24 h of culture, fibroblasts were washed with 1× PBS and incubated for 18 h at 38 °C with 5% CO_2_ in a preinduction medium (DMEM/F12 supplemented with 13C6 15N2 L-Lysine-2HCl; 0.5% dialyzed FBS, and 1× AAM). After this period, 10 ng/mL each of TGF-β1 (100-21-10UG), TNF-α (300-01A-50UG), IL-6 (AF-200-06-20UG), and IL-1β (200-01B-10UG) (all, Gibco™, Thermo Fisher Scientific Inc.) (induction cocktail) were added, and the fibroblasts were further incubated for 24 h at 38 °C with 5% CO_2_.

#### 2.2.2. ET-eMSC Preconditioning with PGE_2_

After 48 h of culture, ET-eMSCs were washed with 1× PBS and incubated for an additional 24 h at 38 °C with 5% CO_2_ in preconditioning medium (DMEM/F12 supplemented with 13C_6 15N_4 L-Arginine·HCl, 1× AAM, and 3 μM PGE_2_; Cayman Chemical 14010, Ann Arbor, MI, USA). For the control group, ET-eMSCs were maintained in medium containing DMEM/F12 supplemented with 13C_6 15N_4 L-Arginine·HCl and 1× AAM, without PGE_2_.

#### 2.2.3. Co-Cultures

On Day 19, the induction medium was collected from the myofibroblast cultures (sample T0MYO) and supplemented with a Protease Inhibitor Cocktail (Cell Signaling Technology™ 7012) at a final concentration of 1× and stored at −80 °C until further processing. Myofibroblasts were then rinsed twice with 1× PBS. The transwell inserts containing the preconditioned ET-eMSCs were placed into the myofibroblast plates, and 1 mL of conditioned medium (supplemented with 13C_6 15N_4 L-Arginine·HCl) was added to both the upper and lower chambers to enable bidirectional molecular exchange. The co-cultures were maintained for 48 h. As illustrated in Figure 2, at this time, T0MSC culture supernatant samples were taken, following the same procedure as the T0MYO sample.

Co-cultures were maintained for 48 h. At that time, culture supernatants were collected from the ET-eMSC preconditioned with PGE_2_ wells following the same protocol used for the T0MYO samples (Sample T0MSC; Figure 2). The culture supernatant samples for secretome analysis were collected, supplemented with a Protease Inhibitor Cocktail (Cell Signaling Technology™ 7012) at a final concentration of 1x, and stored at −80 °C until further processing.

The steps of protein extraction for spectrometry analysis, preparation for mass spectrometry (MS), and liquid chromatography-tandem mass spectrometry (LC-MS/MS) were similar to those described above.

### 2.3. Protein Extraction and Preparation for Mass Spectrometry

Protein extraction was performed using 100 µL of lysis buffer containing 50 mM HEPES (pH 8.0), 1% (*wt*/*vol*) Triton X-100, 1% (*vol*/*vol*) NP-40, 1% (*vol*/*vol*) Tween 20, 1% (*wt*/*vol*) sodium deoxycholate, 5 mM EDTA, 50 mM NaCl, 1% (*vol*/*vol*) glycerol, 1X Complete Protease Inhibitor, and 5 mM DTT. Samples were incubated for 30 min at 60 °C and homogenized by sonication for 2 min in 10 s cycles at 40% amplitude. Subsequently, proteins were alkylated with 20 mM iodoacetamide in 25 mM ammonium bicarbonate and incubated in the dark for 30 min at room temperature.

Protein purification was carried out using the chloroform/methanol precipitation method. Briefly, one volume of protein extract was mixed with five volumes of methanol, one volume of chloroform, and three volumes of Milli-Q water. After centrifugation at 15,000× *g* for 5 min, the protein disk was washed four times with 100% methanol and dried in a rotary concentrator overnight at 40 °C.

To ensure further purification and compatibility with digestion, the protein pellet was also subjected to cold acetone precipitation (5 volumes of −20 °C acetone, overnight at −80 °C). Pellets were centrifuged at 16,000× *g* for 15 min at 4 °C, washed three times with 80% cold acetone, and dried.

Protein pellets were then resuspended in 30 µL of 8 M urea in 25 mM ammonium bicarbonate. Reduction was performed with 20 mM DTT, followed by alkylation with 20 mM iodoacetamide under light-protected conditions. Samples were diluted 8-fold with 25 mM ammonium bicarbonate prior to enzymatic digestion.

Trypsin digestion was conducted at a 1:50 enzyme-to-protein ratio (*w*/*w*) for 16 h at 37 °C. The digestion reaction was quenched with 10% formic acid, and 200 ng of peptides were cleaned using disposable C18 Evotips columns (EVOSEP EVO2018, Biosystems, Odense, Denmark) according to the manufacturer’s instructions. 2.5. Liquid Chromatography—Tandem Mass Spectrometry.

The Evotips columns were deposited on an Evosep One (Evosep Biosystems) coupled to a timsTOF Pro 2 mass spectrometer (“Trapped Ion Mobility Spectrometry—Quadrupole Time of Flight Mass Spectrometer”, Bruker Daltonics) using an EVOSEP Performance column (15 cm × 150 µm, 1.5 µm beads ReproSil-Pur C18, EVOSEP Biosystems). Liquid chromatography was performed using the 30 SPD (“Samples per Day”) mode on all samples. The gradient used was 2% to 35% buffer B (0.1% formic acid in acetonitrile). The results collection was performed using Tims Control 2.0 software (Bruker Daltonics) under 10 PASEF cycles, with a mass range of 100–1700 *m*/*z*, capillary ionization of 1500 V, and a capillary temperature of 180 °C. The TOF frequency was set at 10 kHz with a resolution of 50,000 FWHM.

### 2.4. Protein Database Matching and Relative Quantification

The data obtained were analyzed using MSFragger v4.1 [25] on the Fragpipe v22.0 platform (https://fragpipe.nesvilab.org/, accessed on 3 October 2024) with the “default” workflow, on a data analysis server equipped with 48 cores and 512 GB of RAM. Mass tolerance parameters for precursors ranged from −20 to 20 PPM, and fragment mass tolerance was set to 40 PPM. Trypsin was used as the enzyme within the digestion options, employing a specific digestion mode with a maximum of 2 missed cleavages per peptide. The following post-translational modifications (PTMs) were applied: Carbamidomethylation of cysteine (fixed PTM), methionine oxidation (M), and N-terminal acetylation (variable PTMs). The database used was the proteome of Equus caballus (UP000002281), available at UniProt. An FDR < 1% was estimated using a decoy database. Additionally, a database of common contaminants in mass spectrometry was included for canonical protein matching analysis.

The spectral counts of matched to database entry canonical proteins were used to perform differential abundance analysis using the EdgeR R package v4 and the Limma package 3.46.0. Proteins with total spectral counts below 5 counts were excluded and presence in at least two of the three replicates, and TMM (Trimmed Mean of M-values) method was used to normalize. Differentially abundant canonical protein analysis was performed by fitting a general linear model with Poisson negative binomial distribution with FDR < 0.05. The results were plotted using the Volcano Plot and Heatmap functions in the ggplot2 R package. gProfiler2 R package was used for gene ontology (Biological Process, Cellular Component, Molecular Function and Reactome analysis).

Differentially abundant proteins were analyzed using Gene Ontology (GO) annotation, which included classifications into Biological Process (BP), Cellular Component (CC), and Molecular Function (MF) categories. The KEGG database (http://www.genome.jp/kegg, accessed on 17 November 2024) and the Reactome database (https://reactome.org, accessed on 17 November 2024) were used to categorize the identified proteins into their associated pathways. Additionally, the STRING database was used to examine the protein–protein interaction (PPI) networks, focusing on both physical and functional connections among the selected protein. Additionally, Enrichment Analysis of Fibrosis-Related Protein in Myofibroblasts and Secretome was performed using the FibroAtlas database (http://biokb.ncpsb.org/fibroatlas/, accessed on 5 October 2024). 

#### SILAC-Based Protein Quantification

Protein quantification by SILAC was performed using the IonQuant package (version 1.11.9). Three channels were defined: a “Heavy” channel (K8/R10 for lysine-8 and arginine-10) and a “Light” channel (K0/R0 for unlabeled lysine and arginine). From these isotopic labels, Heavy/Light (H/L) intensity ratios were calculated for each experimental sample, enabling relative quantification of canonical proteins according to their cellular origin. Three independent biological replicates were analyzed, and proteins were considered valid when a non-zero SILAC H/L ratio was quantified in at least two replicates with a minimum of two peptides detected.

The quantitative output files generated by FragPipe (https://fragpipe.nesvilab.org, accessed on 12 June 2025) were imported into R (v4.3.2). We first filtered to retain only proteins annotated to Equus caballus (UP000002281), available at UniProt, discarding any duplicates or low-quality samples. Using a custom R script, we extracted the intensities from the Light (K0/R0) and Heavy (K8/R10) channels and computed H/L ratios for every protein in each sample. The resulting data matrix was then reshaped and normalized using quantile normalization via functions in the Limma package (version 3.58.1).

Next, pairwise comparisons between experimental groups were performed by fitting a linear model in Limma and evaluating differential abundance with the eBayes method. Proteins with an adjusted *p*-value < 0.05 were flagged as differentially abundant. We also produced comprehensive tables listing all quantified canonical proteins, as well as those classified as differentially abundant (adjusted *p*-value < 0.05).

Finally, information from labeled peptides (modifications: K [+8.0142], R [+10.0083]) was integrated to assign the most likely cellular origin of each signal (eMSC, MYO, or both). This annotation was appended to the final abundance and differential abundance tables.

### 2.5. Bioinformatics Analysis for the SILAC Experiment

The quantified proteins were filtered using the non-parametric Mann–Whitney U-test, corrected for multiple testing using a Benjamini–Hochberg-adjusted *p*-value < 0.05, and a fold-change (FC) > 0.5 or <−0.5, to investigate further only those statistically significant and altered in fibrosis progression and drug-mediated slowdown. The comparisons made were as follows:

- Secretome at different times: secretome 48 h (MYO-ET-eMSC + PGE_2_) vs. secretome T0 (ET-eMSC + PGE_2_).
- Myofibroblast at different times: proteome 48 h vs. proteome T0.

The output of the string analyses shows only the top ten most significant processes or pathways for each analysis, selected based on the lowest FDR values and the highest number of associated proteins (gene count). The actual number and detailed description of pathways involved is displayed in Table S5 (Supplementary files). The size of the circles reflects the number of proteins associated with each term. At the same time, the color gradient indicates the level of statistical significance (FDR). These enriched terms highlight biological processes and functional pathways closely linked to cellular interactions during co-culture, facilitating a focused interpretation of the most relevant results.

Activities from 2.3 to 2.5 were subcontracted to Melisa Institute, San Pedro de la Paz, Concepción, Chile and the protocols for treatment and analyses of the samples were those used by the provider of the paid service.

## 3. Results

### 3.1. Experiment 1: Analysis of Differential Protein Abundance During Co-Culture of Myofibroblasts with PGE_2_-Preconditioned ET-eMSC

To investigate the anti-fibrotic response induced in myofibroblasts by interaction with PGE_2_-preconditioned ET-eMSC, a proteomic analysis was performed using LC-MS/MS. Proteomic profiles were evaluated by collecting samples from the secretomes of ET-eMSC and myofibroblasts at various time points.

For ET-eMSC + PGE_2_, secretome samples were collected at two time points: at the initial time (secretome 0 h) and after 48 h of co-culture with myofibroblasts (secretome 48 h) (Figure 1). For myofibroblasts, samples were collected before co-culture (proteome 0 h) and after 48 h of co-culture with ET-eMSC + PGE_2_ (proteome 48 h). This experimental design enabled the analysis and comparison of proteomic changes in both cell populations during interaction using a transwell system.

Protein extraction was performed on the secretome of both ET-eMSC and myofibroblast samples, and the resulting mass spectrometry data were analyzed using the FragPipe computational platform with the MSFragger search engine, employing the Equus caballus reference proteome (UniProt ID: UP000002281) for protein matching databases.

**Figure 3:**
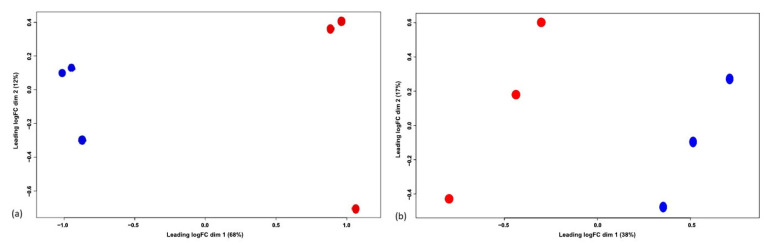
Principal Component Analysis (PCA) of the proteomic profiles. (**a**) PCA of the secretome of ET-eMSC samples shows a clear separation between the 0 h (blue) and 48 h (red) conditions, highlighting distinct biological differences after preconditioning with PGE_2_. (**b**) PCA of the proteome of myofibroblast samples also demonstrates separation between 0 h (blue) and 48 h (red) conditions, indicating proteomic changes induced by the co-culture.

A Principal Component Analysis (PCA) revealed a clear separation between 0 h and 48 h samples in both the secretome of ET-eMSC (Figure 3a) and myofibroblasts (Figure 3b), highlighting distinct biological differences between the two points.

**Figure 4:**
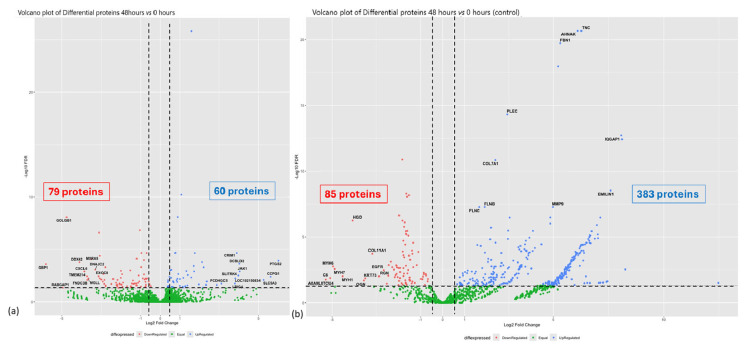
Volcano plots showing differential protein abundance in myofibroblasts and secretome of ET-eMSC samples after co-culture with ET-eMSC for 48 h compared to 0 h. (**a**) Volcano plot depicting protein abundance changes in myofibroblasts. Key proteins of interest are labeled. (**b**) Volcano plot for the secretome of ET-eMSC samples. In both graphs, red indicates low-abundance proteins, and green indicates high-abundance proteins. Notable proteins with high fold changes are highlighted. Dashed lines represent significance thresholds (FDR < 0.05 and |logFC| > 0.5.

A total of 3861 quantifiable proteins that matched the database were detected in the myofibroblast samples, and 1410 proteins in the secretome of ET-eMSC samples. In the myofibroblasts, 139 proteins showed significant differential abundance when comparing the proteome at 0 h and the proteome at 48 h of co-culture with ET-eMSC. Among these, 79 proteins decreased in abundance (logFC < −0.5, FDR < 0.05), while 60 proteins were increased in abundance (logFC > 0.5, FDR < 0.05), as visualized in the volcano plot diagram in Figure 4a.

For the secretome of ET-eMSC, out of the 1410 quantifiable proteins, 468 proteins exhibited significant differential abundance after 48 h of co-culture compared to time zero. Among these, 85 proteins were low abundance (logFC < −0.5, FDR < 0.05), and 383 proteins were increased abundance (logFC > 0.5, FDR < 0.05), as represented in Figure 4b. This analysis highlights the proteomic changes in both myofibroblasts and the secretome of ET-eMSC under co-culture conditions. Tables S1 and S2 (Additional Files) provide the lists of differentially abundant proteins.

Additionally, gene functional and physical network reconstruction was performed using STRING to visualize the interactions between low and high abundance proteins in myofibroblasts and the secretome of ET-eMSC under co-culture conditions (Supplementary Figure S1). The constructed networks for both low and high abundance proteins in myofibroblasts showed significant enrichment of protein–protein interaction compared to random protein sets (*p* = 1.12 × 10^−11^ and *p* = 1.33 × 10^−6^, respectively). For the secretome of ET-eMSC, the enrichment of low and high abundance proteins was highly significant (*p* < 10^−16^).

Differentially abundant proteins were analyzed, revealing that, for low-abundance proteins, ten significant clusters were identified through protein–protein interaction (PPI) network analysis using MCL clustering. Similarly, ten significant clusters were also identified among the high-abundance proteins (Table S3, Additional Files). In parallel, analysis of the ET-eMSC secretome revealed 12 clusters among the low-abundance proteins and 78 clusters among the high-abundance proteins (Table S4, Additional Files).

#### 3.1.1. Gene Ontology and Reactome Pathways Enrichment of DAPs in the Secretome of ET-eMSC

To better understand the functions and pathways associated with the DAPs, GO and Reactome pathway enrichment analyses were performed (Supplementary Figure S2 and Table S5, Additional Files). The study revealed that proteins of high abundance were linked to 127 enrichment terms for biological processes (BP). The top ten most significant GO terms included supramolecular fiber organization, protein folding, actin cytoskeleton organization, ECM organization, vesicle-mediated transport from the endoplasmic reticulum to the Golgi, cellular detoxification, and regulation of RNA splicing (Supplementary Figure S2a and Table S5, Additional Files). In contrast, low-abundance proteins were associated with 48 enrichment terms. The top ten terms were related to the negative regulation of endopeptidase activity, proteolysis, protein metabolism, catalytic activity, blood coagulation, and supramolecular fiber organization (Supplementary Figure S2b and Table S5, Additional Files).

In terms of molecular function (MF), the high-abundance proteins were associated with 58 enrichment terms. The top ten most significant terms included ATP-dependent protein folding chaperones, actin binding, RNA binding, ECM structural constituents, aminoacyl-tRNA ligase activity, monosaccharide binding, hydrolase activity, actin filament binding, and small molecule binding (Supplementary Figure S2c and Table S5, Additional Files). Conversely, low-abundance proteins were associated with 13 enrichment terms, which included structural constituents of the skin epidermis, endopeptidase inhibitor activity, peptidase regulator activity, serine-type endopeptidase inhibitor activity, glycosaminoglycan binding, enzyme regulator activity, structural molecule activity, lipid binding, epidermal growth factor receptor activity, and metal ion binding (Supplementary Figure S2d and Table S5, Additional Files).

For cellular components (CC), high-abundance proteins were enriched in 76 terms, including locations such as the chaperonin-containing T-complex, coated vesicle membranes, vesicle coats, collagen-containing extracellular matrix, basement membrane, endopeptidase complex, actin cytoskeleton, and proteasome accessory complex (Supplementary Figure S2e and Table S5, Additional Files). In contrast, low-abundance proteins were associated with cellular components such as extracellular space, intermediate filaments, keratin filaments, supramolecular fibers, myofibrils, polymeric cytoskeletal fibers, cornified envelopes, sarcomeres, and the membrane attack complex (Supplementary Figure S2f and Table S5, Additional Files).

In the KEGG analysis of the secretome, proteins with decreased abundance at 48 h were enriched in immune and coagulation functions, especially within the complement and coagulation cascades pathway, as well as infection-related pathways such as *Staphylococcus aureus* and autoimmune diseases like systemic lupus erythematosus. Conversely, secreted proteins with increased abundance at 48 h showed broad and diverse functional enrichments. Notably, pathways such as focal adhesion, protein processing in the endoplasmic reticulum, sugar and protein metabolism, and cytoskeleton organization were enriched. There was also significant involvement in pathways related to neurodegenerative diseases, immune responses, and intracellular signaling, with key proteins including integrins, collagens, proteasome components, and vesicular transport elements, indicating an active cellular response (Supplementary Figure S2g,h, and Table S5, Additional Files).

Reactome pathway enrichment analysis revealed that high abundance proteins were involved in 194 enriched terms, which included pathways such as TriC/CCT-mediated protein folding, ECM organization, neutrophil degranulation, AUF1 (hnRNP D0)-mediated mRNA destabilization, cytosolic tRNA aminoacylation, ER-to-Golgi anterograde transport, regulation of mRNA stability by AU-rich elements, innate immune responses, and cellular stress responses (Supplementary Figure S2i and Table S5, Additional Files). In contrast, low abundance proteins were associated with 30 enrichment terms, including pathways such as insulin-like growth factor (IGF) transport and uptake by IGFBPs, formation of the cornified envelope, post-translational protein phosphorylation, platelet degranulation, keratinization, fibrin clot formation, platelet activation and aggregation, hemostasis, and regulation of the complement cascade (Supplementary Figure S2j and Table S5, Additional Files).

#### 3.1.2. Gene Ontology and Reactome Pathways Enrichment of DAPs in the Myofibroblast

GO analysis comparing myofibroblasts co-cultured for 48 h with those at 0 h revealed significant enrichment terms across biological processes, molecular functions, and cellular components (Supplementary Figure S3 and Table S5, Additional Files). Specifically, 11 significant terms were identified for BP (3 associated with low-abundance proteins and 8 with high-abundance proteins (Supplementary Figure S3a and Figure S3b, respectively, and Table S5, Additional Files), 12 for MF (1 linked to low-abundance proteins and 10 to high-abundance proteins (Supplementary Figure S3c and Figure S3d, respectively, and Table S5, Additional Files), and 15 for CC (5 related to low-abundance proteins and 10 to high-abundance proteins; Supplementary Figure S3e and Figure S3f, respectively, and Table S5, Additional Files).

In BP, the low-abundance proteins were linked to ECM organization, circulatory system development, and collagen fibril organization. Conversely, the top 10 significant GO terms associated with high-abundance proteins included regulation of multicellular organismal processes, angiogenesis, cell adhesion, cell migration, positive regulation of locomotion, response to organic substances, regulation of cell migration, and positive regulation of cell migration. (Supplementary Figure S3a,b and Table S5, Additional Files).

For MF, the only term identified among low-abundance proteins was ECM structural constituent (Supplementary Figure S3c). In contrast, high-abundance proteins were linked to glycosaminoglycan binding, heparin binding, proteoglycan binding, growth factor binding, carbohydrate derivative binding, integrin binding, modified amino acid binding, protein-containing complex binding, signaling receptor binding, syndecan binding, and phosphatidylserine binding. (Supplementary Figure S3c,d and Table S5, Additional Files).

In CC, the enriched terms for low-abundance proteins included fibrillary collagen trimer, collagen-containing extracellular matrix, collagen trimer, extracellular matrix, and basement membrane. In contrast, high-abundance proteins were enriched in the extracellular region, cell surface, cell periphery, extracellular space, extracellular matrix, plasma membrane, endomembrane system, cytoplasmic vesicle, and nuclear envelope lumen. (Supplementary Figure S3e,f, and Table S5, Additional Files).

In the KEGG analysis of fibroblasts functional enrichment of proteins with differential abundance between 48 h and baseline (0 h) revealed biologically meaningful pathways associated with myofibroblast responses, influenced by co-culture with ET-eMSC + PGE_2_. In myofibroblasts, proteins with decreased abundance were significantly linked to pathways such as ECM-receptor interaction (FDR = 0.0036), AGE-RAGE signaling in diabetic complications (FDR = 0.0420), and protein digestion and absorption (FDR = 0.0420), mainly involving collagens and extracellular matrix proteins like COL1A1, COL1A2, COL2A1, and THBS2. (Supplementary Figure S3g and Table S5, Additional Files). No significant pathway enrichments were found among proteins with increased abundance in myofibroblasts in KEGG.

Reactome pathway analysis identified 14 enriched pathways for low-abundance proteins, including non-integrin membrane-ECM interactions, assembly of collagen fibrils and other multimeric structures, ECM proteoglycans, collagen degradation, ECM degradation, collagen chain trimerization, signaling by PDGF, ECM organization, collagen formation, and integrin cell surface interactions. Notably, no significant Reactome-enriched pathways were identified for proteins with increased abundance (Supplementary Figure S3h and Table S5, Additional Files).

#### 3.1.3. Enrichment Analysis of Fibrosis-Related Proteins in Myofibroblasts and Secretome of ET-eMSC

Enrichment analysis was performed using the fibrosis-related gene list from Liu et al. to examine proteins involved in fibrosis regulation [26]. Quantifiable proteins were analyzed in both myofibroblasts and the culture supernatant (secretome) of ET-eMSC samples. In the secretome of ET-eMSC, 137 fibrosis-related proteins matched databases, with 33 showing increased abundance and 15 showing decreased abundance (Table S6, Additional Files). In myofibroblasts, 212 proteins related to fibrotic processes matched the FibroAtlas database, among which 11 had significantly low abundance and nine had high abundance (Table S7, Additional Files). 

The abundance of proteins related to the fibrotic process in the secretome of ET-eMSC and myofibroblasts under experimental conditions was analyzed using an assembly algorithm, which revealed two distinct branches: A (high abundance proteins) and B (low abundance proteins). This algorithm also allowed the visualization of protein abundance changes in response to the interaction between myofibroblasts and ET-eMSC + PGE_2_ at both myofibroblast and secretome levels.

**Figure 5:**
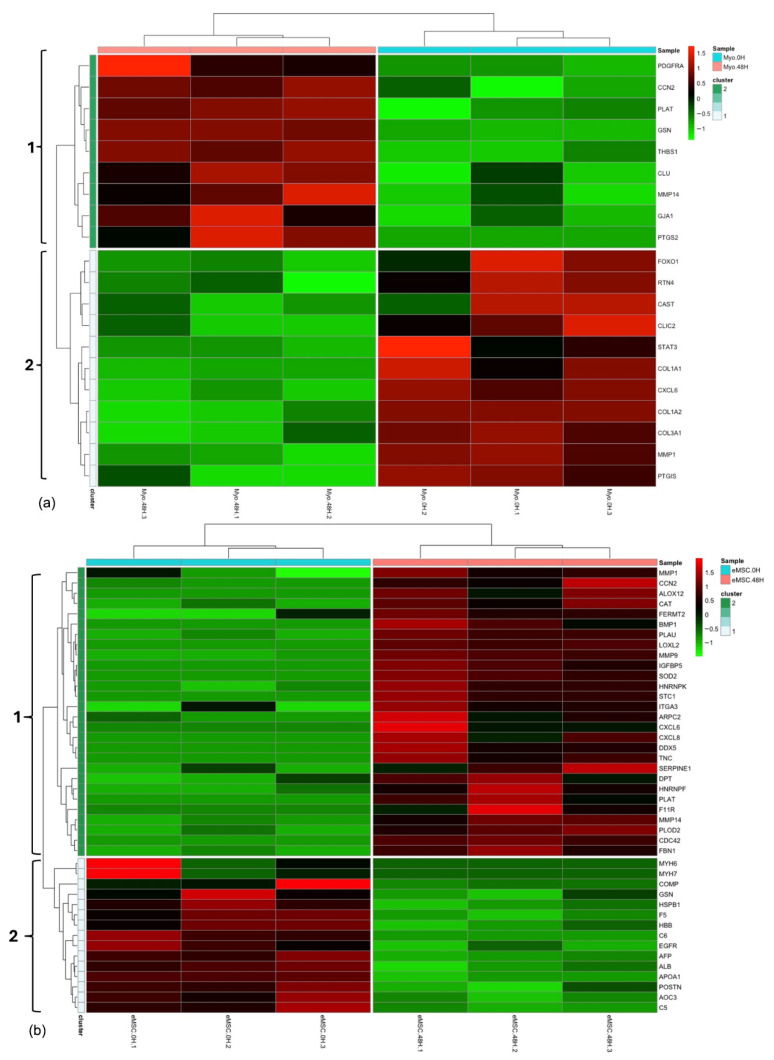
Differential abundance heatmaps of fibrosis-related proteins in myofibroblasts and the secretome of ET-eMSC samples. (**a**) The heatmap illustrates the abundance profiles of differentially abundant fibrosis-related proteins in myofibroblast samples co-cultured with ET-eMSC + PGE_2_ at two time points: 0 h (Myo.0H) and 48 h (Myo.48H). (**b**) The heatmap depicts DAPs in secretome of ET-eMSC samples from ET-eMSC preconditioned with PGE_2_ (eMSC.0H) and after 48 h of co-culture with myofibroblasts (eMSC.48H). The color scale represents changes in protein abundance, with red indicating upregulation, green indicating downregulation, and black representing minimal or no change.

The heatmap (Figure 5a) showed the abundance profiles of differentially abundant fibrosis-related proteins in myofibroblast samples at two time points: 0 h (Myo. 0H) and 48 h (Myo. 48H) of co-culture with ET-eMSC + PGE_2_. The color scale indicated changes in abundance, with red signaling high protein levels, green representing low levels, and black showing minimal or no change. Hierarchical clustering identified two distinct groups. Cluster 1, on the left panel, included proteins that mostly increased in abundance at 48 h, such as Thrombospondin 1 (THBS1), Prostaglandin G/H synthase 2 (PTGS2), Platelet-derived growth factor receptor alpha (PDGFRα), Matrix metallopeptidase 14 (MMP14), Clusterin alpha chain (CLU), Plasminogen activator (PLAT), Cellular communication network factor 2 (CCN2/CTGF), and Gap junction protein (GJA1), all linked to pro-fibrotic processes. Conversely, Cluster 2 contained proteins that significantly decreased at 48 h, including collagen type I alpha 1 and 2 (COL1A1, COL1A2), and collagen type III alpha 1 (COL3A1), which confirmed the PCR results. Additional decreases were also seen in Matrix metallopeptidase (MMP1), Prostacyclin synthase (PTGIS), Signal transducer and activator of transcription (STAT3), Fork head box protein O1 (FOXO1), and C-X-C motif chemokine 6 (CXCL6).

The heatmap (Figure 5b) presents the analysis of differentially abundant proteins in secretome samples from ET-eMSC preconditioned with PGE_2_ (eMSC.0H) and after 48 h of co-culture with myofibroblasts (eMSC.48H). Hierarchical clustering revealed two distinct clusters, with high-abundance proteins displayed in red and low-abundance proteins in green.

In the eMSC.0H group, proteins involved in complement and coagulation cascade pathways, such as C5, C6, and F5, were highly abundant. However, after 48 h of co-culture (eMSC.48H), these proteins exhibited a notable decrease in abundance, indicating reduced protein levels. Conversely, proteins such as MMP-9, MMP-1, and MMP-14, involved in collagen catabolic processes, as well as sarcoglycan alpha (SGCA), lysyl oxidase homolog (LOXL2), junctional adhesion molecule A (F11R), CCN2, PLAT, Urokinase-type plasminogen activator (PLAU), and Interleukin-8 (CXCL8), related to cell migration, and others like gelsolin (GSN), FERM domain containing kindlin 2 (FERMT2), and Arp2/3 complex 34 kDa subunit (ARPC2), associated with actin filament organization, were significantly increased in abundance. These findings indicated a dynamic response of the secretome of ET-eMSC during its interaction with myofibroblasts.

### 3.2. Experiment 2: SILAC Proteomic Analyses of Myofibroblasts Co-Cultured with Equine Endometrial-Derived MSCs (ET-eMSCs) Preconditioned with PGE_2_ Reveal Bidirectional Interchange Transfer of Proteins

To examine each cell type’s role in the overall secretome, an assay was developed using stable isotope labeling by amino acids in cell culture (SILAC). This method enabled us to determine the source of proteins in the secretome based on the molecular weight of the light or heavy amino acids incorporated during cell growth. For this study, a group of ET-eMSCs that had not been preconditioned with PGE_2_ was included (Figure 2).

**Figure 6:**
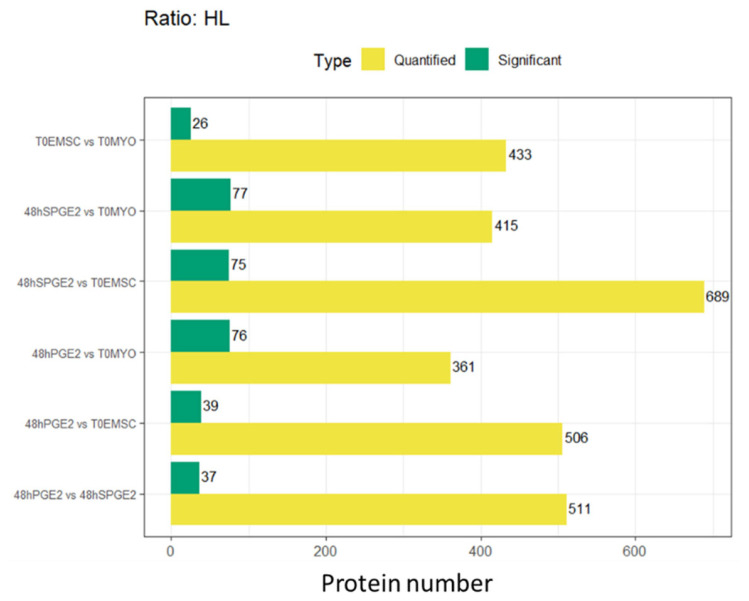
Comparison of the total number of quantified proteins (yellow bars) and the number of significantly differentially abundant proteins (green bars) for each contrast evaluated using the Heavy/Light (H/L) SILAC ratio.

Fibroblasts were cultured in media containing heavy isotopologues of lysine, and ET-eMSC were cultured in media containing heavy isotopologues of arginine to allow for >98.5% incorporation (Figure S4, Additional Files). Liquid chromatography-tandem mass spectrometry (LC-MS/MS) enabled finding heavy-labeled proteins within the secretome of co-cultures and individual secretomes for Myofibroblasts and ET-eMSC (Figure 6 and Table S8, Additional Files).

In the T0EMSC versus T0MYO comparison, 433 proteins were quantified, with 26 found to be significantly more abundant. For the 48Hspge_2_ versus T0MYO contrast, 415 proteins were quantified, and 77 showed significant changes. In the 48hSPGE_2_ versus T0EMSC analysis, a total of 689 proteins were quantified, and 75 were significantly regulated. The 48hPGE_2_ versus T0MYO contrast yielded 361 quantified proteins, with 76 displaying significant regulation. In the 48hPGE_2_ versus T0EMSC comparison, 506 proteins were quantified, and 39 were substantially regulated. Finally, the 48hPGE_2_ versus 48hSPGE_2_ contrast demonstrated 511 quantified proteins, with 37 showing statistically significant differences.

After identifying the proteins matching databases present in the secretome of the different experimental groups, we conducted a targeted analysis of those proteins that had previously been associated with modulating the fibrotic process. The focus was placed on MMPs, their inhibitors, cytokines, and fibrosis markers, as indicated in Table 1. We also included proteins that, although not differentially abundant in the previous experiment, held significant biological relevance (labeled “equal” in Table 1).

Notably, MMP-1, MMP-2, and MMP-9 were detected across all groups and were traced to both ET eMSCs and myofibroblasts, whereas MMP14 appeared in the 48 h PGE_2_ and 48 h sPGE_2_ groups and T0MYO, but not in T0MSC. TIMP1 and TIMP2, as well as THBS1 and THBS2, were present in all samples and were attributed to both cell types.

The matrix-remodeling protein MXR5 was detected in the 48 h PGE_2_, 48 h sPGE_2_, and T0MYO groups from both populations, whereas MXR8 was exclusive to the 48 h PGE_2_ and 48 h sPGE_2_ groups and originated only from ET eMSCs. The chemokines CXCL6 and CXCL8 were identified in the secretomes of the 48 h PGE_2_ and 48 h sPGE_2_ groups, with CXCL6 also appearing in T0MYO. CXCL6 was secreted solely by myofibroblasts, whereas CXCL8 originated from both ET eMSCs and myofibroblasts.

Finally, fibrosis markers CCN2 and TGF-β1 were detected in the 48 h sPGE_2_ secretome and myofibroblast samples, produced by both cell populations. This SILAC-based approach allowed precise protein attribution and enabled a comprehensive evaluation of the cellular interactions underlying the fibrotic phenotype.

## 4. Discussion

The present study shows the intricate molecular dialog between endometrial myofibroblasts and equine endometrium-derived mesenchymal stem cells in an in vitro model. It evaluates the potentialities of ET-eMSC preconditioned with PGE_2_ and their secretome for the treatment of endometrosis. The results obtained in the present study demonstrated that ET-eMSCs preconditioned with PGE_2_ induced partial reversal of the myofibroblastic phenotype. Specifically, the secretome of PGE_2_-preconditioned ET-eMSCs contained soluble factors that attenuated the abundance of pro-fibrotic markers in myofibroblasts and promoted a phenotype characterized by reduced contractile capacity and decreased synthesis of fibrotic extracellular matrix. Reciprocally, the myofibroblasts secreted mediators that enhanced immunomodulatory functions and viability maintenance in PGE_2_-preconditioned ET-eMSCs. This bidirectional paracrine modulation mechanism suggests the existence of a dynamic crosstalk that has favored the restoration of endometrial homeostasis in the co-culture model, which has not been described for endometrial fibrosis to date.

Label-free quantitative proteomic analysis revealed significant modulation of fibrosis-associated markers in myofibroblasts upon co-culture with PGE_2_-preconditioned ET-eMSC, which supports the hypothesis that paracrine interactions mediate a bidirectional regulatory mechanism. This crosstalk not only promoted antifibrotic signaling in myofibroblasts but also reinforced the therapeutic potential of the ET-eMSC secretome, as previously demonstrated by our research group [15,16,17,18].

When endometrial myofibroblasts were co-cultured with PGE_2_-preconditioned ET-eMSCs for 48 h, an attenuation of the fibrotic phenotype was observed, which was associated with a significant inhibition of fibrillar matrix proteins (COL1A1, COL1A2, COL3A1, COL5A1, COL2A1, COL7A1) and their scaffolds (fibronectin, versican). This finding was deemed highly relevant because previous studies had associated high ECM deposition and accumulation in endometrial tissue, particularly surrounding endometrial glands and fibrous nests, with loss of secretory function and exacerbation of the fibrotic process [27,28,29,30,31,32].

Although the role of MMP14 in the development of endometrosis had not been described in detail, it had been reported that MMP-14 not only degraded type I collagen directly but also activated pro-MMP-2 and pro-MMP-9, thereby amplifying the pericellular degradation of the ECM [30,33]. Another important finding was that a significant decrease in MMP1 levels was observed in the myofibroblasts after 48 h of co-culture with PGE_2_-preconditioned ET-eMSCs, which may have been associated with the reduction in collagen concentration values.

Previous work has demonstrated that PGE_2_ can reverse TGFβ1–induced myofibroblast differentiation, resulting in a significant, dose- and time-dependent decrease in collagen I mRNA and protein in lung, skin, and dermal fibroblast systems [34,35], but not in the endometrium. In dermal fibroblasts treated with TGF-β1, PGE_2_ reduced scar-related collagen synthesis through EP_2_–cAMP signaling and by shifting the MMP/TIMP balance. Likewise, in aged skin organ cultures, elevated levels of PGE_2_ were correlated with a diminished abundance of procollagen I [36,37]. Similar antifibrotic effects on collagen accumulation have been reported in tendon fibroblasts and fibrotic rat liver slices [38].

Our results revealed that the abundance of profibrotic signaling mediators, specifically STAT3 and FOXO1, decreased. STAT3 is a well-known downstream effector of TGF-β1 and IL-6 that drives the myofibroblastic phenotype; its transcriptional inhibition contributed to reduced fibrogenic activation [39,40]. These findings support the idea that PGE_2_ modulates TGF-β1 signaling indirectly via second messengers. By attenuating STAT3 activity, PGE_2_ interrupted the subsequent transcription of profibrotic and proliferative genes, positioning PGE_2_ as a negative feedback regulator within JAK/STAT-driven fibrotic pathways. Consistent with this, Lee et al. reported that PGE_2_ directly suppressed STAT3 activity in breast cancer cells and xenograft models [41].

In this study JAK1 was upregulated, its activation may have been associated with feedback pathways that modulated the EP receptor antiproliferative axis in fibroblasts, particularly when accompanied by a significant increase in Prostaglandin-endoperoxide synthase 2 (PTGS2/COX-2) production. PTGS2 was identified as the rate-limiting enzyme in PGE_2_ synthesis and was responsible for catalyzing prostaglandin production, thereby reinforcing the antifibrotic loop by amplifying the PGE_2_ signal [42]. This loop illustrates how the positive regulation of JAK1 and PGE_2_ production interacts to fine-tune STAT3 activities, with potential implications for controlling fibrotic progression through targeted modulation of these signaling nodes.

These facts lead us to hypothesize that upregulation of JAK1 enhances prostaglandin E_2_ (PGE_2_) synthesis via STAT3-mediated induction of PTGS2, while elevated PGE_2_ feeds back to suppress STAT3 signaling. Together, these dynamics established a regulatory loop in which JAK1 was upregulated, driving PGE_2_ up, and PGE_2_, in turn, downregulated STAT3.

After 48 h of co-culture, lumican (LUM), thrombospondin-1 (THBS1), CCN1, and CCN2 were markedly overabundant in myofibroblast samples, indicating that the ECM had been reorganized into a more orderly and less rigid structure. Lumican regulated the diameter and spacing of type I collagen fibrils, thereby preventing their aggregation into thick bundles and maintaining a balanced fibrillar network [43,44]. Thrombospondin-1 activates the latent form of TGF-β1 and, through its binding to matrix metalloproteases such as MMP-14, enables the focal anchoring of these enzymes within the matrix, thereby promoting controlled, less pathological remodeling [45].

In a regulated context such as the secretome derived from MSCs preconditioned with PGE_2_ this localized effect could effectively dismantle excess collagen and reduce tissue stiffness Meanwhile, CCN2, whose abundance was upregulated by TGF-β1, functioned as a cofactor that amplified profibrotic signaling, stimulating myofibroblast activation, cellular proliferation, and collagen deposition, as previously studied [46,47].

It seems plausible that the antifibrotic effect observed in myofibroblast samples co-cultured for 48 h with ET-eMSCs preconditioned with PGE_2_ is mediated by molecules secreted into the culture medium, and that there is a bidirectional exchange between the two cell types. Differentially abundant proteins related to fibrotic processes were identified at the secretome level.

In the secretome samples, a decreased abundance was observed for proteins involved in scaffolding and fibrotic markers (such as periostin and cartilage oligomeric matrix protein [COMP]), as well as proteins associated with cytoskeletal dynamics and cellular stress responses (including gelsolin, heat shock protein β1, and myosin heavy chains). The reduction in Periostin and COMP suggests the dismantling of rigid ECM bridges [48,49,50]. Likewise, lower levels of coagulation factors and complement components may indicate a less inflammatory and less thrombogenic milieu. The downregulation of cytoskeletal and stress-related proteins implied that myofibroblast activation pathways were probably attenuated.

The high abundance protein in the secretome were related to several functional groups: matricellular organizers (tenascin-C, fibrillin-1, CCN2/CTGF, dermatopontin); matrix proteases and their regulators (MMP-1, MMP-9, MMP-14, Urokinase-type plasminogen activator [PLAU], and its inhibitor PAI-1 [SERPINE1]); collagen-modifying enzymes (procollagen-lysine 5-dioxygenase, Lysyl oxidase); antioxidant and homeostatic factors (superoxide dismutase, catalase, arachidonate 12-lipoxygenase); cell adhesion and cytoskeletal remodelers (integrin α3, kindlin-2, the Arp2/3 complex, JAM-A); growth-factor modulators and chemokines (IGFBP-5, CXCL6, CXCL8, stanniocalcin-1).

At the secretome level, matrix-associated proteins reshaped the ECM scaffold to favor elasticity and maintain controlled stiffness. Tenascin-C has been shown to modulate the ECM architecture and mechanotransduction, thereby preventing excessive rigidity by influencing fibroblast behavior in fibrotic contexts [51,52]. Fibrillin assemblies impart elasticity and resilience to connective tissues while sequestering growth factors that regulate ECM remodeling [53]. CCN2 (connective tissue growth factor) functioned as a versatile matricellular factor, regulating adhesion, proliferation, and ECM synthesis in a context-dependent manner and thereby influencing matrix organization without invariably driving uncontrolled stiffening. Dermatopontin regulates collagen and fibronectin fibrillogenesis, promoting balanced fibril formation that supports tissue plasticity rather than pathological stiffening [54].

Our results showed that proteases and their regulators form a finely tuned degradative network that enables localized collagen breakdown in myofibroblasts without excessive matrix loss. Specifically, matrix metalloproteases such as MMP-1, MMP-9, and MMP-14, along with the urokinase-type plasminogen activator system (uPA/PLAU) and its inhibitor, PAI-1 (SERPINE1), play central roles in regulating ECM turnover in fibrotic models. Their coordinated abundance limits pathological collagen accumulation while preventing unchecked degradation. Likewise, the balance between plasminogen activators and PAI-1 maintains tissue homeostasis by regulating plasmin- and MMP-mediated proteolysis, with dysregulation leading to either fibrosis or excessive matrix loss, depending on the context, as reported by other authors [55,56].

The upregulation of chemokines CXCL6 and CXCL8 was observed in the secretome obtained after 48 h of co-culture between myofibroblasts and ET-eMSc preconditioning with PGE_2_. CXCL6 had been shown to recruit neutrophils and modulate immune cell trafficking in inflammatory settings, and its neutralization had reduced lung inflammation and fibrosis in experimental models [57]. CXCL8 was implicated in recruiting neutrophils and monocytes to fibrotic lesions and in promoting fibroblast activation via CXCR1/2 signaling. Elevated CXCL8 levels were reported in pulmonary and bone marrow fibrosis and were associated with disease progression [58]. Thus, the observed increase in CXCL6 and CXCL8 supported a modulatory role in the fibrotic process, consistent with prior findings that these chemokines were upregulated in fibrotic microenvironments.

During Experiment 1, a comparative analysis of the proteomic profile of matrix metalloproteinases (MMPs) and the profile detected in the secretome obtained after 48 h of co-culture of myofibroblasts and ET-eMSCs in a transwell system was performed. This analysis revealed a differential modulation of MMPs in the secretome compared to the proteome, accompanied by a significant reduction in type I and type III collagen concentrations in myofibroblasts. At the same time, a restructuring of the ECM and alterations in the cytoskeletal organization of the myofibroblasts were observed, indicating that the secretome derived from the co-culture influenced the recovery of tissue homeostasis in the experimental model.

The use of SILAC was instrumental in this study, as it enabled the precise differentiation of the cellular origin of secreted proteins within our co-culture model. This approach provided superior quantitative accuracy and reproducibility compared to traditional or label-free proteomics techniques. Consequently, SILAC facilitated a rigorous, cell-specific analysis of proteolytic enzymes, inhibitors, cytokines, and matrix remodeling markers -key components of the fibrotic phenotype, thereby significantly enhancing the depth and reliability of our results.

By employing SILAC, it was confirmed that MMP-1, MMP-2, and MMP-9 were produced by both ET eMSCs and myofibroblasts, in agreement with the findings from Experiment 1. This result was particularly significant because it demonstrated that endometrial myofibroblasts secreted MMP-14, highlighting its potential role in activating other MMPs and sustaining tissue homeostasis. Likewise, the metalloproteinase inhibitors TIMP-1 and TIMP-2 were detected under all experimental conditions, reinforcing their essential role in regulating proteolytic activity. The presence of thrombospondins THBS1 and THBS2 across all cell groups further corroborated their classical function in mediating cell–cell and cell–matrix communication [59].

Regarding matrix-remodeling proteins, MXRA5 and MXRA8 displayed distinct abundance patterns: MXRA5 was identified in the 48 h PGE_2_, 48 h sPGE_2_, and T0MYO groups and was secreted by both cell types, consistent with its documented role in ECM assembly, collagen organization, and anti-inflammatory and anti-fibrotic functions [60,61]. Conversely, MXRA8 was found exclusively in the 48 h PGE_2_ and 48 h sPGE_2_ groups and was produced solely by ET eMSCs, what is in line with its known function in cellular adhesion and ECM remodeling [62,63]. Chemokine CXCL6 was observed only in the secretomes of myofibroblasts from the 48h PGE_2_, 48 h sPGE_2_, and T0MYO groups, suggesting a specific induction linked to the myofibroblast phenotype, which corroborates previous reports [64,65]. In contrast, CXCL8 was detected in both cell types under the same conditions, supporting its pro-inflammatory role and potential involvement in the activation of myofibroblasts.

The results for CCN2 (CTGF) were inconsistent when compared to those from Experiment 1, as this protein was not detected in the 48 h PGE_2_-conditioned secretome samples during the SILAC-based Experiment 2. Nevertheless, CCN2 was identified in the time-zero myofibroblast secretome and in the 48 h sPGE_2_ secretome, where both cell types secreted it. This discrepancy represents a limitation of our study, given that the 48 h PGE_2_-conditioned group was expected to yield results comparable to those obtained in Experiment 1 for the corresponding time point. Finally, TGF β1 was detected only in the 48 h sPGE_2_ secretome and not in the T0MYO, 48 h PGE_2_, or ET eMSC samples, which supported the anti-fibrotic effect of PGE_2_ by inhibiting TGF β1 signaling. Its absence in T0MYO additionally suggested that TGF β1 release occurred in intermittent pulses rather than continuously, consistent with previous observations of its episodic secretion and long-lasting biological impact [66].

Limitations of this work include working with an in vitro two-dimensional cell model which does not necessarily reflects the actual interactions of the in vivo situation, thus the “native” state migh not be accurately represented, this is a common limitation to in vitro models. Other technical and biological limitations are reliance on spectral counts, which are semi-quantitative and less accurate for larger proteins, mainly indicating presence or absence. Additionally, the bottom-up approach used here assigns peptides to protein groups or families; detecting a peptide does not necessarily confirm the presence of the full-length protein, and intact proteoforms with modifications are undetectable; it could be a degradation product or an isoform/proteoform. For example, differentiating pro-MMPs from active forms was not possible in this research. Independent verification of SILAC labeling via LC-MS/MS on the heavy-labeled sample alone was not part of the original workflow. Since raw material cannot be recovered, retrospective assessment of labeling efficacy is not possible. We recognize the absence of direct SILAC verification, which may introduce variability. Future research should utilize top-down proteomics, multi-omics, improved separation methods, and advanced bioinformatics to better characterize proteoforms, aiming for more precise functional and clinically relevant insights.

## 5. Conclusions

Together, these observations supported a model in which PGE_2_-primed ET-eMSCs in co-culture induced myofibroblasts to adopt a secretory profile that coordinated ECM remodeling characterized by reduced stiffness through balanced matrix degradation and deposition, antioxidant protection, cytoskeletal plasticity, and regulated growth factor signaling, thus favoring resolution over the progression of fibrosis.

## Figures and Tables

**Table 1:** Summary of key secreted proteins mediating bidirectional crosstalk between PGE_2_-preconditioned mesenchymal stem cells and myofibroblasts: differential regulation in experiment 1 and SILAC-based cellular origin in experiment 2.

	Secretome Experiment 1 (Abundance)	Secretome Experiment 2 (in Donor Samples)				Origin Cell
Gene Name	Secretome 48 h vs. 0 h	48H PGE_2_	48H SPGE_2_	T0MSC	T0MYO	
MMP1	high abundance	+	+	+	+	both
MMP2	Equal	+	+	+	+	both
MMP9	high abundance	+	+	+	+	both
MMP14	high abundance	+	+	-	+	both
TIMP1	Equal	+	+	+	+	both
TIMP2	Equal	+	+	+	+	both
MXRA5	high abundance	+	+	-	+	both
MXRA8	high abundance	+	+	-	-	ET-eMSC
CXCL6	high abundance	+	+	-	+	MYO
CXCL8	high abundance	+	+	-	-	both
C-C motif chemo kine	Equal	+	+	-	+	MYO
HMGB1	Equal	+	+	-	-	both
THBS 1	Equal	+	+	+	+	both
THBS 2	Equal	+	+	+	+	both
PLAU	high abundance	+	+	+	+	both
SERPINE 1	high abundance	+	+	+	+	both
CCN2/CTGF	high abundance	-	+	-	+	both
TGF β1	Equal	-	+	-	-	both
INHBA	high abundance	+	+	-	+	both
PCOLCE	high abundance	+	+	+	+	both
PCOLCE2	Equal	+	+	+	+	both

Both: Presence of labeled peptides with heavy arginine as lysine, denoting proteins produced by ET-eMSC as myofibroblasts, respectively. ET-eMSC: detection of peptides only labeled with 13C_615N_4 L-Arginine HCl. MYO: detection of peptides only labeled with 13C_6 15N_2 L-Lysine-2HCl. + Represents the detection of the protein m in the sample. - Absence of the detection of the protein in the sample.

## Data Availability

The mass spectrometry proteomics data have been deposited to the ProteomeXchange Consortium via the PRIDE partner repository with the dataset identifier PXD067551.

## Supplementary Materials

The following supporting information can be downloaded at: https://www.mdpi.com/article/10.3390/proteomes13030041/s1, Supplementary Figure S1: Protein–protein interaction regulatory network; Supplementary Figure S2: Gene ontology analysis of the secretome; Supplementary Figure S3: Gene ontology analysis of the proteome of myofibroblasts. Supplementary Figure S4: Distribution of the number of proteins quantified per sample. Table S1: DAPs in Myofibroblasts; Table S2: DAPs in Secretome; Tables S3 and S4: Cluster; Table S5: Cluster GO; Tables S6 and S7: DAP fibrotic; Table S8: Silac complete mapping with intensities.

## Abbreviations

The following abbreviations are used in this manuscript:

48Hpge_2_	48 h with PGE_2_ (contextual)
AAM	Antibiotic-Antimycotic Solution
Akt	Proteins Kinase B (PKB)
Arg	Arginine
ARPC2	Arp2/3 complex 34 kDa subunit
AT-eMSCs	Adipose tissue-derived mesenchymal stem cells
AU-rich elements	Elementos ricos en adenina y uracilo
AUF1	AU-rich element RNA-binding factor 1 (hnRNP D0)
BP	Biological Process
C5	Complement component 5
C6	Complement component 6
cAMP	Cyclic adenosine monophosphate
CC	Cellular Component
CCN1	Cellular Communication Network Factor 1
CCN2	Cellular Communication Network Factor 2
CCN2/CTGF	Cellular communication network factor 2/Connective Tissue Growth Factor
CLU	Clusterin alpha chain
CO_2_	Dióxido de carbono
COL1A1	Collagen type I alpha 1
COL2A1	Collagen type II alpha 1
COL3A1	Collagen type III alpha 1
COL5A1	Collagen type V alpha 1
COL7A1	Collagen type VII alpha 1
COMP	Cartilage oligomeric matrix protein
COX-2/PTGS2	Cyclooxygenase-2/Prostaglandin-endoperoxide synthase 2
CTGF	Connective Tissue Growth Factor
CTSK	Cathepsin K
CXCL6	C-X-C motif chemokine 6
CXCL8	C-X-C motif chemokine 8
DAPs	Differentially Abundant Proteins
DMEM	Dulbecco’s Modified Eagle Medium
DTT	Dithiothreitol
ECM	Extracellular Matrix
EDTA	Ethylenediaminetetraacetic acid
eMSC.0H	ET-eMSC preconditioned with PGE_2_ (contextual)
eMSC.48H	ET-eMSC after 48 h of co-culture with myofibroblasts (contextual)
eMSCs	equine MSCs
EP_2_	Prostaglandin E_2_ receptor subtype 2
ER-to-Golgi	Endoplasmic Reticulum to Golgi
ET-eMSC	Equine endometrium-derived mesenchymal stem cells
ET-eMSCs	Equine mesenchymal stem cells derived from endometrial tissue
ET-eMSCs	equine endometrial-derived MSCs
F11R	Junctional adhesion molecule A
F5	Factor V
FBS	Fetal Bovine Serum
FC	Fold-Change
FDR	False Discovery Rate
FERMT2	FERM domain containing kindlin 2
FOXO1	Forkhead box protein O1
FWHM	Full Width at Half Maximum
GJA1	Gap junction protein
GO	Gene Ontology
GSN	Gelsolin
h	hour(s)
H/L	Heavy/Light (ratio)
HEPES	4-(2-hydroxyethyl)-1-piperazineethanesulfonic acid
hnRNP D0	Heterogeneous Nuclear Ribonucleoprotein D0
HPLC	High-Performance Liquid Chromatography
IGF	Insulin-like Growth Factor
IGFBPs	IGF Binding Proteins
IL-1β	Interleukin-1 beta
IL-6	Interleukin-6
JAK/STAT	Janus Kinase/Signal Transducer and Activator of Transcription
JAK1	Janus Kinase 1
K0/R0	Unlabeled lysine/arginine (SILAC labels)
K8/R10	Lysine-8/Arginine-10 (SILAC labels)
KEGG	Kyoto Encyclopedia of Genes and Genomes
LC-MS/MS	Liquid Chromatography Tandem-Mass Spectrometry
logFC	Logarithm of Fold Change
LOXL2	Lysyl oxidase homolog 2
LUM	Lumican
Lys	Lysine
m/z	Masa por carga (mass-to-charge ratio)
MAPK	Mitogen-Activated Protein Kinase
MCL	Markov Clustering
MF	Molecular Function
MMP-1	Matrix metalloproteinase-1
MMP-14	Matrix metalloproteinase-14
MMP-2	Matrix metalloproteinase-2
MMP-9	Matrix metalloproteinase-9
MMP/TIMP	Matrix metalloproteinase/Tissue Inhibitor of metalloproteinases
MMPs	Matrix metalloproteinase
mRNA	messenger RNA
MS	Mass Spectrometry
MSCs	Mesenchymal Stem Cells
MXR5	Matrilysin 5
MXR8	Matrilysin 8
MXRA5	Matrix Remodeling Associated 5
MXRA8	Matrix Remodeling Associated 8
MYO-ET-eMSC	Myofibroblasts co-cultured with ET-eMSCs (contextual)
Myo.0H	Myofibroblast samples at 0 h (contextual)
Myo.48H	Myofibroblast samples at 48 h (contextual)
NaCl	Cloruro de sodio
PAI-1/SERPINE1	Plasminogen Activator Inhibitor-1/Serpin Family E Member 1
PBS	Phosphate-Buffered Saline
PCA	Principal Component Analysis
PCR	Polymerase Chain Reaction
PDGF	Platelet-Derived Growth Factor
PDGFRα	Platelet-derived growth factor receptor alpha
PGE_2_	Prostaglandin E_2_
PI3K/AKT	Phosphoinositide 3-kinase/Protein Kinase B
PKA	ProteinKinase A
PLAT	Plasminogen activator, urokinase-type
PLAU	Urokinase-type plasminogen activator
PPI	Protein-ProteinInteraction
PPM	Parts per million
PTGIS	Prostacyclin synthase
PTGS2	Prostaglandin G/H synthase 2
PTM	Post-Translational Modifications
Rho/ROCK	Rho-associated protein kinase
SGCA	Sarcoglycan alpha
SILAC	Stable isotope labeling by amino acids in cell culture
SPD	Samples per Day
sPGE_2_	Sin PGE_2_ (referring to conditioned medium without PGE_2_) (contextual)
STAT3	Signal transducer and activator of transcription 3
T0EMSC	ET-eMSCs at time zero (contextual)
T0MYO	Myofibroblasts at time zero (contextual)
TGF-β1	Transforming Growth Factor-beta 1
THBS1	Thrombospondin-1
THBS2	Thrombospondin 2
TIMP-1	Tissue Inhibitor of Metalloproteinase-1
TIMP-2	Tissue Inhibitor of Metalloproteinase-2
TMM	Trimmed Mean of M-values
TNF-α	Tumor Necrosis Factor-alpha
TriC/CCT	TCP-1 Ring Complex/Chaperonin Containing TCP-1
uPA	Urokinase-type plasminogen activator
YAP/TAZ	Yes-associated protein/Transcriptional coactivator with PDZ-binding motif
α-SMA	α-smooth muscle actin
µL	microliters
μM	micromolar
