# Supplementary material for: Bidirectional Interaction Between PGE2-Preconditioned Mesenchymal Stem Cells and Myofibroblasts Mediates Anti-Fibrotic Effects: A Proteomic Investigation into Equine Endometrial Fibrosis Reversal
Source: Proteomes. 2025 Sep 8;13(3):41. doi: 10.3390/proteomes13030041 (PMC12452512; doi:10.3390/proteomes13030041)
Supplement: Supplementary file 1 [file proteomes-13-00041-s001.zip › proteomes-3748745-supplementary-8.22/Figure Supplementary 1.docx]

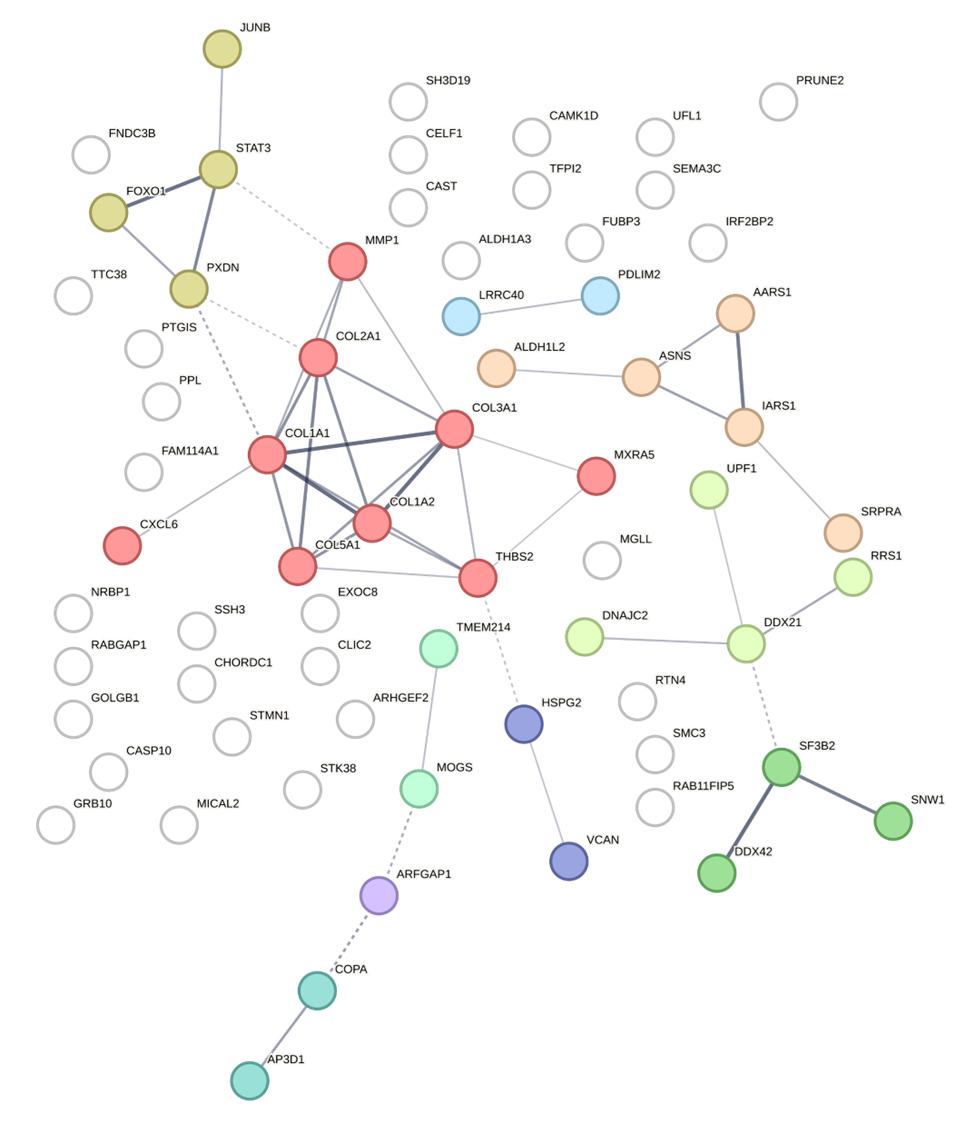


**Figure Supplementary 1: Proteome (myofibroblasts) low-abundance proteins**

**a)**

**Figure Supplementary 1: Proteome (myofibroblasts) high-abundance proteins**

**b)**


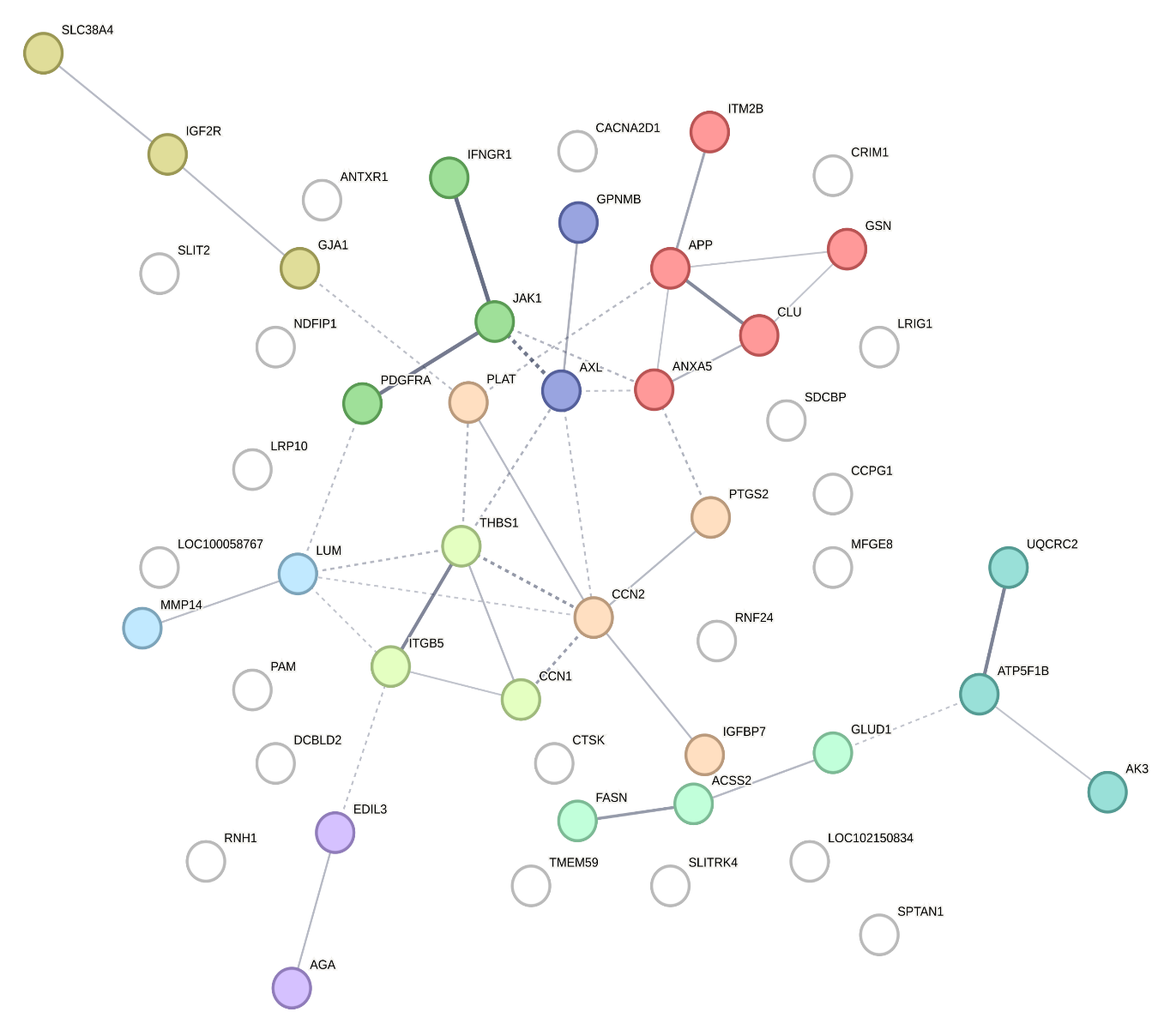


**Figure Supplementary 1: Secretome (culture supernatant): low-abundance proteins.**


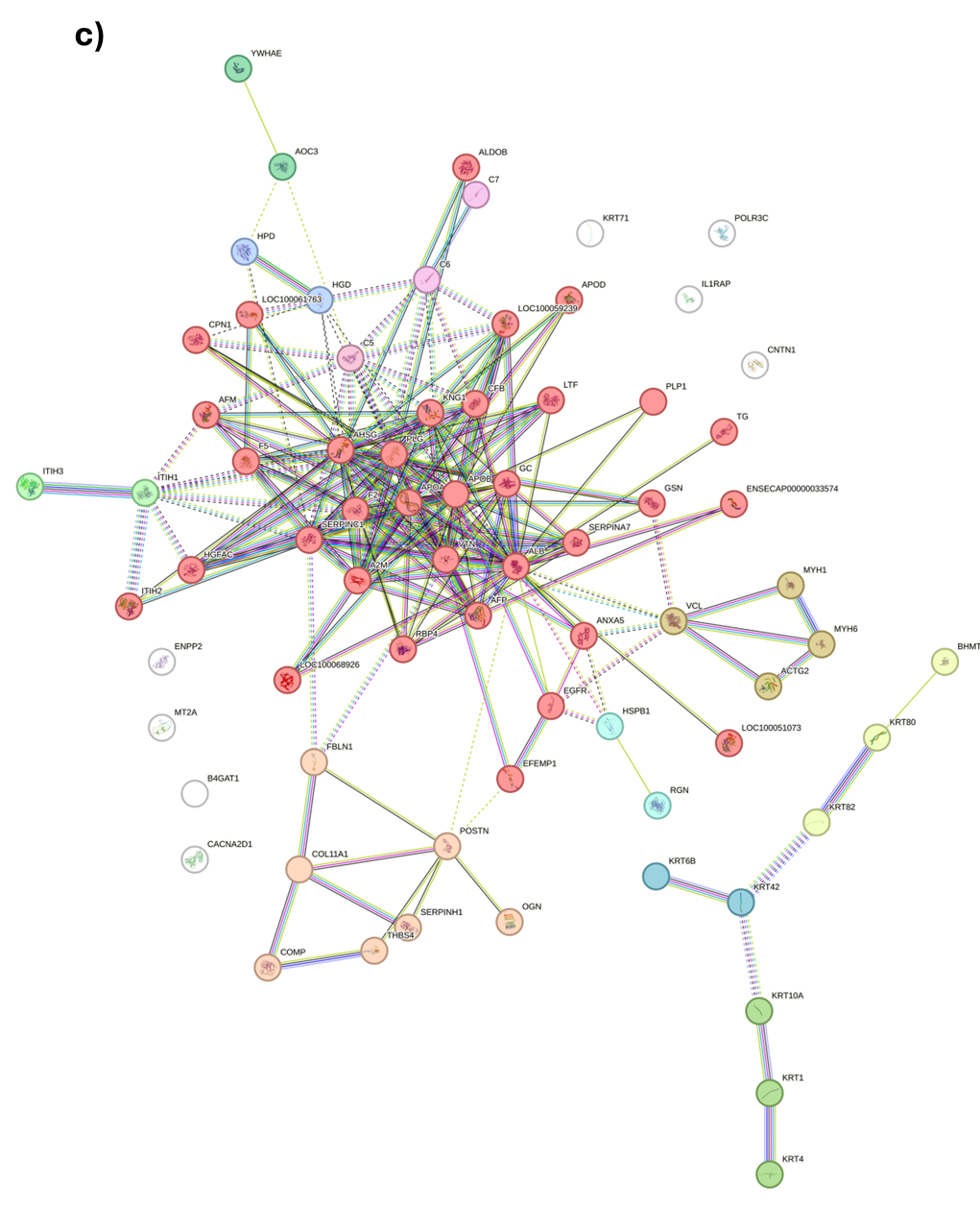


**Figure Supplementary 1: Secretome (culture supernatant): high-abundance proteins**


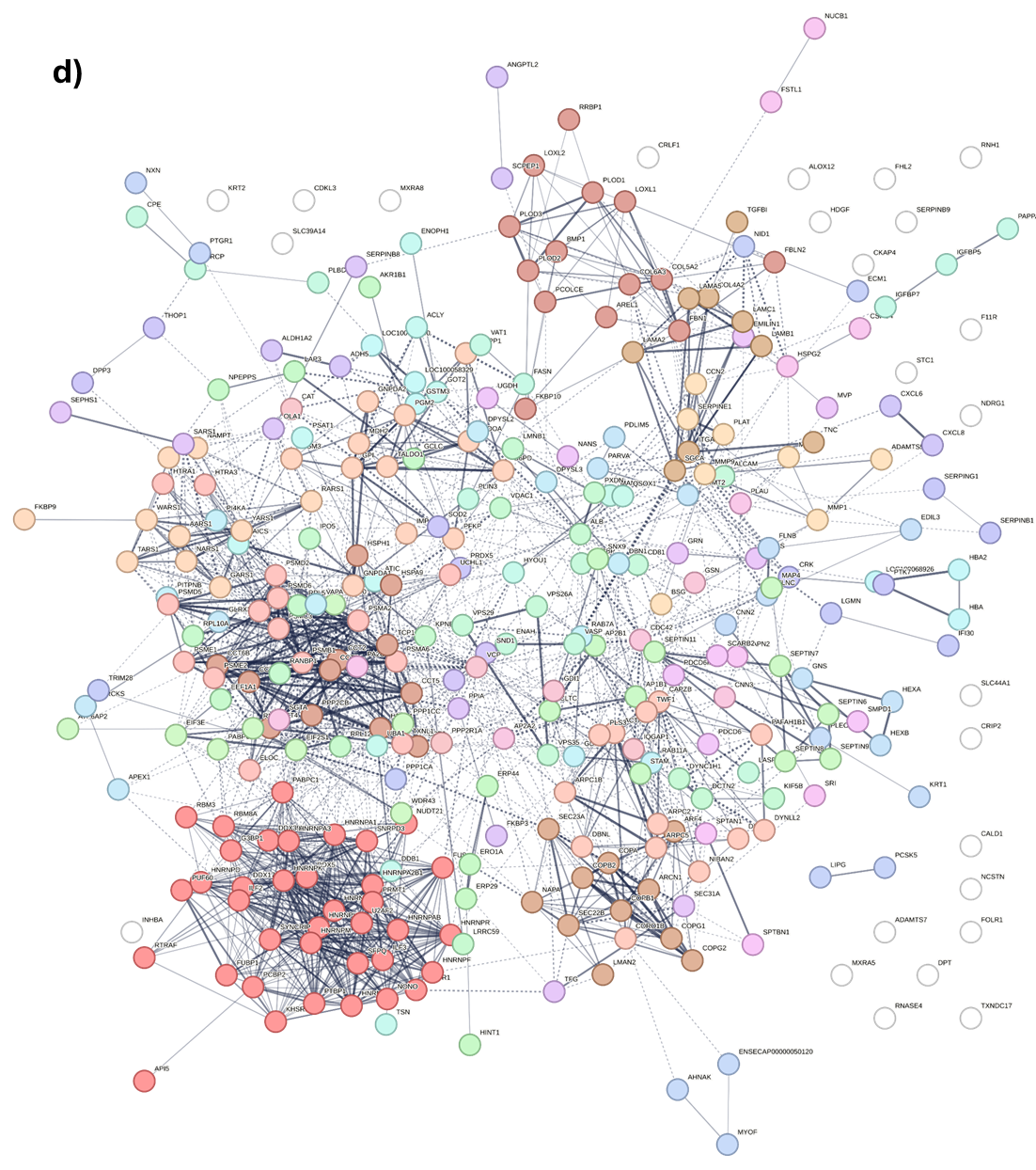


**Figure Supplementary 1.** Protein-protein interaction regulatory network. Differentially abundant protein (DAPs) in myofibroblasts and the secretome of ET-eMSC were used to construct PPI networks with STRING software. Active interaction sources included Text Mining, Experiments, Databases, Abundance, Neighborhood, Gene Fusion, and Co-occurrence, with a minimum interaction confidence score of 0.4 (medium confidence). Panels (a) and (c) represent low-abundance proteins, while panels (b) and (d) represent high-abundance proteins in the proteome and secretome, respectively. The clusters are identified by color; circles of the same color are grouped in the same cluster (Table S3 and S4).
