# Supplementary material for: Bidirectional Interaction Between PGE2-Preconditioned Mesenchymal Stem Cells and Myofibroblasts Mediates Anti-Fibrotic Effects: A Proteomic Investigation into Equine Endometrial Fibrosis Reversal
Source: Proteomes. 2025 Sep 8;13(3):41. doi: 10.3390/proteomes13030041 (PMC12452512; doi:10.3390/proteomes13030041)
Supplement: Supplementary file 1 [file proteomes-13-00041-s001.zip › proteomes-3748745-supplementary-8.22/Figure Supplementary 3.docx]

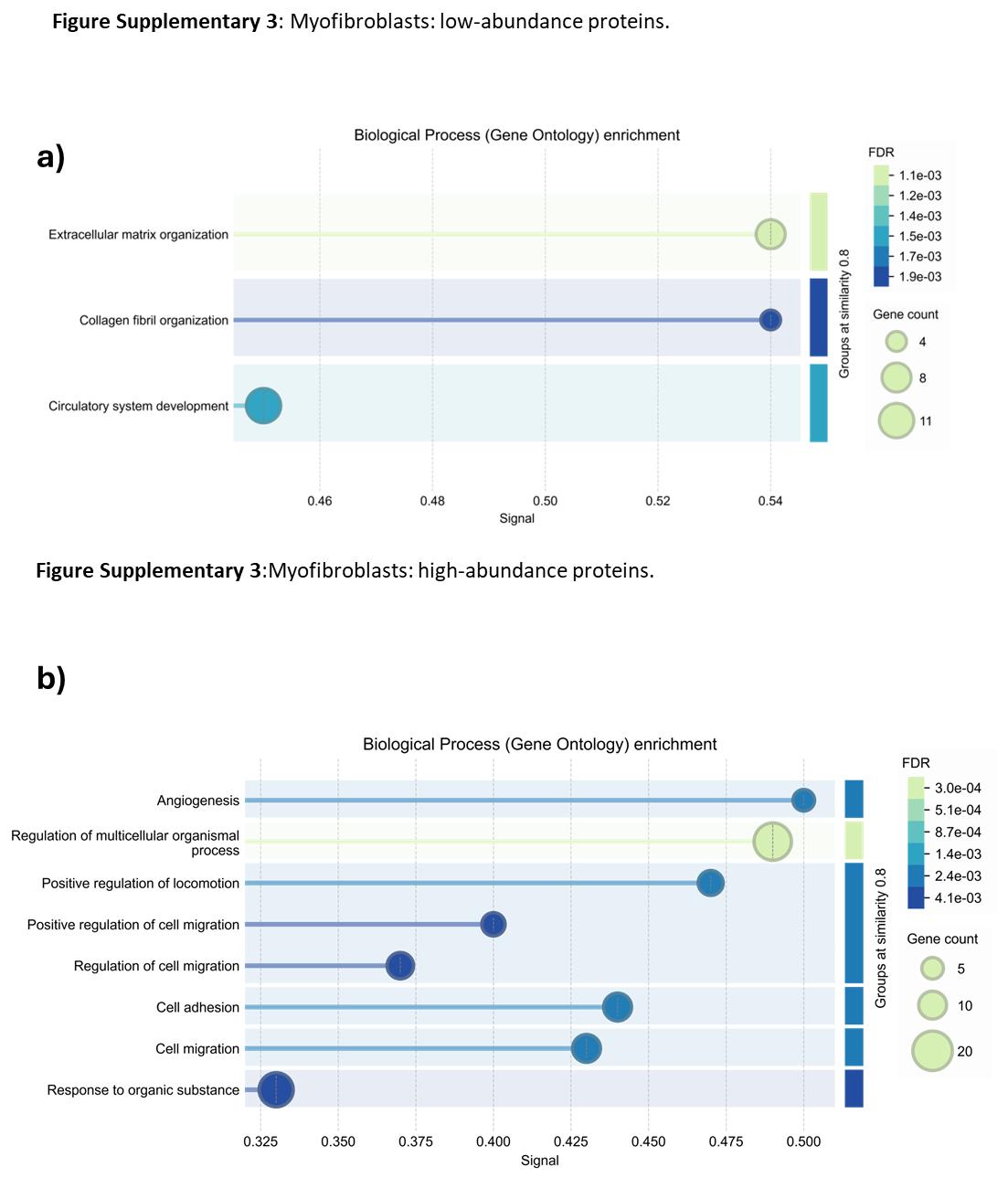


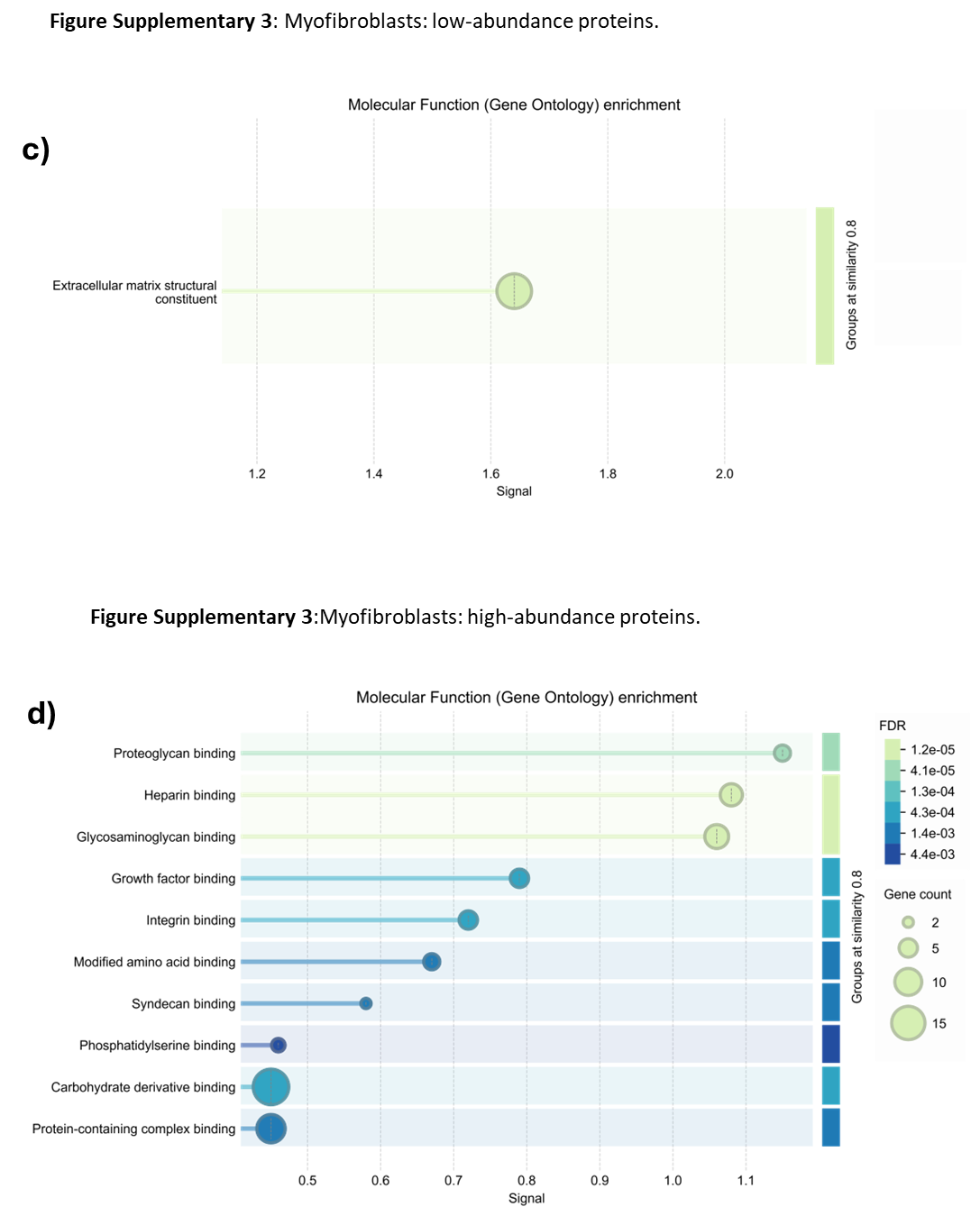


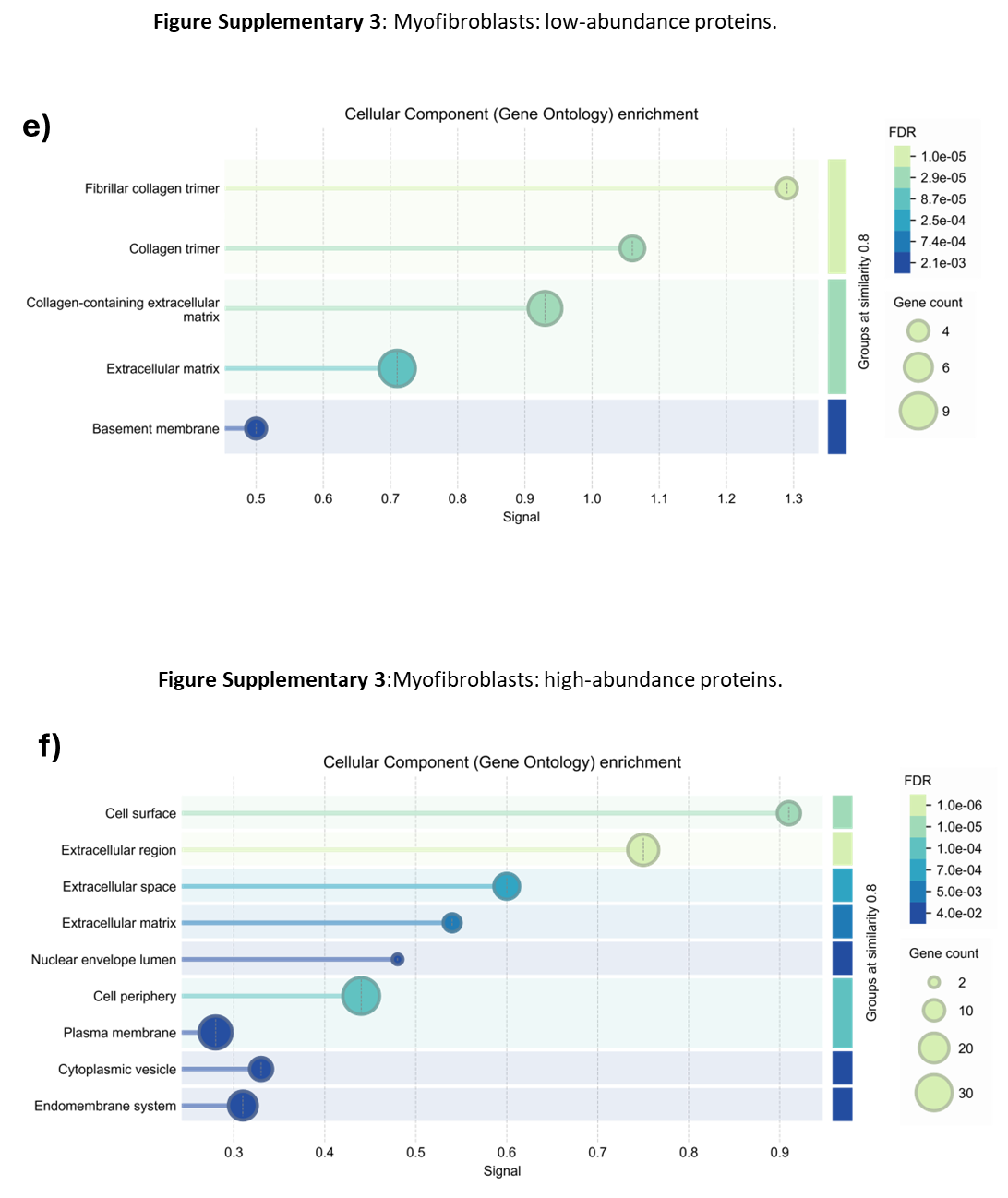


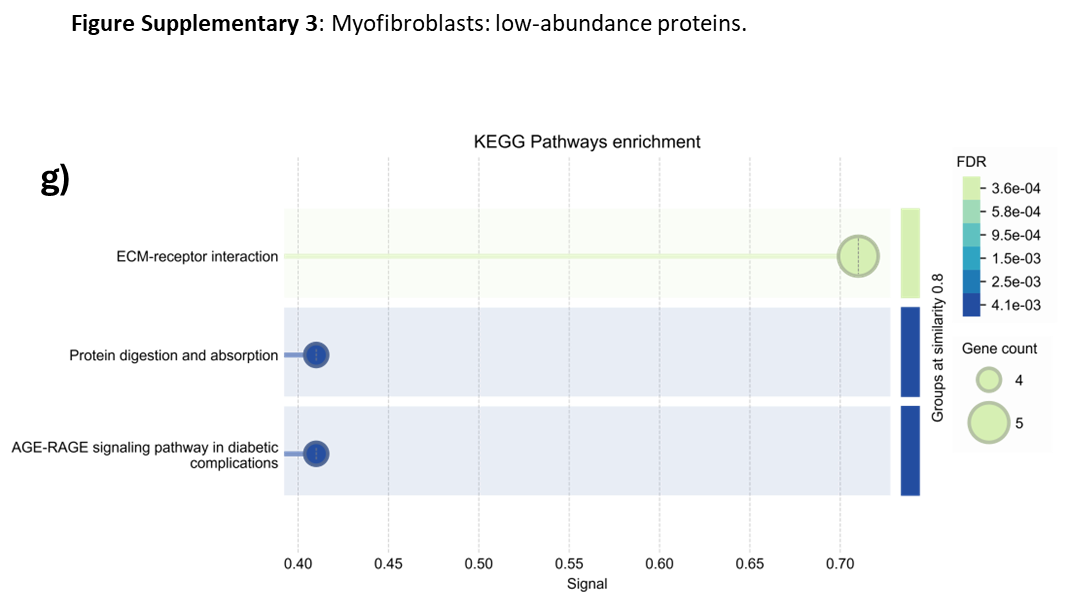


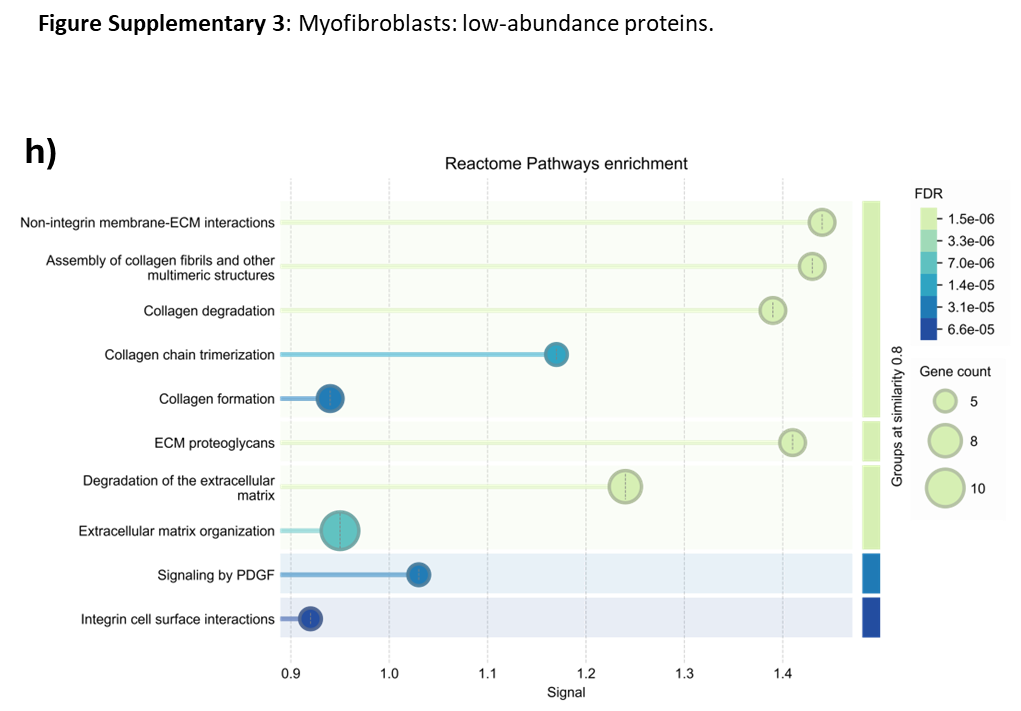


**Figure Supplementary 3:** Gene ontology analysis of 136 DAPs between myofibroblast (0 hour) and myofibroblast co-culture with ET-eMSCs (48 hours). The top ten biological process categories, cellular component categories, and molecular function are presented. The plots on the left represent the analysis of downregulated genes, while those on the right correspond to upregulated genes. The size of each bubble indicates the number of genes enriched in each category, and the color gradient represents the false discovery rate (FDR) values. The analysis was performed using the STRING v12.0 analysis tool, with an FDR threshold of 0,05 and a minimal signal of 0,01. For Proteome (Myofibroblasts) Up-regulated proteins 48hours VS 0 hours were no significant pathway enrichments observed in the following categories: Reactome Pathways, KEGG Pathways.
