# Supplementary material for: Bidirectional Interaction Between PGE2-Preconditioned Mesenchymal Stem Cells and Myofibroblasts Mediates Anti-Fibrotic Effects: A Proteomic Investigation into Equine Endometrial Fibrosis Reversal
Source: Proteomes. 2025 Sep 8;13(3):41. doi: 10.3390/proteomes13030041 (PMC12452512; doi:10.3390/proteomes13030041)
Supplement: Supplementary file 1 [file proteomes-13-00041-s001.zip › proteomes-3748745-supplementary-8.22/Figure Supplementary 4 _quality control for SILAC.docx]

**Figure S4. Quality control charts and statistics for SILAC data.**

**
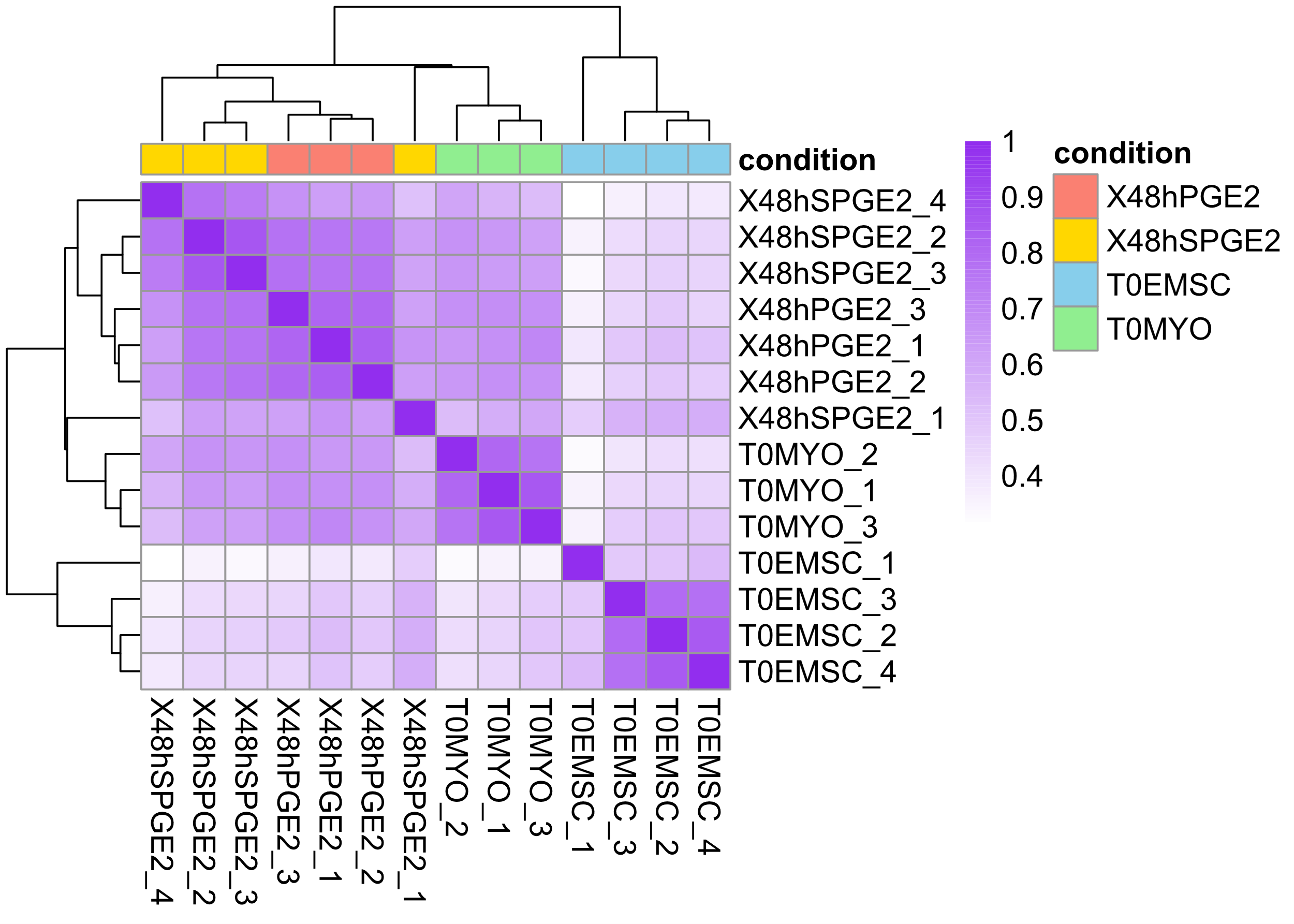
Pearson correlation Heavy**

**Pearson correlation Light**

**
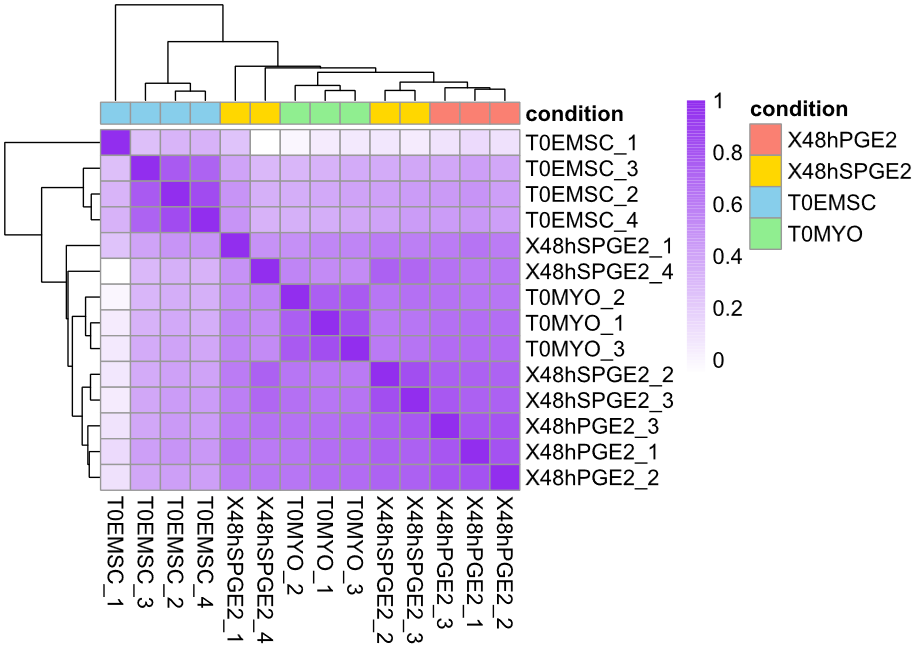
**

**Distribution of the number of quantified proteins per sample, broken down by isotopic channel (left) and by type of calculated ratio (right). The highest coverage was observed in the Light and Heavy channels, consistent with the type of cell labeling and the efficiency of isotope incorporation.**

**
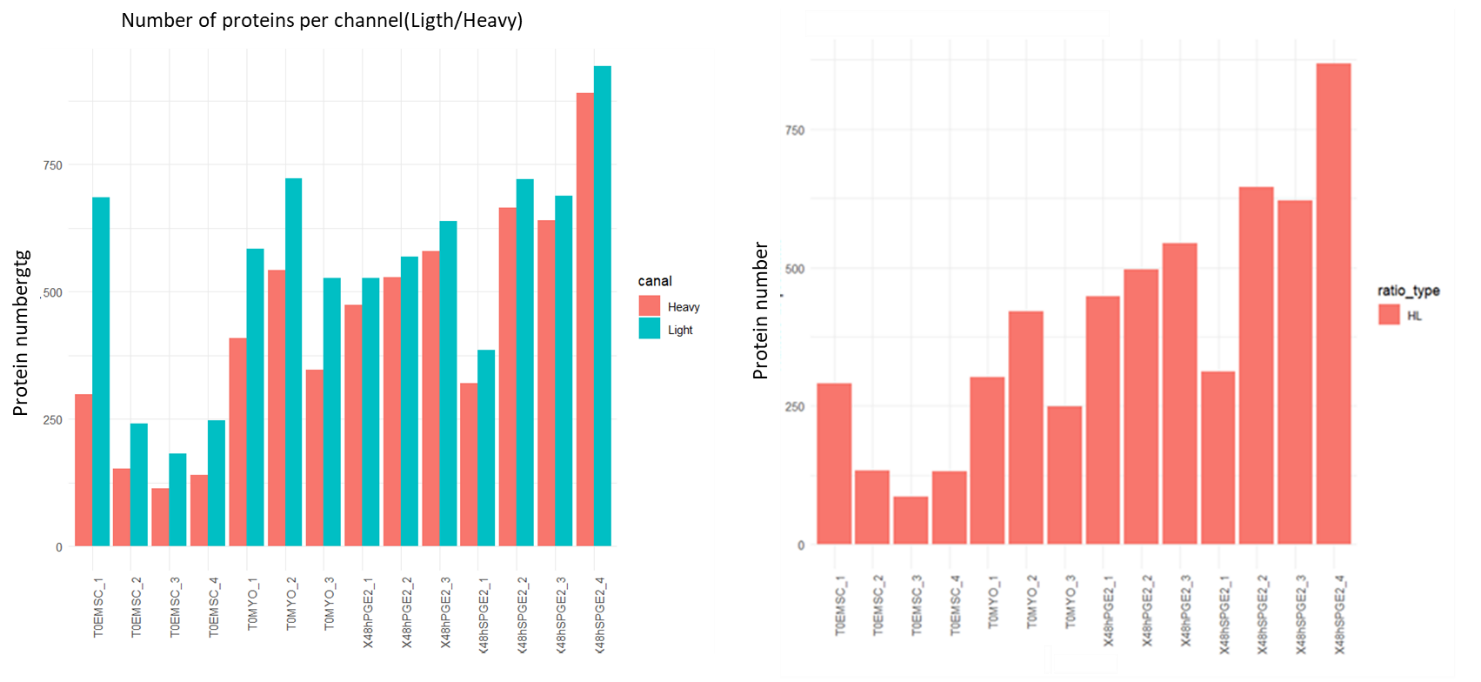
**

**Log2 intensity distribution per sample, before and after quantile normalization. A substantial improvement in the homogeneity of the post-normalization distributions is observed, supporting the data quality for subsequent statistical analysis.**

**
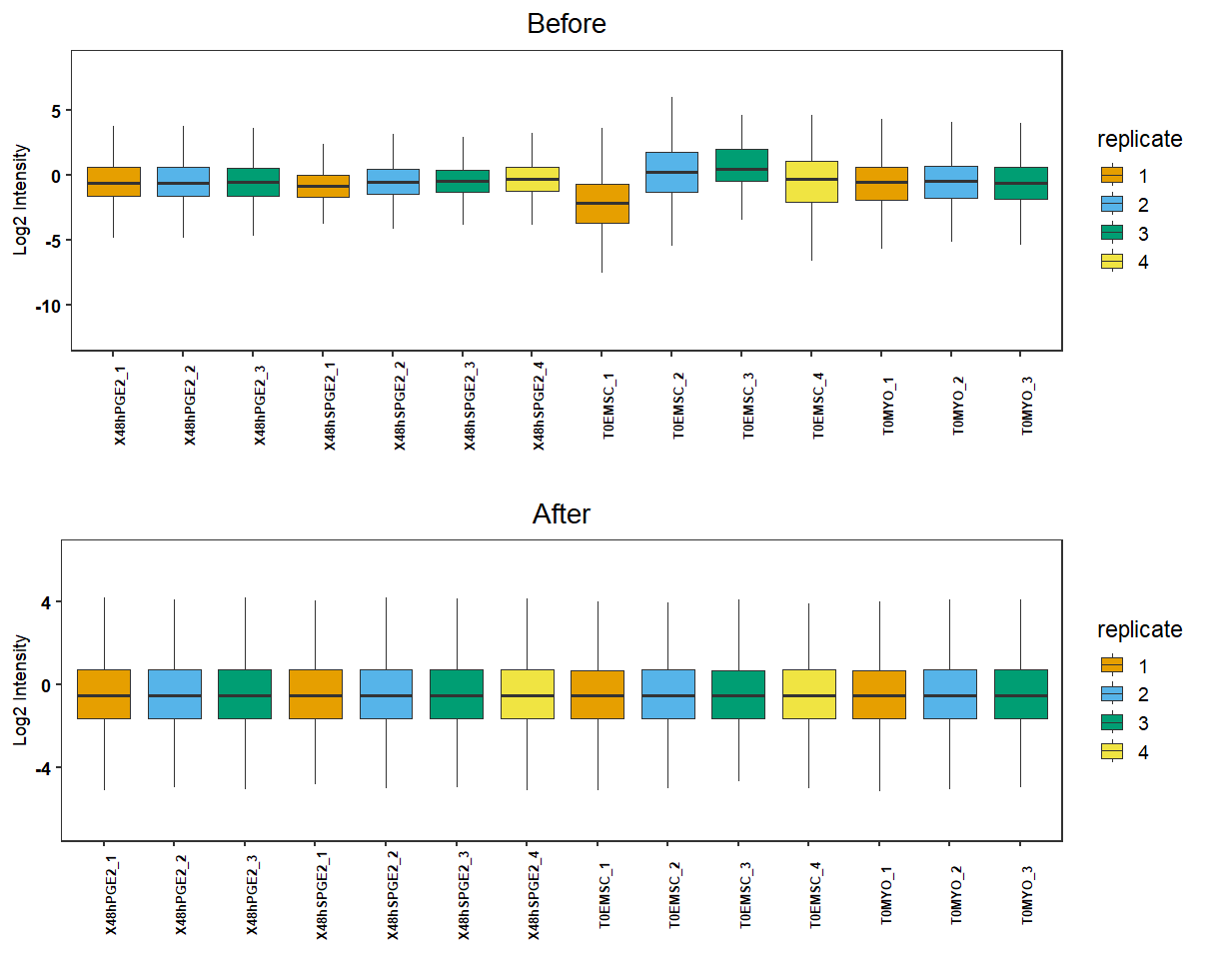
**
