# Supplementary material for: Bidirectional Interaction Between PGE2-Preconditioned Mesenchymal Stem Cells and Myofibroblasts Mediates Anti-Fibrotic Effects: A Proteomic Investigation into Equine Endometrial Fibrosis Reversal
Source: Proteomes. 2025 Sep 8;13(3):41. doi: 10.3390/proteomes13030041 (PMC12452512; doi:10.3390/proteomes13030041)
Supplement: Supplementary file 1 [file proteomes-13-00041-s001.zip › proteomes-3748745-supplementary-8.22/Table supplementary 1 DAPs in Myofibroblasts.docx]

| **DAPs in Myofiblasts** | | | | | | | |
| --- | --- | --- | --- | --- | --- | --- | --- |
| **Protein.ID** | **GenName** | **Description** | **logFC** | **logCPM** | **PValue** | **FDR** | **DEGs** |
| A0A5F5PZ45 | GBP1 | Guanylate binding protein 1 | -5,8092717 | 6,12280009 | 1,41E-06 | 0,000259852 | Down |
| A0A3Q2HJY6 | RABGAP1 | RAB GTPase activating protein 1 | -4,9551447 | 5,62092791 | 0,00149652 | 0,043773102 | Down |
| A0A3Q2HZS8 | GOLGB1 | Golgin B1 | -4,7653958 | 7,47576079 | 8,78E-12 | 8,47E-09 | Down |
| A0A9L0SSV6 | DDX42 | DEAD-box helicase 42 | -4,1032499 | 6,32074221 | 8,12E-07 | 0,000164965 | Down |
| Q8MIN2 | CXCL6 | C-X-C motif chemokine ligand 6 | -3,8662149 | 6,16491171 | 1,10E-05 | 0,001267333 | Down |
| A0A5F5PH27 | TMEM214 | Transmembrane protein 214 | -3,7312804 | 6,08016768 | 4,03E-05 | 0,003662359 | Down |
| A0A3Q2GUK5 | FNDC3B | Fibronectin type III domain containing 3B | -3,723051 | 6,07987255 | 0,00013813 | 0,008152751 | Down |
| A0A3Q2HH66 | MGLL | Monoglyceride lipase | -3,6595069 | 6,03584702 | 8,54E-05 | 0,005889031 | Down |
| F6PJV0 | MXRA5 | Matrix remodeling associated 5 | -3,5003674 | 6,89224768 | 3,32E-07 | 8,53E-05 | Down |
| A0A3Q2HJ99 | DNAJC2 | DnaJ heat shock protein family (Hsp40) member C2 | -3,3040146 | 6,35709957 | 5,66E-06 | 0,00084048 | Down |
| F6YSE4 | EXOC8 | Exocyst complex component 8 | -3,1301356 | 6,24503923 | 4,13E-05 | 0,003662359 | Down |
| F6PSS0 | RRS1 | Ribosome biogenesis regulator 1 homolog | -3,1294103 | 6,24486617 | 4,17E-05 | 0,003662359 | Down |
| Q9XSZ5 | MMP1 | Matrix metallopeptidase 1 | -3,10653 | 7,14295489 | 3,91E-10 | 2,52E-07 | Down |
| A0A9L0RT02 | CAMK1D | Calcium/calmodulin dependent protein kinase ID | -3,0669289 | 6,2051554 | 7,91E-05 | 0,005760786 | Down |
| F6V7B1 | TFPI2 | Tissue factor pathway inhibitor 2 | -3,0650434 | 6,86782775 | 1,41E-07 | 4,18E-05 | Down |
| A0A3Q2I8Z8 | GRB10 | Growth factor receptor bound protein 10 | -3,0010976 | 6,16493011 | 0,00012154 | 0,007448915 | Down |
| A0A9L0SRE2 | STK38 | Serine/threonine kinase 38 | -2,9320781 | 6,12287078 | 0,00022876 | 0,011803341 | Down |
| F6Y5A4 | JUNB | JunB proto-oncogene, AP-1 transcription factor subunit | -2,8978658 | 6,46144247 | 2,11E-05 | 0,00226549 | Down |
| A0A9L0TQS8 | RIGI | RNA sensor RIG-I | -2,8594206 | 6,08007214 | 0,00046171 | 0,019376556 | Down |
| A0A3Q2ICG0 | ARFGAP1 | ARF GTPase activating protein 1 | -2,8451015 | 6,42763234 | 0,00014033 | 0,008152751 | Down |
| F6UF33 | CLIC2 | Chloride intracellular channel 2 | -2,79915 | 6,67851166 | 3,12E-06 | 0,000502557 | Down |
| F6VYT3 | FOXO1 | Forkhead box O1 | -2,7850339 | 6,03596255 | 0,00098671 | 0,032842111 | Down |
| A0A3Q2KXT4 | RAB11FIP5 | RAB11 family interacting protein 5 | -2,7831675 | 6,03595433 | 0,00073204 | 0,026918225 | Down |
| F6YMG4 | PTGIS | Prostaglandin I2 synthase | -2,4897756 | 6,20548635 | 0,00048953 | 0,020107058 | Down |
| A0A9L0QZX0 | UFL1 | UFM1 specific ligase 1 | -2,4159338 | 6,42811828 | 0,00010385 | 0,006637118 | Down |
| A0A9L0RF01 | AP3D1 | Adaptor related protein complex 3 subunit delta 1 | -2,3736245 | 7,14281293 | 9,86E-05 | 0,006561165 | Down |
| A0A9L0RQ27 | DDX21 | DExD-box helicase 21 | -2,2391098 | 6,89254217 | 0,00033279 | 0,015296219 | Down |
| A0A9L0SJ42 | STAT3 | Signal transducer and activator of transcription 3 | -2,2226136 | 6,52728648 | 0,00063878 | 0,024387853 | Down |
| F6VQ75 | SSH3 | Slingshot protein phosphatase 3 | -2,1797887 | 6,28361745 | 0,00101691 | 0,033557863 | Down |
| A0A9L0TNK8 | IRF2BP2 | Interferon regulatory factor 2 binding protein 2 | -2,1708771 | 6,49500709 | 0,00061527 | 0,023781558 | Down |
| A0A9L0RGY6 | PDLIM2 | PDZ and LIM domain 2 | -2,1691268 | 6,98939154 | 0,00014148 | 0,008152751 | Down |
| A0A3Q2I4K8 | NRBP1 | Nuclear receptor binding protein 1 | -2,1134338 | 6,24496115 | 0,00159114 | 0,04550675 | Down |
| A0A9L0RCX2 | SEMA3C | Semaphorin 3C | -2,0831389 | 6,94109893 | 0,00042372 | 0,017999966 | Down |
| A0A9L0RTV8 | CASP10 | Caspase 10 | -2,0617668 | 6,42776108 | 0,0005467 | 0,022136035 | Down |
| A0A5F5PIB7 | COL2A1 | Collagen type II alpha 1 chain | -2,0159074 | 6,89320547 | 6,58E-05 | 0,005107894 | Down |
| A0A5F5PRZ2 | ARHGEF2 | Rho/Rac guanine nucleotide exchange factor 2 | -2,0099383 | 7,14324876 | 8,72E-06 | 0,001122558 | Down |
| F7CGE1 | LRRC40 | Leucine rich repeat containing 40 | -1,9755374 | 6,55862243 | 0,00058461 | 0,023032245 | Down |
| A0A9L0S4V0 | A0A9L0S4V0 | High mobility group nucleosome binding domain 1 | -1,9477469 | 7,10099301 | 2,25E-05 | 0,002350509 | Down |
| A0A3Q2GSP0 | CELF1 | CUGBP Elav-like family member 1 | -1,9434323 | 6,3575817 | 0,00151552 | 0,043995634 | Down |
| A0A3Q2L9H8 | STAT3 | Signal transducer and activator of transcription 3 | -1,9239306 | 6,52722358 | 0,00113076 | 0,035494717 | Down |
| F6ULE3 | MOGS | Mannosyl-oligosaccharide glucosidase | -1,9239262 | 6,52806991 | 0,00131971 | 0,040081112 | Down |
| A0A3Q2HE76 | SNW1 | SNW domain containing 1 | -1,8982119 | 6,81625415 | 0,00031661 | 0,01472793 | Down |
| A0A3Q2I3I2 | MICAL2 | Microtubule associated monooxygenase, calponin and LIM domain containing 2 | -1,6940339 | 6,8168412 | 0,00094138 | 0,031966263 | Down |
| A0A3Q2IDL1 | ALDH1A3 | Aldehyde dehydrogenase 1 family member A3 | -1,5240389 | 7,87850464 | 7,69E-06 | 0,001024043 | Down |
| A0A3Q2HBK0 | SH3D19 | SH3 domain containing 19 | -1,4963652 | 7,01240773 | 0,00090105 | 0,031067812 | Down |
| A0A3Q2HBC4 | SMC3 | Structural maintenance of chromosomes 3 | -1,4916589 | 7,42794593 | 0,00138394 | 0,041102973 | Down |
| A0A9L0R623 | TTC38 | Tetratricopeptide repeat domain 38 | -1,4481494 | 7,0801594 | 0,0012948 | 0,039676499 | Down |
| A0A9L0S7Y5 | FAM114A1 | Family with sequence similarity 114 member A1 | -1,4413818 | 7,2445188 | 0,00039488 | 0,017728205 | Down |
| A0A5F5PZQ6 | SRPRA | SRP receptor subunit alpha | -1,3825822 | 7,42723879 | 0,00016652 | 0,009184613 | Down |
| A0A9L0SHD1 | PPL | Periplakin | -1,3663458 | 7,87862 | 0,00047172 | 0,019583982 | Down |
| A0A9L0SXF6 | FAM114A1 | Family with sequence similarity 114 member A1 | -1,3007326 | 7,73463006 | 0,00042424 | 0,017999966 | Down |
| F7BEY7 | FUBP3 | Far upstream element binding protein 3 | -1,2285457 | 7,78960458 | 0,00040751 | 0,017999966 | Down |
| A0A9L0TCZ6 | CHORDC1 | Cysteine and histidine rich domain containing 1 | -1,2162181 | 7,51055954 | 0,00076645 | 0,027656496 | Down |
| A0A5F5PRZ7 | COL5A1 | Collagen type V alpha 1 chain | -1,2017347 | 8,57093761 | 2,81E-07 | 7,74E-05 | Down |
| A0A5F5Q292 | PRUNE2 | Prune homolog 2 with BCH domain | -1,1948586 | 7,66349693 | 0,00073973 | 0,026944289 | Down |
| A0A3Q2H6U5 | ALDH1A3 | Aldehyde dehydrogenase | -1,1387681 | 7,95226952 | 0,00034925 | 0,015864283 | Down |
| A0A3Q2GZL7 | ASNS | Asparagine synthetase [glutamine-hydrolyzing] | -1,1058935 | 8,16306938 | 3,18E-05 | 0,003071404 | Down |
| A0A9L0RA24 | STMN1 | Stathmin 1 | -1,0719223 | 8,2446812 | 0,00021585 | 0,011441637 | Down |
| A0A9L0R6B9 | UPF1 | UPF1 RNA helicase and ATPase | -1,0528229 | 7,94108657 | 0,00159115 | 0,04550675 | Down |
| A0A9L0TK32 | COL1A2 | Collagen type I alpha 2 chain | -1,0442891 | 9,55197572 | 1,95E-10 | 1,51E-07 | Down |
| A0A9L0R7P4 | COL5A1 | Collagen type V alpha 1 chain | -1,0419968 | 8,04424808 | 0,00161563 | 0,045867191 | Down |
| A0A9L0R910 | THBS2 | Thrombospondin 2 | -0,986817 | 7,97733647 | 0,00103463 | 0,033853513 | Down |
| A0A9L0T4M1 | A0A9L0T4M1 | Collagen type VII alpha 1 chain | -0,9798343 | 9,42104241 | 2,95E-06 | 0,00049505 | Down |
| A0A3Q2HNE1 | PXDN | Peroxidasin | -0,9658773 | 9,25588075 | 5,33E-08 | 2,24E-05 | Down |
| A0A5F5PJP7 | IARS1 | Isoleucine--tRNA ligase | -0,8898913 | 8,91415477 | 7,66E-05 | 0,005717946 | Down |
| A0A9L0RHE1 | COL3A1 | Collagen type III alpha 1 chain | -0,8311792 | 9,54943889 | 4,20E-07 | 0,00010145 | Down |
| A0A3Q2HT74 | HSPG2 | Heparan sulfate proteoglycan 2 | -0,8298922 | 9,02238084 | 2,40E-05 | 0,002441714 | Down |
| A0A9L0SU25 | ALDH1L2 | 10-formyltetrahydrofolate dehydrogenase | -0,8017578 | 8,80080542 | 0,00011848 | 0,007377925 | Down |
| A0A5F5PFU1 | SF3B2 | Splicing factor 3b subunit 2 | -0,7586946 | 8,49444252 | 0,00120805 | 0,037615041 | Down |
| A0A3Q2IF55 | CAST | Calpastatin | -0,7533014 | 8,8861527 | 0,00024927 | 0,012498854 | Down |
| A0A9L0RE13 | SF3B2 | Splicing factor 3b subunit 2 | -0,7477633 | 8,53473994 | 0,00109179 | 0,034837916 | Down |
| A0A3Q2LD09 | CAST | Calpastatin | -0,7447398 | 9,08327092 | 8,47E-05 | 0,005889031 | Down |
| A0A5F5PJ23 | IARS1 | Isoleucine--tRNA ligase | -0,7308564 | 8,8332338 | 0,00061594 | 0,023781558 | Down |
| F7DY34 | VCAN | Versican | -0,7115924 | 9,19781381 | 0,00010101 | 0,006609907 | Down |
| A0A9L0RB71 | COL1A1 | Collagen type I alpha 1 chain | -0,6950866 | 10,636761 | 6,17E-08 | 2,24E-05 | Down |
| A0A5F5PVK6 | VCAN | Versican core protein | -0,6667117 | 9,40760707 | 8,50E-05 | 0,005889031 | Down |
| A0A3Q2HV04 | RTN4 | Isoform B2 of Reticulon-4 | -0,5787575 | 9,56862932 | 0,00023052 | 0,011803341 | Down |
| A0A3Q2HWW0 | COPA | Coatomer subunit alpha | -0,5369969 | 9,64494192 | 0,00055885 | 0,022244559 | Down |
| A0A3Q2I6Z2 | AARS1 | Alanine--tRNA ligase | -0,4726079 | 9,87250003 | 0,00090122 | 0,031067812 | Down |
| O19183 | PTGS2 | Prostaglandin-endoperoxide synthase 2 | 5,99828874 | 6,12583165 | 5,15E-07 | 0,000116931 | Up |
| A0A3Q2GYF3 | CCPG1 | Cell cycle progression 1 | 5,61357732 | 5,89515268 | 4,59E-05 | 0,003936433 | Up |
| A0A9L0SUE0 | SLC5A3 | Solute carrier family 5 member 3 | 5,25030916 | 5,68141992 | 0,00013554 | 0,008152751 | Up |
| A0A3Q2H1C4 | JAK1 | Janus kinase 1 | 4,04772468 | 6,08190382 | 1,10E-05 | 0,001267333 | Up |
| A0A9L0R2X6 | DCBLD2 | Discoidin, CUB and LCCL domain containing 2 | 4,02847566 | 6,6513719 | 1,23E-06 | 0,000236726 | Up |
| A0A3Q2KWV4 | SLITRK4 | SLIT and NTRK like family member 4 | 3,97775789 | 6,03791817 | 3,26E-05 | 0,003071404 | Up |
| A0A3Q2HUT5 | CRIM1 | Cysteine rich transmembrane BMP regulator 1 | 3,89313012 | 6,52996347 | 6,95E-08 | 2,24E-05 | Up |
| A0A9L0T6Q8 | LOC102150834 | Tumor necrosis factor receptor superfamily  member 1A isoform X3 | 3,81762529 | 5,9441777 | 7,70E-05 | 0,005717946 | Up |
| A0A3Q2H4R1 | LRIG1 | Leucine rich repeats and immunoglobulin like domains 1 | 3,79916763 | 5,94370903 | 0,00041761 | 0,017999966 | Up |
| A0A5F5PZ67 | PCDHGC5 | Protocadherin gamma subfamily C, 5 | 3,11482749 | 6,03718382 | 0,00041137 | 0,017999966 | Up |
| A0A9L0S247 | ACSS2 | Acyl-CoA synthetase short chain family member 2 | 2,87784855 | 6,24596873 | 0,00065744 | 0,024644427 | Up |
| A0A9L0SG62 | RNF24 | RING finger protein 24 | 2,36547606 | 6,39544027 | 0,00021633 | 0,011441637 | Up |
| A0A9L0RI43 | SLC38A4 | Solute carrier family 38 member 4 | 2,32630145 | 6,16701132 | 0,00064428 | 0,024387853 | Up |
| A0A9L0R720 | IFNGR1 | Interferon gamma receptor 1 | 2,21382514 | 6,92044652 | 3,31E-06 | 0,000511368 | Up |
| F6VEV4 | AXL | AXL receptor tyrosine kinase | 2,1649096 | 6,08155887 | 0,00149526 | 0,043773102 | Up |
| A0A3Q2HGZ3 | TMEM59 | Transmembrane protein 59 | 2,12953964 | 7,53149652 | 7,54E-07 | 0,000161801 | Up |
| A0A5F5PFI0 | PAM | Peptidylglycine alpha-amidating monooxygenase | 1,96162249 | 6,49649037 | 0,00055039 | 0,022136035 | Up |
| A0A9L0S909 | ITM2B | Integral membrane protein 2B | 1,7526177 | 7,73959756 | 6,69E-08 | 2,24E-05 | Up |
| A0A9L0RLH1 | NDFIP1 | Nedd4 family interacting protein 1 | 1,71493267 | 7,26708885 | 1,12E-05 | 0,001267333 | Up |
| F6UGF3 | LRP10 | LDL receptor related protein 10 | 1,69777648 | 7,46521603 | 2,19E-06 | 0,000384762 | Up |
| F6YR34 | THBS1 | Thrombospondin 1 | 1,57584055 | 10,136963 | 3,97E-30 | 1,53E-26 | Up |
| A0A3Q2HJQ9 | SLIT2 | Slit guidance ligand 2 | 1,56726343 | 7,24742804 | 5,79E-05 | 0,004759985 | Up |
| A0A9L0RV36 | TPBG | Trophoblast glycoprotein | 1,40964239 | 7,623093 | 2,78E-05 | 0,002753791 | Up |
| F6SK35 | GJA1 | Gap junction protein alpha 1 | 1,38790777 | 7,2081341 | 0,00073131 | 0,026918225 | Up |
| A0A9L0RET5 | PDGFRA | Platelet derived growth factor receptor alpha | 1,33494808 | 8,08222601 | 9,46E-06 | 0,001177693 | Up |
| F6RIZ2 | IGFBP7 | Insulin like growth factor binding protein 7 | 1,27708005 | 7,20794754 | 0,00106766 | 0,034640774 | Up |
| F6RUJ8 | CCN1 | Cellular communication network factor 1 | 1,27270987 | 7,2672645 | 0,00089985 | 0,031067812 | Up |
| A0A3Q2IDK5 | PLAT | Plasminogen activator, tissue type | 1,17574095 | 7,87171653 | 6,61E-05 | 0,005107894 | Up |
| A0A9L0R5F9 | ANTXR1 | ANTXR cell adhesion molecule 1 | 1,16862611 | 7,52905683 | 0,00137464 | 0,041102973 | Up |
| A0A9L0T021 | LUM | Lumican | 1,14345539 | 7,46436343 | 0,00108204 | 0,034814724 | Up |
| F7CW51 | SDCBP | Syndecan binding protein | 1,07396827 | 10,158092 | 3,13E-14 | 6,04E-11 | Up |
| A0A3Q2H0H3 | CACNA2D1 | Calcium voltage-gated channel auxiliary subunit alpha2delta 1 | 0,99948839 | 8,18927958 | 0,00010486 | 0,006637118 | Up |
| A0A5F5PLI4 | AGA | Aspartylglucosaminidase | 0,99576849 | 7,90855929 | 0,00082302 | 0,029422893 | Up |
| Q29482 | CLU | Clusterin | 0,91465871 | 8,74830664 | 2,06E-05 | 0,00226549 | Up |
| A0A9L0QYF4 | CTSK | Cathepsin K | 0,88500406 | 10,1883211 | 6,31E-12 | 8,12E-09 | Up |
| A0A3Q2HMD7 | UQCRC2 | Ubiquinol-cytochrome c reductase core protein 2 | 0,86809852 | 8,62565969 | 9,17E-05 | 0,00621089 | Up |
| F6X0K3 | CCN2 | Cellular communication network factor 2 | 0,85615626 | 8,47330313 | 0,00026371 | 0,01272727 | Up |
| A0A5F5PR03 | AK3 | Adenylate kinase 3 | 0,82501671 | 8,67722 | 0,00015267 | 0,008644151 | Up |
| A0A3Q2KSV4 | GPNMB | Glycoprotein nmb | 0,77347914 | 9,9425693 | 3,89E-08 | 2,14E-05 | Up |
| A0A3Q2HEQ2 | GPNMB | Glycoprotein nmb | 0,74161989 | 10,019002 | 5,94E-08 | 2,24E-05 | Up |
| A0A3Q2HP57 | IGF2R | Insulin like growth factor 2 receptor | 0,67245809 | 9,07189343 | 0,00030935 | 0,014565727 | Up |
| A0A5F5PLA1 | FASN | Fatty acid synthase | 0,66437954 | 10,0693377 | 0,00132877 | 0,040081112 | Up |
| A0A3Q2HIF9 | APP | Amyloid beta precursor protein | 0,66127243 | 9,16851261 | 0,00025351 | 0,012548548 | Up |
| A0A9L0SLM5 | MFGE8 | Lactadherin | 0,63873908 | 9,29766032 | 0,0001989 | 0,01081631 | Up |
| A0A3Q2HT17 | EDIL3 | EGF like repeats and discoidin domains 3 | 0,63742819 | 9,23996955 | 0,00023234 | 0,011803341 | Up |
| A0A3Q2GWX4 | ITGB5 | Integrin subunit beta 5 | 0,59216658 | 9,14404885 | 0,00111755 | 0,03536778 | Up |
| A0A9L0TIU4 | GLUD1 | Glutamate dehydrogenase 1, mitochondrial | 0,55392647 | 10,326982 | 6,24E-06 | 0,000891641 | Up |
| A0A9L0RNS2 | MMP14 | Matrix metalloproteinase-14 | 0,54616307 | 10,5489411 | 6,29E-05 | 0,005060433 | Up |
| F6U187 | ATP5F1B | ATP synthase subunit beta | 0,51541062 | 10,6116269 | 7,45E-06 | 0,001024043 | Up |
| Q28372 | GSN | Gelsolin | 0,47802625 | 10,4350692 | 4,69E-05 | 0,003938728 | Up |
| A0A3Q2GW51 | RNH1 | Ribonuclease inhibitor | 0,46617187 | 9,83538229 | 0,00094384 | 0,031966263 | Up |
| F7AZD1 | LOC100072592 | Purine nucleoside phosphorylase | 0,42379597 | 10,1620135 | 0,00084526 | 0,029940728 | Up |
| F6UPN4 | SPTAN1 | Spectrin alpha, non-erythrocytic 1 | 0,404351 | 11,0534337 | 0,00026196 | 0,01272727 | Up |
| F6UPM7 | SPTAN1 | Spectrin alpha, non-erythrocytic 1 | 0,40222163 | 11,0524622 | 0,00015448 | 0,008644151 | Up |
| A0A3Q2GZL9 | SPTAN1 | Spectrin alpha, non-erythrocytic 1 | 0,39353813 | 11,0536456 | 0,00027969 | 0,013331911 | Up |
| A0A5F5Q3H0 | ANXA5 | Annexin | 0,38742705 | 10,8387652 | 0,00124236 | 0,038373895 | Up |
| A0A9L0TKA3 | ATP | ATP synthase subunit beta, mitochondrial | 0,3772779 | 10,5905105 | 0,00098249 | 0,032842111 | Up |

**Proteoforms Sequence Alignment by CLUSTALW**

CLUSTAL 2.1 Multiple Sequence Alignments

**Protein: aldehyde dehydrogenase (NAD(+))**

Sequence type explicitly set to Protein

Sequence format is Pearson

Sequence 1: **A0A3Q2IDL1** 484 aa

Sequence 2: **A0A3Q2H6U5** 463 aa

Start of Pairwise alignments

Aligning...

Sequences (1:2) Aligned. Score: 93.0886

Guide tree file created: [[clustalw.dnd]](https://www.genome.jp/tools-bin/pushfile?250213053355oeHbr+clustalw.dnd)

There are 1 groups

Start of Multiple Alignment

Aligning...

Group 1: Sequences: 2 Score:7069

Alignment Score 2664

CLUSTAL-Alignment file created [[clustalw.aln]](https://www.genome.jp/tools-bin/pushfile?250213053355oeHbr+clustalw.aln)

[clustalw.aln](https://www.genome.jp/tools-bin/pushfile?250213053355oeHbr+clustalw.aln)

CLUSTAL 2.1 multiple sequence alignment

A0A3Q2IDL1 MATTNGAVENGQPDRKPPALPRPIRNLEVKFTKIFINNEWHESKSGKKFATYNPSTLEKI

A0A3Q2H6U5 MATTNGAVENGQPDRKPPALPRPIRNLEVKFTKIFINNEWHESKSGKKFATYNPSTLEKI

************************************************************

A0A3Q2IDL1 CEVEEGDKPDVDKAVEAAQAAFQRGSPWRRLDALSRGRLLHQLADLVERDRAVLATLETM

A0A3Q2H6U5 CEVEEGDKPDVDKAVEAAQAAFQRGSPWRRLDALSRGRLLHQLADLVERDRAVLATLETM

************************************************************

A0A3Q2IDL1 DSGKPFLHAFFIDLEGCIKTLRYFAGWADKIQGRTIP-----------------------

A0A3Q2H6U5 DSGKPFLHAFFIDLEGCIKTLRYFAGWADKIQGRTIPTDDNVVCFTRHEPVGVCGAITPW

*************************************

A0A3Q2IDL1 -----TVVWKLAPALCCGNTVVVKPAEQTPLTALYLGSLIKEVGFPPGVVNIVPGFGPTA

A0A3Q2H6U5 NFPLLMLVWKLAPALCCGNTVVVKPAEQTPLTALYLGSLIKEVGFPPGVVNIVPGFGPTA

:*****************************************************

A0A3Q2IDL1 GAAISSHPQISKIAFTGSTEVGKLVKEASSQSNLKRVTLELGGKNPCIVCADADLDLAVE

A0A3Q2H6U5 GAAISSHPQISKIAFTGSTEVGKLVKEASSQSNLKRVTLELGGKNPCIVCADADLDLAVE

************************************************************

A0A3Q2IDL1 CAHQGVFFNQGQCCTAASRVFVEEQVYADFVRRSVEYAKKRPVGDPFDVRTEQGPQIDQK

A0A3Q2H6U5 CAHQGVFFNQGQCCTAASRVFVEEQVYADFVRRSVEYAKKRPVGDPFDVRTEQGPQIDQK

************************************************************

A0A3Q2IDL1 QFNKILDLIDSGKEEGAKLECGGSAMEDRGLFIKPTVFSEVTDTMRIAREEIFGPVQPIL

A0A3Q2H6U5 QFNKILDLIDSGKEEGAKLECGGSAMEDRGLFIKPTVFSEVTDTMRIAREEIFGPVQPIL

************************************************************

A0A3Q2IDL1 KFKSIEEVIKRANSLEYGLTAAVFTKNLDKALKLASALEAGTVWINCYNALYAQAPFGGF

A0A3Q2H6U5 KFKSIEEVIKRANSLEYGLTAAVFTKNLDKALKLASALEAGTV-----------------

*******************************************

A0A3Q2IDL1 KMSGNGRELGEYALTEYTEVKTVTIKLDDKNP

A0A3Q2H6U5 --------------------------------

CLUSTAL 2.1 Multiple Sequence Alignments

**Protein: ATP synthase subunit beta**

Sequence type explicitly set to Protein

Sequence format is Pearson

Sequence 1: **F6U187** 487 aa

Sequence 2: **A0A9L0TKA3** 530 aa

Start of Pairwise alignments

Aligning...

Sequences (1:2) Aligned. Score: 84.5996

Guide tree file created: [[clustalw.dnd]](https://www.genome.jp/tools-bin/pushfile?250213053934GXjeV+clustalw.dnd)

There are 1 groups

Start of Multiple Alignment

Aligning...

Group 1: Sequences: 2 Score:6738

Alignment Score 2401

CLUSTAL-Alignment file created [[clustalw.aln]](https://www.genome.jp/tools-bin/pushfile?250213053934GXjeV+clustalw.aln)

[clustalw.aln](https://www.genome.jp/tools-bin/pushfile?250213053934GXjeV+clustalw.aln)

CLUSTAL 2.1 multiple sequence alignment

F6U187 -------------------------------------------------------MLGLV

A0A9L0TKA3 MVEEMRDCQPIGAALRVMGSKFIQWTCLLQSRPPPNCRRVGAGTAAGLSLHPDSAMLGLV

*****

F6U187 GRVAAASASGALRGLSPSAPLPQAQLLLRAAPAALQPARDYAAQTSPAPKAGAATGRIVA

A0A9L0TKA3 GRVAAASASGALRGLSPSAPLPQAQLLLRAAPAALQPARDYAAQTSPAPKAGAATGRIVA

************************************************************

F6U187 VIGAVVDVQFDEGLPPILNALEVQGRETRLVLEVAQHLGESTVRTIAMDGTEGLVRGQKV

A0A9L0TKA3 VIGAVVDVQFDEGLPPILNALEVQGRETRLVLEVAQHLGESTVRTIAMDGTEGLVRGQKV

************************************************************

F6U187 LDSGAPIKIPVGPETLGRIMNVIGEPIDERGPIKTKQFAAIHAEAPEFMEMSVEQEILVT

A0A9L0TKA3 LDSGAPIKIPVGPETLGRIMNVIGEPIDERGPIKTKQFAAIHAEAPEFMEMSVEQEILVT

************************************************************

F6U187 GIKVVDLLAPYAKGGKIGLFGGAGVGKTVLIMELINNVAKAHGGYSVFAGVGERTREGND

A0A9L0TKA3 GIKVVDLLAPYAKGGKIGLFGGAGVGKTVLIMELINNVAKAHGGYSVFAGVGERTREGND

************************************************************

F6U187 LYHEMIESGVINLKDATSKVALVYGQMNEPPGARARVALTGLTVAEYFRDQEGQDVLLFI

A0A9L0TKA3 LYHEMIESGVINLKDAT-------------------------------------------

*****************

F6U187 DNIFRFTQAGSEVSALLGRIPSAVGYQPTLATDMGTMQERITTTKKGSITSVQAIYVPAD

A0A9L0TKA3 ----------SKVSALLGRIPSAVGYQPTLATDMGTMQERITTTKKGSITSVQAIYVPAD

*:************************************************

F6U187 DLTDPAPATTFAHLDATTVLSRAIAELGIYPAVDPLDSTSRIMDPNIVGTEHYEVARGVQ

A0A9L0TKA3 DLTDPAPATTFAHLDATTVLSRAIAELGIYPAVDPLDSTSRIMDPNIVGTEHYEVARGVQ

************************************************************

F6U187 KILQDYKSLQDIIAILGMDELSEEDKLTVSRARKIQRFLSQPFGEVGTPERDHQRIPADF

A0A9L0TKA3 KILQDYKSLQDIIAILGMDELSEEDKLTVSRARKIQRFLSQPFQVAEVFTGHLGKLVPLK

******************************************* . . . :: .

F6U187 GR-----------------------------------------

A0A9L0TKA3 ETIKGFQQILAGDYDHLPEQAFYMVGPIEEAVAKADKLAEEHS

CLUSTAL 2.1 Multiple Sequence Alignments

**Protein: Calpastatin**

Sequence type explicitly set to Protein

Sequence format is Pearson

Sequence 1: **A0A3Q2IF55** 779 aa

Sequence 2: **A0A3Q2LD09**  785 aa

Start of Pairwise alignments

Aligning...

(Partial alignment)

Sequences (1:2) Aligned. Score: 85.8793

Guide tree file created: [[clustalw.dnd]](https://www.genome.jp/tools-bin/pushfile?250213054434czipg+clustalw.dnd)

There are 1 groups

Start of Multiple Alignment

Aligning...

Group 1: Sequences: 2 Score:12401

Alignment Score 4442

CLUSTAL-Alignment file created [[clustalw.aln]](https://www.genome.jp/tools-bin/pushfile?250213054434czipg+clustalw.aln)

[clustalw.aln](https://www.genome.jp/tools-bin/pushfile?250213054434czipg+clustalw.aln)

CLUSTAL 2.1 multiple sequence alignment

A0A3Q2IF55 ---------------MYDSCHKVNNILSYCNKKATSLGSSQPSRTHAGETAPATKVSTSF

A0A3Q2LD09 MAFASWWYQTHVNEKTSGSPSKSGEKKGSDEKKATSLGSSQPSRTHAGETAPATKVSTSF

.* * .: . :*****************************

A0A3Q2IF55 ASTSKSYSMNPTETKAIPVSKQMEGPHSPNKKRHRKQAVKTEPEKKSQSTKPSVVHEKKT

A0A3Q2LD09 ASTSKSYSMNPTETKAIPVSKQMEGPHSPNKKRHRKQAVKTEPEKKSQSTKPSVVHEKKT

************************************************************

A0A3Q2IF55 QEVKPKEHTEPKSLPKHTSDAGSKDAHQEKAVSTSSEQLKSEKSAKPKAKSQDTIPADGK

A0A3Q2LD09 QEVKPKEHTEPKSLPKHTSDAGSKDAHQEKAVSTSSEQLKSEKSAKPKAKSQDTIPADGK

************************************************************

A0A3Q2IF55 TVVAGVAAASGKPDDKKKESKSLTSAVPVESKPEKPSGESGIDAALNDLIDTLGEPEGAE

A0A3Q2LD09 TVVAGVAAASGKPDDKKKESKSLTSAVPVESKPEKPSGESGIDAALNDLIDTLGEPEGAE

************************************************************

A0A3Q2IF55 EDTTTYTGPEVLDPMSSTFIEELGKREVTLPPKYRELLAKKGVPGPPPDSLTPVGPDDAI

A0A3Q2LD09 EDTTTYTGPEVLDPMSSTFIEELGKREVTLPPKYRELLAKKGVPGPPPDSLTPVGPDDAI

************************************************************

A0A3Q2IF55 DALASDFTCSSPTASGQKTEEEKSTGEVLKAQSAGFVKSPAPPQEKKRKVEEDAISDQAL

A0A3Q2LD09 DALASDFTCSSPTASGQKTEEEKSTGEVLKAQSAGFVKSPAPPQEKKRKVEEDAISDQAL

************************************************************

A0A3Q2IF55 EALSASLGSPKPDPEPDFSSLKEVDEAKAKEEKLKKCGEDDETVPSEYRLKPATDKDGKP

A0A3Q2LD09 EALSASLGSPKPDPEPDFSSLKEVDEAKAKEEKLKKCGEDDETVPSEYRLKPATDKDGKP

************************************************************

A0A3Q2IF55 LLPESEEKPKPLSESELIDELSEDFDQSKRKEKQLKPAEKTKESRAAAPAPVGEAVSQTS

A0A3Q2LD09 LLPESEEKPKPLSESELIDELSEDFDQSKRKEKQLKPAEKTKESRAAAPAPVGEAVSQTS

************************************************************

A0A3Q2IF55 MCSVQSAPPKPATVVSDPLGIEKKGTVPDDAVEALAGSLGKKEADPEGGKPVEDQVKEKS

A0A3Q2LD09 MCSVQSAPPKPATVK---------GTVPDDAVEALAGSLGKKEADPEGGKPVEDQVKEKS

************** ************************************

A0A3Q2IF55 KEEDREKLGEKEETIPPDYRLEEVKDKDGKPLLHKDPKESLPPLSDDFLLDALSEDFAGP

A0A3Q2LD09 KEEDREKLGEKEETIPPDYRLEEVKDKDGKPLLHKDPKESLPPLSDDFLLDALSEDFAGP

************************************************************

A0A3Q2IF55 QDTSSLQKQFEDAKLSAVISEVVSQTPAPTTHAARPPPDALQSDNKELDDALDQLSDSLG

A0A3Q2LD09 QDTSSLQKQFEDAKLSAVISEVVSQTPAPTTHAARPPPDALQSDNKELDDALDQLSDSLG

************************************************************

A0A3Q2IF55 QRQPDPDENKPVEDKVKEKVKAEHRDKLGERDDTIPPEYRHLLDKNDEGKPVKPPEKKPG

A0A3Q2LD09 QRQPDPDENKPVEDKVKEKVKAEHRDKLGERDDTIPPEYRHLLDKNDEGKPVKPPEKKPG

************************************************************

A0A3Q2IF55 ESKKTADDQDPIDALSGDFDSCPSTTESSENTAKDKDKKNASSSKAPKTGGKAKDSAKAK

A0A3Q2LD09 ESKKTADDQDPIDALSGDFDSCPSTTESSENTAKDKDKKNASSSKAPKTGGKAKDSAKAK

************************************************************

A0A3Q2IF55 KETSKPKADEKKTS

A0A3Q2LD09 KETSKPKADEKKTS

**************

CLUSTAL 2.1 Multiple Sequence Alignments

**Protein: Collagen type V alpha 1 chain**

Sequence type explicitly set to Protein

Sequence format is Pearson

Sequence 1: **A0A9L0R7P4** 1718 aa

Sequence 2: **A0A5F5PRZ7** 1843 aa

Start of Pairwise alignments

Aligning...

Sequences (1:2) Aligned. Score: 99.2433

Guide tree file created: [[clustalw.dnd]](https://www.genome.jp/tools-bin/pushfile?250213054720PWqo5+clustalw.dnd)

There are 1 groups

Start of Multiple Alignment

Aligning...

Group 1: Sequences: 2 Score:29415

Alignment Score 11569

CLUSTAL-Alignment file created [[clustalw.aln]](https://www.genome.jp/tools-bin/pushfile?250213054720PWqo5+clustalw.aln)

[clustalw.aln](https://www.genome.jp/tools-bin/pushfile?250213054720PWqo5+clustalw.aln)

CLUSTAL 2.1 multiple sequence alignment

A0A9L0R7P4 MDVHTRWKARSPLRPGAPLLSPLLLLLLLWAPPPSRAAQPADLLKVLDFHNLPDGITKTT

A0A5F5PRZ7 MDVHTRWKARSPLRPGAPLLSPLLLLLLLWAPPPSRAAQPADLLKVLDFHNLPDGITKTT

************************************************************

A0A9L0R7P4 GFCATRRSSKGPDVAYRVTKDAQLSAPTKQLYPASAFPEDFSILTTVKAKKGSQAFLVSI

A0A5F5PRZ7 GFCATRRSSKGPDVAYRVTKDAQLSAPTKQLYPASAFPEDFSILTTVKAKKGSQAFLVSI

************************************************************

A0A9L0R7P4 YNEQGIQQIGLEMGRSPVFLYEDHTGKPGPEDYPLFRGINLSDGKWHRIALSVHKKNVTL

A0A5F5PRZ7 YNEQGIQQIGLEMGRSPVFLYEDHTGKPGPEDYPLFRGINLSDGKWHRIALSVHKKNVTL

************************************************************

A0A9L0R7P4 ILDCKKKTTKFLDRSDHPMIDVNGIIVFGTRILDEEVFEGDIQQLLFVSDHRAAYDYCEH

A0A5F5PRZ7 ILDCKKKTTKFLDRSDHPMIDVNGIIVFGTRILDEEVFEGDIQQLLFVSDHRAAYDYCEH

************************************************************

A0A9L0R7P4 YSPDCDTAVPDTPQSQDPNPDEYYPEGENEGETYYYEYPYYEDTEDVSKEPPPTKTPVEA

A0A5F5PRZ7 YSPDCDTAVPDTPQSQDPNPDEYYPEGENEGETYYYEYPYYEDTEDVSKEPPPTKTPVEA

************************************************************

A0A9L0R7P4 ARETTEIAEELTLPPTEAAPVTDASEGPGKEDDVGIEDYDYVPSEDYYTPPPYEDLNYGE

A0A5F5PRZ7 ARETTEIAEELTLPPTEAAPVTDASEGPGKEDDVGIEDYDYVPSEDYYTPPPYEDLNYGE

************************************************************

A0A9L0R7P4 GLENPDENPDQLPEPRARAEVPTSTVSTSNGSNPAPPPEEGRDDLEGEFTEETIKNLDEN

A0A5F5PRZ7 GLENPDENPDQLPEPRARAEVPTSTVSTSNGSNPAPPPEEGRDDLEGEFTEETIKNLDEN

************************************************************

A0A9L0R7P4 YYDPYYDPTVSPSEIGPGMPANQDTIYEGIGGPRGEKGQKGEPAIIEPGMLLEGPPGPEG

A0A5F5PRZ7 YYDPYYDPTVSPSEIGPGMPANQDTIYEGIGGPRGEKGQKGEPAIIEPGMLLEGPPGPEG

************************************************************

A0A9L0R7P4 PAGLPGPPGTTGPTGQVGDPGERGPPGRPGLPGADGLPGPPGTMLMLPFRFGGGGDAGSK

A0A5F5PRZ7 PAGLPGPPGTTGPTGQVGDPGERGPPGRPGLPGADGLPGPPGTMLMLPFRFGGGGDAGSK

************************************************************

A0A9L0R7P4 GPMVSAQESQAQAILQQARLALRGPAGPMGLTGRPGPMGPPGSGGLKGEPGDMGPQGPRG

A0A5F5PRZ7 GPMVSAQESQAQAILQQARLALRGPAGPMGLTGRPGPMGPPGSGGLKGEPGDMGPQGPRG

************************************************************

A0A9L0R7P4 VQGPPGPAGKPGRRGRAGSDGARGMPGQTGPKGDRGFDGLAGLPGEKGHRGDPGPSGPPG

A0A5F5PRZ7 VQGPPGPAGKPGRRGRAGSDGARGMPGQTGPKGDRGFDGLAGLPGEKGHRGDPGPSGPPG

************************************************************

A0A9L0R7P4 PPGDDGERGDDGEVGPRGLPGEPGPRGLLGPKGPPGPPGPPGVTGMDGQPGLKGNVGPQG

A0A5F5PRZ7 PPGDDGERGDDGEVGPRGLPGEPGPRGLLGPKGPPGPPGPPGVTGMDGQPGLKGNVGPQG

************************************************************

A0A9L0R7P4 EPGPPGQQGNPGAQGLPGPQGAIGPPGEKGPLGKPGLPGMPGADGPPGHPGKEGPPGEKG

A0A5F5PRZ7 EPGPPGQQGNPGAQGLPGPQGAIGPPGEKGPLGKPGLPGMPGADGPPGHPGKEGPPGEKG

************************************************************

A0A9L0R7P4 GQGPPGPQGPIGYPGPRGVKGADGIRGLKGTKGEKGEDGFPGFKGDMGIKGDRGEIGPPG

A0A5F5PRZ7 GQGPPGPQGPIGYPGPRGVKGADGIRGLKGTKGEKGEDGFPGFKGDMGIKGDRGEIGPPG

************************************************************

A0A9L0R7P4 PRGEDGPEGPKGRGGPNGDPGPLGPPGEKGKLGVPGLPGYPGRQGPKGSIGFPGFPGANG

A0A5F5PRZ7 PRGEDGPEGPKGRGGPNGDPGPLGPPGEKGKLGVPGLPGYPGRQGPKGSIGFPGFPGANG

************************************************************

A0A9L0R7P4 EKGGRGTPGKPGPRGQRGPTGPRGERGPRGITGKPGPKGNSGGDGPAGPPGERGPNGPQG

A0A5F5PRZ7 EKGGRGTPGKPGPRGQRGPTGPRGERGPRGITGKPGPKGNSGGDGPAGPPGERGPNGPQG

************************************************************

A0A9L0R7P4 PTGFPGPKGPPGPPGKDGLPGHPGQRGETGFQGKTGPPGPPGVVGPQGPTGETGPMGERG

A0A5F5PRZ7 PTGFPGPKGPPGPPGKDGLPGHPGQRGETGFQGKTGPPGPPGVVGPQGPTGETGPMGERG

************************************************************

A0A9L0R7P4 HPGPPGPPGEQGLPGVAGKEGTKGDPGPAGLPGKDGPPGLRGFPGDRGLPGPVGALGLKG

A0A5F5PRZ7 HPGPPGPPGEQGLPGVAGKEGTKGDPGPAGLPGKDGPPGLRGFPGDRGLPGPVGALGLKG

************************************************************

A0A9L0R7P4 NEGPPGPPGPAGSPGERGPAGAAGPIGIPGRPGPQGPPGPAGEKGAPGEKGPQGPAGRDG

A0A5F5PRZ7 NEGPPGPPGPAGSPGERGPAGAAGPIGIPGRPGPQGPPGPAGEKGAPGEKGPQGPAGRDG

************************************************************

A0A9L0R7P4 LQGPVGLPGPAGPVGPPGEDGDKGEIGEPGQKGSKGDKGEQGPPGPTGPQGPIGQPGPSG

A0A5F5PRZ7 LQGPVGLPGPAGPVGPPGEDGDKGEIGEPGQKGSKGDKGEQGPPGPTGPQGPIGQPGPSG

************************************************************

A0A9L0R7P4 ADGEPGPRGQQGLFGQKGDEGPRGFPGPPGPVGLQGLPGPPGEKGETGDVGQMGPPGPPG

A0A5F5PRZ7 ADGEPGPRGQQGLFGQKGDEGPRGFPGPPGPVGLQGLPGPPGEKGETGDVGQMGPPGPPG

************************************************************

A0A9L0R7P4 PRGPSGAPGADGPQGPPGGIGNPGAVGEKGEPGEAGEPGLPGEGGPPGPKGERGEKGESG

A0A5F5PRZ7 PRGPSGAPGADGPQGPPGGIGNPGAVGEKGEPGEAGEPGLPGEGGPPGPKGERGEKGESG

************************************************************

A0A9L0R7P4 PSGAAGPPGPKGPPGDDGPKGSPGPVGFPGDPGPPGEPGPAGQDGPPGDKGDDGEPGQTG

A0A5F5PRZ7 PSGAAGPPGPKGPPGDDGPKGSPGPVGFPGDPGPPGEPGPAGQDGPPGDKGDDGEPGQTG

************************************************************

A0A9L0R7P4 SPGPTGEPGPSGPPGKRGPPGPAGPEGRQGEKGAKGEAGLEGPPGKTGPIGPQGAPGKPG

A0A5F5PRZ7 SPGPTGEPGPSGPPGKRGPPGPAGPEGRQGEKGAKGEAGLEGPPGKTGPIGPQGAPGKPG

************************************************************

A0A9L0R7P4 PDGLRGIPGPVGEQGLPGSPGPDGPPGPMGPPGLPGLKGDSGPKGEKGHPGLIGLIGPPG

A0A5F5PRZ7 PDGLRGIPGPVGEQGLPGSPGPDGPPGPMGPPGLPGLKGDSGPKGEKGHPGLIGLIGPPG

************************************************************

A0A9L0R7P4 EQGEKGDRGLPGPQGSSGPKGEQGITGPSGPIGPPGPPGLPGPPGPKGAKGSSGPTGPKG

A0A5F5PRZ7 EQGEKGDRGLPGPQGSSGPKGEQGITGPSGPIGPPGPPGLPGPPGPKGAKGSSGPTGPKG

************************************************************

A0A9L0R7P4 EAGHPGPPGPPGPPGEVIQPLPIQASRTRRNIDASQMLDDGDGENYMDYADGMEEIFGSL

A0A5F5PRZ7 EAGHPGPPGPPGPPGEVIQPLPIQASRTRRNIDASQMLDDGDGENYMDYADGMEEIFGSL

************************************************************

A0A9L0R7P4 NSLKLEIEQMKRPLGTQQNPARTCKDLQLCHPDFPDGEYWVDPNQGCSRDSFKVYCNFTA

A0A5F5PRZ7 NSLKLEIEQMKRPLGTQQNPARTCKDLQLCHPDFPDGEYWVDPNQGCSRDSFKVYCNFTA

************************************************************

A0A9L0R7P4 GGATCVFPDKKSEGSKMARWPKEQPSTWYSQYKRGSLI----------------------

A0A5F5PRZ7 GGATCVFPDKKSEGARITSWPKENPGSWFSEFKRGKLLSYVDAEGNPVGVVQMTFLRLLS

**************:::: ****:*.:*:*::***.*:

A0A9L0R7P4 ------------------------------------------------------------

A0A5F5PRZ7 ASAHQNITYNCYQSVAWQDAATGSYDKAMRFLGSNDEEMSFDNSPYIRALVDGCATRKGY

A0A9L0R7P4 -------------------------------------------

A0A5F5PRZ7 QKTVLEIDTPKVEQVPIVDIMFNDFGEASQKFGFEVGPACFLG

CLUSTAL 2.1 Multiple Sequence Alignments

**Protein: Family with sequence similarity 114 member A1**

Sequence type explicitly set to Protein

Sequence format is Pearson

Sequence 1: **A0A9L0S7Y5** 517 aa

Sequence 2: **A0A9L0SXF6** 642 aa

Start of Pairwise alignments

Aligning...

Sequences (1:2) Aligned. Score: 98.2592

Guide tree file created: [[clustalw.dnd]](https://www.genome.jp/tools-bin/pushfile?250213055223Uk16x+clustalw.dnd)

There are 1 groups

Start of Multiple Alignment

Aligning...

Group 1: Sequences: 2 Score:8188

Alignment Score 3025

CLUSTAL-Alignment file created [[clustalw.aln]](https://www.genome.jp/tools-bin/pushfile?250213055223Uk16x+clustalw.aln)

[clustalw.aln](https://www.genome.jp/tools-bin/pushfile?250213055223Uk16x+clustalw.aln)

CLUSTAL 2.1 multiple sequence alignment

A0A9L0S7Y5 MSDDVGDTLATGEKAEITEMPSSGSLPKDAEVHCDSATISNEPTPADPRGDEHENAAIQG

A0A9L0SXF6 MSDDVGDTLATGEKAEITEMPSSGSLPKDAEVHCDSATISNEPTPADPRGDEHENAAIQG

************************************************************

A0A9L0S7Y5 AETADIRHPEQPKDISAMEPPVNGEVTEDTLTECIDSVSLEAEPGSEIPLKEQSNPAVDS

A0A9L0SXF6 AETADIRHPEQPKDISAMEPPVNGEVTEDTLTECIDSVSLEAEPGSEIPLKEQSNPAVDS

************************************************************

A0A9L0S7Y5 PPHGGGWAGWGSWGKSLLSSASATVGHGLTAVKEKAGATLRIHSVNSSSSEGAQTDTENG

A0A9L0SXF6 PPHGGGWAGWGSWGKSLLSSASATVGHGLTAVKEKAGATLRIHSVNSSSSEGAQTDTENG

************************************************************

A0A9L0S7Y5 VPQTDAATDESPAESPPASPSSGSRGVLSAITSVVQNT----------------------

A0A9L0SXF6 VPQTDAATDESPAESPPASPSSGSRGVLSAITSVVQNTGKSVLTGGLDALEFIGKKTMNV

**************************************

A0A9L0S7Y5 -----------------------MLREAKEKEKQRLAQQLTAERTAHYGMLFDEYQGLSH

A0A9L0SXF6 LAESDPGFKRTKTLMERTVSLSQMLREAKEKEKQRLAQQLTAERTAHYGMLFDEYQGLSH

*************************************

A0A9L0S7Y5 LEALEILSNESESKVQSFLSSLDGEKLELLKNDLISIKDIFAAKELENEENQEEQG--LE

A0A9L0SXF6 LEALEILSNESESKVQSFLSSLDGEKLELLKNDLISIKDIFAAKELENEENQEEQGKGLE

******************************************************** **

A0A9L0S7Y5 EKGEEFASMLTELLFELHVAATPDKLN---------------------------------

A0A9L0SXF6 EKGEEFASMLTELLFELHVAATPDKLNKHAVVDTGPHLSKWVRKGQKEAVRWRESNFFIS

***************************

A0A9L0S7Y5 ---------------------------------------------KAMKKAHDCMDEDQT

A0A9L0SXF6 GRGHRLWQVNDLNTVGNCLIHLRAAPATAQSVYDASKVPLVYLHSQAMKKAHDCMDEDQT

:**************

A0A9L0S7Y5 VVSVDVAEESEEKTKKEEGEEKPENPKEDGKEGRRTKTVEEVYMLSIESLAEVTARCIEQ

A0A9L0SXF6 VVSVDVAEESEEKTKKEEGEEKPENPKEDGKEGRRTKTVEEVYMLSIESLAEVTARCIEQ

************************************************************

A0A9L0S7Y5 LHKVAELILHGQEEEKSAQYQAKVLIKLTTAMCKEVASLSKKFTNSLTTVGSNKKAEVLN

A0A9L0SXF6 LHKVAELILHGQEEEKSAQYQAKVLIKLTTAMCKEVASLSKKFTNSLTTVGSNKKAEVLN

************************************************************

A0A9L0S7Y5 PMINSVLLEGCNSMTYVQDAFQLLLPVLQVSHIQTSCSKAQP

A0A9L0SXF6 PMINSVLLEGCNSMTYVQDAFQLLLPVLQVSHIQTSCSKAQP

******************************************

CLUSTAL 2.1 Multiple Sequence Alignments

**Protein:Transmembrane glycoprotein NMB**

Sequence type explicitly set to Protein

Sequence format is Pearson

Sequence 1: **A0A3Q2KSV4**  527 aa

Sequence 2: **A0A3Q2HEQ2** 521 aa

Start of Pairwise alignments

Aligning..

Sequences (1:2) Aligned. Score: 83.6852

Guide tree file created: [[clustalw.dnd]](https://www.genome.jp/tools-bin/pushfile?250213055645W3yA8+clustalw.dnd)

There are 1 groups

Start of Multiple Alignment

Aligning...

Group 1: Sequences: 2 Score:7650

Alignment Score 2797

CLUSTAL-Alignment file created [[clustalw.aln]](https://www.genome.jp/tools-bin/pushfile?250213055645W3yA8+clustalw.aln)

[clustalw.aln](https://www.genome.jp/tools-bin/pushfile?250213055645W3yA8+clustalw.aln)

CLUSTAL 2.1 multiple sequence alignment

A0A3Q2KSV4 MECVYCFLGFLLLAARLPLDAAKRFHDVLSNERPSGYMREHNQLKGWSSDENDWNEKLYP

A0A3Q2HEQ2 -------------------------------------MREHNQLKGWSSDENDWNEKLYP

***********************

A0A3Q2KSV4 VWKRGDPRWKNSWKGGRVQAVLTSDSPALVGSTMTFVVSLVFPRCQKEDANGNIVYETNC

A0A3Q2HEQ2 VWKRGDPRWKNSWKGGRVQAVLTSDSPALVGSTMTFVVSLVFPRCQKEDANGNIVYETNC

************************************************************

A0A3Q2KSV4 RNDTGPSPDPFVYNWTAWTEDDDWGNDSSQGHHNVFPDGKPFPRPPGWKKRNFVYVFHTL

A0A3Q2HEQ2 RNDTGPSPDPFVYNWTAWTEDDDWGNDSSQGHHNVFPDGKPFPRPPGWKKRNFVYVFHTL

************************************************************

A0A3Q2KSV4 GQYFQRLGRCSARVSINTTNVPLGPQLMEVTVYRRHGRAYCPIAKVKDVYVVTDRIPIFV

A0A3Q2HEQ2 GQYFQRLGRCSARVSINTTNVPLGPQLMEVTVYRRHGRAYCPIAKVKDVYVVTDRIPIFV

************************************************************

A0A3Q2KSV4 TMSQKNDRNSSDETFLRDLPIVFNVLIHDPSHFLIESSINYKWNFGDNTGLFVSNNSTLN

A0A3Q2HEQ2 TMSQKNDRNSSDETFLRDLPIVFNVLIHDPSHFLIESSINYKWNFGDNTGLFVSNNSTLN

************************************************************

A0A3Q2KSV4 HTYVLNGTFSLNLTVQAAVPGPCPTPSPRPLTPTPALLPGGDNPLQLRDVPDENCRINRY

A0A3Q2HEQ2 HTYVLNGTFSLNLTVQAAVPGPCPTPSPRPLTPTPALLPGGDNPLQLRDVPDENCRINRY

************************************************************

A0A3Q2KSV4 GYFKATIEIVDGILEVNIIKMTDVLMPVPQPDDSVVDFVVTCEGTIPTEVCTVISDPTCR

A0A3Q2HEQ2 GYFKATIEIVDGILEVNIIKMTDVLMPVPQPDDSVVDFVVTCEGTIPTEVCTVISDPTCR

************************************************************

A0A3Q2KSV4 ITQSTVCDPVDVDVGELCLLTVRRAFSGSGTYCMNLTLGDEASLALTSTLKTQG------

A0A3Q2HEQ2 ITQSTVCDPVDVDVGELCLLTVRRAFSGSGTYCMNLTLGDEASLALTSTLVSVPGRDPAS

************************************************** :

A0A3Q2KSV4 ------------------------LQTDRKQYWDRGQRKEAECFSQPCKGCVLPRKPGKR

A0A3Q2HEQ2 PLRIANGVLFSVGCLTIVVTVIALLVYKKHKDYRPIENSTGIVVKGKRLNVFLNRAKAVF

* .::: : :.. . .. . .* * .

A0A3Q2KSV4 STAQEPPRNSLNLQPYS-

A0A3Q2HEQ2 SRGNQEKDPLLKNHPGTL

* .:: *: :* :

CLUSTAL 2.1 Multiple Sequence Alignments

**Protein:** **Isoleucine--tRNA ligase, cytoplasmic**

Sequence type explicitly set to Protein

Sequence format is Pearson

Sequence 1: **A0A5F5PJP7** 1230 aa

Sequence 2: **A0A5F5PJ23**  1455 aa

Start of Pairwise alignments

Aligning...

Sequences (1:2) Aligned. Score: 99.7561

Guide tree file created: [[clustalw.dnd]](https://www.genome.jp/tools-bin/pushfile?250213060207UGf0r+clustalw.dnd)

There are 1 groups

Start of Multiple Alignment

Aligning...

Group 1: Sequences: 2 Score:20269

Alignment Score 7694

CLUSTAL-Alignment file created [[clustalw.aln]](https://www.genome.jp/tools-bin/pushfile?250213060207UGf0r+clustalw.aln)

[clustalw.aln](https://www.genome.jp/tools-bin/pushfile?250213060207UGf0r+clustalw.aln)

CLUSTAL 2.1 multiple sequence alignment

A0A5F5PJP7 ------------------------------------------------------------

A0A5F5PJ23 MCRDGARALQVGGRESARGRAARTLVQRRSRAPRSWRAARACLPADSGVTVRARRTGNSP

A0A5F5PJP7 ------------------------------------------------------------

A0A5F5PJ23 SVGHSRACRRGAQPRTARSRLRNVSPGATSLDSTQAGRSAQDTERRHTFAGPEPRGRLPG

A0A5F5PJP7 ------------------------------------------------------------

A0A5F5PJ23 THRGTARGRGRYERPIGTLLVGGCRRRVLIGPCVPAPPSWLRAVRALHHSPAGTRRGRCC

A0A5F5PJP7 -------------MVQQVPENINFPAEEEKILQFWSEFNCFQECLKQSKHRPKYTFYDGP

A0A5F5PJ23 FPRATVRETLSNKMVQQVPENINFPAEEEKILQFWSEFNCFQECLKQSKHRPKYTFYDGP

***********************************************

A0A5F5PJP7 PFATGLPHYGHILAGTIKDIVTRYAHQSGFHVDRRFGWDCHGLPVEYEIDKTLGIRGPED

A0A5F5PJ23 PFATGLPHYGHILAGTIKDIVTRYAHQSGFHVDRRFGWDCHGLPVEYEIDKTLGIRGPED

************************************************************

A0A5F5PJP7 VAKMGIVEYNNQCRAIVMRYSTEWKSIVTRLGRWIDFDNDYKTLYPQFMESVWWVFKQLY

A0A5F5PJ23 VAKMGIVEYNNQCRAIVMRYSTEWKSIVTRLGRWIDFDNDYKTLYPQFMESVWWVFKQLY

************************************************************

A0A5F5PJP7 DKGLVYRGVKVMPFSTACNTPLSNFESHQNYKDVQDPSVFVTFPLEEDENISLVAWTTTP

A0A5F5PJ23 DKGLVYRGVKVMPFSTACNTPLSNFESHQNYKDVQDPSVFVTFPLEEDENISLVAWTTTP

************************************************************

A0A5F5PJP7 WTLPSNLALCVNPDMQYVKIKDVVRGKLLILMEARLSALYKLESDYEILERFPGAYLKGK

A0A5F5PJ23 WTLPSNLALCVNPDMQYVKIKDVVRGKLLILMEARLSALYKLESDYEILERFPGAYLKGK

************************************************************

A0A5F5PJP7 KYTPLFDYFVKD--------------------------------DYRVCMDFGIIQKDSL

A0A5F5PJ23 KYTPLFDYFVKCKENGAFTVLVDNYVREEEGTGVVHQAPYFGADDYRVCMDFGIIQKDSL

*********** ****************

A0A5F5PJP7 PICPVDASGCFTAEVTDFMGQYVKDADKNIIRTLKEHGRLLVASTFTHSYPFCWRSDTPL

A0A5F5PJ23 PICPVDASGCFTAEVTDFMGQYVKDADKNIIRTLKEHGRLLVASTFTHSYPFCWRSDTPL

************************************************************

A0A5F5PJP7 IYKAVPSWFVRVEHMVDQLLRNNDLCYWVPEFVREKRFGNWLKDARDWAVSRNRYWGTPI

A0A5F5PJ23 IYKAVPSWFVRVEHMVDQLLRNNDLCYWVPEFVREKRFGNWLKDARDWAVSRNRYWGTPI

************************************************************

A0A5F5PJP7 PLWVSDDLEEVVCVGSMAELEELSGAKISDLHRESIDHLTIPSRCGKGSLHRISEVFDCW

A0A5F5PJ23 PLWVSDDLEEVVCVGSMAELEELSGAKISDLHRESIDHLTIPSRCGKGSLHRISEVFDCW

************************************************************

A0A5F5PJP7 FESGSMPYAQVHYPFESKREFEEAFPADFIAEGIDQTRGWFYTLLVLATALFGQPPFKNV

A0A5F5PJ23 FESGSMPYAQVHYPFESKREFEEAFPADFIAEGIDQTRGWFYTLLVLATALFGQPPFKNV

************************************************************

A0A5F5PJP7 IVNGLVLASDGQKMSKRKKNYPDPLSVIHKYGADALRLYLINSPVVRAENLRFKEEGVRD

A0A5F5PJ23 IVNGLVLASDGQKMSKRKKNYPDPLSVIHKYGADALRLYLINSPVVRAENLRFKEEGVRD

************************************************************

A0A5F5PJP7 VLKDVLLPWYNAYRFFIQNILRLQKEEEMEFLYNENTVKESANITDRWVLSFMQSLVEFF

A0A5F5PJ23 VLKDVLLPWYNAYRFFIQNILRLQKEEEMEFLYNENTVKESANITDRWVLSFMQSLVEFF

************************************************************

A0A5F5PJP7 KAEMAAYRLYTVVPRLVKFVDVLTNWYVRMNRRRLKGENGMEDCVLALETLFSVLLSLCR

A0A5F5PJ23 KAEMAAYRLYTVVPRLVKFVDVLTNWYVRMNRRRLKGENGMEDCVLALETLFSVLLSLCR

************************************************************

A0A5F5PJP7 LMAPYTPFLTEMMYQNLKMLIDPVSVQDKDTLSIHYLMLPHVREELIDKKTESAVSRMQS

A0A5F5PJ23 LMAPYTPFLTEMMYQNLKMLIDPVSVQDKDTLSIHYLMLPHVREELIDKKTESAVSRMQS

************************************************************

A0A5F5PJP7 VIELGRVIRDRKTIPIKYPLKEIVVIHQDPEALNDIKSLEKYIIEELNVRKVTLSTDKSK

A0A5F5PJ23 VIELGRVIRDRKTIPIKYPLKEIVVIHQDPEALNDIKSLEKYIIEELNVRKVTLSTDKSK

************************************************************

A0A5F5PJP7 YGIRLRAEPDHMVLGKRLKGAFKVVMTAIKQLSSEELEQFQRRGTIVVEGHELHEEDIRL

A0A5F5PJ23 YGIRLRAEPDHMVLGKRLKGAFKVVMTAIKQLSSEELEQFQRRGTIVVEGHELHEEDIRL

************************************************************

A0A5F5PJP7 MCTFDQAASGTAQFEAHSDAQALVLLDVTPDQSMVDEGMAREVINRIQKLRKKCNLVPTD

A0A5F5PJ23 MCTFDQAASGTAQFEAHSDAQALVLLDVTPDQSMVDEGMAREVINRIQKLRKKCNLVPTD

************************************************************

A0A5F5PJP7 EITVYYKAKSEGEYLNNVIESHTEFIFATIKAPLKPYPVPTADKILIQEKTQLKGSDLEI

A0A5F5PJ23 EITVYYKAKSEGEYLNNVIESHTEFIFATIKAPLKPYPVPTADKILIQEKTQLKGSDLEI

************************************************************

A0A5F5PJP7 TLTRGSSVPGPACAYVNLNICTNGSEQGGVLLLENPKGDNRLDLLKLKSVVTSIFGVKTT

A0A5F5PJ23 TLTRGSSVPGPACAYVNLNICTNGSEQGGVLLLENPKGDNRLDLLKLKSVVTSIFGVKTT

************************************************************

A0A5F5PJP7 RLAVFHGETEIQNQTDLLSLSGKTLCVTAGSAPSLIDSPSALLCQYVNLQLLDADPQECL

A0A5F5PJ23 RLAVFHGETEIQNQTDLLSLSGKTLCVTAGSAPSLIDSPSALLCQYVNLQLLDADPQECL

************************************************************

A0A5F5PJP7 MGTVGTLLLENPLGQSGLTHQGLLYEAAKVFGLRSRKLKLFLNETQTDEITEEIPMKTLN

A0A5F5PJ23 MGTVGTLLLENPLGQSGLTHQGLLYEAAKVFGLRSRKLKLFLNETQTDEITEEIPMKTLN

************************************************************

A0A5F5PJP7 MKTVYVSVLPTTADF

A0A5F5PJ23 MKTVYVSVLPTTADF

***************

CLUSTAL 2.1 Multiple Sequence Alignments

**Protein :** **Splicing factor 3b subunit 2**

Sequence type explicitly set to Protein

Sequence format is Pearson

Sequence 1: **A0A5F5PFU1** 879 aa

Sequence 2: **A0A9L0RE13** 897 aa

Start of Pairwise alignments

Aligning...

Sequences (1:2) Aligned. Score: 99.3174

Guide tree file created: [[clustalw.dnd]](https://www.genome.jp/tools-bin/pushfile?250213060626shEZz+clustalw.dnd)

There are 1 groups

Start of Multiple Alignment

Aligning...

Group 1: Sequences: 2 Score:14520

Alignment Score 5512

CLUSTAL-Alignment file created [[clustalw.aln]](https://www.genome.jp/tools-bin/pushfile?250213060626shEZz+clustalw.aln)

[clustalw.aln](https://www.genome.jp/tools-bin/pushfile?250213060626shEZz+clustalw.aln)

CLUSTAL 2.1 multiple sequence alignment

A0A5F5PFU1 MATEHPEPPKGELQLPPPPPPGHYGAWAAQELQAKLAEIGAPIQG-SREELVERLQTYTR

A0A9L0RE13 MATEHPEPPKGELQLPPPPPPGHYGAWAAQELQAKLAEIGAPIQAGSREELVERLQTYTR

********************************************. **************

A0A5F5PFU1 QTGIVLNRPVLRGEDGDKAAPPPMSAQLSGIPMPPPPMGLPPLQPPPPPPPPPPGLGLGF

A0A9L0RE13 QTGIVLNRPVLRGEDGDKAAPPPMSAQLSGIPMPPPPMGLPPLQPPPPPPPPPPGLGLGF

************************************************************

A0A5F5PFU1 PMAVGPRPPNLGPPPPLRVGEPVALSEEERLKLAQQQAALLMQQEERAKQ----------

A0A9L0RE13 PMAVGPRPPNLGPPPPLRVGEPVALSEEERLKLAQQQAALLMQQEERAKQQGDHSLKEHE

**************************************************

A0A5F5PFU1 -------AAVLLEQERQQQEIAKMGTPVPRPPQDLGQIGVRTPLGPRVAAPVGPTPTVLP

A0A9L0RE13 LLEQQKRAAVLLEQERQQQEIAKMGTPVPRPPQDLGQIGVRTPLGPRVAAPVGPTPTVLP

*****************************************************

A0A5F5PFU1 MGAPVPRPRGPPPPPGDENREMDDPSVGPKIPQALEKILQLKESRQEEMNSQQEEEEMET

A0A9L0RE13 MGAPVPRPRGPPPPPGDENREMDDPSVGPKIPQALEKILQLKESRQEEMNSQQEEEEMET

************************************************************

A0A5F5PFU1 DARSSLGQSASETEEDTVSVSKKEKNRKRRNRKKKKKPQRVRGASSESSGDREKESARPR

A0A9L0RE13 DARSSLGQSASETEEDTVSVSKKEKNRKRRNRKKKKKPQRVRGASSESSGDREKESARPR

************************************************************

A0A5F5PFU1 GSDSPAADVEIEYVTEEPEIYEPNFIFFKRIFEAFKLTDDVKKEKEKEPEKLDKLENSAA

A0A9L0RE13 GSDSPAADVEIEYVTEEPEIYEPNFIFFKRIFEAFKLTDDVKKEKEKEPEKLDKLENSAA

************************************************************

A0A5F5PFU1 PKKKGFEEEHKDSDDDSSDDEQEKKPEAPKLSKKKLRRMNRFTVAELKQLVARPDVVEMH

A0A9L0RE13 PKKKGFEEEHKDSDDDSSDDEQEKKPEAPKLSKKKLRRMNRFTVAELKQLVARPDVVEMH

************************************************************

A0A5F5PFU1 DVTAQDPKLLVHLKATRNSVPVPRHWCFKRKYLQGKRGIEKPPFELPDFIKRTGIQEMRE

A0A9L0RE13 DVTAQDPKLLVHLKATRNSVPVPRHWCFKRKYLQGKRGIEKPPFELPDFIKRTGIQEMRE

************************************************************

A0A5F5PFU1 ALQEKEEQKTMKSKMREKVRPKMGKIDIDYQKLHDAFFKWQTKPKLTIHGDLYYEGKEFE

A0A9L0RE13 ALQEKEEQKTMKSKMREKVRPKMGKIDIDYQKLHDAFFKWQTKPKLTIHGDLYYEGKEFE

************************************************************

A0A5F5PFU1 TRLKEKKPGDLSDELRISLGMPVGPNAHKVPPPWLIAMQRYGPPPSYPNLKIPGLNSPIP

A0A9L0RE13 TRLKEKKPGDLSDELRISLGMPVGPNAHKVPPPWLIAMQRYGPPPSYPNLKIPGLNSPIP

************************************************************

A0A5F5PFU1 ESCSFGYHAGGWGKPPVDETGKPLYGDVFGTNAAEFQTKTEEEEIDRTPWGELEPSDEES

A0A9L0RE13 ESCSFGYHAGGWGKPPVDETGKPLYGDVFGTNAAEFQTKTEEEEIDRTPWGELEPSDEES

************************************************************

A0A5F5PFU1 SEEEEEEESDEDKPDETGFITPADSGLITPGGFSSVPAGMETPELIELRKKKIEEAMDGS

A0A9L0RE13 SEEEEEEESDEDKPDETGFITPADSGLITPGGFSSVPAGMETPELIELRKKKIEEAMDGS

************************************************************

A0A5F5PFU1 ETPQLFTVLPEKRTATVGGAMMGSTHIYDMSTVMSRKGPAPELQGVEVALAPEELELDPM

A0A9L0RE13 ETPQLFTVLPEKRTATVGGAMMGSTHIYDMSTVMSRKGPAPELQGVEVALAPEELELDPM

************************************************************

A0A5F5PFU1 AMTQKYEEHVREQQAQVEKEDFSDMVAEHAAKQKQKKRKAQPQDSRGGSKKYKEFKF

A0A9L0RE13 AMTQKYEEHVREQQAQVEKEDFSDMVAEHAAKQKQKKRKAQPQDSRGGSKKYKEFKF

*********************************************************

CLUSTAL 2.1 Multiple Sequence Alignments

**Protein: Spectrin alpha chain, non-erythrocytic 1**

Sequence type explicitly set to Protein

Sequence format is Pearson

Sequence 1: **F6UPN4**  2464 aa

Sequence 2: **F6UPM7**  2463 aa

Sequence 3: **A0A3Q2GZL9** 2489 aa

Start of Pairwise alignments

Aligning...

Sequences (1:2) Aligned. Score: 95.2497

Sequences (1:3) Aligned. Score: 99.7565

Sequences (2:3) Aligned. Score: 99.7564

Guide tree file created: [[clustalw.dnd]](https://www.genome.jp/tools-bin/pushfile?250213060934kiqwa+clustalw.dnd)

There are 2 groups

Start of Multiple Alignment

Aligning...

Group 1: Sequences: 2 Score:40081

Group 2: Sequences: 3 Score:39674

Alignment Score 44551

CLUSTAL-Alignment file created [[clustalw.aln]](https://www.genome.jp/tools-bin/pushfile?250213060934kiqwa+clustalw.aln)

[clustalw.aln](https://www.genome.jp/tools-bin/pushfile?250213060934kiqwa+clustalw.aln)

CLUSTAL 2.1 multiple sequence alignment

F6UPM7 MLSSFRKRRVQKMDPSGVKVLETAEDIQERRQQVLDRYHRFKELSTLRRQKLEDSYRFQF

A0A3Q2GZL9 MLSSFRKRRVQKMDPSGVKVLETAEDIQERRQQVLDRYHRFKELSTLRRQKLEDSYRFQF

F6UPN4 ------------MDPSGVKVLETAEDIQERRQQVLDRYHRFKELSTLRRQKLEDSYRFQF

************************************************

F6UPM7 FQRDAEELEKWIQEKLQIASDENYKDPTNLQGKLQKHQAFEAEVQANSGAIVKLDETGNL

A0A3Q2GZL9 FQRDAEELEKWIQEKLQIASDENYKDPTNLQGKLQKHQAFEAEVQANSGAIVKLDETGNL

F6UPN4 FQRDAEELEKWIQEKLQIASDENYKDPTNLQGKLQKHQAFEAEVQANSGAIVKLDETGNL

************************************************************

F6UPM7 MISEGHFASETIRTRLMELHRQWELLLEKMREKGVKLLQAQKLVQYLRECEDVMDWINDK

A0A3Q2GZL9 MISEGHFASETIRTRLMELHRQWELLLEKMREKGVKLLQAQKLVQYLRECEDVMDWINDK

F6UPN4 MISEGHFASETIRTRLMELHRQWELLLEKMREKGVKLLQAQKLVQYLRECEDVMDWINDK

************************************************************

F6UPM7 EAIVTSEELGQDLEHVEVLQKKFEEFQTDMAAHEERVNEVNQFAAKLIQEQHPEEELIKT

A0A3Q2GZL9 EAIVTSEELGQDLEHVEVLQKKFEEFQTDMAAHEERVNEVNQFAAKLIQEQHPEEELIKT

F6UPN4 EAIVTSEELGQDLEHVEVLQKKFEEFQTDMAAHEERVNEVNQFAAKLIQEQHPEEELIKT

************************************************************

F6UPM7 KQDEVNAAWQRLKGLALQRQGKLFGAAEVQRFNRDVDETISWIKEKEQLMASDDFGRDLA

A0A3Q2GZL9 KQDEVNAAWQRLKGLALQRQGKLFGAAEVQRFNRDVDETISWIKEKEQLMASDDFGRDLA

F6UPN4 KQDEVNAAWQRLKGLALQRQGKLFGAAEVQRFNRDVDETISWIKEKEQLMASDDFGRDLA

************************************************************

F6UPM7 SVQALLRKHEGLERDLAALEDKVKALCAEADRLQQSHPLSATQIQVKREELITNWEQIRT

A0A3Q2GZL9 SVQALLRKHEGLERDLAALEDKVKALCAEADRLQQSHPLSATQIQVKREELITNWEQIRT

F6UPN4 SVQALLRKHEGLERDLAALEDKVKALCAEADRLQQSHPLSATQIQVKREELITNWEQIRT

************************************************************

F6UPM7 LAAERHARLNDSYRLQRFLADFRDLTSWVTEMKALINADELANDVAGAEALLDRHQEHKG

A0A3Q2GZL9 LAAERHARLNDSYRLQRFLADFRDLTSWVTEMKALINADELANDVAGAEALLDRHQEHKG

F6UPN4 LAAERHARLNDSYRLQRFLADFRDLTSWVTEMKALINADELANDVAGAEALLDRHQEHKG

************************************************************

F6UPM7 EIDAHEDSFKSADESGQALLAAGHYASDEVKEKLTILSEERAALLELWELRRQQYEQCMD

A0A3Q2GZL9 EIDAHEDSFKSADESGQALLAAGHYASDEVKEKLTILSEERAALLELWELRRQQYEQCMD

F6UPN4 EIDAHEDSFKSADESGQALLAAGHYASDEVKEKLTILSEERAALLELWELRRQQYEQCMD

************************************************************

F6UPM7 LQLFYRDTEQVDNWMSKQEAFLLNEDLGDSLDSVEALLKKHEDFEKSLSAQEEKITALDE

A0A3Q2GZL9 LQLFYRDTEQVDNWMSKQEAFLLNEDLGDSLDSVEALLKKHEDFEKSLSAQEEKITALDE

F6UPN4 LQLFYRDTEQVDNWMSKQEAFLLNEDLGDSLDSVEALLKKHEDFEKSLSAQEEKITALDE

************************************************************

F6UPM7 FATKLIQNNHYAMEDVATRRDALLSRRNALHERAMYRRAQLADSFHLQQFFRDSDELKSW

A0A3Q2GZL9 FATKLIQNNHYAMEDVATRRDALLSRRNALHERAMYRRAQLADSFHLQQFFRDSDELKSW

F6UPN4 FATKLIQNNHYAMEDVATRRDALLSRRNALHERAMYRRAQLADSFHLQQFFRDSDELKSW

************************************************************

F6UPM7 VNEKMKTATDEAYKDPSNLQGKVQKHQAFEAELSANQSRIDALEKAGQKLIDVNHYAKDE

A0A3Q2GZL9 VNEKMKTATDEAYKDPSNLQGKVQKHQAFEAELSANQSRIDALEKAGQKLIDVNHYAKDE

F6UPN4 VNEKMKTATDEAYKDPSNLQGKVQKHQAFEAELSANQSRIDALEKAGQKLIDVNHYAKDE

************************************************************

F6UPM7 VAARMNEVISLWKKLLEATELKGIKLREANQQQQFNRNVEDIELWLYEVEGHLASDDYGK

A0A3Q2GZL9 VAARMNEVISLWKKLLEATELKGIKLREANQQQQFNRNVEDIELWLYEVEGHLASDDYGK

F6UPN4 VAARMNEVISLWKKLLEATELKGIKLREANQQQQFNRNVEDIELWLYEVEGHLASDDYGK

************************************************************

F6UPM7 DLTNVQNLQKKHALLEADVAAHQDRIDGITIQARQFQDAGHFDAENIKKKQEALVARYEA

A0A3Q2GZL9 DLTNVQNLQKKHALLEADVAAHQDRIDGITIQARQFQDAGHFDAENIKKKQEALVARYEA

F6UPN4 DLTNVQNLQKKHALLEADVAAHQDRIDGITIQARQFQDAGHFDAENIKKKQEALVARYEA

************************************************************

F6UPM7 LKEPMIARKQKLADSLRLQQLFRDVEDEETWIREKEPIAASTNRGKDLIGVQNLLKKHQA

A0A3Q2GZL9 LKEPMIARKQKLADSLRLQQLFRDVEDEETWIREKEPIAASTNRGKDLIGVQNLLKKHQA

F6UPN4 LKEPMIARKQKLADSLRLQQLFRDVEDEETWIREKEPIAASTNRGKDLIGVQNLLKKHQA

************************************************************

F6UPM7 LQAEIAGHEPRIKAVTQKGHAMVEEGHFAAEDVKAKLNELNQKWESLKSKASQRRQDLED

A0A3Q2GZL9 LQAEIAGHEPRIKAVTQKGHAMVEEGHFAAEDVKAKLNELNQKWESLKSKASQRRQDLED

F6UPN4 LQAEIAGHEPRIKAVTQKGHAMVEEGHFAAEDVKAKLNELNQKWESLKSKASQRRQDLED

************************************************************

F6UPM7 SLQAQQYFADANEAESWMREKEPIVGSTDYGKDEDSAEALLKKHEALMSDLSAYGSSIQA

A0A3Q2GZL9 SLQAQQYFADANEAESWMREKEPIVGSTDYGKDEDSAEALLKKHEALMSDLSAYGSSIQA

F6UPN4 SLQAQQYFADANEAESWMREKEPIVGSTDYGKDEDSAEALLKKHEALMSDLSAYGSSIQA

************************************************************

F6UPM7 LREQAQSCRQQVAPMDDETGKELVLALYDYQEKSPREVTMKKGDILTLLNSTNKDWWKVE

A0A3Q2GZL9 LREQAQSCRQQVAPMDDETGKELVLALYDYQEKSPREVTMKKGDILTLLNSTNKDWWKVE

F6UPN4 LREQAQSCRQQVAPMDDETGKELVLALYDYQEKSPREVTMKKGDILTLLNSTNKDWWKVE

************************************************************

F6UPM7 VNDRQGFVPAAYVKKLDPAQSASRENLLEEQGSIALRQE--------------------Q

A0A3Q2GZL9 VNDRQGFVPAAYVKKLDPAQSASRENLLEEQGSIALRQEQIDNQTRITKEAGSVSLRMKQ

F6UPN4 VNDRQGFVPAAYVKKLDPAQSASRENLLEEQGSIALRQEQIDNQTRITKEAGSVSLRMKQ

*************************************** *

F6UPM7 IDNQYRSLLELGEKRKGMLEKSCKKFMLFREANELQQWINEKEAALTSEEVGADLEQVEV

A0A3Q2GZL9 VEELYRSLLELGEKRKGMLEKSCKKFMLFREANELQQWINEKEAALTSEEVGADLEQVEV

F6UPN4 VEELYRSLLELGEKRKGMLEKSCKKFMLFREANELQQWINEKEAALTSEEVGADLEQVEV

::: ********************************************************

F6UPM7 LQKKFDDFQKDLKANESRLKDINKVAEALESEGLMAEEVQAVQQQEVYGAMPRDEGDSKT

A0A3Q2GZL9 LQKKFDDFQKDLKANESRLKDINKVAEALESEGLMAEEVQAVQQQEVYGAMPRDEGDSKT

F6UPN4 LQKKFDDFQKDLKANESRLKDINKVAEALESEGLMAEEVQAVQQQ--------DEGDSKT

********************************************* *******

F6UPM7 ASPWKSARLMVHTVATFNSIKELNERWRSLQQLAEERSQLLGSAHEVQRFHRDADETKEW

A0A3Q2GZL9 ASPWKSARLMVHTVATFNSIKELNERWRSLQQLAEERSQLLGSAHEVQRFHRDADETKEW

F6UPN4 ASPWKSARLMVHTVATFNSIKELNERWRSLQQLAEERSQLLGSAHEVQRFHRDADETKEW

************************************************************

F6UPM7 IEEKNQALNTDNYGHDLASVQALQRKHEGFERDLAALGDKVNSLGETAERLIQSHPESAE

A0A3Q2GZL9 IEEKNQALNTDNYGHDLASVQALQRKHEGFERDLAALGDKVNSLGETAERLIQSHPESAE

F6UPN4 IEEKNQALNTDNYGHDLASVQALQRKHEGFERDLAALGDKVNSLGETAERLIQSHPESAE

************************************************************

F6UPM7 DLQEKCTELNQAWSSLGKRADQRKAKLGDSHDLQRFLSDFRDLMSWINGIRGLVSSDELA

A0A3Q2GZL9 DLQEKCTELNQAWSSLGKRADQRKAKLGDSHDLQRFLSDFRDLMSWINGIRGLVSSDELA

F6UPN4 DLQEKCTELNQAWSSLGKRADQRKAKLGDSHDLQRFLSDFRDLMSWINGIRGLVSSDELA

************************************************************

F6UPM7 KDVTGAEALLERHQEHRTEIDARAGTFQAFEQFGQQLLAHRHYASPEIKEKLDILDQERA

A0A3Q2GZL9 KDVTGAEALLERHQEHRTEIDARAGTFQAFEQFGQQLLAHRHYASPEIKEKLDILDQERA

F6UPN4 KDVTGAEALLERHQEHRTEIDARAGTFQAFEQFGQQLLAHRHYASPEIKEKLDILDQERA

************************************************************

F6UPM7 DLEKAWVQRRMMLDQCLELQLFHRDCEQAENWMAAREAFLNTEDKGDSLDSVEALIKKHE

A0A3Q2GZL9 DLEKAWVQRRMMLDQCLELQLFHRDCEQAENWMAAREAFLNTEDKGDSLDSVEALIKKHE

F6UPN4 DLEKAWVQRRMMLDQCLELQLFHRDCEQAENWMAAREAFLNTEDKGDSLDSVEALIKKHE

************************************************************

F6UPM7 DFDKAINVQEEKIAALQSFADQLIAAGHYAKGDISSRRNEVLDRWRRLKAQMIEKRSKLG

A0A3Q2GZL9 DFDKAINVQEEKIAALQSFADQLIAAGHYAKGDISSRRNEVLDRWRRLKAQMIEKRSKLG

F6UPN4 DFDKAINVQEEKIAALQSFADQLIAAGHYAKGDISSRRNEVLDRWRRLKAQMIEKRSKLG

************************************************************

F6UPM7 ESQTLQQFSRDVDEIEAWISEKLQTASDESYKDPTNIQLSKLLSKHQKHQAFEAELHANA

A0A3Q2GZL9 ESQTLQQFSRDVDEIEAWISEKLQTASDESYKDPTNIQLSKLLSKHQKHQAFEAELHANA

F6UPN4 ESQTLQQFSRDVDEIEAWISEKLQTASDESYKDPTNIQS-----KHQKHQAFEAELHANA

************************************** ****************

F6UPM7 DRIRGVIDMGNSLIERGACAGSEDAVKARLAALADQWQFLVQKSAEKSQKLKEANKQQNF

A0A3Q2GZL9 DRIRGVIDMGNSLIERGACAGSEDAVKARLAALADQWQFLVQKSAEKSQKLKEANKQQNF

F6UPN4 DRIRGVIDMGNSLIERGACAGSEDAVKARLAALADQWQFLVQKSAEKSQKLKEANKQQNF

************************************************************

F6UPM7 NTGIKDFDFWLSEVEALLASEDYGKDLASVNNLLKKHQLLEADISAHEDRLKDLNSQADS

A0A3Q2GZL9 NTGIKDFDFWLSEVEALLASEDYGKDLASVNNLLKKHQLLEADISAHEDRLKDLNSQADS

F6UPN4 NTGIKDFDFWLSEVEALLASEDYGKDLASVNNLLKKHQLLEADISAHEDRLKDLNSQADS

************************************************************

F6UPM7 LMTSSAFDTSQVKDKRDTINGRFQKIKSMAASRRAKLNESHRLHQFFRDMDDEESWIKEK

A0A3Q2GZL9 LMTSSAFDTSQVKDKRDTINGRFQKIKSMAASRRAKLNESHRLHQFFRDMDDEESWIKEK

F6UPN4 LMTSSAFDTSQVKDKRDTINGRFQKIKSMAASRRAKLNESHRLHQFFRDMDDEESWIKEK

************************************************************

F6UPM7 KLLVSSEDYGRDLTGVQNLRKKHKRLEAELAAHEPAIQGVLDTGKKLSDDNTIGKEEIQQ

A0A3Q2GZL9 KLLVSSEDYGRDLTGVQNLRKKHKRLEAELAAHEPAIQGVLDTGKKLSDDNTIGKEEIQQ

F6UPN4 KLLVSSEDYGRDLTGVQNLRKKHKRLEAELAAHEPAIQGVLDTGKKLSDDNTIGKEEIQQ

************************************************************

F6UPM7 RLAQFVEHWKELKQLAAARGQRLEESLEYQQFVANVEEEEAWINEKMTLVASEDYGDTLA

A0A3Q2GZL9 RLAQFVEHWKELKQLAAARGQRLEESLEYQQFVANVEEEEAWINEKMTLVASEDYGDTLA

F6UPN4 RLAQFVEHWKELKQLAAARGQRLEESLEYQQFVANVEEEEAWINEKMTLVASEDYGDTLA

************************************************************

F6UPM7 AIQGLLKKHEAFETDFTVHKDRVNDVCTNGQDLVKKNNHHEENISSKMKCLNGKVSDLEK

A0A3Q2GZL9 AIQGLLKKHEAFETDFTVHKDRVNDVCTNGQDLVKKNNHHEENISSKMKCLNGKVSDLEK

F6UPN4 AIQGLLKKHEAFETDFTVHKDRVNDVCTNGQDLVKKNNHHEENISSKMKCLNGKVSDLEK

************************************************************

F6UPM7 AASQRKAKLDENSAFLQFNWKADVVESWIGEKENSLKTDDYGRDLSSVQTLLTKQETFDA

A0A3Q2GZL9 AASQRKAKLDENSAFLQFNWKADVVESWIGEKENSLKTDDYGRDLSSVQTLLTKQETFDA

F6UPN4 AASQRKAKLDENSAFLQFNWKADVVESWIGEKENSLKTDDYGRDLSSVQTLLTKQETFDA

************************************************************

F6UPM7 GLQAFQQEGIANITALKDQLLAAKHIQSKAIEARHASLMKRWSQLLANSATRKKKLLEAQ

A0A3Q2GZL9 GLQAFQQEGIANITALKDQLLAAKHIQSKAIEARHASLMKRWSQLLANSATRKKKLLEAQ

F6UPN4 GLQAFQQEGIANITALKDQLLAAKHIQSKAIEARHASLMKRWSQLLANSATRKKKLLEAQ

************************************************************

F6UPM7 SHFRKVEDLFLTFAKKASAFNSWFENAEEDLTDPVRCNSLEEIKALREAHDAFRSSLSSA

A0A3Q2GZL9 SHFRKVEDLFLTFAKKASAFNSWFENAEEDLTDPVRCNSLEEIKALREAHDAFRSSLSSA

F6UPN4 SHFRKVEDLFLTFAKKASAFNSWFENAEEDLTDPVRCNSLEEIKALREAHDAFRSSLSSA

************************************************************

F6UPM7 QADFNQLAELDRQIKSFRVASNPYTWFTMEALEETWRNLQKIIKERELELQKEQRRQEEN

A0A3Q2GZL9 QADFNQLAELDRQIKSFRVASNPYTWFTMEALEETWRNLQKIIKERELELQKEQRRQEEN

F6UPN4 QADFNQLAELDRQIKSFRVASNPYTWFTMEALEETWRNLQKIIKERELELQKEQRRQEEN

************************************************************

F6UPM7 DKLRQEFAQHANAFHQWIQETR------SCMVEESGTLESQLEATKRKHQEIRAMRSQLK

A0A3Q2GZL9 DKLRQEFAQHANAFHQWIQETRTYLLDGSCMVEESGTLESQLEATKRKHQEIRAMRSQLK

F6UPN4 DKLRQEFAQHANAFHQWIQETRTYLLDGSCMVEESGTLESQLEATKRKHQEIRAMRSQLK

********************** ********************************

F6UPM7 KIEDLGAAMEEALILDNKYTEHSTVGLAQQWDQLDQLGMRMQHNLEQQIQARNTTGVTEE

A0A3Q2GZL9 KIEDLGAAMEEALILDNKYTEHSTVGLAQQWDQLDQLGMRMQHNLEQQIQARNTTGVTEE

F6UPN4 KIEDLGAAMEEALILDNKYTEHSTVGLAQQWDQLDQLGMRMQHNLEQQIQARNTTGVTEE

************************************************************

F6UPM7 ALKEFSMMFKHFDKDKSGRLNHQEFKSCLRSLGYDLPMVEEGEPDPEFEAILDTVDPNRD

A0A3Q2GZL9 ALKEFSMMFKHFDKDKSGRLNHQEFKSCLRSLGYDLPMVEEGEPDPEFEAILDTVDPNRD

F6UPN4 ALKEFSMMFKHFDKDKSGRLNHQEFKSCLRSLGYDLPMVEEGEPDPEFEAILDTVDPNRD

************************************************************

F6UPM7 GHVSLQEYMAFMISRETENVKSSEEIESAFRALSSEGKPYVTKEELYQNLTREQADYCVS

A0A3Q2GZL9 GHVSLQEYMAFMISRETENVKSSEEIESAFRALSSEGKPYVTKEELYQNLTREQADYCVS

F6UPN4 GHVSLQEYMAFMISRETENVKSSEEIESAFRALSSEGKPYVTKEELYQNLTREQADYCVS

************************************************************

F6UPM7 HMKPYVDSKGRELPTAFDYVEFTRSLFVN

A0A3Q2GZL9 HMKPYVDSKGRELPTAFDYVEFTRSLFVN

F6UPN4 HMKPYVDSKGRELPTAFDYVEFTRSLFVN

*****************************

CLUSTAL 2.1 Multiple Sequence Alignments

**Protein:** **Signal transducer and activator of transcription**

Sequence type explicitly set to Protein

Sequence format is Pearson

Sequence 1**: A0A9L0SJ42** 702 aa

Sequence 2: **A0A3Q2L9H8**  694 aa

Start of Pairwise alignments

Aligning...

Sequences (1:2) Aligned. Score: 89.6254

Guide tree file created: [[clustalw.dnd]](https://www.genome.jp/tools-bin/pushfile?250213061253R4ENn+clustalw.dnd)

There are 1 groups

Start of Multiple Alignment

Aligning...

Group 1: Sequences: 2 Score:10242

Alignment Score 3872

CLUSTAL-Alignment file created [[clustalw.aln]](https://www.genome.jp/tools-bin/pushfile?250213061253R4ENn+clustalw.aln)

[clustalw.aln](https://www.genome.jp/tools-bin/pushfile?250213061253R4ENn+clustalw.aln)

CLUSTAL 2.1 multiple sequence alignment

A0A9L0SJ42 MAQWNQLQQLDTRYLEQLHQLYSDSFPMELRQFLAPWIESQDWAYAASKESHATLVFHNL

A0A3Q2L9H8 MAQWNQLQQLDTRYLEQLHQLYSDSFPMELRQFLAPWIESQDWAYAASKESHATLVFHNL

************************************************************

A0A9L0SJ42 LGEIDQQYSRFLQESNVLYQHNLRRIKQFLQSRYLEKPMEIARIVARCLWEESRLLQTAA

A0A3Q2L9H8 LGEIDQQYSRFLQESNVLYQHNLRRIKQFLQSRYLEKPMEIARIVARCLWEESRLLQTAA

************************************************************

A0A9L0SJ42 TAAQQGGQANHPTAAVVTEKQQMLEQHLQDVRKRVQDLEQKMKVVENLQDDFDFNYKTLK

A0A3Q2L9H8 TAAQQGGQANHPTAAVVTEKQQMLEQHLQDVRKRVQDLEQKMKVVENLQDDFDFNYKTLK

************************************************************

A0A9L0SJ42 SQGDMQDLNGNNQSVTRQKMQQLEQMLTALDQMRRSIVSELAGLLSAMEYVQKTLTDEEL

A0A3Q2L9H8 SQGDMQDLNGNNQSVTRQKMQQLEQMLTALDQMRRSIVSELAGLLSAMEYVQKTLTDEEL

************************************************************

A0A9L0SJ42 ADWKRRQQIACIGGPPNICLDRLENWITSLAESQLQTRQQIKKLEELQQKVSYKGDPIVQ

A0A3Q2L9H8 ADWKRRQQIACIGGPPNICLDRLENWITSLAESQLQTRQQIKKLEELQQKVSYKGDPIVQ

************************************************************

A0A9L0SJ42 HRPMLEERIVELFRNLMKSAFVVERQPCMPMHPDRPLVIKTGVQFTTKVRLLVKFPELNY

A0A3Q2L9H8 HRPMLEERIVELFRNLMKSAFVVERQPCMPMHPDRPLVIKTGVQFTTKVRLLAS------

****************************************************..

A0A9L0SJ42 QLKIKVCIDKDSGDVAALRGSRKFNILGTNTKVMNMEESNNGSLSAEFKHLTLREQRCGN

A0A3Q2L9H8 ------------------------------------------------------------

A0A9L0SJ42 GGRANCDASLIVTEELHLITFETEVYHQGLKIDLETHSLPVVVISNICQMPNAWASILWY

A0A3Q2L9H8 ---------LIVTEELHLITFETEVYHQGLKIDLETHSLPVVVISNICQMPNAWASILWY

***************************************************

A0A9L0SJ42 NMLTNNPKNVNFFTKPPIGTWDQVAEVLSWQFSSTTKRGLSIEQLTTLAEKLLGPGVNYS

A0A3Q2L9H8 NMLTNNPKNVNFFTKPPIGTWDQVAEVLSWQFSSTTKRGLSIEQLTTLAEKLLGPGVNYS

************************************************************

A0A9L0SJ42 GCQITWAKFCKENMAGKGFSFWVWLDNIIDLVKKYILALWNEGYIMGFISKERERAILST

A0A3Q2L9H8 GCQITWAKFCKENMAGKGFSFWVWLDNIIDLVKKYILALWNEGYIMGFISKERERAILST

************************************************************

A0A9L0SJ42 KPPGTFLLRFSESSKEGGVTFTWVEKDISGKTQIQSVEPYTKQQLNNMSFAEIIMGYKIM

A0A3Q2L9H8 KPPGTFLLRFSESSKEGGVTFTWVEKDISGKTQIQSVEPYTKQQLNNMSFAEIIMGYKIM

************************************************************

A0A9L0SJ42 DATNILVSPLVYLYPDIPKEEAFGKYCRPESQEHPEADPGSC------------------

A0A3Q2L9H8 DATNILVSPLVYLYPDIPKEEAFGKYCRPESQEHPEADPGAAPYLKTKFICVTPTTCSNT

****************************************:.

A0A9L0SJ42 -------------------------------------------------

A0A3Q2L9H8 IDLPMSPRTLDSLMQFGNNGEGAEPSAGGQFESLTFDMELTSECATSPM

CLUSTAL 2.1 Multiple Sequence Alignments

**Protein:** **Versican core protein**

Sequence type explicitly set to Protein

Sequence format is Pearson

Sequence 1: **A0A5F5PVK6** 3400 aa

Sequence 2: **F7DY34**  2260 aa

Start of Pairwise alignments

Aligning...

Sequences (1:2) Aligned. Score: 99.5575

Guide tree file created: [[clustalw.dnd]](https://www.genome.jp/tools-bin/pushfile?250213061605by2LD+clustalw.dnd)

There are 1 groups

Start of Multiple Alignment

Aligning...

Group 1: Sequences: 2 Score:37170

Alignment Score 13787

CLUSTAL-Alignment file created [[clustalw.aln]](https://www.genome.jp/tools-bin/pushfile?250213061605by2LD+clustalw.aln)

[clustalw.aln](https://www.genome.jp/tools-bin/pushfile?250213061605by2LD+clustalw.aln)

CLUSTAL 2.1 multiple sequence alignment

A0A5F5PVK6 MLINIKSILWMCSTLIATHALHKVKVEKSPPVKGSLSGKVNLPCHFSTLPTLPPSYNTTS

F7DY34 ------------------------------------------------------------

A0A5F5PVK6 EFLRIKWSKIELDKNGKDLKETTVLVAQNGNIKIGQGYKGRVAVPTHPEDVGDASLTVVK

F7DY34 ------------------------------------------------------------

A0A5F5PVK6 LRASDAGRYRCDVMYGIEDTQDTVSLAVDGVVFHYRAATSRYTLNFESAQKACLDIGAVI

F7DY34 -----------------------MNTSKHGVVFHYRAATSRYTLNFESAQKACLDIGAVI

:. : .*******************************

A0A5F5PVK6 ATPEQLHAAYEDGFEQCDAGWLSDQTVRYPIRTPREGCYGDMMGKEGVRTYGFRSPHETY

F7DY34 ATPEQLHAAYEDGFEQCDAGWLSDQTVRYPIRTPREGCYGDMMGKEGVRTYGFRSPHETY

************************************************************

A0A5F5PVK6 DVYCYVDHLDGDVFHITAPKKFTFEEAEEECENRDARLATVGELQAAWRNGFDQCDYGWL

F7DY34 DVYCYVDHLDGDVFHITAPKKFTFEEAEEECENRDARLATVGELQAAWRNGFDQCDYGWL

************************************************************

A0A5F5PVK6 SDASVRHPVTVARAQCGGGLLGVRTLYRFENQTGFPPPDSRFDAYCFKPKQNISEATTIE

F7DY34 SDASVRHPVTVARAQCGGGLLGVRTLYRFENQTGFPPPDSRFDAYCFKR-----------

************************************************

A0A5F5PVK6 LNILAETASPSLSEELQMVPDRTTPIVPLITELPVITTKFPPVGNIVSFEQKATVQSQAV

F7DY34 ------------------------------------------------------------

A0A5F5PVK6 THRLVTESPTPAGSTKKPGDMHYYSPSASGPLGKPDITEIKEEVPQSTTVISHRATDSWD

F7DY34 ------------------------------------------------------------

A0A5F5PVK6 GVMEDTQTQESVTQIEQIEVGPLVTSMETSKHLPSKEFSVTETPFVSATMTLESKTEKKT

F7DY34 ------------------------------------------------------------

A0A5F5PVK6 VSVISESVTPSHYGFTLGKADGEDRTFTVRSGQSTLVFSQIPEVITVSKTSEDITPTQPE

F7DY34 ------------------------------------------------------------

A0A5F5PVK6 DAESVSASTLVSPVTIPDIDGSSMDVWEEKQTNGRMTKDFFGQNMSTTPFPSEHHTEVEF

F7DY34 ------------------------------------------------------------

A0A5F5PVK6 FPYSGDKRLVEGMSTVIYPSPQIEMTQEGERRETLRPEMRTDTYTADEIQERITKDPFIG

F7DY34 ------------------------------------------------------------

A0A5F5PVK6 KIEEEDFSGMKFSTASSEQIHLTESSVEMTKSFDSPALITTKLSVAPTEARDVKEDFTTT

F7DY34 ------------------------------------------------------------

A0A5F5PVK6 PVGLETDGYQDTTTYDEGITTVHLTHSTLDVEVVTVSKWSWDEDNTTSKPFGSTEHAGSP

F7DY34 ------------------------------------------------------------

A0A5F5PVK6 KLPPALFPTMGVSGKDEDIPSFTEDGGDEFTLIPGSTQKPFKEFPEEDTTDHGKFSVRFQ

F7DY34 ------------------------------------------------------------

A0A5F5PVK6 PTTSTGIAEKSTLRDSTTEERVSPITSTEGLVVYATVEGSALDEGEDVDVSKPVSTVPQF

F7DY34 ------------------------------------------------------------

A0A5F5PVK6 VHTSDVEGLAFVNYSSTQEPTTYVDTSHTVSLSLIPKTEWGVLVPSVPSEGEVLGEPSED

F7DY34 ------------------------------------------------------------

A0A5F5PVK6 IHVLDQTPLEATISPETIRTTTEITQGTTQEEFPWREQTSEKPIPALSSTVGTAKEATTS

F7DY34 ------------------------------------------------------------

A0A5F5PVK6 LDEQESDGSAYTVSEDRLVTSSERVPVLETTPVGKIEYSMSYPPGAVTEHKAKTDEVVTP

F7DY34 ------------------------------------------------------------

A0A5F5PVK6 SMGPKVSLRPGLDQKYETEGTSPGEIVSPFSTAVTQFIEETTTEKREETSLDYIDLGSGL

F7DY34 ------------------------------------------------------------

A0A5F5PVK6 FEKPKATELPEFSTINATVPSDIIAAFSSVDRLHTTSASTEKPPLIDREPGEETTSDMVI

F7DY34 ------------------------------------------------------------

A0A5F5PVK6 IGESTSRVPPTTLEDVVVKETETDIDREYFTTSSTPSATQPTRPPTVEGQEAFRPQALST

F7DY34 ------------------------------------------------------------

A0A5F5PVK6 PEPSAGTKFRPDINVYIIEVRENKTGRMSDLSVIGHPIDSESKEDEPCSEETDPEHDLIA

F7DY34 --------------------------RMSDLSVIGHPIDSESKEDEPCSEETDPEHDLIA

**********************************

A0A5F5PVK6 EILPELIEIDIYHSEEDEAEDEECANATDVTTTPSVQYINGKHLVTTVPKDPEAAEARRG

F7DY34 EILPELIEIDIYHSEEDEAEDEECANATDVTTTPSVQYINGKHLVTTVPKDPEAAEARRG

************************************************************

A0A5F5PVK6 QFESVAPSQNFSDSSESGTHQFVIAITGLSTAMQPNESKETTESLEITWKPETYPETPEH

F7DY34 QFESVAPSQNFSDSSESGTHQFVIAITGLSTAMQPNESKETTESLEITWKPETYPETPEH

************************************************************

A0A5F5PVK6 SSSGEPDVFPTVSFHEGEATEGPESITERGPELDTLVHGHTEPVPLFSEESSGDATIDQE

F7DY34 SSSGEPDVFPTVSFHEGEATEGPESITERGPELDTLVHGHTEPVPLFSEESSGDATIDQE

************************************************************

A0A5F5PVK6 SQKMIFSGATEVTFDEEAEKRTSVTYTPSAVPSSVSASVSEEVSVTLTEKSWPDDSLSTV

F7DY34 SQKMIFSGATEVTFDEEAEKRTSVTYTPSAVPSSVSASVSEEVSVTLTEKSWPDDSLSTV

************************************************************

A0A5F5PVK6 ESWVEITPRPIVELSGSPSIPIPEGSGEAEEDKDKMFTAVTNLPQRSTTDTLVTLDASKI

F7DY34 ESWVEITPRPIVELSGSPSIPIPEGSGEAEEDKDKMFTAVTNLPQRSTTDTLVTLDASKI

************************************************************

A0A5F5PVK6 MITEGLFDISATTVYSVSEQPFAEVVPTKFVRETDTSEWVFSTSLEGKKRKDEEVGATGT

F7DY34 MITEGLFDISATTVYSVSEQPFAEVVPTKFVRETDTSEWVFSTSLEGKKRKDEEVGATGT

************************************************************

A0A5F5PVK6 ASTVQAHSPTQRSDQLILPPELEGSNDTVSRDSASATRNGFMSLITPMQSEREMTSSTLV

F7DY34 ASTVQAHSPTQRSDQLILPPELEGSNDTVSRDSASATRNGFMSLITPMQSEREMTSSTLV

************************************************************

A0A5F5PVK6 FTETSVLDDLEAPTAEPSSSGQPVVQEGLSPAPGSPVSLFMEQGSGEAAADPEATTVSSF

F7DY34 FTETSVLDDLEAPTAEPSSSGQPVVQEGLSPAPGSPVSLFMEQGSGEAAADPEATTVSSF

************************************************************

A0A5F5PVK6 SFNLEPEIQTKKEAAGTLAPHVETILPFEPTGLVLSTVMDREVTEIINQTSKENLISEVS

F7DY34 SFNLEPEIQTKKEAAGTLAPHVETILPFEPTGLVLSTVMDREVTEIINQTSKENLISEVS

************************************************************

A0A5F5PVK6 GEPNHGAERKGFSTDFPLEEDFSGDLREYSTVSYPITKEETVVMEGSGDAAFKDTQILPS

F7DY34 GEPNHGAERKGFSTDFPLEEDFSGDLREYSTVSYPITKEETVVMEGSGDAAFKDTQILPS

************************************************************

A0A5F5PVK6 AIPTSDHITHIADLEGPGSTSVSTSAFPWEEFTASAEGSGEQLVPVSSSVDQVFPSAMGN

F7DY34 AIPTSDHITHIADLEGPGSTSVSTSAFPWEEFTASAEGSGEQLVPVSSSVDQVFPSAMGN

************************************************************

A0A5F5PVK6 VSGTEFPFIDQGLGEVGAITEANKRSTILPTAEAEGTEAPTEKGEVTVDGTVSVDFPQTM

F7DY34 VSGTEFPFIDQGLGEVGAITEANKRSTILPTAEAEGTEAPTEKGEVTVDGTVSVDFPQTM

************************************************************

A0A5F5PVK6 EPAKLWSRQEVNPVRQGNESEVASEENIQEQKSSESPQSSISPEQTIFDSQTFTESGLQT

F7DY34 EPAKLWSRQEVNPVRQGNESEVASEENIQEQKSSESPQSSISPEQTIFDSQTFTESGLQT

************************************************************

A0A5F5PVK6 TDYSTLMTKKTYRTDEEMEEEGISLVDVTTPDPDSKGLEPYTTLPEVTEKSHLFLVTTSE

F7DY34 TDYSTLMTKKTYRTDEEMEEEGISLVDVTTPDPDSKGLEPYTTLPEVTEKSHLFLVTTSE

************************************************************

A0A5F5PVK6 TESIPAESVVTDSSIKEEESIKSFPEVMRPISKESDTDLLFSGLGSGEEVLPTAVSVNFT

F7DY34 TESIPAESVVTDSSIKEEESIKSFPEVMRPISKESDTDLLFSGLGSGEEVLPTAVSVNFT

************************************************************

A0A5F5PVK6 DVEQIISTLYPQTSQVESVETSSLNDTTEDYKGIENVADEVRPLISKTDSISEDKETSPS

F7DY34 DVEQIISTLYPQTSQVESVETSSLNDTTEDYKGIENVADEVRPLISKTDSISEDKETSPS

************************************************************

A0A5F5PVK6 TTLSEISGDTRTEGPSTALLPFSTDTEHAQNQTRSWAEEIQTSRPQPTSEQVSSESSSTA

F7DY34 TTLSEISGDTRTEGPSTALLPFSTDTEHAQNQTRSWAEEIQTSRPQPTSEQVSSESSSTA

************************************************************

A0A5F5PVK6 ETKETATSSTDFLARTYGLEMAKEFVTSASKPSDSFYEHSGEGSGELDIVDLVRTSGTTQ

F7DY34 ETKETATSSTDFLARTYGLEMAKEFVTSASKPSDSFYEHSGEGSGELDIVDLVRTSGTTQ

************************************************************

A0A5F5PVK6 ATRQGGTTFVSDKSLERHPEVSRAEAVTVDGFPTVSVVVPLHLEQNASSPDPTSTPPSNT

F7DY34 ATRQGGTTFVSDKSLERHPEVSRAEAVTVDGFPTVSVVVPLHLEQNASSPDPTSTPPSNT

************************************************************

A0A5F5PVK6 VSSETSSEGAADSFQDHVRGFEDSTLKPDRRKATENIIIDLDKEDKDLILTITESTILEI

F7DY34 VSSETSSEGAADSFQDHVRGFEDSTLKPDRRKATENIIIDLDKEDKDLILTITESTILEI

************************************************************

A0A5F5PVK6 LPELTSDKNTIIDIDHTKPVYEDILGMPTNLDPEVPSGPHDSNEESVQEKYEAGFNLSST

F7DY34 LPELTSDKNTIIDIDHTKPVYEDILGMPTNLDPEVPSGPHDSNEESVQEKYEAGFNLSST

************************************************************

A0A5F5PVK6 EENFEGSGDLLPANYTQATHNQSMPSEDRSQVDHRGFIFTTGIPVSSTETELDILLPTAT

F7DY34 EENFEGSGDLLPANYTQATHNQSMPSEDRSQVDHRGFIFTTGIPVSSTETELDILLPTAT

************************************************************

A0A5F5PVK6 SLPIPSKSATVNPETEVPSTESKALDDIFESSTLSDGQAIADQSEIISTMGHLERTQDEY

F7DY34 SLPIPSKSATVNPETEVPSTESKALDDIFESSTLSDGQAIADQSEIISTMGHLERTQDEY

************************************************************

A0A5F5PVK6 EEKKYVGPSFQPEFSSGAEEALIDATPYVSISTIHLTAQSLTEAPNVLEGSSPPAYTDTT

F7DY34 EEKKYVGPSFQPEFSSGAEEALIDATPYVSISTIHLTAQSLTEAPNVLEGSSPPAYTDTT

************************************************************

A0A5F5PVK6 SAVSASAKLFSPTSSSPLTVHLGSGASEHTEGPQPSALLSTSASTSQMSPGKLANIDVTS

F7DY34 SAVSASAKLFSPTSSSPLTVHLGSGASEHTEGPQPSALLSTSASTSQMSPGKLANIDVTS

************************************************************

A0A5F5PVK6 KPSSEEYFYITEPPALPPDTQLEPSEDETKPKSSEQIEAAPTQLVAEEGTEIPRDSQNKT

F7DY34 KPSSEEYFYITEPPALPPDTQLEPSEDETKPKSSEQIEAAPTQLVAEEGTEIPRDSQNKT

************************************************************

A0A5F5PVK6 NVQLPGETIKIFASIRTPEAGTVVTAADEVKLERATSWPHSTSASVIYGIEAHVVPQPSP

F7DY34 NVQLPGETIKIFASIRTPEAGTVVTAADEVKLERATSWPHSTSASVIYGIEAHVVPQPSP

************************************************************

A0A5F5PVK6 QTPERPTVPSSLEVKPETQAALIRGEDFTVAAPEQQASARILDSNNQATVSTAELNTELA

F7DY34 QTPERPTVPSSLEVKPETQAALIRGEDFTVAAPEQQASARILDSNNQATVSTAELNTELA

************************************************************

A0A5F5PVK6 TPSFSLLETSNETDFLIGINEESVEGTAVYLPGPDRCKTNPCLNGGTCYPTETSYVCTCV

F7DY34 TPSFSLLETSNETDFLIGINEESVEGTAVYLPGPDRCKTNPCLNGGTCYPTETSYVCTCV

************************************************************

A0A5F5PVK6 PGYSGDQCELDFDECHSNPCRNGATCIDGFNTFRCLCLPSYIGALCEQDTETCDYGWHKF

F7DY34 PGYSGDQCELDFDECHSNPCRNGATCIDGFNTFRCLCLPSYIGALCEQDTETCDYGWHKF

************************************************************

A0A5F5PVK6 QGQCYKYFAHRRTWDAAERECRLQGAHLTSILSHEEQMFVNRVGHDYQWIGLNDKMFEHD

F7DY34 QGQCYKYFAHRRTWDAAERECRLQGAHLTSILSHEEQMFVNRVGHDYQWIGLNDKMFEHD

************************************************************

A0A5F5PVK6 FRWTDGSTLQYENWRPNQPDSFFSAGEDCVVIIWHENGQWNDVPCNYHLTYTCKKGTVAC

F7DY34 FRWTDGSTLQYENWRPNQPDSFFSAGEDCVVIIWHENGQWNDVPCNYHLTYTCKKGTVAC

************************************************************

A0A5F5PVK6 GQPPVVENAKTFGKMKPRYEINSLIRYHCKDGFIQRHPPTIRCLGNGKWAMPKITCMNPS

F7DY34 GQPPVVENAKTFGKMKPRYEINSLIRYHCKDGFIQRHPPTIRCLGNGKWAMPKITCMNPS

************************************************************

A0A5F5PVK6 AYQRTYSKKYFKNSSSAKDNSINTSKHEHRWSRRWQESRR

F7DY34 AYQRTYSKKYFKNSSSAKDNSINTSKHEHRWSRRWQESRR

****************************************
