# Supplementary material for: Bidirectional Interaction Between PGE2-Preconditioned Mesenchymal Stem Cells and Myofibroblasts Mediates Anti-Fibrotic Effects: A Proteomic Investigation into Equine Endometrial Fibrosis Reversal
Source: Proteomes. 2025 Sep 8;13(3):41. doi: 10.3390/proteomes13030041 (PMC12452512; doi:10.3390/proteomes13030041)
Supplement: Supplementary file 1 [file proteomes-13-00041-s001.zip › proteomes-3748745-supplementary-8.22/Table supplementary 2 DAPs in Secretome.docx]

| **DEGs in Secretome** | | | | | | | |
| --- | --- | --- | --- | --- | --- | --- | --- |
| **Protein.ID** | **GenName** | **Description** | **logFC** | **logCPM** | **PValue** | **FDR** | **DEGs** |
| **A0A3Q2L5E0** | **A0A3Q2L5E0** | **Keratin, type I cytoskeletal 19** | **-1,6674841** | **8,65831392** | **0,01002833** | **0,03399024** | **DownRegulated** |
| **A0A9L0SD08** | **A0A9L0SD08** | **Glutathione S-transferase (333 aa)** | **-2,164286** | **8,53529431** | **0,00222736** | **0,01146194** | **DownRegulated** |
| **A0A9L0TCU4** | **A0A9L0TCU4** | **EGF-like domain-containing protein (366aa)** | **-5,2792455** | **7,84873875** | **0,0040072** | **0,01749273** | **DownRegulated** |
| **A0A5F5PU06** | **A2M** | **Alpha-2-macroglobulin. (1480 aa)** | **-1,3707829** | **12,8599716** | **1,35E-05** | **0,0001949** | **DownRegulated** |
| **F6Z8K0** | **ACTG2** | **Actin gamma 2, smooth muscle; Belongs to the actin family. (408 aa)** | **-0,7610887** | **12,8514542** | **0,00109894** | **0,00664456** | **DownRegulated** |
| **F7B3I5** | **AFM** | **Afamin. (624 aa)** | **-1,4986712** | **9,9620217** | **0,004121** | **0,01787878** | **DownRegulated** |
| **P49066** | **AFP** | **Alpha-fetoprotein (609 aa)** | **-1,8272748** | **12,0019061** | **7,45E-14** | **1,31E-11** | **DownRegulated** |
| **A0A5F5PJ36** | **AHSG** | **Alpha 2-HS glycoprotein. (398 aa)** | **-0,8902842** | **11,0631952** | **0,00197215** | **0,0104539** | **DownRegulated** |
| **A0A3Q2H333** | **ALB** | **Albumin (637 aa)** | **-1,7745236** | **14,8304626** | **0,00030181** | **0,00237741** | **DownRegulated** |
| **A0A9L0TTQ9** | **ALDOB** | **Fructose-bisphosphate aldolase (398 aa)** | **-1,9665427** | **8,58395061** | **0,00548578** | **0,02235533** | **DownRegulated** |
| **A0A3Q2GSR0** | **ANXA5** | **Annexin. (326 aa)** | **-0,8471172** | **11,37021** | **0,0011494** | **0,00680945** | **DownRegulated** |
| **A0A5F5Q3H0** | **ANXA5** | **Annexin. (221 aa)** | **-0,7982424** | **10,7809742** | **0,01435999** | **0,04450017** | **DownRegulated** |
| **F6T7X3** | **AOC3** | **Amine oxidase. (763 aa)** | **-1,6206179** | **8,82076871** | **0,01257385** | **0,04038524** | **DownRegulated** |
| **A0A9L0RR06** | **APOA1** | **Apolipoprotein A1. (266 aa)** | **-1,9669111** | **10,6959723** | **2,84E-09** | **2,35E-07** | **DownRegulated** |
| **A0A3Q2HRX3** | **APOB** | **Apolipoprotein B. (4423 aa)** | **-1,4673513** | **10,5495029** | **4,53E-05** | **0,00054607** | **DownRegulated** |
| **A0A9L0RQ55** | **APOD** | **Apolipoprotein D. (213 aa)** | **-1,7718306** | **9,2124014** | **0,00174832** | **0,00955476** | **DownRegulated** |
| **A0A9L0SFM5** | **B4GAT1** | **Beta-1,4-glucuronyltransferase 1. (415 aa)** | **-2,2817771** | **8,85516991** | **0,0002378** | **0,0020031** | **DownRegulated** |
| **A0A9L0R124** | **BHMT** | **Betaine-homocysteine S-methyltransferase 1 (407 aa)** | **-1,4029081** | **9,71517451** | **0,0099107** | **0,03391768** | **DownRegulated** |
| **A0A9L0RP73** | **C3** | **Complement C3(1662 aa)** | **-1,742221** | **12,6656596** | **3,19E-08** | **1,73E-06** | **DownRegulated** |
| **A0A3Q2HWQ6** | **C3** | **Complement C3(1662aa)** | **-1,6570079** | **12,8639438** | **8,17E-08** | **3,60E-06** | **DownRegulated** |
| **A0A9L0SDT3** | **C3** | **Complement C3(1732 aa)** | **-1,6559128** | **12,8642238** | **1,33E-07** | **5,70E-06** | **DownRegulated** |
| **A0A9L0RMF3** | **C5** | **Complement C5. (1679 aa)** | **-2,0974908** | **9,32970204** | **0,00014209** | **0,00142089** | **DownRegulated** |
| **A0A5F5PGY6** | **C6** | **Complement C6. (954 aa)** | **-5,0878377** | **7,76396428** | **0,00292689** | **0,01335569** | **DownRegulated** |
| **A0A9L0TLN0** | **C7** | **Complement C7. (910 aa)** | **-1,4754974** | **9,64432244** | **0,00516408** | **0,02135293** | **DownRegulated** |
| **A0A3Q2H0H3** | **CACNA2D1** | **Calcium voltage-gated channel auxiliary subunit alpha2delta 1. (1106 aa)** | **-1,9217451** | **9,37511936** | **0,00068003** | **0,00462415** | **DownRegulated** |
| **A0A3Q2IDD2** | **CFB** | **Complement factor B (768 aa)** | **-1,7751218** | **10,4153536** | **1,81E-05** | **0,00024971** | **DownRegulated** |
| **A0A3Q2I6U4** | **CNTN1** | **Contactin 1. (1032 aa)** | **-1,5844563** | **10,3875744** | **0,00092655** | **0,00591146** | **DownRegulated** |
| **A0A5F5PKC8** | **COL11A1** | **Collagen type XI alpha 1 chain. (1818 aa)** | **-3,1824456** | **8,57670843** | **1,33E-05** | **0,00019315** | **DownRegulated** |
| **A0A9L0QZF3** | **COMP** | **Cartilage oligomeric matrix protein. (793 aa)** | **-1,3878716** | **11,1885714** | **5,84E-06** | **0,00010972** | **DownRegulated** |
| **F6WZA8** | **CPN1** | **Carboxypeptidase N subunit 1. (462 aa)** | **-1,8605598** | **8,52401876** | **0,00949207** | **0,03308277** | **DownRegulated** |
| **F6PVG3** | **EFEMP1** | **EGF containing fibulin extracellular matrix protein 1. (614 aa)** | **-1,1660912** | **10,8226217** | **0,00013607** | **0,00138032** | **DownRegulated** |
| **A0A3Q2I5Q7** | **EGFR** | **Receptor protein-tyrosine kinase. (1195 aa)** | **-2,6607302** | **8,65101618** | **0,00047477** | **0,00338091** | **DownRegulated** |
| **A0A3Q2H8V4** | **ENPP2** | **Ectonucleotide pyrophosphatase/phosphodiesterase 2. (1011 aa)** | **-1,6688358** | **9,00969162** | **0,00813853** | **0,03030657** | **DownRegulated** |
| **F7BFJ1** | **F2** | **Prothrombin. (622 aa)** | **-1,5504446** | **10,2647508** | **0,00045649** | **0,00328391** | **DownRegulated** |
| **F7DZ01** | **F5** | **Coagulation factor V (2266 aa)** | **-1,7791876** | **10,603319** | **6,59E-07** | **1,94E-05** | **DownRegulated** |
| **F7D281** | **F7D281** | **IF rod domain-containing protein (595 aa)** | **-2,1271525** | **10,1180548** | **0,00142764** | **0,00814968** | **DownRegulated** |
| **A0A3Q2GXX5** | **FBLN1** | **Fibulin-1; for (705 aa)** | **-1,0887672** | **11,7245901** | **8,45E-06** | **0,00014019** | **DownRegulated** |
| **F6T0P6** | **GC** | **GC vitamin D binding protein. (522 aa)** | **-1,6540299** | **12,9418756** | **0,00015671** | **0,00153445** | **DownRegulated** |
| **Q28372** | **GSN** | **Gelsolin (731 aa)** | **-0,9854814** | **12,3933279** | **0,00345652** | **0,01552133** | **DownRegulated** |
| **P02062** | **HBB** | **Hemoglobin subunit beta(146 aa)** | **-1,6358492** | **11,867373** | **1,62E-07** | **6,51E-06** | **DownRegulated** |
| **A0A3Q2H2J4** | **HGD** | **Homogentisate 1,2-dioxygenase. (446 aa)** | **-4,0703992** | **9,20616641** | **8,53E-09** | **5,73E-07** | **DownRegulated** |
| **A0A3Q2HCG1** | **HGFAC** | **HGF activator. (719 aa)** | **-1,2569753** | **9,92715402** | **0,008685** | **0,03130576** | **DownRegulated** |
| **A0A9L0S0M8** | **HPD** | **4-hydroxyphenylpyruvate dioxygenase. (393 aa)** | **-2,0763708** | **9,47217742** | **0,00015196** | **0,00149834** | **DownRegulated** |
| A0A3Q2GTN6 | HSPB1 | Heat shock protein beta-1 (189 aa) | -0,9150688 | 10,7331516 | 0,00374248 | 0,01649029 | DownRegulated |
| A0A9L0SNS8 | IL1RAP | Interleukin 1 receptor accessory protein. (687 aa) | -1,9264832 | 8,87344021 | 0,00292509 | 0,01335569 | DownRegulated |
| F6PKE1 | INHCA | Transferrin(656 aa) | -1,66301 | 9,00564634 | 0,00837599 | 0,03051719 | DownRegulated |
| A0A3Q2GWN9 | INHCA | Serotransferrin (707 aa) | -1,5167394 | 13,1401517 | 1,95E-05 | 0,00026428 | DownRegulated |
| A0A9L0S1S1 | ITIH1 | Inter-alpha-trypsin inhibitor heavy chain 1. (908 aa) | -2,0750482 | 8,96671168 | 0,00103281 | 0,00638714 | DownRegulated |
| A0A5F5PM55 | ITIH2 | Inter-alpha-trypsin inhibitor heavy chain 2. (946 aa) | -1,5171839 | 11,8557097 | 5,90E-11 | 6,93E-09 | DownRegulated |
| A0A3Q2HN28 | ITIH3 | Inter-alpha-trypsin inhibitor heavy chain 3. (890 aa) | -1,6385322 | 11,91377 | 4,02E-11 | 5,15E-09 | DownRegulated |
| A0A5F5PUE2 | KNG1 | Kininogen 1. (653 aa) | -1,9270721 | 9,63392572 | 0,00028547 | 0,00231327 | DownRegulated |
| A0A5F5PMV0 | KRT1 | Keratin, type II cytoskeletal 1 (626 aa) | -2,0606071 | 9,59643728 | 0,00025093 | 0,00208121 | DownRegulated |
| F7B7X0 | KRT1 | Keratin, type II cytoskeletal 1 (606 aa) | -1,8073699 | 10,4216823 | 7,72E-06 | 0,00013108 | DownRegulated |
| A0A9L0R3G9 | KRT10A | Keratin, type I cytoskeletal 10 (587 aa) | -1,5908648 | 11,7933601 | 7,20E-08 | 3,38E-06 | DownRegulated |
| A0A5F5PUZ8 | KRT16 | Keratin 16 (518 aa) | -2,345675 | 9,64897271 | 6,98E-05 | 0,00077514 | DownRegulated |
| A0A3Q2GXH5 | KRT42 | Keratin, type I cytoskeletal 17(419 aa) | -2,1999899 | 8,80573793 | 0,00042394 | 0,00306539 | DownRegulated |
| F7ATL5 | KRT42 | Keratin, type I cytoskeletal 17 (470aa) | -1,986397 | 9,26029146 | 0,00029415 | 0,00234319 | DownRegulated |
| F7AGY4 | KRT73 | Keratin 73 (540 aa) | -3,4952944 | 8,18407696 | 0,00307815 | 0,01400061 | DownRegulated |
| F6XWG3 | KRT79 | Keratin 79(507 aa) | -2,0250411 | 8,711105 | 0,00211165 | 0,0111098 | DownRegulated |
| F7A808 | KRT80 | Keratin 80 (503 aa) | -1,8063887 | 8,6186268 | 0,01294723 | 0,04111619 | DownRegulated |
| A0A3Q2I6F9 | KRT82 | Keratin 82 (521 aa) | -2,4480149 | 8,5574544 | 0,00157324 | 0,00869908 | DownRegulated |
| F7DYB1 | LOC100051073 | Alpha-amylase. (511 aa) | -1,7492857 | 9,64864365 | 0,00096621 | 0,00607644 | DownRegulated |
| A0A9L0R7H5 | LOC100059239 | C4a anaphylatoxin (1744 aa) | -1,6041584 | 11,469245 | 8,16E-11 | 8,85E-09 | DownRegulated |
| F6QAD8 | LOC100061656 | Alpha-2-macroglobulin(1467 aa) | -1,5402975 | 12,1625665 | 2,17E-07 | 8,05E-06 | DownRegulated |
| F6REX3 | LOC100061763 | Pregnancy zone protein (1477 aa) | -1,491068 | 9,9200869 | 0,00245298 | 0,01209336 | DownRegulated |
| P02801 | LOC100630543 | Metallothionein-1B (61aa) | -1,6839651 | 8,94361735 | 0,00468722 | 0,01978734 | DownRegulated |
| O77811 | LTF | Lactotransferrin (695 aa) | -1,3295752 | 10,4738166 | 0,00023355 | 0,0020031 | DownRegulated |
| Q8MJV0 | MYH1 | Myosin-1 (1940 aa) | -4,5225228 | 8,32519116 | 0,00181789 | 0,0098966 | DownRegulated |
| A0A5F5PP47 | MYH6 | Myosin-6(1850 aa) | -4,95798 | 8,61072754 | 0,00018284 | 0,00173019 | DownRegulated |
| Q8MJU9 | MYH7 | Myosin-7 (1850 aa) | -4,8792762 | 8,55689662 | 0,00036012 | 0,00272991 | DownRegulated |
| A0A9L0RCU7 | OGN | Mimecan(298 aa) | -3,5606398 | 7,83571418 | 0,00492381 | 0,02060112 | DownRegulated |
| A0A9L0SQ87 | PLG | Plasminogen(878 aa) | -1,688638 | 10,6713744 | 1,59E-06 | 3,81E-05 | DownRegulated |
| A0A3Q2HZI0 | PLP1 | Proteolipid protein 1. (396 aa) | -1,9874077 | 8,32567364 | 0,01475658 | 0,04523211 | DownRegulated |
| A0A3Q2H7K2 | POSTN | Periostin. (836 aa) | -1,71116 | 11,0968219 | 1,28E-08 | 7,84E-07 | DownRegulated |
| Q28369 | RBP4 | Retinol-binding protein 4 (201 aa) | -1,820804 | 10,8544493 | 9,08E-09 | 5,82E-07 | DownRegulated |
| A0A0B4J1C3 | RGN | Regucalcin (299 aa) | -2,8745658 | 8,41478132 | 0,00182706 | 0,00990827 | DownRegulated |
| F6ZRF6 | SERPINA7 | Thyroxine-binding globulin (437 aa) | -1,3711197 | 9,72777695 | 0,00624655 | 0,02453379 | DownRegulated |
| A0A9L0S3A7 | SERPINC1 | Antithrombin-III (463 aa) | -1,3344166 | 11,0918455 | 9,83E-07 | 2,77E-05 | DownRegulated |
| A0A9L0RZN5 | SERPINH1 | Serpin family H member 1 (411 aa) | -0,7445893 | 12,3093315 | 0,00150262 | 0,00841937 | DownRegulated |
| F6PWV8 | SERPINH1 | Serpin family H member 1 (412 aa) | -0,6707382 | 12,2594125 | 0,00603623 | 0,0239749 | DownRegulated |
| F6QSC5 | TG | Thyroglobulin. (2768 aa) | -2,430963 | 9,47641508 | 0,00013177 | 0,00136096 | DownRegulated |
| A0A9L0R791 | THBS4 | Thrombospondin 4. (961 aa) | -1,735346 | 10,2602206 | 2,69E-05 | 0,00035493 | DownRegulated |
| A0A5F5PHR4 | VCL | Vinculin. (1284 aa) | -0,6559505 | 12,3196412 | 0,00350284 | 0,01567937 | DownRegulated |
| F6V881 | VTN | Vitronectin. (478 aa) | -2,5187447 | 8,23119856 | 0,01054794 | 0,03532681 | DownRegulated |
| A0A5F5PYF1 | YWHAE | Tyrosine 3-monooxygenase/tryptophan 5-monooxygenase activation protein epsilon (240 aa) | -0,8539981 | 11,303922 | 0,00079579 | 0,00526793 | DownRegulated |
| A0A3Q2HE43 | A0A3Q2HE43 | FUS RNA binding protein (508aa) | 4,96919468 | 8,22458904 | 0,01391222 | 0,0433552 | UpRegulated |
| A0A3Q2HSD4 | A0A3Q2HSD4 | UBC core domain-containing protein(212 aa) | 2,69871583 | 8,98597983 | 0,0012717 | 0,00736619 | UpRegulated |
| A0A3Q2I9R9 | A0A3Q2I9R9 | Glutathione S-transferase (334 aa) | 3,56933191 | 8,95228032 | 0,00013224 | 0,00136096 | UpRegulated |
| A0A5F5PN84 | A0A5F5PN84 | AHNAK nucleoprotein 2 (2061aa) | 5,90747584 | 8,79039314 | 5,13E-05 | 0,00059783 | UpRegulated |
| A0A5F5Q1J8 | A0A5F5Q1J8 | Large ribosomal subunit protein uL10 (255 aa) | 2,29723617 | 8,73132751 | 0,00682696 | 0,02641478 | UpRegulated |
| A0A9L0R8X8 | A0A9L0R8X8 | NADP-dependent oxidoreductase domain-containing protein | 5,03046283 | 8,24315708 | 0,00835164 | 0,03051719 | UpRegulated |
| A0A9L0RPI3 | A0A9L0RPI3 | UV excision repair protein RAD23 (354 aa) | 3,88864633 | 9,17709003 | 1,10E-05 | 0,00016951 | UpRegulated |
| A0A9L0S2L4 | A0A9L0S2L4 | AHNAK nucleoprotein 2 | 5,81965516 | 8,73041991 | 6,84E-05 | 0,00076537 | UpRegulated |
| A0A9L0S410 | A0A9L0S410 | Elongation factor 1-alpha (462 aa) | 0,97926857 | 10,7766266 | 0,00539135 | 0,02209828 | UpRegulated |
| A0A9L0S4D7 | A0A9L0S4D7 | NADP-dependent oxidoreductase domain-containing protein (374 aa) | 5,03047362 | 8,24315708 | 0,00835889 | 0,03051719 | UpRegulated |
| A0A9L0SAI6 | A0A9L0SAI6 | AHNAK nucleoprotein 2 (1877aa) | 5,56734436 | 8,56504523 | 0,00023533 | 0,0020031 | UpRegulated |
| A0A9L0T4M1 | A0A9L0T4M1 | Collagen type VII alpha 1 chain (2942 aa) | 2,38464717 | 11,2795676 | 9,17E-14 | 1,44E-11 | UpRegulated |
| A0A3Q2I6Z2 | AARS1 | Alanine--tRNA ligase (977 aa) | 6,65173349 | 9,32873796 | 1,05E-06 | 2,79E-05 | UpRegulated |
| A0A3Q2HPV4 | ACLY | ATP-citrate synthase (1098 aa) | 7,02959177 | 9,61122344 | 3,61E-07 | 1,24E-05 | UpRegulated |
| F6WM79 | ACTR2 | Actin-related protein 2 (392 aa) | 1,99286869 | 8,9166858 | 0,00963454 | 0,03329583 | UpRegulated |
| A0A5F5PZN1 | ADAMTS5 | ADAM metallopeptidase with thrombospondin type 1 motif 5. (929 aa) | 2,45776686 | 9,22897968 | 0,00148544 | 0,00837788 | UpRegulated |
| F6W0M1 | ADAMTS7 | ADAM metallopeptidase with thrombospondin type 1 motif 7. (1686 aa) | 4,95603711 | 8,22076774 | 0,01404649 | 0,04362456 | UpRegulated |
| P19854 | ADH5 | Alcohol dehydrogenase class-3 (374 aa) | 5,87409448 | 8,76920664 | 2,82E-05 | 0,00036174 | UpRegulated |
| A0A3Q2HJF2 | AHNAK | AHNAK nucleoprotein. (5865 aa) | 6,10785162 | 10,948386 | 3,18E-24 | 2,25E-21 | UpRegulated |
| F7DMQ3 | AKR1B1 | Aldo-keto reductase family 1 member B. (378 aa) | 3,01165355 | 9,2025702 | 0,00023604 | 0,0020031 | UpRegulated |
| A0A3Q2HCD2 | AKR1B1 | Aldo-keto reductase family 1 member B. (378 aa) | 3,48339896 | 8,89077425 | 0,00036953 | 0,00276043 | UpRegulated |
| A0A3Q2GZ81 | ALCAM | Activated leukocyte cell adhesion molecule. (583 aa) | 6,29500471 | 9,06375503 | 1,85E-06 | 4,06E-05 | UpRegulated |
| F7CVL4 | ALDH1A2 | Aldehyde dehydrogenase 1 family member A2; Belongs to the aldehyde dehydrogenase family. (518 aa) | 5,62482601 | 8,60331959 | 0,00038596 | 0,00286422 | UpRegulated |
| P00883 | ALDOA | Fructose-bisphosphate aldolase; Belongs to the class I fructose-bisphosphate aldolase family. (477 aa) | 5,96727884 | 8,87541851 | 0,01464003 | 0,04516945 | UpRegulated |
| F6X3M3 | ALOX12 | Arachidonate 12-lipoxygenase, 12S type. (663 aa) | 5,97448024 | 8,8382485 | 3,52E-05 | 0,00044749 | UpRegulated |
| A0A9L0QYB9 | ANGPTL2 | Angiopoietin like 2. (494 aa) | 4,85499825 | 8,14547943 | 0,01470858 | 0,04519101 | UpRegulated |
| A0A9L0TQP3 | AP1B1 | Adaptor related protein complex 1 subunit beta 1. (969 aa) | 2,93953866 | 8,54779447 | 0,01002574 | 0,03399024 | UpRegulated |
| A0A9L0R5S8 | AP2A2 | AP-2 complex subunit alpha (910 aa) | 5,39337784 | 8,45881552 | 0,00088228 | 0,00568044 | UpRegulated |
| A0A3Q2LQ45 | AP2B1 | AP complex subunit beta (937 aa) | 2,7863118 | 9,04593679 | 0,00221486 | 0,01146194 | UpRegulated |
| A0A9L0RWG4 | APEX1 | DNA-(apurinic or apyrimidinic site) endonuclease (432 aa) | 5,09689798 | 8,27796737 | 0,0060177 | 0,0239749 | UpRegulated |
| A0A3Q2I292 | API5 | Tetratricopeptide repeat domain 17. (1198 aa) | 4,42222676 | 7,92364652 | 0,01676699 | 0,04977148 | UpRegulated |
| A0A3Q2IE84 | ARCN1 | Coatomer subunit delta; (519 aa) | 5,92063689 | 8,80079899 | 4,82E-05 | 0,00056643 | UpRegulated |
| A0A9L0R2I2 | ARF4 | ADP-ribosylation factor (162 aa) | 1,78396169 | 9,07483849 | 0,01670988 | 0,04977148 | UpRegulated |
| A0A3Q2I591 | ARPC1B | Actin related protein 2/3 complex subunit 1B. (408 aa) | 1,98752337 | 9,20340374 | 0,0121103 | 0,03916403 | UpRegulated |
| F7APR4 | ARPC2 | Arp2/3 complex 34 kDa subunit (301 aa) | 3,44737662 | 8,86862654 | 0,00095423 | 0,00603346 | UpRegulated |
| A0A5F5PPW1 | ARPC5 | Actin-related protein 2/3 complex subunit 5 (136 aa) | 3,11198378 | 8,6535762 | 0,00393986 | 0,01725217 | UpRegulated |
| F7B570 | ARPC5 | Actin-related protein 2/3 complex subunit 5 (154 aa) | 2,87656375 | 8,50985687 | 0,00990727 | 0,03391768 | UpRegulated |
| A0A9L0RQ86 | ATIC | Bifunctional purine biosynthesis protein ATIC (562 aa) | 1,9009115 | 9,91650428 | 0,00441547 | 0,01892342 | UpRegulated |
| A0A3Q2GXY5 | ATP6AP2 | Renin receptor (342 aa) | 4,94145711 | 8,19115041 | 0,01084331 | 0,03568381 | UpRegulated |
| F6YTB8 | BMP1 | Metalloendopeptidase. (1083 aa) | 6,03380664 | 8,87689193 | 1,61E-05 | 0,00022859 | UpRegulated |
| A0A3Q2GVR9 | BSG | Basigin. (271 aa) | 4,95439176 | 8,19928798 | 0,01078003 | 0,03568381 | UpRegulated |
| A0A3Q2GSD5 | CALD1 | Caldesmon 1. (562 aa) | 1,4641052 | 10,0897532 | 0,0059105 | 0,02381089 | UpRegulated |
| A0A5F5PK83 | CAPN2 | Calpain 2 (732 aa) | 7,01851055 | 9,60660813 | 1,59E-07 | 6,51E-06 | UpRegulated |
| A0A9L0S1R2 | CAPZB | F-actin-capping protein subunit beta (357 aa) | 2,18669229 | 9,58095759 | 0,00213956 | 0,0112148 | UpRegulated |
| F7D854 | CAT | Catalase. (598 aa) | 1,38880825 | 9,82380858 | 0,01557839 | 0,04699923 | UpRegulated |
| F6X0K3 | CCN2 | Cellular communication network factor 2. (475 aa) | 4,46811464 | 9,60181306 | 1,41E-06 | 3,62E-05 | UpRegulated |
| A0A3Q2GXR2 | CCT2 | T-complex protein 1 subunit beta (538 aa) | 2,45209893 | 9,22089656 | 0,00127994 | 0,00736619 | UpRegulated |
| F6V3D5 | CCT3 | T-complex protein 1 subunit gamma. (528 aa) | 3,43189523 | 9,938134 | 3,00E-06 | 6,04E-05 | UpRegulated |
| F6Z6Q7 | CCT4 | T-complex protein 1 subunit delta. (539 aa) | 5,91996689 | 8,80118568 | 2,45E-05 | 0,0003264 | UpRegulated |
| F6UH83 | CCT5 | T-complex protein 1 subunit epsilon (541 aa) | 5,96898261 | 8,83514532 | 2,40E-05 | 0,00032294 | UpRegulated |
| A0A5F5PMG6 | CCT6B | Chaperonin containing TCP1 subunit 6B (589 aa) | 4,66206088 | 8,04094384 | 0,00952477 | 0,03308277 | UpRegulated |
| F6XSN2 | CCT7 | T-complex protein 1 subunit eta (579 aa) | 4,06721932 | 9,30695465 | 6,72E-06 | 0,00011838 | UpRegulated |
| F6ZQE2 | CCT8 | T-complex protein 1 subunit theta (534 aa) | 3,93196237 | 9,89037216 | 3,23E-07 | 1,14E-05 | UpRegulated |
| A0A3Q2I0B3 | CD81 | Tetraspanin. (259 aa) | 5,24944087 | 8,36945718 | 0,00582698 | 0,02354165 | UpRegulated |
| A0A9L0SD60 | CDC42 | Cell division control protein 42-like protein. (192 aa) | 4,85111667 | 8,14068674 | 0,01328087 | 0,04189268 | UpRegulated |
| F6VUR4 | CKAP4 | Serine/threonine-protein phosphatase. (848 aa) | 5,82120055 | 8,73112556 | 5,75E-05 | 0,00065402 | UpRegulated |
| A0A3Q2GYQ4 | CLTC | Clathrin heavy chain (1681aa) | 2,15242135 | 10,9477805 | 4,81E-07 | 1,51E-05 | UpRegulated |
| A0A3Q2HEE5 | CNN2 | Calponin (270 aa) | 5,73865236 | 8,68058882 | 0,00011047 | 0,00116236 | UpRegulated |
| F7DCH4 | CNN3 | Calponin (336 aa) | 5,62745685 | 8,60634645 | 0,00029211 | 0,00234017 | UpRegulated |
| A0A9L0S7L5 | CNN3 | Calponin (329 aa) | 1,95607731 | 8,89635829 | 0,01386722 | 0,04335428 | UpRegulated |
| A0A3Q2I3N3 | COL4A2 | Collagen type IV alpha 2 chain. (1712 aa) | 1,84095927 | 9,72173958 | 0,00248752 | 0,01222091 | UpRegulated |
| A0A5F5PR28 | COL5A2 | Collagen type V alpha 2 chain. (1481 aa) | 1,45853996 | 10,3612618 | 0,00096964 | 0,00607644 | UpRegulated |
| A0A5F5PTP6 | COL6A3 | Collagen type VI alpha 3 chain. (3273 aa) | 0,90404505 | 12,2828536 | 0,00090013 | 0,00576904 | UpRegulated |
| A0A3Q2HWW0 | COPA | Coatomer subunit alpha (1234 aa) | 6,35120484 | 9,10473597 | 1,73E-06 | 4,00E-05 | UpRegulated |
| F6YZP7 | COPB1 | Coatomer subunit beta (953 aa) | 2,45073142 | 9,51505651 | 0,00218837 | 0,01138601 | UpRegulated |
| A0A3Q2HN97 | COPB2 | Coatomer subunit beta (937 aa) | 3,69725014 | 9,70754105 | 4,43E-06 | 8,55E-05 | UpRegulated |
| F7CY72 | COPG1 | Coatomer subunit gamma (814 aa) | 6,23754704 | 9,02297657 | 2,65E-06 | 5,42E-05 | UpRegulated |
| A0A9L0RV49 | COPG2 | Coatomer subunit gamma (758 aa) | 4,84940356 | 8,13981156 | 0,01524653 | 0,04653161 | UpRegulated |
| F7CVF5 | CORO1B | Coronin (489 aa) | 4,82000321 | 8,13244745 | 0,0082261 | 0,03044304 | UpRegulated |
| A0A9L0R585 | CPE | Carboxypeptidase E. (476 aa) | 5,51322076 | 8,53373333 | 0,00040058 | 0,00294177 | UpRegulated |
| A0A3Q2H843 | CRIP2 | Cysteine rich protein 2. (208 aa) | 5,72383812 | 8,66703141 | 0,00012388 | 0,0012939 | UpRegulated |
| A0A9L0SV04 | CRIP2 | Cysteine rich protein 2. (208 aa) | 5,15804365 | 8,31892627 | 0,0107158 | 0,03568381 | UpRegulated |
| F7BME5 | CRK | Adapter molecule crk. (304 aa) | 5,02448678 | 8,23724402 | 0,0095341 | 0,03308277 | UpRegulated |
| A0A9L0S3E1 | CRLF1 | Cytokine receptor like factor 1. (421 aa) | 5,12259299 | 8,29421667 | 0,00683787 | 0,02641478 | UpRegulated |
| A0A9L0R5T7 | CSPG4 | Chondroitin sulfate proteoglycan 4. (2321 aa) | 3,40508096 | 9,91819959 | 7,88E-05 | 0,00086082 | UpRegulated |
| A0A3Q2HN08 | CTSS | Cathepsin S (332 aa) | 5,10831746 | 8,28671022 | 0,00471536 | 0,01984674 | UpRegulated |
| Q8MIN2 | CXCL6 | C-X-C motif chemokine 6 (113 aa) | 5,16895682 | 8,32426336 | 0,01392901 | 0,0433552 | UpRegulated |
| A0A9L0SA98 | CXCL8 | C-X-C motif chemokine (95 aa) | 4,94925656 | 8,19501057 | 0,01333205 | 0,04196026 | UpRegulated |
| A0A5F5PGQ0 | DBN1 | PDZ and LIM domain protein 7 (476 aa) | 5,55662203 | 8,55857262 | 0,00078085 | 0,00519335 | UpRegulated |
| A0A9L0SBP6 | DBN1 | Drebrin 1. (770aa) | 5,38449788 | 8,44894988 | 0,00086015 | 0,00561484 | UpRegulated |
| A0A5F5PJ10 | DBNL | Drebrin like. (565 aa) | 5,50556905 | 8,52717884 | 0,00110147 | 0,00664456 | UpRegulated |
| A0A9L0T5N9 | DCTN2 | Dynactin subunit 2. (422 aa) | 5,3134981 | 8,40763291 | 0,00257083 | 0,01241392 | UpRegulated |
| A0A9L0SEP4 | DDB1 | Damage specific DNA binding protein 1. (1286 aa) | 4,94525222 | 8,19414882 | 0,009347 | 0,03284429 | UpRegulated |
| A0A3Q2H9C5 | DDX17 | RNA helicase (644 aa) | 5,72386269 | 8,66775548 | 0,00026838 | 0,00220101 | UpRegulated |
| A0A5F5PW07 | DDX39B | DExD-box helicase 39B. (428 aa) | 1,60149846 | 9,55374331 | 0,01086773 | 0,03568381 | UpRegulated |
| A0A5F5Q254 | DDX5 | DEAD-box helicase 5 (614 aa) | 5,5669719 | 8,56540013 | 0,0003408 | 0,00259744 | UpRegulated |
| A0A3Q2HPC0 | DNPEP | Aspartyl aminopeptidase (526 aa) | 5,78999822 | 8,71193175 | 0,00020554 | 0,00184592 | UpRegulated |
| F6TNY4 | DPP3 | Dipeptidyl peptidase 3 (759 aa) | 5,10833676 | 8,28671022 | 0,00473437 | 0,01986745 | UpRegulated |
| A0A9L0S8Y9 | DPT | Dermatopontin. (210 aa) | 1,82955909 | 9,33446947 | 0,01012014 | 0,0341373 | UpRegulated |
| A0A3Q2H363 | DPYSL2 | Dihydropyrimidinase-related protein 2 (678 aa) | 1,72520259 | 10,7776246 | 2,25E-06 | 4,80E-05 | UpRegulated |
| A0A9L0SCI0 | DPYSL3 | Dihydropyrimidinase like 3 (684 aa) | 2,39329048 | 10,4156196 | 9,41E-07 | 2,71E-05 | UpRegulated |
| F7CL80 | DSTN | Destrin, actin depolymerizing factor (176 aa) | 3,525707 | 8,9206565 | 0,00021624 | 0,00190558 | UpRegulated |
| A0A9L0REI6 | DYNC1H1 | Dynein cytoplasmic 1 heavy chain 1 (4617 aa) | 3,86764445 | 9,15891358 | 0,00104846 | 0,00642752 | UpRegulated |
| A0A3Q2GTG5 | DYNLL2 | Dynein light chain (239 aa) | 3,04880196 | 8,60893205 | 0,00701993 | 0,02697029 | UpRegulated |
| F6QYS3 | ECM1 | Extracellular matrix protein 1. (543 aa) | 0,78133127 | 11,7123356 | 0,00226099 | 0,01146761 | UpRegulated |
| A0A3Q2HT17 | EDIL3 | EGF like repeats and discoidin domains 3 (470 aa) | 5,18622898 | 8,33329945 | 0,00269829 | 0,01276513 | UpRegulated |
| A0A9L0RZ21 | EEF1A1 | Elongation factor 1-alpha (425aa ) | 0,82468727 | 10,940011 | 0,00999203 | 0,03399024 | UpRegulated |
| A2Q0Z0 | EEF1A1 | Elongation factor 1-alpha 1 (462 aa) | 0,80512921 | 11,0017703 | 0,01533434 | 0,04662115 | UpRegulated |
| A0A9L0TM85 | EIF2S1 | Eukaryotic translation initiation factor subunit 1-like protein. (317 aa) | 5,11459847 | 8,2912979 | 0,00543838 | 0,02222642 | UpRegulated |
| A0A9L0SL37 | EIF3E | Eukaryotic translation initiation factor 3 subunit E (460 aa) | 5,04844216 | 8,24992485 | 0,0079988 | 0,03003433 | UpRegulated |
| A0A9L0TC24 | EIF3E | Eukaryotic translation initiation factor 3 subunit E (445 aa) | 5,04846717 | 8,24992485 | 0,00800915 | 0,03003433 | UpRegulated |
| F7D5C0 | ELOC | Elongin C (112 aa) | 5,02778515 | 8,24019983 | 0,00834809 | 0,03051719 | UpRegulated |
| F6SMZ4 | EMILIN1 | Elastin microfibril interfacer 1. (1016 aa) | 7,60381252 | 10,0797151 | 2,14E-11 | 3,01E-09 | UpRegulated |
| A0A3Q2HWB8 | ENAH | ENAH actin regulator. (816 aa) | 5,18287522 | 8,32960012 | 0,00267298 | 0,01273279 | UpRegulated |
| A0A9L0RN75 | ENOPH1 | Enolase-phosphatase (246 aa) | 5,01750989 | 8,23428966 | 0,01270825 | 0,04053989 | UpRegulated |
| A0A9L0S3J4 | ERO1A | Endoplasmic reticulum oxidoreductase 1 alpha. (468 aa) | 4,9473547 | 8,19456295 | 0,0115145 | 0,03740886 | UpRegulated |
| A0A9L0RSF5 | ERP29 | Endoplasmic reticulum resident protein 29. (341 aa) | 5,66889584 | 8,62950365 | 0,00019027 | 0,00177934 | UpRegulated |
| A0A9L0SY49 | ERP44 | Endoplasmic reticulum protein 44. (415 aa) | 2,32645826 | 9,43571363 | 0,00077745 | 0,00519335 | UpRegulated |
| A0A5F5PER4 | EWSR1 | EWS RNA binding protein 1. (661 aa) | 5,31565683 | 8,40643001 | 0,00169523 | 0,0093007 | UpRegulated |
| F7B6C3 | F11R | F11 receptor. (307 aa) | 4,63757577 | 8,03238456 | 0,01689299 | 0,04993526 | UpRegulated |
| F6T1A4 | F6T1A4 | Large ribosomal subunit protein uL11(377aa) | 3,43946536 | 8,86347238 | 0,00039653 | 0,00292726 | UpRegulated |
| F6TTP1 | F6TTP1 | Large ribosomal subunit protein uL10(332aa) | 2,39887434 | 8,79538441 | 0,00424732 | 0,01831412 | UpRegulated |
| F6Z2I7 | F6Z2I7 | Glutathione S-transferase (300aa) | 3,26229835 | 8,74695872 | 0,00140763 | 0,00806814 | UpRegulated |
| F6W3W7 | FAM171B | Family with sequence similarity 171 member B(978aa) | 5,57925008 | 8,57303082 | 0,00068214 | 0,00462415 | UpRegulated |
| A0A3Q2HV34 | FAM171B | Family with sequence similarity 171 member B(983aa) | 5,18705778 | 8,33247691 | 0,00342748 | 0,01544006 | UpRegulated |
| A0A5F5PLA1 | FASN | Fatty acid synthase. (2505 aa) | 6,47830692 | 9,17412056 | 0,00185143 | 0,01000197 | UpRegulated |
| A0A3Q2GWF8 | FBLN2 | Fibulin 2. (1183 aa) | 2,96789447 | 10,3788716 | 6,42E-08 | 3,23E-06 | UpRegulated |
| F7CKI7 | FBN1 | Fibrillin 1. (2874 aa) | 5,32652274 | 11,035871 | 4,11E-23 | 1,93E-20 | UpRegulated |
| A0A9L0STN9 | FBN1 | Fibrillin 1. (2827aa) | 5,22780804 | 10,9512871 | 3,07E-21 | 1,08E-18 | UpRegulated |
| A0A9L0S7S8 | FERMT2 | Fermitin family member 2. (707 aa) | 2,24160506 | 9,6186843 | 0,00123261 | 0,00718175 | UpRegulated |
| F6Z272 | FHL2 | Four and a half LIM domains 2. (279 aa) | 5,37934316 | 8,44775992 | 0,00242878 | 0,01201607 | UpRegulated |
| A0A9L0T2S0 | FKBP10 | Peptidylprolyl isomerase. (583 aa) | 2,22649578 | 8,68879971 | 0,01599856 | 0,04799568 | UpRegulated |
| A0A5F5PI89 | FKBP3 | Peptidylprolyl isomerase. (273 aa) | 5,82735796 | 8,73755072 | 4,77E-05 | 0,0005654 | UpRegulated |
| A0A5F5PGB0 | FKBP9 | Peptidylprolyl isomerase. (616 aa) | 5,71865642 | 8,66341094 | 0,00023867 | 0,0020031 | UpRegulated |
| A0A3Q2H1S8 | FLNB | Filamin B. (2633 aa) | 1,90311159 | 11,656732 | 6,02E-10 | 5,40E-08 | UpRegulated |
| A0A5F5PG69 | FLNC | Filamin C. (2917 aa) | 1,66512606 | 11,5957307 | 5,78E-10 | 5,40E-08 | UpRegulated |
| A0A3Q2H4A7 | FOLR1 | Uncharacterized protein. (312 aa) | 6,28226965 | 9,0565225 | 6,04E-06 | 0,00011123 | UpRegulated |
| A0A5F5PT31 | FSTL1 | Follistatin like 1. (317 aa) | 1,78535135 | 10,2087873 | 0,00030054 | 0,00237741 | UpRegulated |
| A0A3Q2I240 | FUBP1 | Far upstream element binding protein 1. (687 aa) | 5,31669691 | 8,40843066 | 0,00195014 | 0,01039113 | UpRegulated |
| A0A5F5PNI2 | FUBP1 | Far upstream element binding protein 1. (687 aa) | 5,31669546 | 8,40843066 | 0,00195053 | 0,01039113 | UpRegulated |
| A0A9L0S793 | G3BP1 | G3BP stress granule assembly factor 1. (508 aa) | 5,71183213 | 8,66196271 | 0,00048563 | 0,00344088 | UpRegulated |
| F7DMG5 | G6PD | Glucose-6-phosphate 1-dehydrogenase (555 aa) | 1,55441409 | 10,0471375 | 0,00416857 | 0,0180297 | UpRegulated |
| F7C4H8 | GANAB | Glucosidase II alpha subunit (966 aa) | 2,3376696 | 9,89754752 | 0,0001998 | 0,00180961 | UpRegulated |
| A0A9L0SIZ1 | GARS1 | Glycyl-tRNA synthetase. (739 aa) | 2,66661685 | 9,67885776 | 8,80E-05 | 0,00095498 | UpRegulated |
| A0A9L0RP17 | GCLC | Glutamate-cysteine ligase catalytic subunit. (637 aa) | 5,40012507 | 8,4623686 | 0,00116761 | 0,00685971 | UpRegulated |
| A0A3Q2HND7 | GDI1 | Rab GDP dissociation inhibitor (574 aa) | 2,19041268 | 10,7184175 | 3,88E-08 | 2,03E-06 | UpRegulated |
| A0A5F5Q2Q4 | GDI2 | Rab GDP dissociation inhibitor (441 aa) | 0,87103088 | 10,8088349 | 0,0125531 | 0,04038524 | UpRegulated |
| A0A9L0RI96 | GLOD4 | Glyoxalase domain containing 4 (264aa) | 3,16165715 | 8,68176169 | 0,00253241 | 0,01239824 | UpRegulated |
| A0A9L0R420 | GLRX3 | Glutaredoxin 3. (339 aa) | 5,09688881 | 8,27796737 | 0,00602163 | 0,0239749 | UpRegulated |
| A0A9L0SZQ7 | GNPDA1 | Glucosamine-6-phosphate isomerase. (289 aa) | 3,48740927 | 8,89774522 | 0,00021606 | 0,00190558 | UpRegulated |
| A0A3Q2HTN4 | GNPDA2 | Glucosamine-6-phosphate isomerase. (276 aa) | 2,58888857 | 8,33833594 | 0,01471112 | 0,04519101 | UpRegulated |
| A0A3Q2I189 | GNS | N-acetylglucosamine-6-sulfatase (553 aa) | 5,18092324 | 8,32672453 | 0,00238111 | 0,01182171 | UpRegulated |
| P08907 | GOT2 | Aspartate aminotransferase, mitochondrial). (401 aa) | 5,20363708 | 8,34070324 | 0,00454073 | 0,01928443 | UpRegulated |
| A0A5F5Q0L8 | GOT2 | Aspartate aminotransferase(481 aa) | 5,20369615 | 8,34070324 | 0,00455599 | 0,01929114 | UpRegulated |
| A0A9L0TIL2 | GPI | Glucose-6-phosphate isomerase; Belongs to the GPI family. (573 aa) | 1,35065892 | 10,2865825 | 0,00310166 | 0,01406218 | UpRegulated |
| F7AB03 | GRN | Granulin (594 aa) | 3,04001353 | 10,4296913 | 4,66E-09 | 3,32E-07 | UpRegulated |
| A0A3Q2GVQ5 | GRN | Granulin. (509 aa) | 2,65673201 | 10,1321955 | 1,11E-05 | 0,00016951 | UpRegulated |
| A0A9L0SB09 | GSTM3 | Glutathione S-transferase. (225 aa) | 3,95051982 | 9,22160846 | 9,48E-06 | 0,00015365 | UpRegulated |
| A0A5F5PVD1 | HDGF | Heparin binding growth factor. (240 aa) | 3,7402807 | 9,07281656 | 0,00020021 | 0,00180961 | UpRegulated |
| F6ZN26 | HDGF | Heparin binding growth factor. (254 aa) | 3,38145214 | 8,82892197 | 0,00270693 | 0,01276513 | UpRegulated |
| A0A5F5PPC4 | HEXA | Beta-hexosaminidase. (529 aa) | 4,16951574 | 9,38173469 | 3,51E-06 | 6,97E-05 | UpRegulated |
| F6VE62 | HEXB | Beta-hexosaminidase. (538 aa) | 6,34102667 | 9,09758147 | 1,56E-06 | 3,80E-05 | UpRegulated |
| A0A5F5PY70 | HINT1 | Histidine triad nucleotide binding protein 1. (266 aa) | 5,11379729 | 8,28962713 | 0,00614644 | 0,02420805 | UpRegulated |
| A0A9L0RFB2 | HINT1 | Histidine triad nucleotide binding protein 1. (78 aa) | 4,94199247 | 8,19156404 | 0,00876636 | 0,03130576 | UpRegulated |
| K9K211 | HNRNPA1 | Heterogeneous nuclear ribonucleoprotein A-like protein. (320 aa) | 1,91239023 | 10,5080907 | 1,00E-05 | 0,00015917 | UpRegulated |
| A0A3Q2GW64 | HNRNPA2B1 | Heterogeneous nuclear ribonucleoproteins A2/B1 (535 aa) | 1,14251984 | 10,8415095 | 0,0005091 | 0,00357129 | UpRegulated |
| F6VYB1 | HNRNPA2B1 | Heterogeneous nuclear ribonucleoproteins A2/B1 (301 aa) | 1,11355111 | 10,8222303 | 0,00087526 | 0,0056611 | UpRegulated |
| A0A9L0R8F1 | HNRNPA3 | RRM domain-containing protein. (355 aa) | 5,56576294 | 8,56429126 | 0,00032639 | 0,00251477 | UpRegulated |
| A0A9L0S6M0 | HNRNPA3 | RRM domain-containing protein (291 aa) | 5,61856855 | 8,59885718 | 0,00046105 | 0,00329991 | UpRegulated |
| A0A9L0SF78 | HNRNPAB | Heteroous nuclear ribonucleoprotein A/B. (286 aa) | 2,20996733 | 9,35260334 | 0,00104725 | 0,00642752 | UpRegulated |
| F6Z0C6 | HNRNPD | Heterogeneous nuclear ribonucleoprotein D like. (420 aa) | 3,76454019 | 9,75781003 | 6,27E-07 | 1,92E-05 | UpRegulated |
| F6WHS3 | HNRNPD | Heterogeneous nuclear ribonucleoprotein D like. (420 aa) | 3,72483828 | 9,72736623 | 1,44E-06 | 3,62E-05 | UpRegulated |
| A0A9L0TRS4 | HNRNPDL | Heteroous nuclear ribonucleoprotein D like(301aa) | 6,26695877 | 9,04337663 | 1,87E-06 | 4,06E-05 | UpRegulated |
| A0A3Q2IA51 | HNRNPDL | Heteroous nuclear ribonucleoprotein D like (363aa) | 6,00004783 | 8,85270392 | 8,14E-06 | 0,00013669 | UpRegulated |
| F6X7G5 | HNRNPF | Heterogeneous nuclear ribonucleoprotein F. (398 aa) | 3,84299526 | 9,14394557 | 3,86E-05 | 0,00048166 | UpRegulated |
| F7C113 | HNRNPH1 | Heterogeneous nuclear ribonucleoprotein H1. (472 aa) | 2,19647232 | 9,04556233 | 0,00409554 | 0,0178232 | UpRegulated |
| F7BZZ0 | HNRNPH3 | Heterogeneous nuclear ribonucleoprotein H3. (346 aa) | 4,81746089 | 8,13111254 | 0,007663 | 0,02920224 | UpRegulated |
| A0A9L0TRC2 | HNRNPK | Heterogeneous nuclear ribonucleoprotein K. (464 aa) | 2,05759225 | 10,1653271 | 7,20E-05 | 0,00079278 | UpRegulated |
| F6RTD6 | HNRNPL | Heterogeneous nuclear ribonucleoprotein L. (588 aa) | 6,31348587 | 9,07808947 | 1,71E-06 | 4,00E-05 | UpRegulated |
| F6XAL9 | HNRNPM | Heterogeneous nuclear ribonucleoprotein M. (730 aa) | 2,95759844 | 9,16583578 | 0,00031823 | 0,00248249 | UpRegulated |
| A0A9L0RKL9 | HNRNPM | Heterogeneous nuclear ribonucleoprotein M. (739 aa) | 2,8186482 | 9,07007639 | 0,00069362 | 0,00466551 | UpRegulated |
| A0A9L0R5P0 | HNRNPM | Heterogeneous nuclear ribonucleoprotein M. (757 aa) | 2,81865141 | 9,07007639 | 0,00069486 | 0,00466551 | UpRegulated |
| A0A3Q2LLQ0 | HNRNPR | Heterogeneous nuclear ribonucleoprotein R. (640 aa) | 5,31511466 | 8,40802529 | 0,00225088 | 0,01146761 | UpRegulated |
| F6YZJ4 | HSPA4 | Heat shock protein family A (Hsp70) member 4. (840 aa) | 3,51867184 | 10,0087581 | 2,28E-06 | 4,80E-05 | UpRegulated |
| A0A9L0T7G8 | HSPA9 | Stress-70 protein, mitochondrial (653 aa) | 5,10340413 | 8,28171483 | 0,00508028 | 0,02107486 | UpRegulated |
| A0A3Q2HT74 | HSPG2 | Heparan sulfate proteoglycan 2. (4396 aa) | 1,2484291 | 11,3404138 | 0,00386063 | 0,01695792 | UpRegulated |
| A0A5F5PI06 | HSPH1 | Heat shock protein family H (Hsp110) member 1. (869 aa) | 5,24003067 | 8,36378554 | 0,00449367 | 0,01920021 | UpRegulated |
| A0A9L0RVJ3 | HTRA1 | HtrA serine peptidase 1. (548 aa) | 1,19619348 | 10,2628521 | 0,00874239 | 0,03130576 | UpRegulated |
| A0A3Q2IC55 | HTRA3 | HtrA serine peptidase 3. (493 aa) | 6,58862567 | 9,28148303 | 4,46E-07 | 1,43E-05 | UpRegulated |
| A0A5F5PPR0 | HYOU1 | Hypoxia up-regulated 1. (1000 aa) | 5,47603825 | 8,50463423 | 0,00147856 | 0,00837256 | UpRegulated |
| F7BN14 | IFI30 | IFI30 lysosomal thiol reductase. (295 aa) | 5,11037105 | 8,28838043 | 0,00500651 | 0,02088514 | UpRegulated |
| F7ALY5 | IGFBP5 | Insulin like growth factor binding protein 5. (271 aa) | 5,02448697 | 8,23639635 | 0,00816466 | 0,03030657 | UpRegulated |
| F6RIZ2 | IGFBP7 | Insulin like growth factor binding protein 7. (282 aa) | 1,50502102 | 10,3962002 | 0,00061984 | 0,0042842 | UpRegulated |
| A0A3Q2HNK3 | ILF2 | Interleukin enhancer binding factor 2. (407 aa) | 4,93526805 | 8,18729263 | 0,01084053 | 0,03568381 | UpRegulated |
| A0A5F5PR42 | ILF3 | Interleukin enhancer binding factor 3. (899 aa) | 5,91311844 | 8,79452195 | 4,51E-05 | 0,00054607 | UpRegulated |
| F6T4X6 | IMPA1 | Inositol monophosphatase 1. (414 aa) | 3,48860331 | 8,89907357 | 0,00019861 | 0,00180961 | UpRegulated |
| P55102 | INHBA | Inhibin beta A chain. (426 aa) | 6,66016749 | 9,33411646 | 1,91E-07 | 7,47E-06 | UpRegulated |
| A0A9L0T427 | IPO5 | Importin 5. (1285 aa) | 3,55305451 | 9,59900705 | 7,28E-06 | 0,0001251 | UpRegulated |
| A0A3Q2I982 | IQGAP1 | IQ motif containing GTPase activating protein 1. (1645 aa) | 8,08433948 | 10,4794908 | 8,40E-16 | 1,97E-13 | UpRegulated |
| A0A3Q2HXB2 | IQGAP1 | IQ motif containing GTPase activating protein 1. (1849 aa) | 8,11392711 | 10,5044166 | 1,87E-15 | 3,77E-13 | UpRegulated |
| A0A3Q2HPF0 | ITGA3 | Integrin subunit alpha 3. (1086 aa) | 2,15736492 | 9,02750741 | 0,00697795 | 0,02688228 | UpRegulated |
| F7A984 | KHSRP | KH-type splicing regulatory protein (714 aa) | 6,13506317 | 8,95209759 | 1,73E-05 | 0,00024438 | UpRegulated |
| A0A9L0RR41 | KIF5B | Kinesin-like protein. (964 aa) | 4,93618044 | 8,18643176 | 0,00908624 | 0,03210926 | UpRegulated |
| A0A3Q2HNC5 | KPNB1 | Karyopherin subunit beta 1. (876 aa) | 1,62493144 | 9,20469048 | 0,01662842 | 0,04967387 | UpRegulated |
| A0A5F5Q211 | LAMA2 | Laminin subunit alpha 2. (3094 aa) | 5,38572032 | 8,45092656 | 0,00097834 | 0,00607863 | UpRegulated |
| F7BXV9 | LAMA5 | Laminin subunit alpha 5. (3754 aa) | 6,03957202 | 8,88135077 | 1,32E-05 | 0,00019315 | UpRegulated |
| A0A9L0TN67 | LAMB1 | Laminin subunit beta 1. (1849 aa) | 1,31563881 | 10,6220504 | 0,00061082 | 0,00424265 | UpRegulated |
| A0A3Q2HSI9 | LAMC1 | Laminin subunit gamma 1. (1612 aa) | 0,91884239 | 11,1166954 | 0,00147417 | 0,00837256 | UpRegulated |
| A0A3Q2HRL1 | LAP3 | Leucine aminopeptidase 3. (545 aa) | 6,23546918 | 9,02135251 | 9,32E-06 | 0,00015277 | UpRegulated |
| A0A3Q2H069 | LASP1 | LIM and SH3 domain protein 1. (258 aa) | 5,61518368 | 8,59434539 | 0,00032509 | 0,00251477 | UpRegulated |
| A0A3Q2HWA0 | LASP1 | LIM and SH3 domain protein 1. (307 aa) | 5,24860815 | 8,36540439 | 0,00269352 | 0,01276513 | UpRegulated |
| A0A5F5PT09 | LGMN | Legumain. (433 aa) | 2,87362576 | 8,50556572 | 0,00987608 | 0,03391768 | UpRegulated |
| A0A3Q2KJA1 | LIPG | Lipase G, endothelial type. (521 aa) | 6,19842156 | 8,99471834 | 3,82E-06 | 7,49E-05 | UpRegulated |
| F6Q8G7 | LMAN2 | Lectin, mannose binding 2 (365 aa) | 5,67993608 | 8,63989063 | 0,00019221 | 0,00177934 | UpRegulated |
| A0A3Q2H2I7 | LMAN2 | Lectin, mannose binding 2. (333 aa) | 5,67994591 | 8,63989063 | 0,00019308 | 0,00177934 | UpRegulated |
| F6UCU8 | LMNB1 | Lamin B1 (586 aa) | 2,28987606 | 9,40827585 | 0,00113175 | 0,00678536 | UpRegulated |
| A0A9L0R138 | LOXL1 | Lysyl oxidase like 1. (578 aa) | 5,87137802 | 8,76603142 | 2,82E-05 | 0,00036174 | UpRegulated |
| F6QDE2 | LOXL2 | Lysyl oxidase like 2. (1047 aa) | 6,43435366 | 9,16588735 | 1,50E-06 | 3,72E-05 | UpRegulated |
| A0A9L0SMJ1 | LRRC59 | Uncharacterized protein. (306 aa) | 4,81995245 | 8,13244745 | 0,00825634 | 0,03047496 | UpRegulated |
| A0A3Q2GXC3 | LTBP2 | Leucine rich repeat containing 59(290aa) | 6,51901702 | 9,22769704 | 1,01E-06 | 2,78E-05 | UpRegulated |
| A0A9L0TPQ3 | MANF | Mesencephalic astrocyte derived neurotrophic factor. (183 aa) | 5,66220443 | 8,62690815 | 0,00028884 | 0,00232725 | UpRegulated |
| A0A3Q2LJQ0 | MAP4 | Microtubule-associated protein. (1106 aa) | 3,78568456 | 9,10259187 | 2,81E-05 | 0,00036174 | UpRegulated |
| A0A9L0R1A9 | MARCKS | Myristoylated alanine rich protein kinase C substrate. (336 aa) | 5,2534752 | 8,36985959 | 0,00222021 | 0,01146194 | UpRegulated |
| F6TYW4 | MDH2 | Malate dehydrogenase. (338 aa) | 3,88178129 | 9,17040293 | 1,80E-05 | 0,00024971 | UpRegulated |
| A0A3Q2I7G4 | MINPP1 | Multiple inositol-polyphosphate phosphatase 1 (493 aa) | 5,38564305 | 8,45211724 | 0,00285386 | 0,01328035 | UpRegulated |
| Q9XSZ5 | MMP1 | Interstitial collagenase. (520 aa) | 0,8082929 | 10,9273224 | 0,01034322 | 0,03480653 | UpRegulated |
| A0A9L0RNS2 | MMP14 | Matrix metallopeptidase 14. (581 aa) | 0,87360571 | 10,7109444 | 0,01361403 | 0,04265729 | UpRegulated |
| A0A3Q2I1G5 | MMP9 | Matrix metallopeptidase 9. (714 aa) | 4,98362813 | 10,0140381 | 6,13E-10 | 5,40E-08 | UpRegulated |
| A0A3Q2GRT7 | MVP | Major vault protein. (854 aa) | 5,21156621 | 8,33823568 | 0,00360752 | 0,016093 | UpRegulated |
| A0A3Q2LCX5 | MVP | Major vault protein. (895aa) | 5,21158148 | 8,33823568 | 0,00361807 | 0,016093 | UpRegulated |
| F6PJV0 | MXRA5 | Matrix remodeling associated 5. (2851 aa) | 5,83818354 | 8,75519106 | 0,00369481 | 0,01638264 | UpRegulated |
| F6UG31 | MXRA8 | Matrix remodeling-associated protein 8. (703 aa) | 2,9412007 | 8,5493879 | 0,00997408 | 0,03399024 | UpRegulated |
| A0A3Q2GSU0 | MYOF | Myoferlin. (2121 aa) | 2,9995262 | 8,58390095 | 0,00639831 | 0,02506006 | UpRegulated |
| A0A3Q2KYJ2 | NAMPT | Nicotinamide phosphoribosyltransferase. (534 aa) | 5,32000253 | 8,41002628 | 0,00154992 | 0,00860388 | UpRegulated |
| F6YM24 | NANS | N-acetylneuraminate synthase. (425 aa) | 6,04597443 | 8,88742034 | 9,85E-06 | 0,0001578 | UpRegulated |
| F7DE75 | NAPA | NSF attachment protein alpha. (388 aa) | 5,77985459 | 8,70582098 | 9,53E-05 | 0,00102597 | UpRegulated |
| A0A5F5Q2P9 | NAPA | NSF attachment protein alpha. (256 aa) | 5,5129085 | 8,53296868 | 0,0004192 | 0,00304673 | UpRegulated |
| A0A3Q2GV25 | NARS1 | Asparaginyl-tRNA synthetase. (559 aa) | 5,17786102 | 8,32467193 | 0,00290672 | 0,01335569 | UpRegulated |
| A0A3Q2LJE9 | NCSTN | Nicastrin. (709 aa) | 4,77094493 | 8,09532014 | 0,01307664 | 0,04143384 | UpRegulated |
| A0A9L0RW21 | NDRG1 | N-myc downstream regulated 1. (384 aa) | 5,11222131 | 8,28713412 | 0,01081148 | 0,03568381 | UpRegulated |
| A0A3Q2HZZ8 | NIBAN2 | Niban apoptosis regulator 2. (848 aa) | 3,16359838 | 8,68467088 | 0,0029002 | 0,01335569 | UpRegulated |
| A0A9L0SQS5 | NID1 | Nidogen 1. (1244 aa) | 2,74617288 | 9,02303569 | 0,00110271 | 0,00664456 | UpRegulated |
| A0A9L0RSS8 | NONO | Non-POU domain containing octamer binding. (470 aa) | 6,19952206 | 8,99561145 | 1,21E-05 | 0,00018187 | UpRegulated |
| A0A5F5PJE0 | NPEPPS | Aminopeptidase. (921 aa) | 1,50224884 | 9,89642791 | 0,01006822 | 0,03404362 | UpRegulated |
| A0A3Q2KN19 | NUCB1 | Nucleobindin 1. (457 aa) | 2,28043351 | 9,64725212 | 0,00063242 | 0,00434979 | UpRegulated |
| A0A9L0R2V1 | NUDT21 | Cleavage and polyadenylation specificity factor subunit 5 (256 aa) | 4,54210264 | 7,9819586 | 0,01091375 | 0,03570392 | UpRegulated |
| A0A9L0SBX0 | OLA1 | Glyoxalase domain containing 4. (449 aa) | 4,42223402 | 7,92364652 | 0,01673492 | 0,04977148 | UpRegulated |
| P00761 | P00761 | **Trypsin(231aa)** | 7,40555684 | 9,95511847 | 0,00652557 | 0,02541729 | UpRegulated |
| F7CMH2 | PA2G4 | Proliferation-associated 2G4. (387 aa) | 5,36373404 | 8,44106863 | 0,00337576 | 0,01525584 | UpRegulated |
| A0A3Q2I1M9 | PABPC1 | Polyadenylate-binding protein (636 aa) | 6,36625448 | 9,11564625 | 6,28E-06 | 0,0001121 | UpRegulated |
| A0A3Q2GZ77 | PABPC4 | Polyadenylate-binding protein. (636 aa) | 5,39343521 | 8,45566836 | 0,00260551 | 0,01253848 | UpRegulated |
| A0A3Q2GUP3 | PAFAH1B1 | Platelet-activating factor acetylhydrolase IB subunit alpha (413 aa) | 5,3200104 | 8,41002628 | 0,00154776 | 0,00860388 | UpRegulated |
| A0A9L0S4E3 | PAICS | PurE domain-containing protein. (384 aa) | 5,57533479 | 8,57032316 | 0,00106862 | 0,00652272 | UpRegulated |
| A0A9L0RT12 | PAICS | PurE domain-containing protein. (504 aa) | 5,25710032 | 8,37431693 | 0,00292515 | 0,01335569 | UpRegulated |
| F6UNF7 | PAPPA | Pappalysin 1. (1627 aa) | 5,18364262 | 8,33124476 | 0,0022968 | 0,01156604 | UpRegulated |
| A0A3Q2I1N3 | PARVA | Parvin alpha. (459 aa) | 5,5714161 | 8,57072279 | 0,00024211 | 0,00201999 | UpRegulated |
| A0A9L0SKS1 | PCBP1 | Poly(rC) binding protein 1(356aa) | 1,86197624 | 9,1214672 | 0,01240553 | 0,040027 | UpRegulated |
| A0A3Q2H8C6 | PCBP2 | K Homology domain-containing protein (467 aa) | 2,66897365 | 8,97109453 | 0,00232801 | 0,01167352 | UpRegulated |
| A0A3Q2H024 | PCBP2 | Poly(rC) binding protein 2. (366 aa) | 2,66899054 | 8,97109453 | 0,00233471 | 0,01167352 | UpRegulated |
| F6WZ69 | PCOLCE | Procollagen C-endopeptidase enhancer. (495 aa) | 1,36962835 | 10,300253 | 0,00508188 | 0,02107486 | UpRegulated |
| A0A3Q2H267 | PCSK5 | Proprotein convertase subtilisin/kexin type 5 (865 aa) | 6,73784479 | 9,39315821 | 7,86E-08 | 3,58E-06 | UpRegulated |
| A0A9L0RZ59 | PDCD6 | Programmed cell death 6. (176 aa) | 5,55100224 | 8,55555801 | 0,00087221 | 0,0056611 | UpRegulated |
| A0A3Q2HWB5 | PDCD6IP | Programmed cell death 6 interacting protein. (907 aa) | 5,67947392 | 8,63949471 | 0,00018112 | 0,00172551 | UpRegulated |
| A0A3Q2HGW8 | PDLIM5 | PDZ and LIM domain 5. (651 aa) | 1,92488457 | 9,93208886 | 0,00114936 | 0,00680945 | UpRegulated |
| A0A5F5PZA2 | PFKP | ATP-dependent 6-phosphofructokinase (851 aa) | 5,62743701 | 8,60560256 | 0,00036795 | 0,00276043 | UpRegulated |
| A0A3Q2H4G7 | PFKP | ATP-dependent 6-phosphofructokinase (842 aa) | 5,62745331 | 8,60560256 | 0,00037002 | 0,00276043 | UpRegulated |
| A0A3Q2GWI8 | PGM2 | Phosphoglucomutase 2. (619 aa) | 2,21039018 | 10,1426664 | 0,00018004 | 0,00172551 | UpRegulated |
| A0A9L0RJ03 | PGM3 | Phosphoacetylglucosamine mutase (542aa) | 5,1853806 | 8,33206715 | 0,00278584 | 0,01305643 | UpRegulated |
| A0A5F5PL45 | PITPNB | Phosphatidylinositol transfer protein beta. (273 aa) | 5,56563701 | 8,56238461 | 0,00023186 | 0,0020031 | UpRegulated |
| A0A3Q2IDK5 | PLAT | Plasminogen activator, tissue type. (615 aa) | 5,49547218 | 8,51990067 | 0,00083807 | 0,00549615 | UpRegulated |
| A0A9L0TAU7 | PLAU | Plasminogen activator, urokinase. (433 aa) | 4,8769315 | 9,93052338 | 4,14E-09 | 3,25E-07 | UpRegulated |
| A0A9L0S640 | PLBD2 | Phospholipase B-like (533 aa) | 2,74158822 | 9,42536807 | 0,00014115 | 0,00142089 | UpRegulated |
| A0A5F5PQE5 | PLEC | Plectin. (4689 aa) | 2,92751464 | 11,7183077 | 1,76E-17 | 4,98E-15 | UpRegulated |
| A0A9L0RV37 | PLIN3 | Perilipin 3. (540 aa) | 5,4027002 | 8,46117613 | 0,00186281 | 0,01002502 | UpRegulated |
| A0A9L0T8Q4 | PLOD1 | Procollagen-lysine,2-oxoglutarate 5-dioxygenase 1. (727 aa) | 2,10341147 | 9,72634019 | 0,0025599 | 0,01241392 | UpRegulated |
| A0A5F5PVY9 | PLOD2 | Procollagen-lysine,2-oxoglutarate 5-dioxygenase 2. (758 aa) | 4,0787289 | 10,0075446 | 7,02E-08 | 3,38E-06 | UpRegulated |
| F7B3H1 | PLOD3 | procollagen-lysine 5-dioxygenase 3 (764 aa) | 2,06788484 | 10,3047815 | 3,63E-05 | 0,00045655 | UpRegulated |
| A0A9L0SEX4 | PLOD3 | Procollagen-lysine,2-oxoglutarate 5-dioxygenase 3. (694 aa) | 2,19645399 | 10,1295817 | 4,68E-05 | 0,00055927 | UpRegulated |
| A0A9L0SZ69 | PLS3 | Plastin 3. (639 aa) | 0,79709458 | 11,002132 | 0,00906135 | 0,03210177 | UpRegulated |
| P62937 | PPIA | Peptidyl-prolyl cis-trans isomerase A (165aa) | 5,96730959 | 8,87541851 | 0,01450102 | 0,04483869 | UpRegulated |
| F6R8R0 | PPP1CA | Serine/threonine-protein phosphatase (330 aa) | 2,3724519 | 9,16426436 | 0,00255752 | 0,01241392 | UpRegulated |
| A0A3Q2HEA5 | PPP1CB | Serine/threonine-protein phosphatase(318aa) | 2,28972421 | 9,11094733 | 0,00223748 | 0,01146761 | UpRegulated |
| A0A9L0S9S2 | PPP1CC | Serine/threonine-protein phosphatase (389 aa) | 2,10168164 | 8,9869353 | 0,00775867 | 0,02940666 | UpRegulated |
| A0A9L0STS1 | PPP2CA | Serine/threonine-protein phosphatase(302aa) | 3,53845137 | 8,93404058 | 0,00014532 | 0,00144293 | UpRegulated |
| A0A9L0S645 | PPP2CB | Serine/threonine-protein phosphatase. (309 aa) | 3,49941388 | 8,90729399 | 0,00026849 | 0,00220101 | UpRegulated |
| A0A9L0SKM1 | PPP2R1A | Protein phosphatase 2 scaffold subunit Aalpha. (538 aa) | 1,60526142 | 9,70724746 | 0,00611308 | 0,02417343 | UpRegulated |
| F6UJZ8 | PRCP | Prolylcarboxypeptidase. (497 aa) | 2,12937297 | 8,62838897 | 0,01686777 | 0,04993526 | UpRegulated |
| A0A9L0T6K3 | PRDX5 | Peroxiredoxin. (255 aa) | 5,10808369 | 8,28587519 | 0,00576503 | 0,02342563 | UpRegulated |
| A0A9L0TGT9 | PRDX5 | Peroxiredoxin. (244 aa) | 4,78148061 | 8,1001919 | 0,00579352 | 0,02347373 | UpRegulated |
| A0A3Q2H1I0 | PRMT1 | Type I protein arginine methyltransferase (347 aa) | 6,68884865 | 9,35611637 | 2,11E-07 | 8,03E-06 | UpRegulated |
| A0A3Q2HFB0 | PRMT1 | Protein arginine methyltransferase 1 (343aa) | 6,87830099 | 9,50209994 | 4,00E-07 | 1,34E-05 | UpRegulated |
| A0A3Q2I2H4 | PSAT1 | Phosphoserine transaminase. (324 aa) | 5,44037533 | 8,48630242 | 0,00169477 | 0,0093007 | UpRegulated |
| A0A3Q2I723 | PSAT1 | Phosphoserine aminotransferase. (375 aa) | 2,7405271 | 8,42693027 | 0,00936412 | 0,03284429 | UpRegulated |
| F6TCI4 | PSMA2 | Proteasome subunit alpha type. (234 aa) | 2,2040839 | 8,67572308 | 0,01536322 | 0,04662115 | UpRegulated |
| A0A3Q2HJF4 | PSMA6 | Proteasome subunit alpha type. (203 aa) | 5,10880881 | 8,2875453 | 0,00453183 | 0,01928443 | UpRegulated |
| A0A3Q2LTZ2 | PSMA6 | Proteasome 20S subunit alpha 6. (235 aa) | 4,94313056 | 8,19328715 | 0,00770314 | 0,02927607 | UpRegulated |
| F6YYB1 | PSMB1 | Proteasome subunit beta 1. (406 aa) | 5,56563323 | 8,56238461 | 0,00023201 | 0,0020031 | UpRegulated |
| F6R449 | PSMC3 | Proteasome 26S subunit, ATPase 3 (450aa) | 6,10952073 | 8,93039962 | 1,14E-05 | 0,00017321 | UpRegulated |
| A0A5F5PHN9 | PSMD13 | Proteasome 26S subunit, non-ATPase 13. (394 aa) | 4,84066391 | 8,13806147 | 0,00612051 | 0,02417343 | UpRegulated |
| A0A3Q2I636 | PSMD13 | 26S proteasome non-ATPase regulatory subunit 13. (349 aa) | 5,02227818 | 8,23680825 | 0,01342403 | 0,04215565 | UpRegulated |
| F6UQI3 | PSMD2 | 26S proteasome non-ATPase regulatory subunit 2. (904 aa) | 6,01242286 | 8,86468977 | 1,82E-05 | 0,00024971 | UpRegulated |
| A0A3Q2H5S0 | PSMD2 | 26S proteasome non-ATPase regulatory subunit 2 (820 aa) | 5,58012575 | 8,57227656 | 0,00116457 | 0,00685971 | UpRegulated |
| A0A5F5PTM2 | PSMD5 | 26S proteasome non-ATPase regulatory subunit 5 (461 aa) | 3,56397002 | 8,94948968 | 0,00021069 | 0,00188019 | UpRegulated |
| A0A5F5PW11 | PSMD6 | 26S proteasome non-ATPase regulatory subunit 6 (441 aa) | 5,03740271 | 8,24441855 | 0,01273777 | 0,04054233 | UpRegulated |
| A0A3Q2IFM4 | PSME1 | Proteasome activator complex subunit 1. (237 aa) | 6,03954311 | 8,88135077 | 1,33E-05 | 0,00019315 | UpRegulated |
| A0A5F5PV20 | PSME2 | Proteasome activator complex subunit 2(228aa) | 4,9556489 | 8,19842567 | 0,01267521 | 0,04052618 | UpRegulated |
| A0A5F5PUI6 | PTBP1 | Polypyrimidine tract binding protein 1. (591 aa) | 2,95729549 | 10,1523342 | 4,29E-07 | 1,41E-05 | UpRegulated |
| A0A9L0S2F1 | PTGR1 | Prostaglandin reductase 1. (329 aa) | 2,40287598 | 9,73844944 | 0,00019198 | 0,00177934 | UpRegulated |
| A0A9L0R3M3 | PTK7 | Protein tyrosine kinase 7 (inactive); (1079 aa) | 4,87616435 | 9,93182096 | 1,65E-08 | 9,69E-07 | UpRegulated |
| A0A3Q2H8R9 | PUF60 | Poly(U) binding splicing factor 60. (561 aa) | 4,74896468 | 8,08825701 | 0,00895483 | 0,0318043 | UpRegulated |
| A0A3Q2HNE1 | PXDN | Peroxidasin. (1596 aa) | 1,57401278 | 11,6439743 | 1,83E-06 | 4,06E-05 | UpRegulated |
| A0A9L0RP87 | QSOX1 | Sulfhydryl oxidase. (752 aa) | 0,90361746 | 10,6872863 | 0,01635918 | 0,04897334 | UpRegulated |
| A0A3Q2I053 | RAB11A | RAB11A, member RAS oncogene family. (216 aa) | 2,67020191 | 8,38869515 | 0,01149042 | 0,03740886 | UpRegulated |
| A0A3Q2H195 | RAB7A | RAB7A, member RAS oncogene family. (229 aa) | 2,27635723 | 9,40200155 | 0,00265394 | 0,01269415 | UpRegulated |
| H9H002 | RANBP1 | RAN binding protein 1. (278 aa) | 4,85802423 | 8,14764707 | 0,01553745 | 0,04699923 | UpRegulated |
| F6YPN7 | RARS1 | Arginyl-tRNA synthetase. (658 aa) | 5,51644113 | 8,53526268 | 0,00056159 | 0,00392 | UpRegulated |
| A0A9L0SCE5 | RBM3 | RNA binding motif protein 3. (156 aa) | 5,00602408 | 8,23005362 | 0,01537506 | 0,04662115 | UpRegulated |
| F6XEW8 | RBM8A | RNA-binding protein 8A. (179 aa) | 5,02624894 | 8,23850391 | 0,00798435 | 0,03003433 | UpRegulated |
| W0UVF5 | RNASE4 | Ribonuclease 4 (147 aa) | 2,31069357 | 9,12548518 | 0,00372683 | 0,01647281 | UpRegulated |
| A0A3Q2GW51 | RNH1 | Ribonuclease inhibitor (683 aa) | 7,13883392 | 9,70478991 | 4,70E-09 | 3,32E-07 | UpRegulated |
| A0A9L0S668 | RNH1 | Ribonuclease inhibitor (451 aa) | 6,97838231 | 9,57877698 | 2,23E-08 | 1,26E-06 | UpRegulated |
| A0A9L0TBX2 | RPL10A | Ribosomal protein; (224 aa) | 5,25669728 | 8,37472501 | 0,00257083 | 0,01241392 | UpRegulated |
| A0A5K1VP18 | RPL5 | Ribosomal protein L5. (297 aa) | 5,38571932 | 8,45092656 | 0,00097862 | 0,00607863 | UpRegulated |
| F7DFM3 | RPS4X | 40S ribosomal protein S4 (263 aa) | 5,7839719 | 8,7097874 | 0,00010329 | 0,00109506 | UpRegulated |
| A0A3Q2HNK9 | RRBP1 | Ribosome binding protein 1. (1577 aa) | 4,01211918 | 9,27732363 | 0,00032878 | 0,00251942 | UpRegulated |
| A0A9L0RKT7 | RTRAF | RNA transcription, translation and transport factor. (248 aa) | 5,41801269 | 8,47775152 | 0,00289291 | 0,01335569 | UpRegulated |
| F7BTP3 | SARS1 | Seryl-tRNA synthetase. (538 aa) | 6,64006976 | 9,31972838 | 2,86E-07 | 1,03E-05 | UpRegulated |
| A0A9L0R1Y5 | SCARB2 | Scavenger receptor class B member 2. (441 aa) | 5,91498067 | 8,79598242 | 4,01E-05 | 0,00049485 | UpRegulated |
| A0A3Q2HE38 | SCARB2 | Scavenger receptor class B member 2. (479 aa) | 5,91496765 | 8,79598242 | 4,04E-05 | 0,00049485 | UpRegulated |
| A0A3Q2H6B6 | SCPEP1 | Carboxypeptidase. (455 aa) | 5,18377935 | 8,33124476 | 0,00236005 | 0,01175853 | UpRegulated |
| F7CW51 | SDCBP | Syndecan binding protein. (299 aa) | 1,13039437 | 10,4341 | 0,01068852 | 0,03568381 | UpRegulated |
| A0A5F5PI19 | SEC22B | SEC22 homolog B, vesicle trafficking protein. (218 aa) | 5,51961839 | 8,53602737 | 0,00081183 | 0,00534894 | UpRegulated |
| A0A3Q2H5Z6 | SEC23A | Protein transport protein SEC23. (765 aa) | 2,3150986 | 9,88098161 | 0,00031868 | 0,00248249 | UpRegulated |
| F6Q3I9 | SEC31A | Protein transport protein Sec31A (1252aa) | 3,11202988 | 8,65375737 | 0,00525309 | 0,02165747 | UpRegulated |
| A0A5F5Q068 | SEPHS1 | Selenophosphate synthetase 1. (392 aa) | 5,32962345 | 8,4184281 | 0,00195294 | 0,01039113 | UpRegulated |
| A0A3Q2HEH6 | SEPTIN11 | Septin 11 (438 aa) | 2,62558694 | 9,65216228 | 0,00017334 | 0,00167403 | UpRegulated |
| A0A3Q2H0H9 | SEPTIN6 | Septin 6. (482 aa) | 3,30088007 | 8,76937416 | 0,00127754 | 0,00736619 | UpRegulated |
| A0A9L0TQH4 | SEPTIN7 | Septin 7 (423 aa) | 3,47695314 | 9,54072922 | 4,72E-06 | 8,99E-05 | UpRegulated |
| A0A5F5PNV6 | SEPTIN8 | Septin 8 (431 aa) | 5,3201706 | 8,41002628 | 0,00150474 | 0,00841937 | UpRegulated |
| A0A5F5PH65 | SEPTIN9 | Septin 9. (578 aa) | 5,67596823 | 8,63542996 | 0,00013525 | 0,00138032 | UpRegulated |
| P05619 | SERPINB1 | Leukocyte elastase inhibitor (379 aa) | 5,57246221 | 8,57263086 | 0,00023244 | 0,0020031 | UpRegulated |
| A0A3Q2LD63 | SERPINB8 | Serpin family B member 8 (415 aa) | 6,27268949 | 9,04845129 | 1,87E-06 | 4,06E-05 | UpRegulated |
| A0A9L0SGR6 | SERPINB8 | Serpin family B member 8 (243 aa) | 5,25441398 | 8,37188688 | 0,00217832 | 0,01137565 | UpRegulated |
| A0A9L0RGU0 | SERPINB9 | Serpin family B member 9. (381 aa) | 3,19262227 | 9,33479596 | 5,50E-05 | 0,00063458 | UpRegulated |
| F7DDC0 | SERPINE1 | Plasminogen activator inhibitor 1 (395 aa) | 1,01711003 | 11,5709992 | 0,00068038 | 0,00462415 | UpRegulated |
| F7CZW9 | SERPING1 | Serpin family G member 1 (568 aa) | 5,68903775 | 8,64582099 | 0,00027897 | 0,00227366 | UpRegulated |
| A0A3Q2H6E4 | SFPQ | Splicing factor proline and glutamine rich. (708 aa) | 6,48995414 | 9,20617413 | 1,02E-06 | 2,78E-05 | UpRegulated |
| A0A9L0QZG5 | SGTA | Small glutamine rich tetratricopeptide repeat co-chaperone alpha (332 aa) | 4,51878845 | 7,97376674 | 0,01559975 | 0,04699923 | UpRegulated |
| A0A3Q2H0Y1 | SLC39A14 | Solute carrier family 39 member 14. (532 aa) | 4,65995258 | 8,04045645 | 0,00816773 | 0,03030657 | UpRegulated |
| A0A3Q2HXV0 | SLC44A1 | Choline transporter-like protein. (627 aa) | 5,1024594 | 8,28212519 | 0,0064455 | 0,02517493 | UpRegulated |
| F6PH25 | SMPD1 | Sphingomyelin phosphodiesterase 1. (824 aa) | 4,54210578 | 7,9819586 | 0,0108823 | 0,03568381 | UpRegulated |
| A0A3Q2HPW2 | SND1 | Staphylococcal nuclease domain-containing protein; (925 aa) | 6,63298096 | 9,31395753 | 6,44E-07 | 1,93E-05 | UpRegulated |
| A0A9L0SSG2 | SNRPD3 | Small nuclear ribonucleoprotein Sm D3. (120 aa) | 5,17817043 | 8,32467193 | 0,00280385 | 0,01309084 | UpRegulated |
| A0A3Q2GWK7 | SNX9 | Sorting nexin. (621 aa) | 5,74293008 | 8,68703473 | 0,00049263 | 0,003473 | UpRegulated |
| A0A9L0S7T2 | SOD2 | Superoxide dismutase (232 aa) | 6,232113 | 9,01810447 | 2,58E-06 | 5,35E-05 | UpRegulated |
| A0A3Q2GZL9 | SPTAN1 | Spectrin alpha, non-erythrocytic 1. (2489aa) | 3,76145368 | 9,76042911 | 6,15E-06 | 0,00011123 | UpRegulated |
| F6UPM7 | SPTAN1 | Spectrin alpha, non-erythrocytic 1. (2463aa) | 3,79669113 | 9,78799585 | 6,15E-06 | 0,00011123 | UpRegulated |
| F6UPN4 | SPTAN1 | Spectrin alpha, non-erythrocytic 1. (2464 aa) | 3,79723543 | 9,7884293 | 6,97E-06 | 0,00012137 | UpRegulated |
| F6TH64 | SPTBN1 | Spectrin beta chain (2331 aa) | 6,35182968 | 9,11825796 | 5,54E-05 | 0,00063458 | UpRegulated |
| A0A9L0SSS3 | SPTBN1 | Spectrin beta chain (2162 aa) | 6,21216338 | 9,02021973 | 0,00016103 | 0,00156591 | UpRegulated |
| A0A3Q2HYH6 | SRI | Sorcin. (216 aa) | 5,25631754 | 8,37553521 | 0,00225744 | 0,01146761 | UpRegulated |
| A0A5F5PKJ8 | STAM | Signal transducing adaptor molecule. (727 aa) | 2,74409429 | 8,43088566 | 0,01044938 | 0,03508005 | UpRegulated |
| A0A9L0R8G6 | STC1 | Stanniocalcin 1. (391 aa) | 4,94086133 | 8,1902891 | 0,00841713 | 0,03058801 | UpRegulated |
| A0A3Q2H857 | SYNCRIP | Synaptotagmin binding cytoplasmic RNA interacting protein. (623 aa) | 3,35212787 | 8,80349149 | 0,00094503 | 0,00600224 | UpRegulated |
| A0A5F5PF23 | TALDO1 | Transaldolase. (318 aa) | 2,21330277 | 9,06250016 | 0,00595621 | 0,02391513 | UpRegulated |
| F7BA40 | TALDO1 | Transaldolase (309 aa) | 2,14144388 | 9,01549294 | 0,00676869 | 0,0262916 | UpRegulated |
| A0A9L0SZP2 | TARS1 | Threonyl-tRNA synthetase. (722 aa) | 6,70265949 | 9,36519951 | 1,16E-06 | 3,03E-05 | UpRegulated |
| A0A3Q2HLJ5 | TCP1 | T-complex 1. (556 aa) | 2,30224667 | 8,73477384 | 0,01074376 | 0,03568381 | UpRegulated |
| A0A3Q2HNR5 | TFG | Trafficking from ER to golgi regulator. (424 aa) | 4,94730212 | 8,19456295 | 0,01143455 | 0,0373211 | UpRegulated |
| F6UMQ4 | TGFBI | Transforming growth factor-beta-induced protein ig-h3. (734 aa) | 0,78353241 | 11,7841959 | 0,00829696 | 0,03051719 | UpRegulated |
| A0A9L0TP23 | THOP1 | Thimet oligopeptidase 1. (695 aa) | 5,27501297 | 8,37958675 | 0,00969227 | 0,03341346 | UpRegulated |
| A0A3Q2HD92 | TNC | Tenascin C. (2293 aa) | 6,27164507 | 11,0938774 | 1,66E-24 | 2,25E-21 | UpRegulated |
| A0A3Q2I129 | TRIM28 | Tripartite motif containing 28 (832 aa) | 5,11324121 | 8,29046249 | 0,00530525 | 0,02180875 | UpRegulated |
| A0A3Q2IE78 | TRIM28 | Tripartite motif containing 28. (751 aa) | 4,95562607 | 8,19842567 | 0,0126565 | 0,04052618 | UpRegulated |
| A0A9L0R156 | TSN | Translin. (218 aa) | 4,94199024 | 8,19156404 | 0,00871711 | 0,03130576 | UpRegulated |
| F6Y1E7 | TWF1 | Twinfilin actin binding protein 1. (384 aa) | 5,83382275 | 8,74397707 | 5,80E-05 | 0,00065473 | UpRegulated |
| A0A9L0RY71 | TXNDC17 | Thioredoxin domain containing 17. (159 aa) | 2,38993067 | 8,78767216 | 0,00434491 | 0,01867783 | UpRegulated |
| A0A3Q2GX96 | TXNL1 | Thioredoxin like 1. (303 aa) | 5,18538364 | 8,33206715 | 0,00278722 | 0,01305643 | UpRegulated |
| A0A9L0R5N5 | U2AF2 | Splicing factor U2AF subunit (456 aa) | 2,8099642 | 8,46897695 | 0,00954942 | 0,03308277 | UpRegulated |
| A0A9L0SGW6 | UBA1 | E1 ubiquitin-activating enzyme (1018 aa) | 1,52985395 | 9,92111426 | 0,00923967 | 0,03256982 | UpRegulated |
| A0A3Q2GVL4 | UCHL1 | Ubiquitin carboxyl-terminal hydrolase isozyme L1 (281 aa) | 1,77729848 | 9,67270753 | 0,00727691 | 0,02780607 | UpRegulated |
| A0A3Q2HZR9 | UGDH | UDP-glucose 6-dehydrogenase (503 aa) | 4,55121468 | 7,98605977 | 0,0131449 | 0,04155674 | UpRegulated |
| A0A3Q2H1L9 | VAPA | VAMP associated protein A. (317 aa) | 5,51169426 | 8,53063721 | 0,00119976 | 0,00701933 | UpRegulated |
| F6VZY5 | VASP | Vasodilator stimulated phosphoprotein (385 aa) | 5,60904937 | 8,5969229 | 0,00210519 | 0,0111098 | UpRegulated |
| F6XC16 | VAT1 | Vesicle amine transport 1. (477 aa) | 1,46829079 | 9,7540811 | 0,01563368 | 0,04700105 | UpRegulated |
| A0A3Q2HWP9 | VCP | Valosin containing protein. (822 aa) | 0,66291067 | 11,4933001 | 0,01179075 | 0,0382183 | UpRegulated |
| A0A3Q2IA99 | VDAC1 | Non-selective voltage-gated ion channel VDAC1 (306 aa) | 5,77672213 | 8,70256976 | 9,98E-05 | 0,0010662 | UpRegulated |
| A0A9L0R7V3 | VDAC1 | Non-selective voltage-gated ion channel VDAC1. (296 aa) | 5,18286742 | 8,32960012 | 0,00265587 | 0,01269415 | UpRegulated |
| A0A9L0TPG2 | VPS26A | VPS26, retromer complex component A. (327 aa) | 5,39878444 | 8,4615816 | 0,00113571 | 0,00678536 | UpRegulated |
| A0A3Q2HL64 | VPS29 | Vacuolar protein sorting-associated protein 29 (186 aa) | 5,33633014 | 8,42202925 | 0,00229022 | 0,01156604 | UpRegulated |
| F6RC09 | VPS35 | Vacuolar protein sorting-associated protein 35 (796 aa) | 6,38917161 | 9,13550866 | 1,05E-05 | 0,00016524 | UpRegulated |
| A0A9L0R368 | WARS1 | Tryptophanyl-tRNA synthetase (466 aa) | 0,82349468 | 10,9000293 | 0,0150578 | 0,0460553 | UpRegulated |
| F6S9J8 | YARS1 | Tyrosine--tRNA ligase. (774 aa) | 5,22910712 | 8,36135434 | 0,00716153 | 0,02743957 | UpRegulated |
