# Supplementary material for: Bidirectional Interaction Between PGE2-Preconditioned Mesenchymal Stem Cells and Myofibroblasts Mediates Anti-Fibrotic Effects: A Proteomic Investigation into Equine Endometrial Fibrosis Reversal
Source: Proteomes. 2025 Sep 8;13(3):41. doi: 10.3390/proteomes13030041 (PMC12452512; doi:10.3390/proteomes13030041)
Supplement: Supplementary file 1 [file proteomes-13-00041-s001.zip › proteomes-3748745-supplementary-8.22/Table supplementary 3 and 4 cluster.docx]

| Proteome (Myofibroblasts) Down-regulated proteins 48hours VS 0 hours | | | | |
| --- | --- | --- | --- | --- |
| **Cluster number** | **Color cluster** | **Gene count** | **Primary description** | **Protein names** |
| 1 | Red | 9 | Collagen degradation | CXCL6, COL1A1, MXRA5, COL3A1, THBS2, MMP1, COL2A1, COL5A1, COL1A2 |
| 2 | Brown | 5 | aminoacyl-tRNA editing activity | ASNS, ALDH1L2, AARS1, IARS1, SRPRA |
| 3 | Dark Golden Rod | 4 | Interleukin-4 and Interleukin-13 signaling | FOXO1, PXDN, STAT3, JUNB |
| 4 | Green Yellow | 4 | - | RRS1, DDX21, UPF1, DNAJC2 |
| 5 | Green 2 | 3 | U2 snRNP, and ATP-dependent RNA helicase DEAD-box, conserved site | SF3B2, DDX42, SNW1 |
| 6 | Green | 2 | - | MOGS, TMEM214 |
| 7 | Blue | 2 | - | AP3D1, COPA |
| 8 | Light Sky Blue | 2 | - | PDLIM2, LRRC40 |
| 9 | Medium Blue | 2 | A tetrasaccharide linker sequence is required for GAG synthesis | HSPG2, VCAN |
| 10 | Purple | 1 | - | ARFGAP1 |

Table S3 Most significant GO pathways enriched in Myofibroblasts clusters

| Proteome (Myofibroblasts) Up-regulated proteins 48hours *VS* 0 hours | | | | |
| --- | --- | --- | --- | --- |
| **Cluster number** | **Color cluster** | **Gene count** | **Primary description** | **Protein names** |
| 1 | Red | 5 | Amyloid fibril formation | CLU, GSN, ANXA5, APP, ITM2B |
| 2 | Brown | 4 | Insulin growth factor-binding protein homologues | PLAT, CCN2, IGFBP7, PTGS2 |
| 3 | Dark Golden Rod | 3 | - | GJA1, IGF2R, SLC38A4 |
| 4 | Green Yellow | 3 | Von Willebrand factor (vWF) type C domain | THBS1, CCN1, ITGB5 |
| 5 | Green 2 | 3 | Regulation of IFNG signaling | IFNGR1, JAK1, PDGFRA |
| 6 | Green | 3 | Transcriptional activation of mitochondrial biogenesis | ACSS2, GLUD1, FASN |
| 7 | Blue | 3 | - | ATP5F1B, AK3, UQCRC2 |
| 8 | Light Sky Blue | 2 | - | MMP14, LUM |
| 9 | Medium Blue | 2 | - | AXL, GPNMB |
| 10 | Purple | 2 | - | EDIL3, AGA |

Table S4 Most significant GO pathways enriched in Secretome clusters

| Secretome (supernatant samples after co-culture for 48 hours compared to 0 hours) Down-regulated | | | | | | | |
| --- | --- | --- | --- | --- | --- | --- | --- |
| **Cluster number** | | **Color cluster** | **Gene count** | | **Primary description** | **Protein names** | |
| 1 | | Red | 34 | | Mixed, incl. Complement and coagulation cascades, and Lipoprotein particle | F5, KNG1, ALB, PLG, SERPINC1, LOC100061763, AHSG, F2, LOC100059239, APOB, VTN, APOA1, CFB, AFP, GSN, LOC100068926, GC, AFM, RBP4, LTF, APOD, A2M, EFEMP1, EGFR, SERPINA7, ANXA5, HGFAC, ITIH2, ALDOB, CPN1, LOC100051073, ENSECAP00000033574, TG, PLP1 | |
| 2 | | Brown | 7 | | Collagen biosynthesis and modifying enzymes, and CS/DS degradation | SERPINH1, POSTN, COL11A1, FBLN1, OGN, THBS4, COMP | |
| 3 | | Dark Golden Rod | 4 | | Thick filament | VCL, MYH1, ACTG2, MYH6 | |
| 4 | | Yellow | 3 | | Structural constituent of skin epidermis | KRT82, KRT80, BHMT | |
| 5 | | Green | 3 | | Structural constituent of skin epidermis | KRT4, KRT1, KRT10A | |
| 6 | | Light Green | 2 | | Vault protein Inter-alpha-Trypsin domain | ITIH3, ITIH1 | |
| 7 | | Lime Green | 2 | | - | YWHAE, AOC3 | |
| 8 | | Cyan | 2 | | - | HSPB1, RGN | |
| 9 | | Dark Cyan | 2 | | - | KRT42, KRT6B | |
| 10 | | Blue | 2 | | Tyrosine catabolism | HGD, HPD | |
| 11 | | Purple | 2 | | Factor I membrane attack complex | C6, C7 | |
| 12 | | Pink | 1 | | - | C5 | |
| Secretome (supernatant samples after co-culture for 48 hours compared to 0 hours) Up-regulated | | | | | | | |
| **Cluster number** | **Color cluster** | | **Gene count** | **Primary description** | | | **Protein names** |
| 1 | Red | | 37 | RNA recognition motif domain, and Heterogeneous nuclear ribonucleoprotein U, SPRY domain | | | NUDT21, SFPQ, NONO, ILF2, HNRNPM, DDX39B, RBM8A, HNRNPK, U2AF2, HNRNPF, HNRNPD, EWSR1, PABPC1, FUS, HNRNPH3, PTBP1, SYNCRIP, HNRNPA3, HNRNPR, DDX5, HNRNPA2B1, HNRNPAB, HNRNPA1, HNRNPL, API5, PRMT1, DDX17, ILF3, G3BP1, SNRPD3, HNRNPH1, FUBP1, KHSRP, PCBP2, RTRAF, RBM3, PUF60 |
| 2 | Salmon 2 | | 15 | Proteasome | | | ELOC, PSMD2, PSMD13, PSMD6, PSMC3, PSMB1, PSMA2, PSMA6, HTRA3, PSMD5, PSME2, PSME1, TXNL1, UCHL1, HTRA1 |
| 3 | Fire Brick 2 | | 14 | Collagen formation | | | FBLN2, LOXL1, COL5A2, FBN1, COL6A3, PLOD2, RRBP1, FKBP10, LOXL2, PLOD3, PLOD1, AREL1, BMP1, PCOLCE |
| 4 | Salmon | | 12 | ATP-dependent protein folding chaperone | | | CCT5, TCP1, HSPA4, CCT6B, CCT3, CCT7, CCT8, CCT4, CCT2, HSPA9, HSPH1, PPP2CB |
| 5 | Fire Brick | | 12 | Pentose phosphate pathway | | | PFKP, ALDOA, GPI, G6PD, MDH2, TALDO1, IMPA1, GNPDA1, GNPDA2, MINPP1, PGM2, PGM3 |
| 6 | Sandy Brown | | 11 | Endoplasmic reticulum to Golgi vesicle-mediated transport | | | ARF4, COPG2, COPG1, SEC22B, COPB2, ARCN1, COPA, COPB1, NAPA, LMAN2, SEC23A |
| 7 | Saddle Brown | | 10 | tRNA aminoacylation for protein translation | | | FKBP9, WARS1, AARS1, YARS1, GARS1, RARS1, DNPEP, NAMPT, TARS1, NARS1 |
| 8 | Brown | | 9 | ECM-receptor interaction | | | ITGAV, SGCA, TNC, LAMB1, COL4A2, LAMA5, LAMA2, LAMC1, TGFBI |
| 9 | Dark Golden Rod | | 8 | Arp2/3 complex-mediated actin nucleation | | | CORO1B, ARPC2, ACTR2, ARPC1B, DSTN, NIBAN2, DBNL, ARPC5 |
| 10 | Sandy Brown 2 | | 8 | Activation of Matrix Metalloproteinases | | | CCN2, MMP1, PLAT, MMP14, MMP9, SERPINE1, BSG, ADAMTS5 |
| 11 | Light Green | | 8 | Eukaryotic 48S preinitiation complex | | | WDR43, EIF2S1, RPS4X, RPLP0, PABPC4, EIF3E, PPP1CC, ATP6AP2 |
| 12 | Light Green 4 | | 6 | Septin ring | | | SEPTIN11, SEPTIN6, SEPTIN8, SEPTIN9, SEPTIN7, MAP4 |
| 13 | Light Green 7 | | 5 | Clathrin coat | | | CLTC, AP1B1, AP2B1, SNX9, STAM |
| 14 | Light Green 9 | | 5 | COPI-independent Golgi-to-ER retrograde traffic | | | CAPZB, DYNLL2, TWF1, PLS3, PAFAH1B1 |
| 15 | Light Green 8 | | 4 | Mixed, incl. Disulfide isomerase, and Protein folding in the endoplasmic reticulum | | | ERP29, HINT1, ERP44, ERO1A |
| 16 | Light Green 5 | | 4 | Metalloaminopeptidase activity | | | NPEPPS, LAP3, GCLC, AKR1B1 |
| 17 | Light Green 2 | | 4 | Cytosolic large ribosomal subunit | | | EEF1A1, RPL12, RPL10A, RPL5 |
| 18 | Green | | 4 | Importin-beta, N-terminal domain, and Importin-alpha, importin-beta-binding domain superfamily | | | RANBP1, IPO5, KPNB1, LRRC59 |
| 19 | Light Green 3 | | 3 | Vesicle transport along microtubules | | | KIF5B, DYNC1H1, DCTN2 |
| 20 | Light Green 6 | | 3 | Retromer, cargo-selective complex | | | VPS35, VPS29, VPS26A |
| 21 | Medium Aqua Marine | | 3 | Immunological synapse | | | CD81, SDCBP, ALCAM |
| 22 | Medium Aqua Marine 2 | | 3 | Profilin binding | | | VASP, LASP1, ENAH |
| 23 | Aquamarine 7 | | 3 | - | | | PLBD2, PRCP, CPE |
| 24 | Aquamarine 5 | | 3 | - | | | PLIN3, FASN, VAT1 |
| 25 | Aquamarine 3 | | 3 | Insulin-like growth factor-binding protein family 1-6, chordata, and Retromer complex binding | | | IGFBP7, IGFBP5, PAPPA |
| 26 | Aquamarine | | 3 | Mixed, incl. Disulfide isomerase, and Protein folding in the endoplasmic reticulum | | | HYOU1, MANF, QSOX1 |
| 27 | Cyan | | 3 | - | | | UBA1, DDB1, TSN |
| 28 | Aquamarine 2 | | 3 | Oxaloacetate metabolism | | | GOT2, ACLY, ENOPH1 |
| 29 | Aquamarine 4 | | 3 | De novo IMP biosynthesis | | | PSAT1, ATIC, PAICS |
| 30 | Aquamarine 6 | | 3 | Drug metabolism - cytochrome P450 | | | GSTM3, LOC100058329, LOC100058290 |
| 31 | Sky Blue 8 | | 3 | G protein activity | | | RAB11A, RAB7A, GDI2 |
| 32 | Sky Blue 6 | | 3 | - | | | VAPA, PI4KA, PITPNB |
| 33 | Sky Blue 4 | | 3 | CRMPs in Sema3A signaling | | | DBN1, DPYSL2, DPYSL3 |
| 34 | Sky Blue 2 | | 3 | - | | | GLRX3, APEX1, MARCKS |
| 35 | Sky Blue | | 3 | Cell-extracellular matrix interactions | | | PARVA, PDLIM5, FERMT2 |
| 36 | Sky Blue 3 | | 3 | Glycosaminoglycan degradation | | | GNS, HEXB, HEXA |
| 37 | Sky Blue 5 | | 3 | Filamin-type immunoglobulin domains | | | FLNC, FLNB, EDIL3 |
| 38 | Sky Blue 7 | | 3 | Structural molecule activity conferring elasticity | | | ENSECAP00000050120, AHNAK, MYOF |
| 39 | Cornflower Blue 5 | | 2 | - | | | PTGR1, NXN |
| 40 | Cornflower Blue 4 | | 2 | Plasma lipoprotein remodeling | | | PCSK5, LIPG |
| 41 | Cornflower Blue 2 | | 2 | Laminin binding | | | ECM1, NID1 |
| 42 | Blue | | 2 | - | | | PPP1CA, TRIM28 |
| 43 | Cornflower Blue | | 2 | - | | | LGMN, IFI30 |
| 44 | Cornflower Blue 3 | | 2 | SERine Proteinase Inhibitors | | | SERPING1, SERPINB1 |
| 45 | Medium Slate Blue 5 | | 2 | - | | | CRK, PTK7 |
| 46 | Medium Slate Blue 3 | | 2 | Detoxification of Reactive Oxygen Species and Methylglyoxal Metabolism | | | SOD2, PRDX5 |
| 47 | Medium Slate Blue | | 2 | - | | | THOP1, DPP3 |
| 48 | Medium Slate Blue 2 | | 2 | Chemotaxis | | | CXCL6, CXCL8 |
| 49 | Medium Slate Blue 4 | | 2 | - | | | GANAB, SND1 |
| 50 | Medium Purple | | 2 | Aldehyde dehydrogenase (NAD+) activity | | | ALDH1A2, ADH5 |
| 51 | Medium Purple 2 | | 2 | - | | | ANGPTL2, SCPEP1 |
| 52 | Medium Purple 3 | | 2 | - | | | OLA1, SERPINB8 |
| 53 | Medium Purple 4 | | 2 | Rotamase | | | PPIA, FKBP3 |
| 54 | Medium Purple 5 | | 2 | Selenocysteine synthesis | | | SARS1, SEPHS1 |
| 55 | Medium Purple 6 | | 2 | COPII-coated vesicle budding | | | TFG, SEC31A |
| 56 | Orchid 8 | | 2 | Mixed, incl. Cysteine peptidase, asparagine active site, and Lysosome-associated membrane glycoprotein | | | CTSS, GRN |
| 57 | Orchid 7 | | 2 | - | | | EMILIN1, MVP |
| 58 | Orchid 6 | | 2 | - | | | NANS, UGDH |
| 59 | Orchid 4 | | 2 | Mixed, incl. ALIX V-shaped domain, and Syndecan binding | | | PDCD6IP, PDCD6 |
| 60 | Orchid 2 | | 2 | - | | | PXDN, LMNB1 |
| 61 | Violet 3 | | 2 | - | | | SCARB2, SMPD1 |
| 62 | Violet | | 2 | NCAM signaling for neurite outgrowth | | | SPTAN1, SPTBN1 |
| 63 | Purple | | 2 | - | | | CAPN2, SRI |
| 64 | Violet 2 | | 2 | Desmoplakin, spectrin-like domain, and Calponin/LIMCH1 | | | PLEC, CNN2 |
| 65 | Violet 4 | | 2 | - | | | FSTL1, NUCB1 |
| 66 | Orchid | | 2 | A tetrasaccharide linker sequence is required for GAG synthesis | | | HSPG2, CSPG4 |
| 67 | Orchid 3 | | 1 | - | | | PA2G4 |
| 68 | Orchid 5 | | 1 | - | | | PLAU |
| 69 | Hot Pink 4 | | 1 | - | | | SGTA |
| 70 | Hot Pink 2 | | 1 | - | | | VDAC1 |
| 71 | Hot Pink | | 1 | - | | | AP2A2 |
| 72 | Hot Pink 3 | | 1 | - | | | CNN3 |
| 73 | Pale Violet Red 2 | | 1 | - | | | CDC42 |
| 74 | Pale Violet Red | | 1 | - | | | GDI1 |
| 75 | Pink | | 1 | - | | | VCP |
| 76 | Light Coral 3 | | 1 | - | | | IQGAP1 |
| 77 | Light Coral | | 1 | - | | | CAT |
| 78 | Light Coral 2 | | 1 | - | | | PPP2R1A |
