# Supplementary material for: Bidirectional Interaction Between PGE2-Preconditioned Mesenchymal Stem Cells and Myofibroblasts Mediates Anti-Fibrotic Effects: A Proteomic Investigation into Equine Endometrial Fibrosis Reversal
Source: Proteomes. 2025 Sep 8;13(3):41. doi: 10.3390/proteomes13030041 (PMC12452512; doi:10.3390/proteomes13030041)
Supplement: Supplementary file 1 [file proteomes-13-00041-s001.zip › proteomes-3748745-supplementary-8.22/Table supplementary 5 cluster GO.docx]

| **Proteome (Myofibroblasts) Down-regulated proteins 48hours VS 0 hours** | | | | |  |  |  |
| --- | --- | --- | --- | --- | --- | --- | --- |
| **#term ID** | **Gene Ontology/secretome pathways enriched** | **Gene count** | **Primary description** | **Protein names** | **strength** | **signal** | **FDR** |
| GO:0030198 | Biological Process | 8 | Extracellular matrix organization | EXOC8,MMP1,COL3A1,COL1A2,PXDN,COL2A1,COL5A1,COL1A1 | 1.03 | 0.54 | 0.0115 |
| GO:0072359 | Biological Process | 11 | Circulatory system development | JUNB,RTN4,PDLIM2,SEMA3C,COL3A1,COL1A2,PXDN,MICAL2,COL2A1,HSPG2,COL1A1 | 0.73 | 0.45 | 0.0156 |
| GO:0030199 | Biológico Process | 4 | Collagen fibril organization | COL3A1,COL1A2,PXDN,COL2A1 | 1.53 | 0.54 | 0.0194 |
| GO:0005201 | Molecular Function | 8 | Extracellular matrix structural constituent | MXRA5,COL3A1,COL1A2,PXDN,COL2A1,COL5A1,VCAN,COL1A1 | 1.49 | 1.64 | 1.22e-06 |
| GO:0005583 | Cellular Component | 4 | Fibrillar collagen trimer | COL3A1,COL2A1,COL5A1,COL1A1 | 2.14 | 1.29 | 0.00010 |
| GO:0062023 | Cellular Component | 8 | Collagen-containing extracellular matrix | THBS2,COL3A1,PXDN,COL2A1,COL5A1,VCAN,HSPG2,COL1A1 | 1.07 | 0.93 | 0.00020 |
| GO:0005581 | Cellular Component | 5 | Collagen trimer | COL3A1,COL1A2,COL2A1,COL5A1,COL1A1 | 1.49 | 1.06 | 0.00027 |
| GO:0031012 | Cellular Component | 9 | Extracellular matrix | MMP1,THBS2,COL3A1,PXDN,COL2A1,COL5A1,VCAN,HSPG2,COL1A1 | 0.87 | 0.71 | 0.00092 |
| GO:0005604 | Cellular Component | 4 | Basement membrane | THBS2,PXDN,COL2A1,HSPG2 | 1.23 | 0.5 | 0.0219 |
| ecb04512 | KEGG Pathways | 5 | ECM-receptor interaction | THBS2,COL1A2,COL2A1,HSPG2,COL1A1 | 1.26 | 0.71 | 0.0036 |
| ecb04933 | KEGG Pathways | 4 | AGE-RAGE signaling pathway in diabetic complications | FOXO1,COL1A2,STAT3,COL1A1 | 1.11 | 0.41 | 0.0420 |
| ecb04974 | KEGG Pathways | 4 | Protein digestion and absorption | COL1A2,COL2A1,COL5A1,COL1A1 | 1.13 | 0.41 | 0.0420 |
| MAP-3000171 | Reactome Pathways | 6 | Non-integrin membrane-ECM interactions | COL3A1,COL1A2,COL2A1,COL5A1,HSPG2,COL1A1 | 1.65 | 1.44 | 1.58e-05 |
| MAP-2022090 | Reactome Pathways | 6 | Assembly of collagen fibrils and other multimeric structures | COL3A1,COL1A2,PXDN,COL2A1,COL5A1,COL1A1 | 1.63 | 1.43 | 1.58e-05 |
| MAP-3000178 | Reactome Pathways | 6 | ECM proteoglycans | COL3A1,COL1A2,COL2A1,VCAN,HSPG2,COL1A1 | 1.57 | 1.41 | 1.58e-05 |
| MAP-1442490 | Reactome Pathways | 6 | Collagen degradation | MMP1,COL3A1,COL1A2,COL2A1,COL5A1,COL1A1 | 1.53 | 1.39 | 1.58e-05 |
| MAP-1474228 | Reactome Pathways | 8 | Degradation of the extracellular matrix | CAST,MMP1,COL3A1,COL1A2,COL2A1,COL5A1,HSPG2,COL1A1 | 1.23 | 1.24 | 1.58e-05 |
| MAP-8948216 | Reactome Pathways | 5 | Collagen chain trimerization | COL3A1,COL1A2,COL2A1,COL5A1,COL1A1 | 1.55 | 1.17 | 0.00012 |
| MAP-186797 | Reactome Pathways | 5 | Signaling by PDGF | THBS2,COL3A1,FNDC3B,STAT3,COL2A1 | 1.45 | 1.03 | 0.00031 |
| MAP-1474244 | Reactome Pathways | 10 | Extracellular matrix organization | CAST,MMP1,COL3A1,COL1A2,PXDN,COL2A1,COL5A1,VCAN,HSPG2,COL1A1 | 0.95 | 0.95 | 5.74e-05 |
| MAP-1474290 | Reactome Pathways | 6 | Collagen formation | COL3A1,COL1A2,PXDN,COL2A1,COL5A1,COL1A1 | 1.23 | 0.94 | 0.00038 |
| MAP-216083 | Reactome Pathways | 5 | Integrin cell surface interactions | COL3A1,COL1A2,COL2A1,HSPG2,COL1A1 | 1.35 | 0.92 | 0.00066 |
| MAP-430116 | Reactome Pathways | 3 | GP1b-IX-V activation signalling | COL1A2,FNDC3B,COL1A1 | 1.74 | 0.74 | 0.0048 |
| MAP-2243919 | Reactome Pathways | 3 | Crosslinking of collagen fibrils | COL1A2,PXDN,COL1A1 | 1.72 | 0.73 | 0.0052 |
| MAP-1650814 | Reactome Pathways | 5 | Collagen biosynthesis and modifying enzymes | COL3A1,COL1A2,COL2A1,COL5A1,COL1A1 | 1.2 | 0.71 | 0.0032 |
| MAP-9006934 | Reactome Pathways | 8 | Signaling by Receptor Tyrosine Kinases | JUNB,THBS2,COL3A1,FNDC3B,STAT3,GRB10,COL2A1,COL5A1 | 0.67 | 0.36 | 0.0366 |

| Proteome (Myofibroblasts) Up-regulated proteins 48hours VS 0 hours | | | | |  |  |  |
| --- | --- | --- | --- | --- | --- | --- | --- |
| **#term ID** | **Gene Ontology/secretome pathways enriched** | **Gene count** | **Primary description** | **Protein names** | **strength** | **signal** | **FDR** |
| GO:0051239 | Biological Process | 18 | Regulation of multicellular organismal process | GJA1,MMP14,THBS1,AXL,CCN2,LUM,JAK1,NDFIP1,APP,ATP5F1B,CCN1,ANXA5,RNH1,GPNMB,SLITRK4,SLIT2,PDGFRA,CACNA2D1 | 0.59 | 0.49 | 0.0030 |
| GO:0001525 | Biological Process | 6 | Angiogenesis | THBS1,CCN2,ATP5F1B,CCN1,SLIT2,PDGFRA | 1.09 | 0.5 | 0.0235 |
| GO:0007155 | Biological Process | 10 | Cell adhesion | THBS1,AXL,IGFBP7,CCN2,APP,CCN1,GPNMB,MFGE8,ITGB5,PDGFRA | 0.79 | 0.44 | 0.0235 |
| GO:0016477 | Biological Process | 10 | Cell migration | MMP14,THBS1,AXL,CCN2,ATP5F1B,CCN1,ITGB5,SLIT2,PDGFRA,PLAT | 0.78 | 0.43 | 0.0235 |
| GO:0040017 | Biological Process | 8 | Positive regulation of locomotion | MMP14,THBS1,APP,ATP5F1B,CCN1,GPNMB,SLIT2,PDGFRA | 0.92 | 0.47 | 0.0235 |
| GO:0010033 | Biological Process | 15 | Response to organic substance | CLU,THBS1,IFNGR1,AXL,CCN2,JAK1,APP,ATP5F1B,ANXA5,GSN,ITGB5,SLIT2,PDGFRA,CACNA2D1,FASN | 0.53 | 0.33 | 0.0375 |
| GO:0030334 | Biological Process | 9 | Regulation of cell migration | GJA1,MMP14,THBS1,APP,ATP5F1B,CCN1,GPNMB,SLIT2,PDGFRA | 0.75 | 0.37 | 0.0419 |
| GO:0030335 | Biological Process | 7 | Positive regulation of cell migration | MMP14,THBS1,APP,ATP5F1B,CCN1,GPNMB,PDGFRA | 0.89 | 0.4 | 0.0419 |
| GO:0005539 | Molecular Function | 8 | Glycosaminoglycan binding | THBS1,CCN2,APP,CCN1,ANXA5,GPNMB,MFGE8,SLIT2 | 1.19 | 1.04 | 0.00016 |
| GO:0008201 | Molecular Function | 7 | Heparin binding | THBS1,CCN2,APP,CCN1,ANXA5,GPNMB,SLIT2 | 1.27 | 1.06 | 0.00019 |
| GO:0043394 | Molecular Function | 4 | Proteoglycan binding | CTSK,SDCBP,GPNMB,SLIT2 | 1.87 | 1.13 | 0.00045 |
| GO:0019838 | Molecular Function | 5 | Growth factor binding | THBS1,IGFBP7,CCN1,IGF2R,PDGFRA | 1.32 | 0.78 | 0.0031 |
| GO:0097367 | Molecular Function | 17 | Carbohydrate derivative binding | THBS1,AXL,ITM2B,CCN2,CTSK,JAK1,APP,ATP5F1B,CCN1,SDCBP,ANXA5,GPNMB,MFGE8,SLIT2,PDGFRA,GLUD1,AK3 | 0.51 | 0.44 | 0.0042 |
| GO:0005178 | Molecular Function | 5 | Integrin binding | THBS1,CCN2,CCN1,GPNMB,ITGB5 | 1.24 | 0.7 | 0.0050 |
| GO:0072341 | Molecular Function | 4 | Modified amino acid binding | THBS1,AXL,ANXA5,FASN | 1.4 | 0.65 | 0.0095 |
| GO:0044877 | Molecular Function | 11 | Protein-containing complex binding | THBS1,CCN2,LUM,CTSK,CCN1,GSN,GPNMB,IGF2R,ITGB5,PDGFRA,SPTAN1 | 0.63 | 0.43 | 0.0129 |
| GO:0005102 | Molecular Function | 12 | Signaling receptor binding | THBS1,CCN2,JAK1,APP,ATP5F1B,CCN1,GPNMB,ITGB5,SLIT2,PDGFRA,PLAT,LRIG1 | 0.56 | 0.37 | 0.0254 |
| GO:0045545 | Molecular Function | 2 | Syndecan binding | SDCBP,GPNMB | 2.31 | 0.56 | 0.0254 |
| GO:0001786 | Molecular Function | 3 | Phosphatidylserine binding | THBS1,AXL,ANXA5 | 1.46 | 0.44 | 0.0479 |
| GO:0005576 | Cellular Component | 22 | Extracellular region | CLU,MMP14,THBS1,AXL,IGFBP7,ITM2B,EDIL3,CCN2,LUM,CTSK,APP,PAM,CCN1,ANXA5,RNH1,GSN,MFGE8,CRIM1,SLIT2,PLAT,LRIG1,AGA | 0.62 | 0.73 | 3.09e-06 |
| GO:0009986 | Cellular Component | 12 | Cell surface | THBS1,AXL,DCBLD2,APP,ATP5F1B,LOC102150834,ANXA5,IGF2R,ITGB5,ANTXR1,PDGFRA,PLAT | 0.87 | 0.89 | 4.36e-05 |
| GO:0071944 | Cellular Component | 31 | Cell periphery | GJA1,MMP14,THBS1,IFNGR1,AXL,ITM2B,CCN2,LUM,DCBLD2,NDFIP1,APP,ATP5F1B,LRP10,CCN1,SDCBP,LOC102150834,ANXA5,GSN,GPNMB,MFGE8,TMEM59,IGF2R,ITGB5,ANTXR1,CRIM1,PDGFRA,CACNA2D1,SLC38A4,LRIG1,SPTAN1,FASN | 0.35 | 0.43 | 0.00024 |
| GO:0005615 | Cellular Component | 15 | Extracellular space | CLU,MMP14,THBS1,AXL,IGFBP7,ITM2B,LUM,CTSK,APP,ANXA5,GSN,SLIT2,PLAT,LRIG1,AGA | 0.62 | 0.58 | 0.00057 |
| GO:0031012 | Cellular Component | 7 | Extracellular matrix | MMP14,THBS1,CCN2,LUM,CCN1,MFGE8,LRIG1 | 0.88 | 0.52 | 0.0115 |
| GO:0005886 | Cellular Component | 25 | Plasma membrane | GJA1,MMP14,THBS1,IFNGR1,AXL,ITM2B,DCBLD2,APP,ATP5F1B,LRP10,SDCBP,LOC102150834,ANXA5,GSN,GPNMB,TMEM59,IGF2R,ITGB5,ANTXR1,CRIM1,PDGFRA,CACNA2D1,SLC38A4,SPTAN1,FASN | 0.29 | 0.27 | 0.0363 |
| GO:0012505 | Cellular Component | 19 | Endomembrane system | GJA1,CLU,MMP14,THBS1,ITM2B,RNF24,PTGS2,NDFIP1,APP,PAM,CCN1,TMEM59,IGF2R,PDGFRA,CACNA2D1,GLUD1,PLAT,AGA,FASN | 0.37 | 0.29 | 0.0363 |
| GO:0031410 | Cellular Component | 12 | Cytoplasmic vesicle | CLU,MMP14,THBS1,ITM2B,NDFIP1,APP,PAM,GSN,TMEM59,IGF2R,PLAT,FASN | 0.51 | 0.32 | 0.0429 |
| GO:0005641 | Cellular Component | 2 | Nuclear envelope lumen | APP, IGF2R | 1.96 | 0.47 | 0.0441 |
|  |  |  |  |  |  |  |  |

*There were no significant pathway enrichments observed in the following categories: Reactome Pathways, KEGG Pathways

| Secretome (supernant ) Down-regulated proteins 48hours VS 0 hours | | | | |  |  |  |
| --- | --- | --- | --- | --- | --- | --- | --- |
| **#term ID** | **Gene Ontology/secretome pathways enriched** | **Gene count** | **Primary description** | **Protein names** | **strength** | **signal** | **FDR** |
| GO:0010951 | Biological Process | 14 | Negative regulation of endopeptidase activity | SERPINH1,ITIH3,LOC100059239,SERPINA7,LOC100061763,SERPINC1,VTN,ITIH2,C5,ITIH1,LTF,A2M,KNG1,AHSG | 1.3 | 2.17 | 1.91e-10 |
| GO:0045861 | Biological Process | 15 | Negative regulation of proteolysis | SERPINH1,ITIH3,LOC100059239,SERPINA7,F2,LOC100061763,SERPINC1,VTN,ITIH2,C5,ITIH1,LTF,A2M,KNG1,AHSG | 1.19 | 1.95 | 2.78e-10 |
| GO:0052547 | Biological Process | 16 | Regulation of peptidase activity | SERPINH1,ITIH3,LOC100059239,SERPINA7,LOC100061763,SERPINC1,VTN,ITIH2,C5,ITIH1,FBLN1,GSN,LTF,A2M,KNG1,AHSG | 1.12 | 1.82 | 3.18e-10 |
| GO:0052548 | Biological Process | 15 | Regulation of endopeptidase activity | SERPINH1,ITIH3,LOC100059239,SERPINA7,LOC100061763,SERPINC1,VTN,ITIH2,C5,ITIH1,GSN,LTF,A2M,KNG1,AHSG | 1.13 | 1.78 | 1.18e-09 |
| GO:0030162 | Biological Process | 17 | Regulation of proteolysis | SERPINH1,ITIH3,LOC100059239,SERPINA7,F2,LOC100061763,SERPINC1,VTN,ITIH2,C5,ITIH1,FBLN1,GSN,LTF,A2M,KNG1,AHSG | 0.92 | 1.31 | 3.17e-08 |
| GO:0051248 | Biological Process | 19 | Negative regulation of protein metabolic process | SERPINH1,ITIH3,LOC100059239,SERPINA7,F2,LOC100061763,SERPINC1,VTN,ITIH2,C5,HSPB1,APOD,ITIH1,FBLN1,LTF,EGFR,A2M,KNG1,AHSG | 0.83 | 1.19 | 3.83e-08 |
| GO:0045109 | Biological Process | 8 | Intermediate filament organization | KRT82,KRT6B,KRT71,KRT4,KRT42,ENSECAP00000033574,KRT1,KRT80 | 1.63 | 2.06 | 4.60e-08 |
| GO:0043086 | Biological Process | 16 | Negative regulation of catalytic activity | SERPINH1,ITIH3,LOC100059239,SERPINA7,LOC100061763,SERPINC1,VTN,ITIH2,C5,HSPB1,ITIH1,LTF,AOC3,A2M,KNG1,AHSG | 0.9 | 1.22 | 1.53e-07 |
| GO:0007596 | Biological Process | 8 | Blood coagulation | F5,F2,SERPINC1,ANXA5,FBLN1,PLG,COMP,KNG1 | 1.4 | 1.54 | 1.71e-06 |
| GO:0097435 | Biological Process | 13 | Supramolecular fiber organization | SERPINH1,KRT82,KRT6B,KRT71,KRT4,KRT42,GSN,ENSECAP00000033574,ACTG2,COMP,MYH6,KRT1,KRT80 | 0.91 | 1.07 | 4.80e-06 |
| GO:0031424 | Biological Process | 6 | Keratinization | KRT82,KRT6B,KRT71,KRT4,KRT1,KRT80 | 1.61 | 1.46 | 1.01e-05 |
| GO:0030216 | Biological Process | 7 | Keratinocyte differentiation | KRT82,KRT6B,KRT71,KRT4,KRT10A,KRT1,KRT80 | 1.37 | 1.29 | 1.85e-05 |
| GO:0050878 | Biological Process | 9 | Regulation of body fluid levels | F5,F2,SERPINC1,ANXA5,FBLN1,PLG,COMP,KRT1,KNG1 | 1.08 | 1.06 | 3.97e-05 |
| GO:0006950 | Biological Process | 26 | Response to stress | ITIH3,F5,LOC100059239,YWHAE,F2,SERPINC1,C5,THBS4,HSPB1,APOD,POLR3C,ANXA5,FBLN1,GSN,MT2A,LTF,PLG,EGFR,COMP,ALB,CFB,PLP1,C6,KRT1,KNG1,AHSG | 0.47 | 0.58 | 7.72e-05 |
| GO:0009611 | Biological Process | 9 | Response to wounding | F5,F2,SERPINC1,APOD,ANXA5,FBLN1,PLG,COMP,KNG1 | 1.03 | 0.97 | 9.51e-05 |
| GO:0043588 | Biological Process | 8 | Skin development | KRT82,KRT6B,KRT71,KRT4,KRT10A,COMP,KRT1,KRT80 | 1.1 | 0.97 | 0.00014 |
| GO:0050790 | Biological Process | 21 | Regulation of catalytic activity | SERPINH1,ITIH3,LOC100059239,SERPINA7,F2,LOC100061763,SERPINC1,VTN,ITIH2,C5,HSPB1,ITIH1,FBLN1,GSN,LTF,EGFR,AOC3,RGN,A2M,KNG1,AHSG | 0.51 | 0.55 | 0.00043 |
| GO:1900046 | Biological Process | 5 | Regulation of hemostasis | F2,SERPINC1,PLG,COMP,KNG1 | 1.47 | 0.98 | 0.00045 |
| GO:0050818 | Biological Process | 5 | Regulation of coagulation | F2,SERPINC1,ANXA5,PLG,KNG1 | 1.45 | 0.96 | 0.00052 |
| GO:0051246 | Biological Process | 22 | Regulation of protein metabolic process | SERPINH1,ITIH3,LOC100059239,SERPINA7,F2,LOC100061763,SERPINC1,VTN,ITIH2,C5,THBS4,HSPB1,APOD,ITIH1,FBLN1,GSN,LTF,EGFR,ENPP2,A2M,KNG1,AHSG | 0.48 | 0.53 | 0.00052 |
| GO:0009888 | Biological Process | 17 | Tissue development | SERPINH1,KRT82,B4GAT1,KRT6B,VCL,VTN,KRT71,OGN,KRT4,KRT10A,POSTN,ENSECAP00000033574,ACTG2,COMP,KRT1,KRT80,COL11A1 | 0.58 | 0.58 | 0.00057 |
| GO:0065009 | Biological Process | 23 | Regulation of molecular function | SERPINH1,IL1RAP,ITIH3,LOC100059239,SERPINA7,F2,LOC100061763,SERPINC1,ALDOB,VTN,ITIH2,C5,HSPB1,ITIH1,FBLN1,GSN,LTF,EGFR,AOC3,RGN,A2M,KNG1,AHSG | 0.44 | 0.46 | 0.0016 |
| GO:0006959 | Biological Process | 7 | Humoral immune response | LOC100059239,F2,C5,LTF,CFB,C6,KRT1 | 1.02 | 0.71 | 0.0019 |
| GO:1900047 | Biological Process | 4 | Negative regulation of hemostasis | F2,PLG,COMP,KNG1 | 1.52 | 0.76 | 0.0031 |
| GO:0050819 | Biological Process | 4 | Negative regulation of coagulation | F2,ANXA5,PLG,KNG1 | 1.51 | 0.76 | 0.0032 |
| GO:0032502 | Biological Process | 31 | Developmental process | SERPINH1,KRT82,EFEMP1,B4GAT1,KRT6B,VCL,VTN,KRT71,OGN,KRT4,C5,KRT10A,APOD,AFP,POSTN,CNTN1,KRT42,GSN,ENSECAP00000033574,LTF,ACTG2,PLG,EGFR,APOB,COMP,PLP1,C6,MYH6,KRT1,KRT80,COL11A1 | 0.33 | 0.38 | 0.0033 |
| GO:0032501 | Biological Process | 37 | Multicellular organismal process | SERPINH1,F5,KRT82,EFEMP1,B4GAT1,F2,KRT6B,VCL,SERPINC1,VTN,KRT71,OGN,KRT4,C5,THBS4,APOD,AFP,CNTN1,ANXA5,FBLN1,GSN,MYH1,ENSECAP00000033574,LTF,ACTG2,PLG,APOB,COMP,ENPP2,CACNA2D1,PLP1,C6,MYH6,KRT1,KRT80,KNG1,COL11A1 | 0.27 | 0.35 | 0.0041 |
| GO:0006956 | Biological Process | 5 | Complement activation | LOC100059239,C5,CFB,C6,KRT1 | 1.23 | 0.68 | 0.0042 |
| GO:0048856 | Biological Process | 29 | Anatomical structure development | SERPINH1,KRT82,EFEMP1,B4GAT1,KRT6B,VCL,VTN,KRT71,OGN,KRT4,C5,KRT10A,APOD,AFP,POSTN,CNTN1,GSN,ENSECAP00000033574,LTF,ACTG2,PLG,APOB,COMP,PLP1,C6,MYH6,KRT1,KRT80,COL11A1 | 0.34 | 0.38 | 0.0042 |
| GO:0051239 | Biological Process | 19 | Regulation of multicellular organismal process | IL1RAP,EFEMP1,F2,VCL,SERPINC1,C5,THBS4,HSPB1,APOD,POLR3C,ANXA5,LTF,PLG,EGFR,COMP,ENPP2,CACNA2D1,KNG1,AHSG | 0.45 | 0.41 | 0.0062 |
| GO:0032101 | Biological Process | 11 | Regulation of response to external stimulus | F2,SERPINC1,C5,THBS4,HSPB1,POLR3C,LTF,PLG,KRT1,KNG1,AHSG | 0.67 | 0.49 | 0.0064 |
| GO:0008015 | Biological Process | 7 | Blood circulation | F5,GSN,ACTG2,COMP,CACNA2D1,MYH6,KNG1 | 0.91 | 0.56 | 0.0070 |
| GO:0006952 | Biological Process | 13 | Defense response | ITIH3,LOC100059239,F2,C5,POLR3C,GSN,LTF,CFB,PLP1,C6,KRT1,KNG1,AHSG | 0.58 | 0.45 | 0.0075 |
| GO:0030193 | Biological Process | 4 | Regulation of blood coagulation | F2,SERPINC1,PLG,KNG1 | 1.38 | 0.64 | 0.0075 |
| GO:0030212 | Biological Process | 3 | Hyaluronan metabolic process | ITIH3,ITIH2,ITIH1 | 1.66 | 0.59 | 0.0131 |
| GO:0048519 | Biological Process | 29 | Negative regulation of biological process | SERPINH1,ITIH3,LOC100059239,EFEMP1,SERPINA7,F2,LOC100061763,SERPINC1,VTN,ITIH2,KRT4,C5,THBS4,HSPB1,APOD,ITIH1,ANXA5,FBLN1,GSN,MT2A,LTF,PLG,EGFR,COMP,ALB,A2M,KRT1,KNG1,AHSG | 0.3 | 0.32 | 0.0155 |
| GO:0048513 | Biological Process | 20 | Animal organ development | SERPINH1,KRT82,EFEMP1,B4GAT1,KRT6B,KRT71,OGN,KRT4,KRT10A,APOD,AFP,CNTN1,ENSECAP00000033574,LTF,ACTG2,PLG,COMP,MYH6,KRT1,KRT80 | 0.39 | 0.33 | 0.0228 |
| GO:0030855 | Biological Process | 8 | Epithelial cell differentiation | KRT82,KRT6B,KRT71,KRT4,KRT10A,ENSECAP00000033574,KRT1,KRT80 | 0.74 | 0.41 | 0.0239 |
| GO:0006953 | Biological Process | 3 | Acute-phase response | ITIH3,F2,AHSG | 1.54 | 0.5 | 0.0248 |
| GO:0051241 | Biological Process | 10 | Negative regulation of multicellular organismal process | EFEMP1,F2,THBS4,APOD,ANXA5,LTF,PLG,EGFR,KNG1,AHSG | 0.62 | 0.38 | 0.0271 |
| GO:0006954 | Biological Process | 7 | Inflammatory response | ITIH3,LOC100059239,F2,C5,PLP1,KNG1,AHSG | 0.8 | 0.41 | 0.0280 |
| GO:0006936 | Biological Process | 5 | Muscle contraction | GSN,MYH1,COMP,CACNA2D1,MYH6 | 1.0 | 0.42 | 0.0327 |
| GO:0030198 | Biological Process | 6 | Extracellular matrix organization | SERPINH1,VTN,POSTN,FBLN1,COMP,COL11A1 | 0.87 | 0.4 | 0.0346 |
| GO:0009605 | Biological Process | 16 | Response to external stimulus | LOC100059239,B4GAT1,F2,C5,HSPB1,POLR3C,POSTN,CNTN1,GSN,LTF,APOB,ENPP2,ALB,CFB,C6,KRT1 | 0.43 | 0.31 | 0.0374 |
| GO:0050896 | Biological Process | 41 | Response to stimulus | IL1RAP,ITIH3,F5,LOC100059239,YWHAE,EFEMP1,B4GAT1,F2,SERPINC1,VTN,OGN,C5,THBS4,HSPB1,APOD,AFP,POLR3C,POSTN,TG,CNTN1,ANXA5,LOC100068926,FBLN1,GSN,MT2A,ENSECAP00000033574,LTF,PLG,EGFR,APOB,AOC3,COMP,ENPP2,ALB,CACNA2D1,CFB,PLP1,C6,KRT1,KNG1,AHSG | 0.2 | 0.25 | 0.0376 |
| GO:0006958 | Biological Process | 3 | Complement activation, classical pathway | LOC100059239,C5,C6 | 1.44 | 0.43 | 0.0417 |
| GO:0030195 | Biological Process | 3 | Negative regulation of blood coagulation | F2,PLG,KNG1 | 1.35 | 2.28 | 1.04e-10 |
| GO:0010927 | Biological Process | 4 | Cellular component assembly involved in morphogenesis | CNTN1,ENSECAP00000033574,ACTG2,MYH6 | 1.28 | 2.17 | 1.04e-10 |
| GO:0004866 | Molecular Function | 13 | Endopeptidase inhibitor activity | SERPINH1,ITIH3,LOC100059239,SERPINA7,LOC100061763,SERPINC1,ITIH2,C5,ITIH1,LTF,A2M,KNG1,AHSG | 1.95 | 2.49 | 4.77e-09 |
| GO:0061134 | Molecular Function | 14 | Peptidase regulator activity | SERPINH1,ITIH3,LOC100059239,SERPINA7,LOC100061763,SERPINC1,ITIH2,C5,ITIH1,FBLN1,LTF,A2M,KNG1,AHSG | 1.07 | 1.6 | 8.10e-09 |
| GO:0030280 | Molecular Function | 7 | Structural constituent of skin epidermis | KRT82,KRT6B,KRT71,KRT4,KRT10A,KRT1,KRT80 | 1.37 | 1.54 | 1.37e-06 |
| GO:0004857 | Molecular Function | 14 | Enzyme inhibitor activity | SERPINH1,ITIH3,LOC100059239,SERPINA7,LOC100061763,SERPINC1,ITIH2,C5,HSPB1,ITIH1,LTF,A2M,KNG1,AHSG | 1.1 | 1.12 | 2.25e-05 |
| GO:0004867 | Molecular Function | 8 | Serine-type endopeptidase inhibitor activity | SERPINH1,ITIH3,SERPINA7,LOC100061763,SERPINC1,ITIH2,ITIH1,A2M | 1.18 | 1.15 | 2.92e-05 |
| GO:0005539 | Molecular Function | 9 | Glycosaminoglycan binding | F2,SERPINC1,THBS4,POSTN,ITIH1,ANXA5,LTF,APOB,COMP | 0.65 | 0.73 | 5.42e-05 |
| GO:0008201 | Molecular Function | 8 | Heparin binding | F2,SERPINC1,THBS4,POSTN,ANXA5,LTF,APOB,COMP | 0.55 | 0.64 | 6.42e-05 |
| GO:0030234 | Molecular Function | 17 | Enzyme regulator activity | SERPINH1,ITIH3,LOC100059239,SERPINA7,LOC100061763,SERPINC1,ITIH2,C5,HSPB1,ITIH1,FBLN1,LTF,EGFR,RGN,A2M,KNG1,AHSG | 0.7 | 0.67 | 0.00039 |
| GO:0098772 | Molecular Function | 21 | Molecular function regulator activity | SERPINH1,ITIH3,LOC100059239,YWHAE,SERPINA7,LOC100061763,SERPINC1,OGN,ITIH2,C5,THBS4,HSPB1,ITIH1,TG,FBLN1,LTF,EGFR,RGN,A2M,KNG1,AHSG | 0.68 | 0.59 | 0.0015 |
| GO:0005198 | Molecular Function | 13 | Structural molecule activity | KRT82,KRT6B,VCL,KRT71,KRT4,KRT10A,KRT42,FBLN1,ENSECAP00000033574,PLP1,KRT1,KRT80,COL11A1 | 2.29 | 0.53 | 0.0231 |
| GO:0008289 | Molecular Function | 12 | Lipid binding | APOA1,F2,APOD,AFP,ANXA5,GC,GSN,LTF,APOB,ALB,AFM,RBP4 | 0.32 | 0.28 | 0.0405 |
| GO:0005006 | Molecular Function | 2 | Epidermal growth factor receptor activity | EFEMP1,EGFR | 1.35 | 2.28 | 1.04e-10 |
| GO:0046872 | Molecular Function | 24 | Metal ion binding | F5,EFEMP1,B4GAT1,F2,HPD,BHMT,LOC100051073,THBS4,AFP,POSTN,CPN1,ANXA5,LOC100068926,FBLN1,GSN,MT2A,HGD,LTF,AOC3,COMP,RGN,ENPP2,ALB,AFM | 1.28 | 2.17 | 1.04e-10 |
| GO:0005615 | Cellular Component | 39 | Extracellular space | SERPINH1,F5,LOC100059239,APOA1,EFEMP1,SERPINA7,F2,LOC100061763,SERPINC1,BHMT,VTN,C5,LOC100051073,THBS4,APOD,AFP,POSTN,CPN1,TG,ANXA5,GC,FBLN1,GSN,HGD,HGFAC,LTF,PLG,APOB,COMP,ENPP2,ALB,AFM,CFB,C6,RBP4,A2M,KNG1,COL11A1,AHSG | 0.89 | 1.92 | 8.44e-23 |
| GO:0005576 | Cellular Component | 43 | Extracellular region | SERPINH1,ITIH3,F5,LOC100059239,APOA1,EFEMP1,SERPINA7,F2,LOC100061763,SERPINC1,BHMT,VTN,OGN,ITIH2,C5,LOC100051073,THBS4,APOD,AFP,POSTN,CPN1,ITIH1,TG,ANXA5,GC,FBLN1,GSN,HGD,HGFAC,LTF,PLG,APOB,COMP,ENPP2,ALB,AFM,CFB,C6,RBP4,A2M,KNG1,COL11A1,AHSG | 0.76 | 1.51 | 3.21e-21 |
| GO:0005882 | Cellular Component | 9 | Intermediate filament | KRT82,KRT6B,KRT71,KRT4,KRT10A,KRT42,ENSECAP00000033574,KRT1,KRT80 | 1.31 | 1.58 | 5.45e-07 |
| GO:0045095 | Cellular Component | 7 | Keratin filament | KRT82,KRT6B,KRT71,KRT4,KRT10A,KRT1,KRT80 | 1.53 | 1.66 | 1.18e-06 |
| GO:0099512 | Cellular Component | 16 | Supramolecular fiber | KRT82,KRT6B,VCL,KRT71,KRT4,HSPB1,KRT10A,KRT42,FBLN1,MYH1,ENSECAP00000033574,ACTG2,MYH6,KRT1,KRT80,COL11A1 | 0.78 | 0.96 | 2.30e-06 |
| GO:0062023 | Cellular Component | 8 | Collagen-containing extracellular matrix | EFEMP1,F2,VTN,THBS4,POSTN,FBLN1,COMP,COL11A1 | 1.04 | 0.93 | 0.00016 |
| GO:0031012 | Cellular Component | 9 | Extracellular matrix | EFEMP1,F2,VTN,OGN,THBS4,POSTN,FBLN1,COMP,COL11A1 | 0.84 | 0.69 | 0.0010 |
| GO:0030016 | Cellular Component | 6 | Myofibril | VCL,HSPB1,MYH1,ENSECAP00000033574,ACTG2,MYH6 | 0.99 | 0.61 | 0.0051 |
| GO:0099513 | Cellular Component | 10 | Polymeric cytoskeletal fiber | KRT82,KRT6B,KRT71,KRT4,KRT10A,KRT42,ENSECAP00000033574,ACTG2,KRT1,KRT80 | 0.69 | 0.52 | 0.0051 |
| GO:0001533 | Cellular Component | 3 | Cornified envelope | HSPB1,KRT10A,KRT1 | 1.6 | 0.64 | 0.0087 |
| GO:0030017 | Cellular Component | 5 | Sarcomere | HSPB1,MYH1,ENSECAP00000033574,ACTG2,MYH6 | 0.97 | 0.47 | 0.0210 |
| GO:0005579 | Cellular Component | 2 | Membrane attack complex | C5,C6 | 1.99 | 0.49 | 0.0291 |
| ecb04610 | KEGG Pathways | 11 | Complement and coagulation cascades | F5,LOC100059239,F2,SERPINC1,VTN,C5,PLG,C7,CFB,C6,KNG1 | 1.64 | 3.03 | 2.02e-12 |
| ecb05150 | KEGG Pathways | 6 | Staphylococcus aureus infection | LOC100059239,C5,KRT10A,KRT42,PLG,CFB | 1.35 | 1.16 | 6.71e-05 |
| ecb00350 | KEGG Pathways | 3 | Tyrosine metabolism | HPD,HGD,AOC3 | 1.35 | 0.47 | 0.0301 |
| ecb05143 | KEGG Pathways | 3 | African trypanosomiasis | APOA1,LOC100068926,KNG1 | 1.4 | 0.47 | 0.0301 |
| ecb05322 | KEGG Pathways | 4 | Systemic lupus erythematosus | LOC100059239,C5,C7,C6 | 1.13 | 0.45 | 0.0301 |
| ecb05144 | KEGG Pathways | 3 | Malaria | THBS4,LOC100068926,COMP | 1.28 | 0.44 | 0.0353 |
| MAP-381426 | Reactome Pathways | 10 | Regulation of Insulin-like Growth Factor (IGF) transport and uptake by Insulin-like Growth Factor Binding Proteins (IGFBPs) | F5,LOC100059239,APOA1,SERPINC1,ITIH2,AFP,APOB,ALB,KNG1,AHSG | 1.29 | 1.67 | 1.40e-07 |
| MAP-6809371 | Reactome Pathways | 9 | Formation of the cornified envelope | KRT82,KRT6B,KRT71,KRT4,KRT10A,KRT42,ENSECAP00000033574,KRT1,KRT80 | 1.4 | 1.77 | 1.40e-07 |
| MAP-8957275 | Reactome Pathways | 10 | Post-translational protein phosphorylation | F5,LOC100059239,APOA1,SERPINC1,ITIH2,AFP,APOB,ALB,KNG1,AHSG | 1.32 | 1.7 | 1.40e-07 |
| MAP-114608 | Reactome Pathways | 10 | Platelet degranulation | ITIH3,F5,APOA1,LOC100061763,VCL,ANXA5,PLG,A2M,KNG1,AHSG | 1.18 | 1.43 | 7.75e-07 |
| MAP-76005 | Reactome Pathways | 10 | Response to elevated platelet cytosolic Ca2+ | ITIH3,F5,APOA1,LOC100061763,VCL,ANXA5,PLG,A2M,KNG1,AHSG | 1.17 | 1.42 | 7.85e-07 |
| MAP-6805567 | Reactome Pathways | 9 | Keratinization | KRT82,KRT6B,KRT71,KRT4,KRT10A,KRT42,ENSECAP00000033574,KRT1,KRT80 | 1.2 | 1.36 | 2.43e-06 |
| MAP-140877 | Reactome Pathways | 6 | Formation of Fibrin Clot (Clotting Cascade) | F5,F2,LOC100061763,SERPINC1,A2M,KNG1 | 1.61 | 1.56 | 3.97e-06 |
| MAP-76002 | Reactome Pathways | 11 | Platelet activation, signaling and aggregation | ITIH3,F5,APOA1,F2,LOC100061763,VCL,ANXA5,PLG,A2M,KNG1,AHSG | 0.95 | 1.05 | 1.14e-05 |
| MAP-109582 | Reactome Pathways | 14 | Hemostasis | ITIH3,F5,APOA1,SERPINA7,F2,LOC100061763,VCL,SERPINC1,ANXA5,PLG,APOB,A2M,KNG1,AHSG | 0.72 | 0.76 | 7.82e-05 |
| MAP-977606 | Reactome Pathways | 7 | Regulation of Complement cascade | LOC100059239,F2,VTN,C5,CPN1,CFB,C6 | 1.19 | 1.07 | 7.98e-05 |
| MAP-140837 | Reactome Pathways | 4 | Intrinsic Pathway of Fibrin Clot Formation | F2,LOC100061763,A2M,KNG1 | 1.79 | 1.18 | 0.00015 |
| MAP-166658 | Reactome Pathways | 7 | Complement cascade | LOC100059239,F2,VTN,C5,CPN1,CFB,C6 | 1.14 | 0.99 | 0.00015 |
| MAP-1474244 | Reactome Pathways | 9 | Extracellular matrix organization | SERPINH1,EFEMP1,LOC100061763,VTN,FBLN1,PLG,COMP,A2M,COL11A1 | 0.87 | 0.76 | 0.00049 |
| MAP-174577 | Reactome Pathways | 3 | Activation of C3 and C5 | LOC100059239,C5,CFB | 2.1 | 1.04 | 0.00055 |
| MAP-8963898 | Reactome Pathways | 4 | Plasma lipoprotein assembly | APOA1,LOC100061763,APOB,A2M | 1.51 | 0.88 | 0.0012 |
| MAP-174824 | Reactome Pathways | 5 | Plasma lipoprotein assembly, remodeling, and clearance | APOA1,LOC100061763,PLG,APOB,A2M | 1.19 | 0.74 | 0.0023 |
| MAP-140875 | Reactome Pathways | 3 | Common Pathway of Fibrin Clot Formation | F5,F2,SERPINC1 | 1.58 | 0.63 | 0.0095 |
| MAP-8963896 | Reactome Pathways | 3 | HDL assembly | APOA1,LOC100061763,A2M | 1.58 | 0.63 | 0.0095 |
| MAP-1266738 | Reactome Pathways | 10 | Developmental Biology | KRT82,KRT6B,KRT71,KRT4,KRT10A,CNTN1,KRT42,ENSECAP00000033574,KRT1,KRT80 | 0.61 | 0.41 | 0.0156 |
| MAP-8963899 | Reactome Pathways | 3 | Plasma lipoprotein remodeling | APOA1,PLG,APOB | 1.48 | 0.56 | 0.0156 |
| MAP-9707616 | Reactome Pathways | 3 | Heme signaling | APOA1,LOC100068926,APOB | 1.48 | 0.56 | 0.0156 |
| MAP-8963684 | Reactome Pathways | 2 | Tyrosine catabolism | HPD,HGD | 2.07 | 0.56 | 0.0186 |
| MAP-166665 | Reactome Pathways | 2 | Terminal pathway of complement | C5,C6 | 1.92 | 0.49 | 0.0303 |
| MAP-2129379 | Reactome Pathways | 3 | Molecules associated with elastic fibres | EFEMP1,VTN,FBLN1 | 1.34 | 0.46 | 0.0303 |
| MAP-8964041 | Reactome Pathways | 2 | LDL remodeling | PLG,APOB | 1.92 | 0.49 | 0.0303 |
| MAP-168249 | Reactome Pathways | 14 | Innate Immune System | LOC100059239,F2,VCL,VTN,C5,CPN1,LOC100068926,GSN,LTF,APOB,CFB,C6,KRT1,AHSG | 0.44 | 0.32 | 0.0329 |
| MAP-3000471 | Reactome Pathways | 2 | Scavenging by Class B Receptors | APOA1,APOB | 1.86 | 0.47 | 0.0329 |
| MAP-8963901 | Reactome Pathways | 2 | Chylomicron remodeling | APOA1,APOB | 1.81 | 0.45 | 0.0379 |
| MAP-1566948 | Reactome Pathways | 3 | Elastic fibre formation | EFEMP1,VTN,FBLN1 | 1.26 | 0.42 | 0.0419 |
| MAP-975634 | Reactome Pathways | 3 | Retinoid metabolism and transport | APOA1,APOB,RBP4 | 1.25 | 0.42 | 0.0428 |
| Secretome (supernant )Up-regulated proteins 48hours VS 0 hours | | | | |  |  |  |
| **#term ID** | **Gene Ontology/secretome pathways enriched** | **Gene count** | **Primary description** | **Protein names** | **strength** | **signal** | **FDR** |
| GO:0006457 | Biological Process | 19 | Protein folding | FKBP9,HSPA9,CCT8,HYOU1,PPIA,CCT7,ERP44,QSOX1,FKBP3,TCP1,HSPH1,CCT5,CCT3,HSPA4,FKBP10,CCT4,ERO1A,CCT2,CCT6B | 0.81 | 1.05 | 5.96e-06 |
| GO:0097435 | Biological Process | 29 | Supramolecular fiber organization | API5,VASP,ACTR2,COL5A2,PLS3,KPNB1,PAFAH1B1,CORO1B,DPT,ARPC5,AREL1,DSTN,PLOD3,ENAH,LOXL1,TWF1,LOXL2,EMILIN1,DYNC1H1,CDC42,FKBP10,DPYSL3,DBN1,PXDN,ARPC2,MARCKS,FLNC,PLEC,ARPC1B | 0.6 | 0.9 | 5.96e-06 |
| GO:0030036 | Biological Process | 29 | Actin cytoskeleton organization | API5,VASP,ACTR2,CRK,PTK7,PLS3,PAFAH1B1,CORO1B,ARPC5,DSTN,CNN3,ENAH,TWF1,SDCBP,CNN2,CDC42,F11R,CAPZB,DPYSL3,DBN1,PARVA,ARPC2,MARCKS,PDLIM5,SPTAN1,FLNC,DBNL,ARPC1B,FLNB | 0.58 | 0.86 | 9.58e-06 |
| GO:0030198 | Biological Process | 19 | Extracellular matrix organization | ADAMTS7,MMP14,FBLN2,LAMB1,COL5A2,DPT,NID1,QSOX1,TGFBI,MMP1,PLOD3,LOXL1,LAMC1,LOXL2,EMILIN1,FKBP10,PXDN,COL4A2,ADAMTS5 | 0.71 | 0.87 | 3.93e-05 |
| GO:0033036 | Biological Process | 64 | Macromolecule localization | ERP29,VPS35,ARF4,CD81,NAPA,HSPA9,LMNB1,GDI2,CPE,AP2B1,PLIN3,VPS26A,SGTA,COPG2,KPNB1,PAFAH1B1,IPO5,RRBP1,CORO1B,AP2A2,SPTBN1,GOT2,SEC31A,TCP1,ARCN1,FBN1,FERMT2,PDCD6,RBM8A,INHBA,COPA,PLOD3,SEPTIN6,TWF1,LIPG,SNX9,F11R,CLTC,RAB7A,SEPTIN9,CCT4,RAB11A,STAM,SEC23A,COPB1,HNRNPA2B1,SEPTIN8,DBN1,SEPTIN11,AP1B1,VPS29,DCTN2,CCT2,GDI1,COPB2,SEPTIN7,VCP,COPG1,SCARB2,SRI,PITPNB,PLEC,HEXA,DDX39B | 0.32 | 0.62 | 3.93e-05 |
| GO:0006888 | Biological Process | 13 | Endoplasmic reticulum to Golgi vesicle-mediated transport | HYOU1,SEC22B,COPG2,SEC31A,ARCN1,TFG,PDCD6,COPA,LMAN2,SEC23A,COPB1,COPB2,COPG1 | 0.88 | 0.91 | 7.35e-05 |
| GO:0009987 | Biological Process | 287 | Cellular process | ELOC,ERP29,FKBP9,NUDT21,API5,KIF5B,VPS35,PTGR1,ARF4,CD81,NAPA,PLAU,HSPA9,NPEPPS,ADAMTS7,VASP,PCSK5,LMNB1,SLC39A14,MMP14,ECM1,ACTR2,GDI2,HNRNPA1,FBLN2,LAMB1,PLBD2,CPE,AP2B1,PLIN3,VPS26A,PLOD2,YARS1,SGTA,PPP1CA,LGMN,CSPG4,RPL12,TRIM28,RPS4X,HDGF,PPP2CB,DDX5,CRK,COL5A2,PTK7,CCT8,MANF,SOD2,HEXB,THOP1,SND1,VDAC1,RTRAF,CXCL6,HNRNPL,DDB1,SMPD1,IGFBP7,IGFBP5,MMP9,PLS3,HYOU1,PSMD5,STC1,RPL10A,SEC22B,COPG2,SEPHS1,KPNB1,PAFAH1B1,UBA1,IPO5,UGDH,ATIC,GANAB,G6PD,RRBP1,ALDH1A2,CORO1B,AP2A2,CXCL8,RPLP0,TSN,DPT,SPTBN1,ANGPTL2,OLA1,PPIA,CCN2,NID1,GOT2,CCT7,ERP44,QSOX1,PPP1CC,PRDX5,GCLC,ILF3,LRRC59,CDKL3,PSAT1,SEC31A,FKBP3,EIF2S1,TCP1,GRN,KHSRP,GPI,HSPH1,ALCAM,ARPC5,AREL1,GSTM3,CCT5,PTBP1,ENOPH1,GARS1,SERPINE1,PAICS,ARCN1,DSTN,FBN1,SARS1,CNN3,TFG,EEF1A1,MXRA8,PSMA2,FERMT2,TGFBI,CCT3,PDCD6,RPL5,TARS1,MMP1,HSPA4,RBM8A,ALOX12,FHL2,APEX1,PSMA6,INHBA,COPA,PLOD3,ENAH,CTSS,PSMD2,PAPPA,IMPA1,GNPDA1,HINT1,SNRPD3,MDH2,HNRNPA3,LOXL1,LAMC1,SEPTIN6,CRLF1,PGM3,TWF1,HNRNPM,RBM3,LOC100058329,RARS1,PUF60,PSMC3,LOC100058290,LOXL2,MVP,EMILIN1,DYNC1H1,LAMA2,EIF3E,LIPG,MYOF,SDCBP,DYNLL2,NIBAN2,NARS1,NUCB1,UCHL1,PGM2,SNX9,BSG,CNN2,CDC42,F11R,CLTC,NAMPT,RANBP1,PABPC4,NXN,FKBP10,RAB7A,WARS1,ADH5,LMAN2,U2AF2,SEPTIN9,DPYSL2,NDRG1,CCT4,PFKP,ACLY,RAB11A,HTRA1,SFPQ,STAM,SLC44A1,SYNCRIP,G3BP1,VAPA,CAPZB,DDX17,SEC23A,DPYSL3,ERO1A,AKR1B1,COPB1,PI4KA,TXNL1,FUS,HNRNPK,HNRNPA2B1,SEPTIN8,DBN1,SEPTIN11,SGCA,AP1B1,VPS29,DCTN2,GLRX3,CCT2,GDI1,ALDOA,COPB2,MINPP1,SERPINB8,TNC,GNPDA2,SEPTIN7,LAMA5,VCP,COPG1,PDCD6IP,MAP4,PARVA,IQGAP1,PXDN,NCSTN,PABPC1,ITGAV,ARPC2,COL4A2,FOLR1,CAT,MARCKS,SERPINB1,PSMD13,AARS1,PDLIM5,PLAT,SCARB2,SRI,SPTAN1,TXNDC17,TALDO1,CCT6B,FLNC,DBNL,PITPNB,CAPN2,PPP2R1A,PRMT1,PLEC,WDR43,HEXA,FSTL1,ARPC1B,COL6A3,FASN,FLNB,PSMD6,DDX39B,ADAMTS5,RNASE4 | 0.06 | 0.42 | 0.00012 |
| GO:0008104 | Biological Process | 53 | Protein localization | ERP29,VPS35,ARF4,CD81,NAPA,HSPA9,LMNB1,GDI2,CPE,AP2B1,VPS26A,SGTA,COPG2,KPNB1,PAFAH1B1,IPO5,RRBP1,CORO1B,AP2A2,SPTBN1,SEC31A,TCP1,ARCN1,FBN1,FERMT2,PDCD6,COPA,PLOD3,SEPTIN6,TWF1,SNX9,F11R,CLTC,RAB7A,SEPTIN9,RAB11A,STAM,SEC23A,COPB1,SEPTIN8,DBN1,SEPTIN11,AP1B1,VPS29,DCTN2,GDI1,COPB2,SEPTIN7,VCP,COPG1,SCARB2,SRI,PLEC | 0.33 | 0.56 | 0.00023 |
| GO:0016043 | Biological Process | 118 | Cellular component organization | NUDT21,API5,KIF5B,VPS35,NAPA,HSPA9,ADAMTS7,VASP,LMNB1,MMP14,ACTR2,FBLN2,LAMB1,PLIN3,SGTA,LGMN,CSPG4,CRK,COL5A2,PTK7,MANF,SMPD1,PLS3,PSMD5,SEC22B,KPNB1,PAFAH1B1,UGDH,ALDH1A2,CORO1B,AP2A2,RPLP0,DPT,SPTBN1,NID1,QSOX1,CDKL3,SEC31A,EIF2S1,GRN,ALCAM,ARPC5,AREL1,DSTN,CNN3,TFG,FERMT2,TGFBI,CCT3,PDCD6,RPL5,MMP1,APEX1,PLOD3,ENAH,SNRPD3,LOXL1,LAMC1,SEPTIN6,TWF1,PUF60,LOXL2,EMILIN1,DYNC1H1,LAMA2,EIF3E,LIPG,MYOF,SDCBP,SNX9,BSG,CNN2,CDC42,F11R,CLTC,RANBP1,FKBP10,RAB7A,LMAN2,NDRG1,SFPQ,G3BP1,VAPA,CAPZB,SEC23A,DPYSL3,FUS,DBN1,SGCA,DCTN2,GLRX3,CCT2,TNC,SEPTIN7,LAMA5,VCP,MAP4,PARVA,IQGAP1,PXDN,ARPC2,COL4A2,FOLR1,MARCKS,PSMD13,PDLIM5,SPTAN1,FLNC,DBNL,PITPNB,PPP2R1A,PRMT1,PLEC,HEXA,ARPC1B,FLNB,DDX39B,ADAMTS5 | 0.19 | 0.48 | 0.00023 |
| GO:0043484 | Biological Process | 13 | Regulation of RNA splicing | HNRNPF,HNRNPH3,DDX5,HNRNPH1,PTBP1,RBM8A,PUF60,U2AF2,DDX17,FUS,HNRNPK,AHNAK,ENSECAP00000050120 | 0.81 | 0.8 | 0.00023 |
| GO:0051641 | Biological Process | 67 | Cellular localization | ERP29,KIF5B,VPS35,ARF4,CD81,NAPA,HSPA9,LMNB1,SLC39A14,GDI2,CPE,AP2B1,VPS26A,SGTA,HYOU1,SEC22B,COPG2,KPNB1,PAFAH1B1,IPO5,RRBP1,CORO1B,AP2A2,SPTBN1,SEC31A,TCP1,GRN,ARCN1,FBN1,TFG,FERMT2,PDCD6,COPA,PLOD3,SEPTIN6,TWF1,DYNC1H1,SNX9,CNN2,F11R,CLTC,RANBP1,RAB7A,LMAN2,SEPTIN9,RAB11A,SFPQ,STAM,SEC23A,COPB1,HNRNPA2B1,SEPTIN8,DBN1,SEPTIN11,AP1B1,VPS29,DCTN2,GDI1,COPB2,SEPTIN7,VCP,COPG1,SCARB2,SRI,PLEC,HEXA,DDX39B | 0.28 | 0.53 | 0.00023 |
| GO:0033043 | Biological Process | 37 | Regulation of organelle organization | VPS35,VASP,GDI2,TRIM28,CRK,VDAC1,SEC22B,PAFAH1B1,CORO1B,SPTBN1,CCN2,VAT1,CCT7,TCP1,GRN,ARPC5,DSTN,FERMT2,CCT3,TWF1,DYNC1H1,SNX9,F11R,CLTC,SEPTIN9,CCT4,SFPQ,STAM,G3BP1,CAPZB,CCT2,IQGAP1,ARPC2,SPTAN1,DBNL,CAPN2,ARPC1B | 0.4 | 0.59 | 0.00033 |
| GO:0071840 | Biological Process | 120 | Cellular component organization or biogenesis | NUDT21,API5,KIF5B,VPS35,NAPA,HSPA9,ADAMTS7,VASP,LMNB1,MMP14,ACTR2,FBLN2,LAMB1,PLIN3,SGTA,LGMN,CSPG4,CRK,COL5A2,PTK7,MANF,SMPD1,PLS3,PSMD5,RPL10A,SEC22B,KPNB1,PAFAH1B1,UGDH,ALDH1A2,CORO1B,AP2A2,RPLP0,DPT,SPTBN1,NID1,QSOX1,CDKL3,SEC31A,EIF2S1,GRN,ALCAM,ARPC5,AREL1,DSTN,CNN3,TFG,FERMT2,TGFBI,CCT3,PDCD6,RPL5,MMP1,APEX1,PLOD3,ENAH,SNRPD3,LOXL1,LAMC1,SEPTIN6,TWF1,PUF60,LOXL2,EMILIN1,DYNC1H1,LAMA2,EIF3E,LIPG,MYOF,SDCBP,SNX9,BSG,CNN2,CDC42,F11R,CLTC,RANBP1,FKBP10,RAB7A,LMAN2,NDRG1,SFPQ,G3BP1,VAPA,CAPZB,SEC23A,DPYSL3,FUS,DBN1,SGCA,DCTN2,GLRX3,CCT2,TNC,SEPTIN7,LAMA5,VCP,MAP4,PARVA,IQGAP1,PXDN,ARPC2,COL4A2,FOLR1,MARCKS,PSMD13,PDLIM5,SPTAN1,FLNC,DBNL,PITPNB,PPP2R1A,PRMT1,PLEC,WDR43,HEXA,ARPC1B,FLNB,DDX39B,ADAMTS5 | 0.18 | 0.46 | 0.00040 |
| GO:1904851 | Biological Process | 5 | Positive regulation of establishment of protein localization to telomere | CCT7,TCP1,CCT3,CCT4,CCT2 | 1.66 | 0.91 | 0.00040 |
| GO:0006418 | Biological Process | 8 | tRNA aminoacylation for protein translation | YARS1,GARS1,SARS1,TARS1,RARS1,NARS1,WARS1,AARS1 | 1.09 | 0.83 | 0.00043 |
| GO:0032970 | Biological Process | 19 | Regulation of actin filament-based process | VASP,CRK,CORO1B,SPTBN1,CCN2,ARPC5,DSTN,FERMT2,TWF1,SNX9,CNN2,F11R,CAPZB,IQGAP1,ARPC2,SRI,SPTAN1,DBNL,ARPC1B | 0.6 | 0.66 | 0.00045 |
| GO:0006082 | Biological Process | 31 | Organic acid metabolic process | PTGR1,PLOD2,YARS1,SEPHS1,UGDH,ALDH1A2,GOT2,GCLC,PSAT1,GPI,ENOPH1,GARS1,SARS1,TARS1,ALOX12,PLOD3,GNPDA1,MDH2,RARS1,LIPG,NARS1,WARS1,ADH5,PFKP,ACLY,AKR1B1,ALDOA,GNPDA2,PXDN,AARS1,FASN | 0.43 | 0.59 | 0.00047 |
| GO:0043603 | Biological Process | 32 | Cellular amide metabolic process | NPEPPS,PCSK5,CPE,YARS1,RPL12,RPS4X,HEXB,THOP1,SMPD1,RPL10A,G6PD,RPLP0,GCLC,EIF2S1,GSTM3,GARS1,SARS1,EEF1A1,RPL5,TARS1,GNPDA1,LOC100058329,RARS1,LOC100058290,EIF3E,NARS1,WARS1,ACLY,GNPDA2,NCSTN,AARS1,HEXA | 0.42 | 0.58 | 0.00049 |
| GO:0019752 | Biological Process | 30 | Carboxylic acid metabolic process | PTGR1,PLOD2,YARS1,SEPHS1,UGDH,ALDH1A2,GOT2,GCLC,PSAT1,GPI,ENOPH1,GARS1,SARS1,TARS1,ALOX12,PLOD3,GNPDA1,MDH2,RARS1,LIPG,NARS1,WARS1,ADH5,PFKP,ACLY,AKR1B1,ALDOA,GNPDA2,AARS1,FASN | 0.44 | 0.59 | 0.00050 |
| GO:0006518 | Biological Process | 26 | Peptide metabolic process | NPEPPS,PCSK5,CPE,YARS1,RPL12,RPS4X,THOP1,RPL10A,G6PD,RPLP0,GCLC,EIF2S1,GSTM3,GARS1,SARS1,EEF1A1,RPL5,TARS1,LOC100058329,RARS1,LOC100058290,EIF3E,NARS1,WARS1,NCSTN,AARS1 | 0.47 | 0.59 | 0.00059 |
| GO:0046907 | Biological Process | 41 | Intracellular transport | KIF5B,VPS35,ARF4,NAPA,HSPA9,LMNB1,AP2B1,VPS26A,SGTA,HYOU1,SEC22B,COPG2,KPNB1,PAFAH1B1,IPO5,AP2A2,SEC31A,GRN,ARCN1,TFG,PDCD6,COPA,DYNC1H1,SNX9,CLTC,RANBP1,RAB7A,LMAN2,RAB11A,SFPQ,STAM,SEC23A,COPB1,HNRNPA2B1,AP1B1,VPS29,COPB2,VCP,COPG1,SCARB2,DDX39B | 0.35 | 0.54 | 0.00059 |
| GO:0009056 | Biological Process | 51 | Catabolic process | ELOC,NPEPPS,ADAMTS7,MMP14,PLBD2,SGTA,LGMN,DDX5,HEXB,SND1,DDB1,SMPD1,MMP9,PAFAH1B1,UBA1,GOT2,PRDX5,KHSRP,GPI,GSTM3,PSMA2,MMP1,PSMB1,RBM8A,APEX1,PSMA6,CTSS,PSMD2,IMPA1,GNPDA1,HINT1,PSMC3,EIF3E,LIPG,UCHL1,PGM2,CLTC,RAB7A,ADH5,PFKP,STAM,ALDOA,GNPDA2,VCP,PXDN,PABPC1,CAT,PSMD13,CAPN2,HEXA,PSMD6 | 0.29 | 0.48 | 0.0012 |
| GO:0016192 | Biological Process | 39 | Vesicle-mediated transport | VPS35,ARF4,CD81,NAPA,GDI2,AP2B1,VPS26A,HYOU1,SEC22B,COPG2,AP2A2,CXCL8,SEC31A,ARCN1,TFG,PDCD6,COPA,LOXL2,SNX9,CNN2,CDC42,CLTC,RAB7A,LMAN2,RAB11A,STAM,SEC23A,COPB1,AP1B1,VPS29,GDI1,COPB2,VCP,COPG1,PDCD6IP,ITGAV,FOLR1,SCARB2,DBNL | 0.35 | 0.5 | 0.0012 |
| GO:0006886 | Biological Process | 26 | Intracellular protein transport | VPS35,ARF4,NAPA,HSPA9,AP2B1,VPS26A,SGTA,COPG2,KPNB1,IPO5,AP2A2,SEC31A,PDCD6,COPA,SNX9,CLTC,RAB7A,STAM,SEC23A,COPB1,AP1B1,VPS29,COPB2,VCP,COPG1,SCARB2 | 0.45 | 0.54 | 0.0013 |
| GO:0110053 | Biological Process | 15 | Regulation of actin filament organization | VASP,CORO1B,SPTBN1,CCN2,ARPC5,DSTN,FERMT2,TWF1,SNX9,F11R,CAPZB,ARPC2,SPTAN1,DBNL,ARPC1B | 0.63 | 0.6 | 0.0015 |
| GO:0007010 | Biological Process | 38 | Cytoskeleton organization | API5,VASP,LMNB1,ACTR2,CRK,PTK7,PLS3,KPNB1,PAFAH1B1,CORO1B,SPTBN1,ARPC5,DSTN,CNN3,ENAH,TWF1,DYNC1H1,SDCBP,CNN2,CDC42,F11R,RANBP1,CAPZB,DPYSL3,DBN1,DCTN2,VCP,MAP4,PARVA,ARPC2,MARCKS,PDLIM5,SPTAN1,FLNC,DBNL,PLEC,ARPC1B,FLNB | 0.35 | 0.49 | 0.0016 |
| GO:0032956 | Biological Process | 17 | Regulation of actin cytoskeleton organization | VASP,CRK,CORO1B,SPTBN1,CCN2,ARPC5,DSTN,FERMT2,TWF1,SNX9,F11R,CAPZB,IQGAP1,ARPC2,SPTAN1,DBNL,ARPC1B | 0.58 | 0.58 | 0.0016 |
| GO:0048519 | Biological Process | 100 | Negative regulation of biological process | HTRA3,VPS35,PA2G4,PLAU,HSPA9,ADAMTS7,LMNB1,MMP14,ECM1,GDI2,SGTA,LGMN,TRIM28,SERPING1,HDGF,PPP2CB,DDX5,CRK,COL5A2,SERPINB9,SND1,VDAC1,RTRAF,DDB1,SMPD1,IGFBP5,HYOU1,SEC22B,PAFAH1B1,G6PD,ALDH1A2,CORO1B,CXCL8,TSN,DPT,SPTBN1,CCN2,VAT1,QSOX1,PRDX5,GCLC,ILF3,CDKL3,EIF2S1,HNRNPAB,GRN,KHSRP,PTBP1,SERPINE1,FBN1,SARS1,FERMT2,PDCD6,MMP1,RBM8A,ALOX12,FHL2,INHBA,CRLF1,TWF1,LOXL2,MVP,EMILIN1,EIF3E,NIBAN2,CNN2,F11R,CLTC,NXN,RAB7A,WARS1,U2AF2,NDRG1,SFPQ,SYNCRIP,G3BP1,CAPZB,DPYSL3,ERO1A,FUS,SGCA,GLRX3,GDI1,SERPINB8,IQGAP1,IFI30,PABPC1,ITGAV,COL4A2,CAT,SERPINB1,AARS1,PLAT,SRI,SPTAN1,PRMT1,FSTL1,DDX39B,ADAMTS5,RNASE4 | 0.18 | 0.42 | 0.0016 |
| GO:0051128 | Biological Process | 53 | Regulation of cellular component organization | VPS35,NAPA,VASP,MMP14,GDI2,TRIM28,CRK,PTK7,VDAC1,SMPD1,IGFBP7,SEC22B,PAFAH1B1,CORO1B,SPTBN1,CCN2,VAT1,CCT7,CDKL3,TCP1,GRN,ARPC5,DSTN,FERMT2,CCT3,INHBA,TWF1,EMILIN1,DYNC1H1,SNX9,CDC42,F11R,CLTC,WARS1,SEPTIN9,CCT4,SFPQ,STAM,G3BP1,CAPZB,DPYSL3,SEPTIN8,DBN1,CCT2,GDI1,TNC,IQGAP1,ITGAV,ARPC2,SPTAN1,DBNL,CAPN2,ARPC1B | 0.28 | 0.46 | 0.0016 |
| GO:0009988 | Biological Process | 8 | Cell-cell recognition | CD81,CCT7,TCP1,CCT3,CCT4,CCT2,ALDOA,FOLR1 | 0.96 | 0.67 | 0.0017 |
| GO:0009653 | Biological Process | 52 | Anatomical structure morphogenesis | KIF5B,CD81,VASP,PCSK5,MMP14,LAMB1,CPE,TRIM28,COL5A2,PTK7,PAFAH1B1,UGDH,ALDH1A2,SPTBN1,CCN2,CDKL3,GPI,ALCAM,FBN1,TGFBI,PDCD6,MMP1,FHL2,INHBA,PLOD3,ENAH,PRCP,LAMC1,LOXL2,EMILIN1,LAMA2,MYOF,BSG,CDC42,FKBP10,CAPZB,SGCA,TNC,LAMA5,PARVA,IQGAP1,PXDN,ITGAV,COL4A2,FLNC,DBNL,CAPN2,PLEC,HEXA,ADAMTS5,RNASE4,HSPG2 | 0.28 | 0.45 | 0.0019 |
| GO:0015031 | Biological Process | 34 | Protein transport | ERP29,VPS35,ARF4,NAPA,HSPA9,GDI2,AP2B1,VPS26A,SGTA,COPG2,KPNB1,PAFAH1B1,IPO5,RRBP1,AP2A2,SEC31A,TCP1,ARCN1,PDCD6,COPA,SNX9,CLTC,RAB7A,RAB11A,STAM,SEC23A,COPB1,AP1B1,VPS29,GDI1,COPB2,VCP,COPG1,SCARB2 | 0.37 | 0.49 | 0.0019 |
| GO:0044281 | Biological Process | 44 | Small molecule metabolic process | PTGR1,SLC39A14,PLOD2,YARS1,SMPD1,SEPHS1,UGDH,ATIC,G6PD,ALDH1A2,OLA1,GOT2,GCLC,PSAT1,GPI,ENOPH1,GARS1,PAICS,FBN1,SARS1,TARS1,ALOX12,PLOD3,IMPA1,GNPDA1,HINT1,MDH2,PGM3,RARS1,LIPG,NARS1,PGM2,NAMPT,WARS1,ADH5,PFKP,ACLY,AKR1B1,ALDOA,GNPDA2,PXDN,CAT,AARS1,FASN | 0.31 | 0.47 | 0.0019 |
| GO:0071704 | Biological Process | 155 | Organic substance metabolic process | ELOC,HTRA3,NUDT21,PTGR1,PLAU,NPEPPS,ADAMTS7,PCSK5,SLC39A14,MMP14,HNRNPA1,PLBD2,CPE,PLOD2,YARS1,SGTA,PPP1CA,LGMN,RPL12,TRIM28,RPS4X,PPP2CB,DDX5,CRK,PTK7,HEXB,THOP1,SND1,RTRAF,HNRNPL,DDB1,SMPD1,MMP9,RPL10A,SEPHS1,PAFAH1B1,UBA1,UGDH,ATIC,GANAB,G6PD,ALDH1A2,RPLP0,TSN,OLA1,PPIA,CCN2,GOT2,ERP44,PPP1CC,GCLC,ILF3,CDKL3,PSAT1,FKBP3,EIF2S1,KHSRP,GPI,GSTM3,PTBP1,ENOPH1,GARS1,PAICS,FBN1,SARS1,EEF1A1,PSMA2,RPL5,TARS1,MMP1,PSMB1,PCOLCE,DNPEP,RBM8A,ALOX12,BMP1,APEX1,PSMA6,INHBA,PLOD3,CTSS,PSMD2,PAPPA,IMPA1,GNPDA1,HINT1,PLOD1,SNRPD3,MDH2,HNRNPA3,PRCP,LOXL1,PGM3,TWF1,HNRNPM,LOC100058329,RARS1,PUF60,PSMC3,LOC100058290,LOXL2,EIF3E,LIPG,NARS1,UCHL1,PGM2,NAMPT,FKBP10,RAB7A,WARS1,ADH5,U2AF2,PFKP,ACLY,HTRA1,SFPQ,STAM,SCPEP1,G3BP1,DDX17,AKR1B1,NANS,PI4KA,FUS,HNRNPK,HNRNPA2B1,GLRX3,LAP3,ALDOA,GNPDA2,VCP,PXDN,GNS,NCSTN,PABPC1,ITGAV,COL4A2,CAT,PSMD13,AARS1,PLAT,SCARB2,TALDO1,DPP3,CAPN2,PPP2R1A,PRMT1,PLEC,WDR43,HEXA,FASN,PSMD6,DDX39B,ADAMTS5,RNASE4 | 0.13 | 0.39 | 0.0019 |
| GO:0006520 | Biological Process | 15 | Cellular amino acid metabolic process | PLOD2,YARS1,SEPHS1,GOT2,GCLC,PSAT1,ENOPH1,GARS1,SARS1,TARS1,PLOD3,RARS1,NARS1,WARS1,AARS1 | 0.61 | 0.57 | 0.0021 |
| GO:0051493 | Biological Process | 21 | Regulation of cytoskeleton organization | VASP,CRK,PAFAH1B1,CORO1B,SPTBN1,CCN2,ARPC5,DSTN,FERMT2,TWF1,DYNC1H1,SNX9,F11R,CLTC,CAPZB,IQGAP1,ARPC2,SPTAN1,DBNL,CAPN2,ARPC1B | 0.49 | 0.52 | 0.0025 |
| GO:0061077 | Biological Process | 8 | Chaperone-mediated protein folding | HSPA9,CCT7,FKBP3,TCP1,HSPH1,CCT3,CCT4,CCT2 | 0.92 | 0.63 | 0.0025 |
| GO:0007015 | Biological Process | 15 | Actin filament organization | API5,VASP,ACTR2,PLS3,CORO1B,ARPC5,DSTN,ENAH,TWF1,CDC42,DPYSL3,DBN1,ARPC2,MARCKS,ARPC1B | 0.59 | 0.54 | 0.0030 |
| GO:1901564 | Biological Process | 106 | Organonitrogen compound metabolic process | ELOC,HTRA3,PLAU,NPEPPS,ADAMTS7,PCSK5,MMP14,CPE,PLOD2,YARS1,SGTA,PPP1CA,LGMN,RPL12,TRIM28,RPS4X,PPP2CB,PTK7,HEXB,THOP1,DDB1,SMPD1,MMP9,RPL10A,SEPHS1,UBA1,UGDH,ATIC,GANAB,G6PD,RPLP0,OLA1,PPIA,GOT2,ERP44,GCLC,ILF3,CDKL3,PSAT1,FKBP3,EIF2S1,GPI,GSTM3,ENOPH1,GARS1,PAICS,SARS1,EEF1A1,PSMA2,RPL5,TARS1,MMP1,PSMB1,PCOLCE,DNPEP,BMP1,PSMA6,INHBA,PLOD3,CTSS,PSMD2,PAPPA,GNPDA1,HINT1,PLOD1,PRCP,LOXL1,TWF1,LOC100058329,RARS1,PSMC3,LOC100058290,LOXL2,EIF3E,NARS1,UCHL1,PGM2,NAMPT,FKBP10,RAB7A,WARS1,ADH5,PFKP,ACLY,HTRA1,SFPQ,STAM,SCPEP1,GLRX3,LAP3,ALDOA,GNPDA2,VCP,GNS,NCSTN,CAT,PSMD13,AARS1,PLAT,DPP3,CAPN2,PPP2R1A,PRMT1,HEXA,PSMD6,ADAMTS5 | 0.16 | 0.38 | 0.0034 |
| GO:0035036 | Biological Process | 7 | Sperm-egg recognition | CCT7,TCP1,CCT3,CCT4,CCT2,ALDOA,FOLR1 | 0.99 | 0.61 | 0.0036 |
| GO:0006508 | Biological Process | 39 | Proteolysis | ELOC,HTRA3,PLAU,NPEPPS,ADAMTS7,PCSK5,MMP14,CPE,SGTA,LGMN,THOP1,DDB1,MMP9,UBA1,PSMA2,MMP1,PSMB1,PCOLCE,DNPEP,BMP1,PSMA6,CTSS,PSMD2,PAPPA,PRCP,PSMC3,UCHL1,HTRA1,STAM,SCPEP1,LAP3,VCP,NCSTN,PSMD13,PLAT,DPP3,CAPN2,PSMD6,ADAMTS5 | 0.32 | 0.44 | 0.0037 |
| GO:0044238 | Biological Process | 147 | Primary metabolic process | ELOC,HTRA3,NUDT21,PTGR1,PLAU,NPEPPS,ADAMTS7,PCSK5,SLC39A14,MMP14,HNRNPA1,PLBD2,CPE,PLOD2,YARS1,SGTA,PPP1CA,LGMN,RPL12,TRIM28,RPS4X,PPP2CB,DDX5,CRK,PTK7,HEXB,THOP1,SND1,RTRAF,HNRNPL,DDB1,SMPD1,MMP9,RPL10A,SEPHS1,PAFAH1B1,UBA1,UGDH,ATIC,GANAB,G6PD,ALDH1A2,RPLP0,TSN,OLA1,PPIA,CCN2,GOT2,ERP44,PPP1CC,GCLC,ILF3,CDKL3,PSAT1,FKBP3,EIF2S1,KHSRP,GPI,PTBP1,ENOPH1,GARS1,PAICS,FBN1,SARS1,EEF1A1,PSMA2,RPL5,TARS1,MMP1,PSMB1,PCOLCE,DNPEP,RBM8A,ALOX12,BMP1,APEX1,PSMA6,INHBA,PLOD3,CTSS,PSMD2,PAPPA,IMPA1,GNPDA1,HINT1,PLOD1,SNRPD3,MDH2,HNRNPA3,PRCP,LOXL1,PGM3,TWF1,HNRNPM,RARS1,PUF60,PSMC3,LOXL2,EIF3E,LIPG,NARS1,UCHL1,PGM2,NAMPT,FKBP10,RAB7A,WARS1,ADH5,U2AF2,PFKP,ACLY,HTRA1,SFPQ,STAM,SCPEP1,G3BP1,DDX17,AKR1B1,NANS,PI4KA,FUS,HNRNPK,HNRNPA2B1,GLRX3,LAP3,ALDOA,GNPDA2,VCP,NCSTN,PABPC1,COL4A2,CAT,PSMD13,AARS1,PLAT,TALDO1,DPP3,CAPN2,PPP2R1A,PRMT1,WDR43,HEXA,FASN,PSMD6,DDX39B,ADAMTS5,RNASE4 | 0.13 | 0.36 | 0.0038 |
| GO:0005975 | Biological Process | 19 | Carbohydrate metabolic process | SLC39A14,PPP1CA,HEXB,UGDH,GANAB,G6PD,PPP1CC,GPI,FBN1,IMPA1,GNPDA1,PGM3,PGM2,PFKP,NANS,ALDOA,GNPDA2,TALDO1,HEXA | 0.5 | 0.49 | 0.0041 |
| GO:0035987 | Biological Process | 6 | Endodermal cell differentiation | MMP14,LAMB1,MMP1,INHBA,ITGAV,COL4A2 | 1.09 | 0.61 | 0.0041 |
| GO:0010770 | Biological Process | 7 | Positive regulation of cell morphogenesis involved in differentiation | CRK,PAFAH1B1,CDKL3,FERMT2,CDC42,DBN1,DBNL | 0.97 | 0.59 | 0.0042 |
| GO:0051130 | Biological Process | 29 | Positive regulation of cellular component organization | VPS35,VASP,TRIM28,PTK7,SMPD1,PAFAH1B1,CORO1B,CCN2,CCT7,CDKL3,TCP1,GRN,DSTN,FERMT2,CCT3,TWF1,EMILIN1,DYNC1H1,SNX9,CDC42,WARS1,SEPTIN9,CCT4,SFPQ,G3BP1,DPYSL3,DBN1,CCT2,DBNL | 0.38 | 0.45 | 0.0042 |
| GO:0008064 | Biological Process | 11 | Regulation of actin polymerization or depolymerization | VASP,SPTBN1,ARPC5,DSTN,TWF1,SNX9,CAPZB,ARPC2,SPTAN1,DBNL,ARPC1B | 0.7 | 0.53 | 0.0045 |
| GO:1990748 | Biological Process | 9 | Cellular detoxification | SOD2,PRDX5,GSTM3,NXN,ADH5,TXNL1,PXDN,CAT,TXNDC17 | 0.8 | 0.55 | 0.0046 |
| GO:0006891 | Biological Process | 6 | intra-Golgi vesicle-mediated transport | COPG2,ARCN1,COPA,COPB1,COPB2,COPG1 | 1.06 | 0.58 | 0.0049 |
| GO:0010467 | Biological Process | 52 | Gene expression | NUDT21,PLAU,PCSK5,MMP14,HNRNPA1,CPE,YARS1,LGMN,RPL12,RPS4X,DDX5,RTRAF,HNRNPL,RPL10A,RPLP0,TSN,EIF2S1,PTBP1,GARS1,SARS1,EEF1A1,RPL5,TARS1,RBM8A,BMP1,INHBA,SNRPD3,HNRNPA3,HNRNPM,RARS1,PUF60,EIF3E,NARS1,WARS1,U2AF2,SFPQ,DDX17,FUS,HNRNPK,HNRNPA2B1,GLRX3,NCSTN,PABPC1,ITGAV,COL4A2,AARS1,PLAT,SCARB2,PRMT1,WDR43,DDX39B,RNASE4 | 0.26 | 0.4 | 0.0049 |
| GO:0016477 | Biological Process | 27 | Cell migration | PLAU,MMP14,LAMB1,CSPG4,CRK,PTK7,CXCL6,MMP9,PAFAH1B1,CORO1B,CXCL8,CCN2,ARPC5,LAMC1,LOXL2,EMILIN1,NARS1,CNN2,F11R,SGCA,LAMA5,PARVA,IQGAP1,ITGAV,PLAT,FSTL1,RNASE4 | 0.39 | 0.45 | 0.0049 |
| GO:0051649 | Biological Process | 44 | Establishment of localization in cell | KIF5B,VPS35,ARF4,NAPA,HSPA9,LMNB1,SLC39A14,AP2B1,VPS26A,SGTA,HYOU1,SEC22B,COPG2,KPNB1,PAFAH1B1,IPO5,AP2A2,SEC31A,GRN,ARCN1,TFG,PDCD6,COPA,DYNC1H1,SNX9,CNN2,CLTC,RANBP1,RAB7A,LMAN2,RAB11A,SFPQ,STAM,SEC23A,COPB1,HNRNPA2B1,AP1B1,VPS29,DCTN2,COPB2,VCP,COPG1,SCARB2,DDX39B | 0.28 | 0.4 | 0.0057 |
| GO:1901575 | Biological Process | 43 | Organic substance catabolic process | ELOC,NPEPPS,ADAMTS7,PLBD2,SGTA,LGMN,DDX5,HEXB,SND1,DDB1,SMPD1,PAFAH1B1,UBA1,GOT2,KHSRP,GPI,PSMA2,PSMB1,RBM8A,APEX1,PSMA6,CTSS,PSMD2,IMPA1,GNPDA1,HINT1,PSMC3,EIF3E,LIPG,UCHL1,PGM2,RAB7A,ADH5,PFKP,STAM,ALDOA,GNPDA2,VCP,PABPC1,PSMD13,CAPN2,HEXA,PSMD6 | 0.29 | 0.4 | 0.0059 |
| GO:0022603 | Biological Process | 25 | Regulation of anatomical structure morphogenesis | VPS35,ECM1,CRK,PTK7,PAFAH1B1,CORO1B,CXCL8,VAT1,CDKL3,SERPINE1,SARS1,FERMT2,PDCD6,EMILIN1,RNH1,CDC42,F11R,WARS1,CAPZB,DBN1,GDI1,ALDOA,SEPTIN7,COL4A2,DBNL | 0.4 | 0.44 | 0.0060 |
| GO:0085029 | Biological Process | 6 | Extracellular matrix assembly | LAMB1,QSOX1,PLOD3,EMILIN1,FKBP10,PXDN | 1.04 | 0.56 | 0.0060 |
| GO:1901998 | Biological Process | 6 | Toxin transport | CCT7,TCP1,CCT3,CCT4,CCT2,COPB2 | 1.04 | 0.56 | 0.0060 |
| GO:0048646 | Biological Process | 26 | Anatomical structure formation involved in morphogenesis | CD81,VASP,MMP14,LAMB1,PTK7,PAFAH1B1,ALDH1A2,CCN2,GPI,TGFBI,PDCD6,MMP1,FHL2,INHBA,PRCP,LOXL2,MYOF,SGCA,PARVA,PXDN,ITGAV,COL4A2,FLNC,CAPN2,ADAMTS5,RNASE4 | 0.39 | 0.42 | 0.0069 |
| GO:0048731 | Biological Process | 71 | System development | KIF5B,CD81,HSPA9,VASP,PCSK5,SLC39A14,MMP14,ECM1,LAMB1,CPE,CSPG4,TRIM28,CRK,COL5A2,PTK7,MANF,PLS3,PAFAH1B1,UGDH,G6PD,ALDH1A2,CXCL8,SPTBN1,CCN2,NID1,CDKL3,ALCAM,ARCN1,FBN1,MXRA8,TGFBI,FHL2,INHBA,PLOD3,ENAH,PRCP,CRLF1,PGM3,LOXL2,EMILIN1,LAMA2,NARS1,BSG,CNN2,CDC42,PABPC4,NXN,FKBP10,NDRG1,CRIP2,DBN1,SGCA,AP1B1,TNC,LAMA5,MAP4,PARVA,IQGAP1,PXDN,NCSTN,ITGAV,MARCKS,AARS1,PDLIM5,DBNL,PRMT1,PLEC,HEXA,FASN,RNASE4,HSPG2 | 0.2 | 0.37 | 0.0069 |
| GO:1903311 | Biological Process | 14 | Regulation of mRNA metabolic process | NUDT21,DDX5,HNRNPAB,PTBP1,RBM8A,APEX1,PUF60,PABPC4,U2AF2,SYNCRIP,DDX17,FUS,HNRNPK,PABPC1 | 0.57 | 0.47 | 0.0069 |
| GO:1902903 | Biological Process | 16 | Regulation of supramolecular fiber organization | VASP,CORO1B,SPTBN1,CCN2,ARPC5,DSTN,FERMT2,TWF1,EMILIN1,SNX9,F11R,CAPZB,ARPC2,SPTAN1,DBNL,ARPC1B | 0.52 | 0.46 | 0.0070 |
| GO:0007275 | Biological Process | 77 | Multicellular organism development | KIF5B,CD81,HSPA9,VASP,PCSK5,SLC39A14,MMP14,ECM1,LAMB1,CPE,CSPG4,TRIM28,CRK,COL5A2,PTK7,MANF,PLS3,PAFAH1B1,UGDH,G6PD,ALDH1A2,CXCL8,SPTBN1,CCN2,NID1,CDKL3,GPI,ALCAM,ARCN1,FBN1,MXRA8,TGFBI,PDCD6,MMP1,FHL2,BMP1,INHBA,PLOD3,ENAH,PRCP,CRLF1,PGM3,LOXL2,EMILIN1,LAMA2,NARS1,BSG,CNN2,CDC42,PABPC4,NXN,FKBP10,NDRG1,CRIP2,DBN1,SGCA,AP1B1,TNC,LAMA5,MAP4,PARVA,IQGAP1,PXDN,NCSTN,ITGAV,COL4A2,MARCKS,AARS1,PDLIM5,DBNL,CAPN2,PRMT1,PLEC,HEXA,FASN,RNASE4,HSPG2 | 0.19 | 0.36 | 0.0071 |
| GO:0007339 | Biological Process | 6 | Binding of sperm to zona pellucida | CCT7,TCP1,CCT3,CCT4,CCT2,ALDOA | 1.01 | 0.54 | 0.0071 |
| GO:0017185 | Biological Process | 4 | Peptidyl-lysine hydroxylation | PLOD2,PLOD3,PLOD1,FKBP10 | 1.41 | 0.57 | 0.0071 |
| GO:0030199 | Biological Process | 6 | Collagen fibril organization | COL5A2,PLOD3,LOXL1,LOXL2,FKBP10,PXDN | 1.01 | 0.54 | 0.0071 |
| GO:0030833 | Biological Process | 10 | Regulation of actin filament polymerization | VASP,SPTBN1,ARPC5,TWF1,SNX9,CAPZB,ARPC2,SPTAN1,DBNL,ARPC1B | 0.71 | 0.5 | 0.0071 |
| GO:0032212 | Biological Process | 5 | Positive regulation of telomere maintenance via telomerase | CCT7,TCP1,CCT3,CCT4,CCT2 | 1.16 | 0.56 | 0.0071 |
| GO:0071705 | Biological Process | 40 | Nitrogen compound transport | ERP29,VPS35,ARF4,NAPA,HSPA9,GDI2,CPE,AP2B1,VPS26A,SGTA,COPG2,KPNB1,PAFAH1B1,IPO5,RRBP1,AP2A2,SEC31A,TCP1,ARCN1,PDCD6,RBM8A,COPA,SNX9,CLTC,RAB7A,RAB11A,STAM,SLC44A1,SEC23A,COPB1,HNRNPA2B1,AP1B1,VPS29,GDI1,COPB2,VCP,COPG1,FOLR1,SCARB2,DDX39B | 0.29 | 0.39 | 0.0072 |
| GO:0008152 | Biological Process | 160 | Metabolic process | ELOC,HTRA3,NUDT21,PTGR1,PLAU,HSPA9,NPEPPS,ADAMTS7,PCSK5,SLC39A14,MMP14,HNRNPA1,PLBD2,CPE,PLOD2,YARS1,SGTA,PPP1CA,LGMN,RPL12,TRIM28,RPS4X,PPP2CB,DDX5,CRK,PTK7,SOD2,HEXB,THOP1,SND1,RTRAF,HNRNPL,DDB1,SMPD1,MMP9,RPL10A,SEPHS1,PAFAH1B1,UBA1,UGDH,ATIC,GANAB,G6PD,ALDH1A2,RPLP0,TSN,OLA1,PPIA,CCN2,GOT2,ERP44,PPP1CC,PRDX5,GCLC,ILF3,CDKL3,PSAT1,FKBP3,EIF2S1,KHSRP,GPI,GSTM3,PTBP1,ENOPH1,GARS1,SERPINE1,PAICS,FBN1,SARS1,EEF1A1,PSMA2,RPL5,TARS1,MMP1,PSMB1,PCOLCE,DNPEP,RBM8A,ALOX12,BMP1,APEX1,PSMA6,INHBA,PLOD3,CTSS,PSMD2,PAPPA,IMPA1,GNPDA1,HINT1,PLOD1,SNRPD3,MDH2,HNRNPA3,PRCP,LOXL1,PGM3,TWF1,HNRNPM,LOC100058329,RARS1,PUF60,PSMC3,LOC100058290,LOXL2,EIF3E,LIPG,NARS1,UCHL1,PGM2,CLTC,NAMPT,FKBP10,RAB7A,WARS1,ADH5,U2AF2,PFKP,ACLY,HTRA1,SFPQ,STAM,SCPEP1,G3BP1,DDX17,AKR1B1,NANS,PI4KA,FUS,HNRNPK,HNRNPA2B1,GLRX3,LAP3,ALDOA,GNPDA2,VCP,PXDN,GNS,NCSTN,PABPC1,ITGAV,COL4A2,CAT,PSMD13,AARS1,PLAT,SCARB2,TALDO1,DPP3,CAPN2,PPP2R1A,PRMT1,PLEC,WDR43,HEXA,FASN,PSMD6,DDX39B,ADAMTS5,RNASE4 | 0.11 | 0.33 | 0.0075 |
| GO:0008380 | Biological Process | 16 | RNA splicing | HNRNPA1,DDX5,RTRAF,PTBP1,RBM8A,SNRPD3,HNRNPA3,HNRNPM,PUF60,U2AF2,SFPQ,DDX17,FUS,PABPC1,PRMT1,DDX39B | 0.51 | 0.45 | 0.0076 |
| GO:0006807 | Biological Process | 134 | Nitrogen compound metabolic process | ELOC,HTRA3,NUDT21,PLAU,NPEPPS,ADAMTS7,PCSK5,MMP14,HNRNPA1,CPE,PLOD2,YARS1,SGTA,PPP1CA,LGMN,RPL12,TRIM28,RPS4X,PPP2CB,DDX5,PTK7,HEXB,THOP1,SND1,RTRAF,HNRNPL,DDB1,SMPD1,MMP9,RPL10A,SEPHS1,UBA1,UGDH,ATIC,GANAB,G6PD,RPLP0,TSN,OLA1,PPIA,CCN2,GOT2,ERP44,GCLC,ILF3,CDKL3,PSAT1,FKBP3,EIF2S1,KHSRP,GPI,GSTM3,PTBP1,ENOPH1,GARS1,PAICS,SARS1,EEF1A1,PSMA2,RPL5,TARS1,MMP1,PSMB1,PCOLCE,DNPEP,RBM8A,BMP1,APEX1,PSMA6,INHBA,PLOD3,CTSS,PSMD2,PAPPA,GNPDA1,HINT1,PLOD1,SNRPD3,HNRNPA3,PRCP,LOXL1,PGM3,TWF1,HNRNPM,LOC100058329,RARS1,PUF60,PSMC3,LOC100058290,LOXL2,EIF3E,NARS1,UCHL1,PGM2,NAMPT,FKBP10,RAB7A,WARS1,ADH5,U2AF2,PFKP,ACLY,HTRA1,SFPQ,STAM,SCPEP1,G3BP1,DDX17,FUS,HNRNPK,HNRNPA2B1,GLRX3,LAP3,ALDOA,GNPDA2,VCP,GNS,NCSTN,PABPC1,COL4A2,CAT,PSMD13,AARS1,PLAT,DPP3,CAPN2,PPP2R1A,PRMT1,WDR43,HEXA,PSMD6,DDX39B,ADAMTS5,RNASE4 | 0.13 | 0.33 | 0.0082 |
| GO:0065008 | Biological Process | 70 | Regulation of biological quality | KIF5B,VPS35,CD81,PLAU,HSPA9,VASP,PCSK5,SLC39A14,CPE,LGMN,SERPING1,DDB1,STC1,PAFAH1B1,G6PD,ALDH1A2,SPTBN1,CCN2,CCT7,PRDX5,GCLC,CDKL3,TCP1,GRN,GPI,ARPC5,DSTN,FBN1,FERMT2,CCT3,ALOX12,APEX1,INHBA,PLOD3,PRCP,TWF1,EMILIN1,DYNC1H1,LIPG,NIBAN2,SNX9,F11R,PABPC4,RAB7A,ADH5,CCT4,RAB11A,SCPEP1,SYNCRIP,CAPZB,ERO1A,AKR1B1,FUS,SEPTIN8,DBN1,GLRX3,CCT2,ALDOA,IFI30,NCSTN,PABPC1,ARPC2,AARS1,SRI,SPTAN1,DBNL,PLEC,FSTL1,ARPC1B,ADAMTS5 | 0.2 | 0.36 | 0.0083 |
| GO:0048856 | Biological Process | 89 | Anatomical structure development | KIF5B,CD81,HSPA9,VASP,PCSK5,SLC39A14,MMP14,ECM1,LAMB1,CPE,CSPG4,TRIM28,DDX5,CRK,COL5A2,PTK7,MANF,DDB1,PLS3,PAFAH1B1,UGDH,G6PD,ALDH1A2,CXCL8,SPTBN1,CCN2,NID1,CDKL3,GPI,ALCAM,ARCN1,FBN1,MXRA8,TGFBI,PDCD6,MMP1,ALOX12,FHL2,BMP1,INHBA,PLOD3,ENAH,PLOD1,PRCP,LAMC1,CRLF1,PGM3,LOXL2,EMILIN1,LAMA2,MYOF,NARS1,BSG,CNN2,CDC42,F11R,PABPC4,NXN,FKBP10,NDRG1,CAPZB,DDX17,CRIP2,DBN1,SGCA,AP1B1,TNC,LAMA5,MAP4,PARVA,IQGAP1,PXDN,NCSTN,ITGAV,COL4A2,MARCKS,AARS1,PDLIM5,FLNC,DBNL,CAPN2,PRMT1,PLEC,HEXA,FSTL1,FASN,ADAMTS5,RNASE4,HSPG2 | 0.17 | 0.33 | 0.0118 |
| GO:0007155 | Biological Process | 25 | Cell adhesion | LAMB1,PTK7,IGFBP7,DPT,CCN2,NID1,ALCAM,FBN1,MXRA8,FERMT2,TGFBI,LAMC1,EMILIN1,LAMA2,BSG,F11R,SGCA,SERPINB8,TNC,LAMA5,PARVA,PXDN,ITGAV,FOLR1,COL6A3 | 0.37 | 0.38 | 0.0126 |
| GO:0001704 | Biological Process | 8 | Formation of primary germ layer | MMP14,LAMB1,GPI,MMP1,INHBA,SGCA,ITGAV,COL4A2 | 0.76 | 0.44 | 0.0145 |
| GO:1904874 | Biological Process | 3 | Positive regulation of telomerase RNA localization to Cajal body | TCP1,CCT4,CCT2 | 1.68 | 0.5 | 0.0145 |
| GO:0007369 | Biological Process | 9 | Gastrulation | MMP14,LAMB1,UGDH,GPI,MMP1,INHBA,SGCA,ITGAV,COL4A2 | 0.69 | 0.41 | 0.0189 |
| GO:0044248 | Biological Process | 39 | Cellular catabolic process | ELOC,NPEPPS,PLBD2,SGTA,LGMN,DDX5,HEXB,SND1,DDB1,SMPD1,UBA1,GOT2,PRDX5,KHSRP,GSTM3,PSMA2,RBM8A,APEX1,PSMA6,PSMD2,GNPDA1,HINT1,PSMC3,EIF3E,LIPG,UCHL1,PGM2,CLTC,RAB7A,ADH5,STAM,GNPDA2,VCP,PXDN,PABPC1,CAT,PSMD13,HEXA,PSMD6 | 0.27 | 0.33 | 0.0190 |
| GO:0044283 | Biological Process | 16 | Small molecule biosynthetic process | SLC39A14,PLOD2,SEPHS1,UGDH,G6PD,ALDH1A2,GOT2,PSAT1,GPI,ENOPH1,ALOX12,PLOD3,IMPA1,LIPG,PGM2,FASN | 0.47 | 0.38 | 0.0190 |
| GO:0045454 | Biological Process | 5 | Cell redox homeostasis | PRDX5,GCLC,APEX1,ERO1A,GLRX3 | 1.04 | 0.44 | 0.0190 |
| GO:0048870 | Biological Process | 28 | Cell motility | PLAU,MMP14,LAMB1,CSPG4,CRK,PTK7,CXCL6,MMP9,PAFAH1B1,CORO1B,CXCL8,CCN2,ARPC5,ENAH,LAMC1,LOXL2,EMILIN1,NARS1,CNN2,F11R,SGCA,LAMA5,PARVA,IQGAP1,ITGAV,PLAT,FSTL1,RNASE4 | 0.33 | 0.35 | 0.0190 |
| GO:0050775 | Biological Process | 4 | Positive regulation of dendrite morphogenesis | PAFAH1B1,CDKL3,DBN1,DBNL | 1.23 | 0.45 | 0.0199 |
| GO:0090666 | Biological Process | 3 | scaRNA localization to Cajal body | TCP1,CCT4,CCT2 | 1.58 | 0.45 | 0.0211 |
| GO:0051179 | Biological Process | 90 | Localization | ERP29,KIF5B,VPS35,ARF4,CD81,NAPA,HSPA9,LMNB1,SLC39A14,GDI2,CPE,AP2B1,PLIN3,VPS26A,SGTA,VDAC1,HYOU1,SEC22B,COPG2,KPNB1,PAFAH1B1,IPO5,RRBP1,CORO1B,AP2A2,CXCL8,SPTBN1,GOT2,CCT7,SEC31A,TCP1,GRN,ARCN1,FBN1,TFG,FERMT2,CCT3,PDCD6,RBM8A,INHBA,COPA,PLOD3,SEPTIN6,TWF1,LOXL2,DYNC1H1,LIPG,NIBAN2,SNX9,CNN2,CDC42,F11R,CLTC,RANBP1,RAB7A,LMAN2,SEPTIN9,CCT4,RAB11A,SFPQ,STAM,SLC44A1,VAPA,SEC23A,ERO1A,COPB1,HNRNPA2B1,SEPTIN8,DBN1,SEPTIN11,AP1B1,VPS29,DCTN2,CCT2,GDI1,COPB2,SEPTIN7,VCP,LASP1,COPG1,PDCD6IP,ITGAV,FOLR1,SCARB2,SRI,DBNL,PITPNB,PLEC,HEXA,DDX39B | 0.15 | 0.3 | 0.0227 |
| GO:0048468 | Biological Process | 38 | Cell development | KIF5B,VASP,ECM1,LAMB1,CRK,MANF,DDB1,PAFAH1B1,UGDH,G6PD,ALDH1A2,CDKL3,ALCAM,MXRA8,PDCD6,FHL2,PLOD3,ENAH,LAMC1,LAMA2,MYOF,BSG,CDC42,F11R,PABPC4,NDRG1,TNC,LAMA5,MAP4,PARVA,IQGAP1,NCSTN,ITGAV,FLNC,DBNL,PRMT1,HEXA,FASN | 0.27 | 0.32 | 0.0229 |
| GO:0034314 | Biological Process | 4 | Arp2/3 complex-mediated actin nucleation | ACTR2,ARPC5,ARPC2,ARPC1B | 1.2 | 0.43 | 0.0231 |
| GO:0008154 | Biological Process | 6 | Actin polymerization or depolymerization | API5,VASP,DSTN,ENAH,TWF1,ARPC2 | 0.88 | 0.4 | 0.0250 |
| GO:0009226 | Biological Process | 4 | Nucleotide-sugar biosynthetic process | UGDH,GNPDA1,PGM3,GNPDA2 | 1.18 | 0.41 | 0.0277 |
| GO:0044087 | Biological Process | 26 | Regulation of cellular component biogenesis | NAPA,VASP,GDI2,TRIM28,SEC22B,SPTBN1,CCN2,ARPC5,FERMT2,TWF1,EMILIN1,DYNC1H1,SNX9,CDC42,F11R,WARS1,SEPTIN9,STAM,G3BP1,CAPZB,DPYSL3,SEPTIN8,ARPC2,SPTAN1,DBNL,ARPC1B | 0.33 | 0.32 | 0.0286 |
| GO:0022604 | Biological Process | 12 | Regulation of cell morphogenesis | CRK,PAFAH1B1,CORO1B,CDKL3,FERMT2,CDC42,F11R,CAPZB,DBN1,ALDOA,SEPTIN7,DBNL | 0.54 | 0.35 | 0.0289 |
| GO:0034641 | Biological Process | 76 | Cellular nitrogen compound metabolic process | NUDT21,NPEPPS,PCSK5,HNRNPA1,CPE,YARS1,RPL12,TRIM28,RPS4X,DDX5,HEXB,THOP1,SND1,RTRAF,HNRNPL,DDB1,SMPD1,RPL10A,UGDH,ATIC,G6PD,RPLP0,TSN,OLA1,CCN2,GCLC,EIF2S1,KHSRP,GPI,GSTM3,PTBP1,GARS1,PAICS,SARS1,EEF1A1,RPL5,TARS1,RBM8A,APEX1,INHBA,GNPDA1,HINT1,SNRPD3,HNRNPA3,PGM3,HNRNPM,LOC100058329,RARS1,PUF60,LOC100058290,EIF3E,NARS1,PGM2,NAMPT,WARS1,U2AF2,PFKP,ACLY,SFPQ,G3BP1,DDX17,FUS,HNRNPK,HNRNPA2B1,ALDOA,GNPDA2,VCP,NCSTN,PABPC1,COL4A2,AARS1,PRMT1,WDR43,HEXA,DDX39B,RNASE4 | 0.17 | 0.29 | 0.0291 |
| GO:0001666 | Biological Process | 9 | Response to hypoxia | PLAU,NPEPPS,PLOD2,HYOU1,PLOD1,LOXL2,NDRG1,PLAT,RNASE4 | 0.64 | 0.36 | 0.0309 |
| GO:1901607 | Biological Process | 6 | Alpha-amino acid biosynthetic process | PLOD2,SEPHS1,GOT2,PSAT1,ENOPH1,PLOD3 | 0.85 | 0.38 | 0.0311 |
| GO:0000380 | Biological Process | 4 | Alternative mRNA splicing, via spliceosome | DDX5,PUF60,SFPQ,DDX17 | 1.15 | 0.4 | 0.0314 |
| GO:0010638 | Biological Process | 17 | Positive regulation of organelle organization | VPS35,VASP,TRIM28,CCN2,CCT7,TCP1,GRN,DSTN,FERMT2,CCT3,DYNC1H1,SNX9,SEPTIN9,CCT4,SFPQ,G3BP1,CCT2 | 0.43 | 0.33 | 0.0320 |
| GO:0043043 | Biological Process | 18 | Peptide biosynthetic process | PCSK5,YARS1,RPL12,RPS4X,RPL10A,RPLP0,GCLC,EIF2S1,GARS1,SARS1,EEF1A1,RPL5,TARS1,RARS1,EIF3E,NARS1,WARS1,AARS1 | 0.41 | 0.32 | 0.0327 |
| GO:0006397 | Biological Process | 16 | mRNA processing | NUDT21,HNRNPA1,DDX5,HNRNPL,PTBP1,RBM8A,SNRPD3,HNRNPA3,HNRNPM,PUF60,U2AF2,SFPQ,DDX17,HNRNPA2B1,PABPC1,DDX39B | 0.44 | 0.33 | 0.0340 |
| GO:0032502 | Biological Process | 93 | Developmental process | KIF5B,CD81,HSPA9,VASP,PCSK5,SLC39A14,MMP14,ECM1,LAMB1,CPE,CSPG4,TRIM28,DDX5,CRK,COL5A2,PTK7,MANF,DDB1,MMP9,PLS3,PAFAH1B1,UGDH,G6PD,ALDH1A2,CXCL8,SPTBN1,CCN2,NID1,CDKL3,GPI,ALCAM,ARCN1,FBN1,MXRA8,TGFBI,PDCD6,MMP1,ALOX12,FHL2,BMP1,INHBA,PLOD3,ENAH,PLOD1,PRCP,LAMC1,SEPTIN6,CRLF1,PGM3,LOXL2,EMILIN1,LAMA2,MYOF,NARS1,BSG,CNN2,CDC42,F11R,PABPC4,NXN,FKBP10,NDRG1,CAPZB,DDX17,CRIP2,DBN1,SGCA,AP1B1,TNC,SEPTIN7,LAMA5,MAP4,PARVA,IQGAP1,PXDN,NCSTN,ITGAV,COL4A2,MARCKS,AARS1,PDLIM5,FLNC,DBNL,CAPN2,PRMT1,PLEC,HEXA,FSTL1,FASN,FLNB,ADAMTS5,RNASE4,HSPG2 | 0.14 | 0.27 | 0.0340 |
| GO:0022607 | Biological Process | 51 | Cellular component assembly | NUDT21,API5,HSPA9,VASP,LMNB1,LAMB1,CSPG4,PLS3,PSMD5,KPNB1,PAFAH1B1,UGDH,ALDH1A2,CORO1B,AP2A2,RPLP0,QSOX1,EIF2S1,TFG,FERMT2,CCT3,PDCD6,RPL5,PLOD3,SNRPD3,LAMC1,SEPTIN6,PUF60,EMILIN1,DYNC1H1,EIF3E,SNX9,CDC42,CLTC,FKBP10,RAB7A,G3BP1,DPYSL3,FUS,GLRX3,CCT2,SEPTIN7,PARVA,PXDN,ARPC2,MARCKS,FLNC,DBNL,PPP2R1A,PRMT1,DDX39B | 0.21 | 0.29 | 0.0347 |
| GO:0016070 | Biological Process | 37 | RNA metabolic process | NUDT21,HNRNPA1,YARS1,DDX5,SND1,RTRAF,HNRNPL,RPL10A,TSN,KHSRP,PTBP1,GARS1,SARS1,TARS1,RBM8A,INHBA,SNRPD3,HNRNPA3,HNRNPM,RARS1,PUF60,EIF3E,NARS1,WARS1,U2AF2,SFPQ,DDX17,FUS,HNRNPK,HNRNPA2B1,PABPC1,COL4A2,AARS1,PRMT1,WDR43,DDX39B,RNASE4 | 0.26 | 0.29 | 0.0350 |
| GO:0009888 | Biological Process | 37 | Tissue development | VASP,SLC39A14,MMP14,ECM1,LAMB1,TRIM28,DDX5,PTK7,PAFAH1B1,ALDH1A2,CCN2,GPI,PDCD6,MMP1,FHL2,INHBA,PLOD3,PLOD1,LAMC1,CRLF1,LOXL2,LAMA2,CDC42,F11R,DDX17,SGCA,TNC,LAMA5,IQGAP1,ITGAV,COL4A2,MARCKS,PRMT1,PLEC,FSTL1,FASN,HSPG2 | 0.26 | 0.29 | 0.0360 |
| GO:0009057 | Biological Process | 27 | Macromolecule catabolic process | ELOC,ADAMTS7,SGTA,LGMN,DDX5,SND1,DDB1,UBA1,KHSRP,PSMA2,PSMB1,RBM8A,APEX1,PSMA6,CTSS,PSMD2,PSMC3,EIF3E,UCHL1,PGM2,RAB7A,STAM,VCP,PABPC1,PSMD13,CAPN2,PSMD6 | 0.31 | 0.3 | 0.0369 |
| GO:0050684 | Biological Process | 8 | Regulation of mRNA processing | NUDT21,DDX5,PTBP1,RBM8A,PUF60,U2AF2,DDX17,HNRNPK | 0.67 | 0.34 | 0.0381 |
| GO:0051246 | Biological Process | 53 | Regulation of protein metabolic process | VPS35,CD81,ECM1,SGTA,LGMN,CSPG4,RPS4X,SERPING1,SERPINB9,RTRAF,DDB1,SMPD1,IGFBP5,SEC22B,G6PD,PRDX5,ILF3,EIF2S1,PSME2,GRN,SERPINE1,FBN1,EEF1A1,FERMT2,PDCD6,PCOLCE,RBM8A,INHBA,PSMD2,CRLF1,TWF1,RBM3,PSMC3,MVP,EMILIN1,EIF3E,LIPG,SDCBP,SNX9,NXN,WARS1,U2AF2,SYNCRIP,SERPINB8,IQGAP1,NCSTN,PABPC1,ITGAV,SERPINB1,AARS1,PSME1,PLAT,RNASE4 | 0.21 | 0.28 | 0.0381 |
| GO:0071702 | Biological Process | 44 | Organic substance transport | ERP29,VPS35,ARF4,NAPA,HSPA9,GDI2,CPE,AP2B1,VPS26A,SGTA,COPG2,KPNB1,PAFAH1B1,IPO5,RRBP1,AP2A2,GOT2,SEC31A,TCP1,ARCN1,PDCD6,RBM8A,INHBA,COPA,LIPG,SNX9,CLTC,RAB7A,RAB11A,STAM,SLC44A1,SEC23A,COPB1,HNRNPA2B1,AP1B1,VPS29,GDI1,COPB2,VCP,COPG1,FOLR1,SCARB2,PITPNB,DDX39B | 0.23 | 0.28 | 0.0381 |
| GO:0009067 | Biological Process | 4 | Aspartate family amino acid biosynthetic process | PLOD2,GOT2,ENOPH1,PLOD3 | 1.11 | 0.37 | 0.0388 |
| GO:0043604 | Biological Process | 20 | Amide biosynthetic process | PCSK5,YARS1,RPL12,RPS4X,SMPD1,RPL10A,RPLP0,GCLC,EIF2S1,GARS1,SARS1,EEF1A1,RPL5,TARS1,RARS1,EIF3E,NARS1,WARS1,ACLY,AARS1 | 0.37 | 0.31 | 0.0388 |
| GO:0048523 | Biological Process | 82 | Negative regulation of cellular process | HTRA3,VPS35,PA2G4,HSPA9,ADAMTS7,MMP14,ECM1,GDI2,SGTA,LGMN,TRIM28,HDGF,PPP2CB,DDX5,CRK,COL5A2,VDAC1,RTRAF,DDB1,SMPD1,IGFBP5,HYOU1,SEC22B,PAFAH1B1,ALDH1A2,CORO1B,CXCL8,DPT,SPTBN1,CCN2,VAT1,QSOX1,PRDX5,GCLC,ILF3,CDKL3,EIF2S1,HNRNPAB,GRN,PTBP1,FBN1,SARS1,FERMT2,PDCD6,ALOX12,FHL2,INHBA,CRLF1,TWF1,LOXL2,MVP,EMILIN1,NIBAN2,CNN2,F11R,CLTC,NXN,RAB7A,WARS1,U2AF2,NDRG1,SFPQ,SYNCRIP,G3BP1,CAPZB,DPYSL3,ERO1A,FUS,SGCA,GDI1,IQGAP1,IFI30,PABPC1,ITGAV,CAT,AARS1,SRI,SPTAN1,PRMT1,FSTL1,DDX39B,RNASE4 | 0.15 | 0.27 | 0.0388 |
| GO:0090114 | Biological Process | 4 | COPII-coated vesicle budding | SEC31A,TFG,PDCD6,SEC23A | 1.11 | 0.37 | 0.0388 |
| GO:0031589 | Biological Process | 9 | Cell-substrate adhesion | LAMB1,CCN2,NID1,FERMT2,LAMC1,EMILIN1,SGCA,PARVA,ITGAV | 0.62 | 0.34 | 0.0390 |
| GO:0046394 | Biological Process | 11 | Carboxylic acid biosynthetic process | PLOD2,SEPHS1,UGDH,ALDH1A2,GOT2,PSAT1,ENOPH1,ALOX12,PLOD3,LIPG,FASN | 0.54 | 0.33 | 0.0390 |
| GO:0061640 | Biological Process | 7 | Cytoskeleton-dependent cytokinesis | SEPTIN6,SNX9,SEPTIN9,SEPTIN8,SEPTIN11,SEPTIN7,PDCD6IP | 0.73 | 0.35 | 0.0390 |
| GO:1901566 | Biological Process | 35 | Organonitrogen compound biosynthetic process | PCSK5,PLOD2,YARS1,RPL12,RPS4X,SMPD1,RPL10A,SEPHS1,UGDH,ATIC,RPLP0,GOT2,GCLC,PSAT1,EIF2S1,ENOPH1,GARS1,PAICS,SARS1,EEF1A1,RPL5,TARS1,INHBA,PLOD3,RARS1,EIF3E,NARS1,PGM2,NAMPT,WARS1,ACLY,ALDOA,NCSTN,AARS1,HEXA | 0.26 | 0.29 | 0.0393 |
| GO:0006048 | Biological Process | 3 | UDP-N-acetylglucosamine biosynthetic process | GNPDA1,PGM3,GNPDA2 | 1.38 | 0.37 | 0.0426 |
| GO:0048024 | Biological Process | 7 | Regulation of mRNA splicing, via spliceosome | DDX5,PTBP1,RBM8A,PUF60,U2AF2,DDX17,HNRNPK | 0.72 | 0.34 | 0.0432 |
| GO:1901576 | Biological Process | 53 | Organic substance biosynthetic process | PCSK5,SLC39A14,PLOD2,YARS1,RPL12,RPS4X,DDX5,SMPD1,RPL10A,SEPHS1,UGDH,ATIC,G6PD,ALDH1A2,RPLP0,CCN2,GOT2,GCLC,PSAT1,EIF2S1,GPI,ENOPH1,GARS1,PAICS,SARS1,EEF1A1,RPL5,TARS1,ALOX12,INHBA,PLOD3,IMPA1,GNPDA1,PGM3,RARS1,EIF3E,LIPG,NARS1,PGM2,NAMPT,WARS1,ACLY,NANS,PI4KA,ALDOA,GNPDA2,VCP,NCSTN,COL4A2,AARS1,HEXA,FASN,RNASE4 | 0.2 | 0.27 | 0.0436 |
| GO:0030154 | Biological Process | 63 | Cell differentiation | KIF5B,CD81,HSPA9,VASP,SLC39A14,MMP14,ECM1,LAMB1,CSPG4,TRIM28,DDX5,CRK,PTK7,MANF,DDB1,PAFAH1B1,UGDH,G6PD,ALDH1A2,CCN2,CDKL3,ALCAM,MXRA8,PDCD6,MMP1,FHL2,INHBA,PLOD3,ENAH,LAMC1,SEPTIN6,LOXL2,LAMA2,MYOF,BSG,CDC42,F11R,PABPC4,NXN,NDRG1,DDX17,DBN1,SGCA,TNC,SEPTIN7,LAMA5,MAP4,PARVA,IQGAP1,NCSTN,ITGAV,COL4A2,MARCKS,FLNC,DBNL,CAPN2,PRMT1,PLEC,HEXA,FSTL1,FASN,FLNB,ADAMTS5 | 0.18 | 0.27 | 0.0437 |
| GO:0072359 | Biological Process | 22 | Circulatory system development | PCSK5,CPE,PTK7,ALDH1A2,CCN2,FBN1,TGFBI,FHL2,PRCP,LOXL2,EMILIN1,NXN,FKBP10,AP1B1,PARVA,PXDN,ITGAV,PDLIM5,PRMT1,PLEC,RNASE4,HSPG2 | 0.35 | 0.29 | 0.0437 |
| GO:0016071 | Biological Process | 19 | mRNA metabolic process | NUDT21,HNRNPA1,DDX5,SND1,HNRNPL,KHSRP,PTBP1,RBM8A,SNRPD3,HNRNPA3,HNRNPM,PUF60,EIF3E,U2AF2,SFPQ,DDX17,HNRNPA2B1,PABPC1,DDX39B | 0.38 | 0.3 | 0.0438 |
| GO:0006890 | Biological Process | 5 | Retrograde vesicle-mediated transport, Golgi to endoplasmic reticulum | SEC22B,ARCN1,COPA,LMAN2,COPB2 | 0.9 | 0.34 | 0.0461 |
| GO:0005488 | Molecular Function | 277 | Binding | ERP29,FKBP9,HTRA3,NUDT21,HNRNPF,KIF5B,VPS35,ARF4,CKAP4,CD81,PA2G4,HNRNPH3,NAPA,HSPA9,NPEPPS,ADAMTS7,VASP,PCSK5,LMNB1,MMP14,ECM1,ACTR2,HNRNPA1,FBLN2,LAMB1,CPE,AP2B1,PLIN3,VPS26A,PLOD2,YARS1,SGTA,PPP1CA,CSPG4,RPL12,TRIM28,RPS4X,HDGF,PPP2CB,DDX5,CRK,COL5A2,PTK7,CCT8,MANF,SOD2,THOP1,SND1,VDAC1,RTRAF,CXCL6,HNRNPL,DDB1,SMPD1,IGFBP7,IGFBP5,MMP9,PLS3,HYOU1,PSMD5,STC1,RPL10A,SEPHS1,KPNB1,PAFAH1B1,UBA1,IPO5,HNRNPH1,UGDH,ATIC,GANAB,G6PD,RRBP1,CORO1B,AP2A2,CXCL8,EDIL3,RPLP0,TSN,SPTBN1,ANGPTL2,OLA1,PPIA,CCN2,NID1,VAT1,GOT2,CCT7,QSOX1,PPP1CC,PRDX5,GCLC,ILF3,LRRC59,CDKL3,PSAT1,EIF2S1,HNRNPAB,TCP1,PSME2,GRN,KHSRP,GPI,HSPH1,ALCAM,ARPC5,AREL1,GSTM3,CCT5,PTBP1,ENOPH1,GARS1,SERPINE1,PAICS,ARCN1,DSTN,FBN1,SARS1,CNN3,TFG,EEF1A1,PSMA2,FERMT2,TGFBI,CCT3,PDCD6,RPL5,TARS1,MMP1,HSPA4,PSMB1,PCOLCE,DNPEP,RBM8A,ALOX12,FHL2,BMP1,APEX1,PSMA6,INHBA,COPA,PLOD3,ENAH,CTSS,PCBP2,IMPA1,GNPDA1,HINT1,PLOD1,SNRPD3,MDH2,HNRNPA3,LOXL1,SEPTIN6,CRLF1,PGM3,TWF1,HNRNPM,RBM3,RARS1,PUF60,PSMC3,LOC100058290,LOXL2,MVP,EMILIN1,DYNC1H1,LAMA2,EIF3E,LIPG,MYOF,SDCBP,DYNLL2,NARS1,NUCB1,PGM2,SNX9,BSG,CNN2,CDC42,F11R,CLTC,NAMPT,PABPC4,FKBP10,RAB7A,WARS1,ADH5,LMAN2,U2AF2,SEPTIN9,DPYSL2,NDRG1,CCT4,PFKP,ACLY,RAB11A,HTRA1,SFPQ,STAM,SYNCRIP,G3BP1,VAPA,CAPZB,DDX17,CRIP2,SEC23A,DPYSL3,ERO1A,PI4KA,FUS,HNRNPK,HNRNPA2B1,SEPTIN8,DBN1,AHNAK,SEPTIN11,SGCA,AP1B1,VPS29,DCTN2,GLRX3,ILF2,CCT2,LAP3,NONO,ALDOA,COPB2,TNC,GNPDA2,SEPTIN7,LAMA5,VCP,LASP1,PDCD6IP,MAP4,PARVA,CALD1,IQGAP1,PXDN,HNRNPD,GNS,NCSTN,PABPC1,ITGAV,ARPC2,HNRNPR,FUBP1,FOLR1,CAT,MARCKS,AARS1,PDLIM5,PLAT,SCARB2,SRI,SPTAN1,CCT6B,FLNC,DBNL,DPP3,PITPNB,CAPN2,EWSR1,PRMT1,PLEC,ENSECAP00000050120,WDR43,HEXA,FSTL1,ARPC1B,FASN,FLNB,DDX39B,ADAMTS5,RNASE4,HSPG2 | 0.12 | 0.63 | 1.82e-12 |
| GO:0003723 | Molecular Function | 55 | RNA binding | NUDT21,HNRNPF,CKAP4,HNRNPH3,HNRNPA1,YARS1,RPL12,RPS4X,DDX5,SND1,RTRAF,HNRNPL,RPL10A,HNRNPH1,RRBP1,RPLP0,TSN,ILF3,LRRC59,EIF2S1,HNRNPAB,KHSRP,PTBP1,ARCN1,SARS1,EEF1A1,RPL5,RBM8A,APEX1,PSMA6,PCBP2,SNRPD3,HNRNPA3,HNRNPM,RBM3,PUF60,CLTC,PABPC4,U2AF2,SFPQ,SYNCRIP,G3BP1,DDX17,FUS,HNRNPK,HNRNPA2B1,ILF2,NONO,HNRNPD,PABPC1,HNRNPR,FUBP1,AARS1,EWSR1,DDX39B | 0.47 | 1.01 | 2.42e-09 |
| GO:0005515 | Molecular Function | 166 | Protein binding | ERP29,HTRA3,NUDT21,KIF5B,VPS35,CD81,PA2G4,NAPA,HSPA9,VASP,LMNB1,ECM1,ACTR2,LAMB1,CPE,AP2B1,PLIN3,VPS26A,SGTA,CSPG4,TRIM28,HDGF,DDX5,CRK,COL5A2,PTK7,CCT8,MANF,RTRAF,CXCL6,DDB1,IGFBP7,IGFBP5,PLS3,PSMD5,STC1,SEPHS1,KPNB1,PAFAH1B1,IPO5,UGDH,ATIC,G6PD,CORO1B,AP2A2,CXCL8,TSN,SPTBN1,ANGPTL2,CCN2,NID1,CCT7,LRRC59,PSAT1,TCP1,PSME2,GRN,GPI,HSPH1,ALCAM,ARPC5,AREL1,GSTM3,CCT5,GARS1,SERPINE1,DSTN,FBN1,SARS1,CNN3,TFG,EEF1A1,PSMA2,FERMT2,TGFBI,CCT3,PDCD6,TARS1,PSMB1,DNPEP,FHL2,BMP1,PSMA6,INHBA,COPA,ENAH,CTSS,GNPDA1,MDH2,CRLF1,TWF1,PUF60,PSMC3,LOC100058290,MVP,EMILIN1,DYNC1H1,LAMA2,EIF3E,SDCBP,DYNLL2,NARS1,NUCB1,SNX9,CNN2,F11R,CLTC,NAMPT,WARS1,ADH5,LMAN2,U2AF2,DPYSL2,NDRG1,CCT4,PFKP,HTRA1,SFPQ,STAM,VAPA,CAPZB,DPYSL3,FUS,DBN1,AHNAK,SGCA,AP1B1,DCTN2,GLRX3,CCT2,ALDOA,COPB2,TNC,GNPDA2,LAMA5,VCP,LASP1,PDCD6IP,MAP4,PARVA,CALD1,IQGAP1,PXDN,NCSTN,ITGAV,ARPC2,CAT,MARCKS,PDLIM5,PLAT,SCARB2,SRI,SPTAN1,CCT6B,FLNC,DBNL,EWSR1,PRMT1,PLEC,ENSECAP00000050120,HEXA,ARPC1B,FLNB,DDX39B,RNASE4,HSPG2 | 0.2 | 0.67 | 3.91e-09 |
| GO:0140662 | Molecular Function | 12 | ATP-dependent protein folding chaperone | HSPA9,CCT8,HYOU1,CCT7,TCP1,HSPH1,CCT5,CCT3,HSPA4,CCT4,CCT2,CCT6B | 1.33 | 1.95 | 4.79e-09 |
| GO:0016787 | Molecular Function | 79 | Hydrolase activity | HTRA3,KIF5B,ARF4,PLAU,HSPA9,NPEPPS,ADAMTS7,PCSK5,MMP14,PLBD2,CPE,PPP1CA,LGMN,PPP2CB,DDX5,CCT8,HEXB,THOP1,SND1,SMPD1,MMP9,ATIC,GANAB,TSN,OLA1,CCT7,PPP1CC,TCP1,ENOPH1,GARS1,EEF1A1,CCT3,MMP1,PSMB1,DNPEP,BMP1,APEX1,CTSS,PAPPA,IMPA1,GNPDA1,HINT1,PRCP,SEPTIN6,PSMC3,LIPG,UCHL1,CDC42,RAB7A,SEPTIN9,DPYSL2,CCT4,RAB11A,HTRA1,SCPEP1,G3BP1,DDX17,DPYSL3,SEPTIN8,SEPTIN11,CCT2,LAP3,MINPP1,GNPDA2,SEPTIN7,VCP,GNS,NCSTN,CAT,AARS1,PLAT,CCT6B,DPP3,CAPN2,HEXA,FASN,DDX39B,ADAMTS5,RNASE4 | 0.33 | 0.79 | 3.76e-08 |
| GO:0042802 | Molecular Function | 63 | Identical protein binding | ERP29,HTRA3,NUDT21,KIF5B,SGTA,RTRAF,CXCL6,SEPHS1,PAFAH1B1,UGDH,ATIC,G6PD,CORO1B,TSN,PSAT1,PSME2,ALCAM,GSTM3,GARS1,FBN1,SARS1,TFG,PSMA2,TGFBI,PDCD6,TARS1,DNPEP,FHL2,BMP1,INHBA,GNPDA1,MDH2,PUF60,PSMC3,LOC100058290,MVP,EMILIN1,SNX9,F11R,CLTC,NAMPT,WARS1,ADH5,PFKP,HTRA1,SFPQ,DPYSL3,FUS,AHNAK,DCTN2,GLRX3,ALDOA,GNPDA2,PDCD6IP,CAT,MARCKS,SCARB2,EWSR1,PRMT1,ENSECAP00000050120,FLNB,DDX39B,RNASE4 | 0.38 | 0.84 | 4.95e-08 |
| GO:0003779 | Molecular Function | 27 | Actin binding | VASP,ACTR2,PLS3,CORO1B,SPTBN1,ARPC5,DSTN,CNN3,FERMT2,ENAH,TWF1,CNN2,CAPZB,DBN1,LASP1,PARVA,CALD1,IQGAP1,ARPC2,MARCKS,PDLIM5,SPTAN1,FLNC,DBNL,ARPC1B,FLNB,RNASE4 | 0.61 | 0.98 | 9.81e-07 |
| GO:0036094 | Molecular Function | 72 | Small molecule binding | KIF5B,ARF4,CD81,HSPA9,ACTR2,PLOD2,YARS1,HDGF,DDX5,PTK7,CCT8,VDAC1,HYOU1,SEPHS1,UBA1,UGDH,G6PD,OLA1,GOT2,CCT7,QSOX1,GCLC,CDKL3,PSAT1,TCP1,GPI,HSPH1,CCT5,GARS1,PAICS,SARS1,EEF1A1,CCT3,TARS1,HSPA4,PLOD3,HINT1,PLOD1,SEPTIN6,TWF1,RARS1,PSMC3,DYNC1H1,NARS1,BSG,CDC42,RAB7A,WARS1,LMAN2,SEPTIN9,CCT4,PFKP,ACLY,RAB11A,G3BP1,DDX17,ERO1A,PI4KA,SEPTIN8,SEPTIN11,CCT2,ALDOA,SEPTIN7,VCP,FOLR1,CAT,AARS1,SCARB2,CCT6B,PRMT1,FASN,DDX39B | 0.29 | 0.63 | 9.97e-06 |
| GO:0097367 | Molecular Function | 68 | Carbohydrate derivative binding | KIF5B,ARF4,HSPA9,ACTR2,YARS1,HDGF,DDX5,PTK7,CCT8,MANF,CXCL6,HYOU1,SEPHS1,UBA1,CXCL8,OLA1,CCN2,NID1,CCT7,GCLC,CDKL3,TCP1,GPI,HSPH1,AREL1,CCT5,GARS1,PAICS,FBN1,SARS1,EEF1A1,CCT3,TARS1,HSPA4,PCOLCE,CTSS,SEPTIN6,TWF1,RARS1,PSMC3,DYNC1H1,LIPG,SDCBP,NARS1,CDC42,RAB7A,WARS1,SEPTIN9,CCT4,PFKP,ACLY,RAB11A,G3BP1,DDX17,DPYSL3,PI4KA,SEPTIN8,SEPTIN11,CCT2,TNC,SEPTIN7,VCP,AARS1,CCT6B,FSTL1,DDX39B,ADAMTS5,RNASE4 | 0.3 | 0.64 | 9.97e-06 |
| GO:0043167 | Molecular Function | 126 | Ion binding | FKBP9,KIF5B,ARF4,HSPA9,NPEPPS,ADAMTS7,MMP14,ACTR2,FBLN2,CPE,PLOD2,YARS1,PPP1CA,TRIM28,PPP2CB,DDX5,COL5A2,PTK7,CCT8,SOD2,THOP1,SMPD1,MMP9,PLS3,HYOU1,SEPHS1,UBA1,EDIL3,OLA1,NID1,VAT1,GOT2,CCT7,QSOX1,PPP1CC,GCLC,CDKL3,PSAT1,TCP1,HSPH1,AREL1,GSTM3,CCT5,ENOPH1,GARS1,PAICS,FBN1,SARS1,EEF1A1,FERMT2,CCT3,PDCD6,TARS1,MMP1,HSPA4,DNPEP,ALOX12,FHL2,BMP1,APEX1,PLOD3,IMPA1,PLOD1,LOXL1,SEPTIN6,PGM3,TWF1,RARS1,PSMC3,LOXL2,DYNC1H1,LIPG,MYOF,NARS1,NUCB1,PGM2,CDC42,FKBP10,RAB7A,WARS1,ADH5,SEPTIN9,CCT4,PFKP,ACLY,RAB11A,G3BP1,DDX17,CRIP2,SEC23A,ERO1A,PI4KA,FUS,SEPTIN8,DBN1,SEPTIN11,SGCA,VPS29,GLRX3,CCT2,LAP3,SEPTIN7,VCP,LASP1,CALD1,IQGAP1,GNS,ITGAV,FOLR1,CAT,AARS1,PDLIM5,SCARB2,SRI,SPTAN1,CCT6B,DPP3,PITPNB,CAPN2,EWSR1,PRMT1,FSTL1,DDX39B,ADAMTS5,RNASE4,HSPG2 | 0.19 | 0.54 | 1.70e-05 |
| GO:0005201 | Molecular Function | 11 | Extracellular matrix structural constituent | LAMB1,COL5A2,DPT,MXRA5,FBN1,LAMC1,EMILIN1,LAMA2,LAMA5,PXDN,COL4A2 | 0.94 | 0.98 | 4.45e-05 |
| GO:0003824 | Molecular Function | 138 | Catalytic activity | FKBP9,HTRA3,KIF5B,PTGR1,ARF4,PLAU,HSPA9,NPEPPS,ADAMTS7,PCSK5,MMP14,PLBD2,CPE,PLOD2,YARS1,PPP1CA,LGMN,TRIM28,PPP2CB,DDX5,PTK7,CCT8,SOD2,HEXB,THOP1,SND1,SMPD1,MMP9,SEPHS1,UBA1,UGDH,ATIC,GANAB,G6PD,ALDH1A2,TSN,OLA1,PPIA,VAT1,GOT2,CCT7,ERP44,QSOX1,PPP1CC,PRDX5,GCLC,CDKL3,PSAT1,FKBP3,TCP1,GPI,GSTM3,ENOPH1,GARS1,PAICS,SARS1,EEF1A1,CCT3,TARS1,MMP1,PSMB1,DNPEP,ALOX12,BMP1,APEX1,PLOD3,CTSS,PAPPA,IMPA1,GNPDA1,HINT1,PLOD1,MDH2,PRCP,LOXL1,SEPTIN6,PGM3,TWF1,LOC100058329,RARS1,PSMC3,LOC100058290,LOXL2,LIPG,NARS1,UCHL1,PGM2,CDC42,NAMPT,NXN,FKBP10,RAB7A,WARS1,ADH5,SEPTIN9,DPYSL2,CCT4,PFKP,ACLY,RAB11A,HTRA1,SCPEP1,G3BP1,DDX17,DPYSL3,ERO1A,AKR1B1,NANS,PI4KA,TXNL1,SEPTIN8,SEPTIN11,GLRX3,CCT2,LAP3,ALDOA,MINPP1,GNPDA2,SEPTIN7,VCP,PXDN,IFI30,GNS,NCSTN,CAT,AARS1,PLAT,TXNDC17,TALDO1,CCT6B,DPP3,CAPN2,PRMT1,HEXA,FASN,DDX39B,ADAMTS5,RNASE4 | 0.16 | 0.5 | 5.50e-05 |
| GO:0044877 | Molecular Function | 40 | Protein-containing complex binding | KIF5B,CD81,LAMB1,SGTA,DDX5,SND1,DDB1,PLS3,PAFAH1B1,CORO1B,SPTBN1,CCN2,NID1,EIF2S1,ARPC5,DSTN,FBN1,FERMT2,TGFBI,PCOLCE,CTSS,TWF1,EMILIN1,SNX9,F11R,PFKP,CAPZB,SGCA,DCTN2,LAMA5,LASP1,IQGAP1,ITGAV,ARPC2,MARCKS,SPTAN1,FLNC,DBNL,ARPC1B,FLNB | 0.39 | 0.64 | 7.58e-05 |
| GO:0004812 | Molecular Function | 8 | aminoacyl-tRNA ligase activity | YARS1,GARS1,SARS1,TARS1,RARS1,NARS1,WARS1,AARS1 | 1.07 | 0.89 | 0.00021 |
| GO:0048029 | Molecular Function | 9 | Monosaccharide binding | PLOD2,G6PD,GPI,PLOD3,PLOD1,BSG,LMAN2,PFKP,ALDOA | 0.95 | 0.82 | 0.00032 |
| GO:0008092 | Molecular Function | 35 | Cytoskeletal protein binding | KIF5B,VASP,ACTR2,PLS3,PAFAH1B1,CORO1B,SPTBN1,HSPH1,ARPC5,DSTN,CNN3,FERMT2,ENAH,TWF1,CNN2,NDRG1,CAPZB,DPYSL3,DBN1,DCTN2,ALDOA,LASP1,MAP4,PARVA,CALD1,IQGAP1,ARPC2,MARCKS,PDLIM5,SPTAN1,FLNC,DBNL,ARPC1B,FLNB,RNASE4 | 0.38 | 0.58 | 0.00035 |
| GO:0051015 | Molecular Function | 15 | Actin filament binding | PLS3,CORO1B,ARPC5,DSTN,TWF1,CAPZB,LASP1,IQGAP1,ARPC2,MARCKS,SPTAN1,FLNC,DBNL,ARPC1B,FLNB | 0.65 | 0.69 | 0.00045 |
| GO:0005198 | Molecular Function | 30 | Structural molecule activity | LMNB1,LAMB1,RPL12,RPS4X,COL5A2,RPL10A,COPG2,RPLP0,DPT,SPTBN1,MXRA5,SEC31A,ARPC5,FBN1,RPL5,LAMC1,EMILIN1,LAMA2,CLTC,COPB1,AHNAK,COPB2,LAMA5,COPG1,PXDN,ARPC2,COL4A2,PSMD13,PLEC,ENSECAP00000050120 | 0.41 | 0.56 | 0.00068 |
| GO:0005178 | Molecular Function | 11 | Integrin binding | CD81,LAMB1,CCN2,FBN1,FERMT2,TGFBI,EMILIN1,F11R,SGCA,LAMA5,ITGAV | 0.78 | 0.7 | 0.00070 |
| GO:0000166 | Molecular Function | 59 | Nucleotide binding | KIF5B,ARF4,HSPA9,ACTR2,YARS1,HDGF,DDX5,PTK7,CCT8,VDAC1,HYOU1,SEPHS1,UBA1,UGDH,G6PD,OLA1,CCT7,QSOX1,GCLC,CDKL3,TCP1,HSPH1,CCT5,GARS1,PAICS,SARS1,EEF1A1,CCT3,TARS1,HSPA4,HINT1,SEPTIN6,TWF1,RARS1,PSMC3,DYNC1H1,NARS1,CDC42,RAB7A,WARS1,SEPTIN9,CCT4,PFKP,ACLY,RAB11A,G3BP1,DDX17,ERO1A,PI4KA,SEPTIN8,SEPTIN11,CCT2,SEPTIN7,VCP,CAT,AARS1,CCT6B,PRMT1,DDX39B | 0.26 | 0.48 | 0.00087 |
| GO:0016874 | Molecular Function | 12 | Ligase activity | YARS1,UBA1,GCLC,GARS1,PAICS,SARS1,TARS1,RARS1,NARS1,UCHL1,WARS1,AARS1 | 0.69 | 0.62 | 0.0014 |
| GO:0043168 | Molecular Function | 62 | Anion binding | KIF5B,ARF4,HSPA9,ACTR2,PLOD2,YARS1,DDX5,PTK7,CCT8,HYOU1,SEPHS1,UBA1,OLA1,GOT2,CCT7,QSOX1,GCLC,CDKL3,PSAT1,TCP1,HSPH1,GSTM3,CCT5,GARS1,PAICS,SARS1,EEF1A1,FERMT2,CCT3,TARS1,HSPA4,PLOD3,PLOD1,SEPTIN6,TWF1,RARS1,PSMC3,DYNC1H1,NARS1,CDC42,RAB7A,WARS1,ADH5,SEPTIN9,CCT4,PFKP,ACLY,RAB11A,G3BP1,DDX17,ERO1A,PI4KA,SEPTIN8,SEPTIN11,CCT2,SEPTIN7,VCP,IQGAP1,FOLR1,AARS1,CCT6B,DDX39B | 0.24 | 0.45 | 0.0014 |
| GO:0017111 | Molecular Function | 24 | Nucleoside-triphosphatase activity | KIF5B,ARF4,HSPA9,CCT8,OLA1,CCT7,TCP1,EEF1A1,CCT3,SEPTIN6,PSMC3,CDC42,RAB7A,SEPTIN9,CCT4,RAB11A,G3BP1,SEPTIN8,SEPTIN11,CCT2,SEPTIN7,VCP,CCT6B,DDX39B | 0.44 | 0.53 | 0.0015 |
| GO:0016853 | Molecular Function | 12 | Isomerase activity | FKBP9,PPIA,ERP44,QSOX1,FKBP3,GPI,ENOPH1,GNPDA1,PGM3,PGM2,FKBP10,GNPDA2 | 0.68 | 0.6 | 0.0017 |
| GO:0097159 | Molecular Function | 124 | Organic cyclic compound binding | NUDT21,HNRNPF,KIF5B,ARF4,CKAP4,CD81,PA2G4,HNRNPH3,HSPA9,LMNB1,ACTR2,HNRNPA1,PLOD2,YARS1,RPL12,TRIM28,RPS4X,HDGF,DDX5,PTK7,CCT8,SND1,VDAC1,RTRAF,HNRNPL,DDB1,HYOU1,RPL10A,SEPHS1,UBA1,HNRNPH1,UGDH,G6PD,RRBP1,RPLP0,TSN,OLA1,GOT2,CCT7,QSOX1,PRDX5,GCLC,ILF3,LRRC59,CDKL3,PSAT1,EIF2S1,HNRNPAB,TCP1,KHSRP,HSPH1,CCT5,PTBP1,GARS1,PAICS,ARCN1,SARS1,EEF1A1,CCT3,RPL5,TARS1,HSPA4,RBM8A,APEX1,PSMA6,PLOD3,PCBP2,HINT1,PLOD1,SNRPD3,HNRNPA3,SEPTIN6,TWF1,HNRNPM,RBM3,RARS1,PUF60,PSMC3,DYNC1H1,EIF3E,NARS1,NUCB1,CDC42,CLTC,PABPC4,FKBP10,RAB7A,WARS1,U2AF2,SEPTIN9,CCT4,PFKP,ACLY,RAB11A,SFPQ,SYNCRIP,G3BP1,DDX17,ERO1A,PI4KA,FUS,HNRNPK,HNRNPA2B1,SEPTIN8,SEPTIN11,ILF2,CCT2,NONO,SEPTIN7,VCP,PXDN,HNRNPD,PABPC1,HNRNPR,FUBP1,FOLR1,CAT,AARS1,SCARB2,CCT6B,EWSR1,PRMT1,DDX39B,RNASE4 | 0.14 | 0.39 | 0.0021 |
| GO:1901681 | Molecular Function | 14 | Sulfur compound binding | HDGF,CXCL6,CXCL8,CCN2,AREL1,GSTM3,FBN1,PCOLCE,LIPG,DPYSL3,PRMT1,FSTL1,ADAMTS5,RNASE4 | 0.6 | 0.57 | 0.0021 |
| GO:0008233 | Molecular Function | 26 | Peptidase activity | HTRA3,PLAU,NPEPPS,ADAMTS7,PCSK5,MMP14,CPE,LGMN,THOP1,MMP9,MMP1,PSMB1,DNPEP,BMP1,CTSS,PAPPA,PRCP,UCHL1,HTRA1,SCPEP1,LAP3,NCSTN,PLAT,DPP3,CAPN2,ADAMTS5 | 0.4 | 0.49 | 0.0022 |
| GO:0016462 | Molecular Function | 25 | Pyrophosphatase activity | KIF5B,ARF4,HSPA9,CCT8,OLA1,CCT7,TCP1,GARS1,EEF1A1,CCT3,SEPTIN6,PSMC3,CDC42,RAB7A,SEPTIN9,CCT4,RAB11A,G3BP1,SEPTIN8,SEPTIN11,CCT2,SEPTIN7,VCP,CCT6B,DDX39B | 0.42 | 0.5 | 0.0022 |
| GO:1901363 | Molecular Function | 122 | Heterocyclic compound binding | NUDT21,HNRNPF,KIF5B,ARF4,CKAP4,PA2G4,HNRNPH3,HSPA9,LMNB1,ACTR2,HNRNPA1,PLOD2,YARS1,RPL12,TRIM28,RPS4X,HDGF,DDX5,PTK7,CCT8,SND1,VDAC1,RTRAF,HNRNPL,DDB1,HYOU1,RPL10A,SEPHS1,UBA1,HNRNPH1,UGDH,G6PD,RRBP1,RPLP0,TSN,OLA1,GOT2,CCT7,QSOX1,PRDX5,GCLC,ILF3,LRRC59,CDKL3,PSAT1,EIF2S1,HNRNPAB,TCP1,KHSRP,HSPH1,CCT5,PTBP1,GARS1,PAICS,ARCN1,SARS1,EEF1A1,CCT3,RPL5,TARS1,HSPA4,RBM8A,APEX1,PSMA6,PLOD3,PCBP2,HINT1,PLOD1,SNRPD3,HNRNPA3,SEPTIN6,TWF1,HNRNPM,RBM3,RARS1,PUF60,PSMC3,DYNC1H1,EIF3E,NARS1,NUCB1,CDC42,CLTC,PABPC4,FKBP10,RAB7A,WARS1,U2AF2,SEPTIN9,CCT4,PFKP,ACLY,RAB11A,SFPQ,SYNCRIP,G3BP1,DDX17,ERO1A,PI4KA,FUS,HNRNPK,HNRNPA2B1,SEPTIN8,SEPTIN11,ILF2,CCT2,NONO,SEPTIN7,VCP,PXDN,HNRNPD,PABPC1,HNRNPR,FUBP1,FOLR1,CAT,AARS1,CCT6B,EWSR1,PRMT1,DDX39B,RNASE4 | 0.14 | 0.39 | 0.0024 |
| GO:0050839 | Molecular Function | 15 | Cell adhesion molecule binding | CD81,LAMB1,CPE,PTK7,CCN2,LRRC59,FBN1,FERMT2,TGFBI,EMILIN1,F11R,NDRG1,SGCA,LAMA5,ITGAV | 0.56 | 0.54 | 0.0025 |
| GO:0050840 | Molecular Function | 7 | Extracellular matrix binding | ECM1,FBLN2,NID1,TGFBI,PXDN,ITGAV,ADAMTS5 | 0.94 | 0.63 | 0.0025 |
| GO:0035639 | Molecular Function | 50 | Purine ribonucleoside triphosphate binding | KIF5B,ARF4,HSPA9,ACTR2,YARS1,DDX5,PTK7,CCT8,HYOU1,SEPHS1,UBA1,OLA1,CCT7,GCLC,CDKL3,TCP1,HSPH1,CCT5,GARS1,PAICS,SARS1,EEF1A1,CCT3,TARS1,HSPA4,SEPTIN6,TWF1,RARS1,PSMC3,DYNC1H1,NARS1,CDC42,RAB7A,WARS1,SEPTIN9,CCT4,PFKP,ACLY,RAB11A,G3BP1,DDX17,PI4KA,SEPTIN8,SEPTIN11,CCT2,SEPTIN7,VCP,AARS1,CCT6B,DDX39B | 0.26 | 0.43 | 0.0028 |
| GO:0008237 | Molecular Function | 13 | Metallopeptidase activity | NPEPPS,ADAMTS7,MMP14,CPE,THOP1,MMP9,MMP1,DNPEP,BMP1,PAPPA,LAP3,DPP3,ADAMTS5 | 0.6 | 0.54 | 0.0032 |
| GO:0051082 | Molecular Function | 9 | Unfolded protein binding | HSPA9,CCT8,CCT7,TCP1,CCT5,CCT3,CCT4,CCT2,CCT6B | 0.76 | 0.56 | 0.0038 |
| GO:0008201 | Molecular Function | 11 | Heparin binding | HDGF,CXCL6,CXCL8,CCN2,AREL1,FBN1,PCOLCE,LIPG,FSTL1,ADAMTS5,RNASE4 | 0.66 | 0.53 | 0.0043 |
| GO:0008475 | Molecular Function | 3 | Procollagen-lysine 5-dioxygenase activity | PLOD2,PLOD3,PLOD1 | 1.8 | 0.62 | 0.0052 |
| GO:0032555 | Molecular Function | 50 | Purine ribonucleotide binding | KIF5B,ARF4,HSPA9,ACTR2,YARS1,DDX5,PTK7,CCT8,HYOU1,SEPHS1,UBA1,OLA1,CCT7,GCLC,CDKL3,TCP1,HSPH1,CCT5,GARS1,PAICS,SARS1,EEF1A1,CCT3,TARS1,HSPA4,SEPTIN6,TWF1,RARS1,PSMC3,DYNC1H1,NARS1,CDC42,RAB7A,WARS1,SEPTIN9,CCT4,PFKP,ACLY,RAB11A,G3BP1,DDX17,PI4KA,SEPTIN8,SEPTIN11,CCT2,SEPTIN7,VCP,AARS1,CCT6B,DDX39B | 0.25 | 0.39 | 0.0056 |
| GO:0015035 | Molecular Function | 6 | Protein-disulfide reductase activity | QSOX1,NXN,ERO1A,TXNL1,GLRX3,TXNDC17 | 0.97 | 0.56 | 0.0057 |
| GO:0016667 | Molecular Function | 7 | Oxidoreductase activity, acting on a sulfur group of donors | QSOX1,NXN,ERO1A,TXNL1,GLRX3,IFI30,TXNDC17 | 0.86 | 0.53 | 0.0064 |
| GO:0097493 | Molecular Function | 3 | Structural molecule activity conferring elasticity | EMILIN1,AHNAK,ENSECAP00000050120 | 1.68 | 0.57 | 0.0078 |
| GO:0016887 | Molecular Function | 14 | ATP hydrolysis activity | KIF5B,HSPA9,CCT8,OLA1,CCT7,TCP1,CCT3,PSMC3,CCT4,G3BP1,CCT2,VCP,CCT6B,DDX39B | 0.52 | 0.45 | 0.0080 |
| GO:0016860 | Molecular Function | 6 | Intramolecular oxidoreductase activity | ERP44,QSOX1,GPI,ENOPH1,GNPDA1,GNPDA2 | 0.92 | 0.51 | 0.0085 |
| GO:0005539 | Molecular Function | 12 | Glycosaminoglycan binding | HDGF,CXCL6,CXCL8,CCN2,AREL1,FBN1,PCOLCE,LIPG,DPYSL3,FSTL1,ADAMTS5,RNASE4 | 0.56 | 0.44 | 0.0107 |
| GO:0008238 | Molecular Function | 8 | Exopeptidase activity | NPEPPS,MMP14,CPE,DNPEP,PRCP,SCPEP1,LAP3,DPP3 | 0.73 | 0.46 | 0.0112 |
| GO:0016491 | Molecular Function | 27 | Oxidoreductase activity | PTGR1,PLOD2,SOD2,UGDH,G6PD,ALDH1A2,VAT1,QSOX1,PRDX5,ALOX12,APEX1,PLOD3,PLOD1,MDH2,LOXL1,LOXL2,NXN,ADH5,ERO1A,AKR1B1,TXNL1,GLRX3,PXDN,IFI30,CAT,TXNDC17,FASN | 0.33 | 0.38 | 0.0117 |
| GO:0140657 | Molecular Function | 21 | ATP-dependent activity | KIF5B,HSPA9,DDX5,CCT8,HYOU1,OLA1,CCT7,TCP1,HSPH1,CCT5,CCT3,HSPA4,PSMC3,DYNC1H1,CCT4,G3BP1,DDX17,CCT2,VCP,CCT6B,DDX39B | 0.38 | 0.38 | 0.0135 |
| GO:0016209 | Molecular Function | 7 | Antioxidant activity | SOD2,PRDX5,NXN,TXNL1,PXDN,CAT,TXNDC17 | 0.78 | 0.45 | 0.0138 |
| GO:0004177 | Molecular Function | 5 | Aminopeptidase activity | NPEPPS,MMP14,DNPEP,LAP3,DPP3 | 0.97 | 0.45 | 0.0168 |
| GO:0004175 | Molecular Function | 18 | Endopeptidase activity | HTRA3,PLAU,ADAMTS7,PCSK5,MMP14,LGMN,THOP1,MMP9,MMP1,PSMB1,BMP1,CTSS,PAPPA,HTRA1,NCSTN,PLAT,CAPN2,ADAMTS5 | 0.41 | 0.37 | 0.0181 |
| GO:0005524 | Molecular Function | 39 | ATP binding | KIF5B,HSPA9,ACTR2,YARS1,DDX5,PTK7,CCT8,HYOU1,SEPHS1,UBA1,CCT7,GCLC,CDKL3,TCP1,HSPH1,CCT5,GARS1,PAICS,SARS1,CCT3,TARS1,HSPA4,TWF1,RARS1,PSMC3,DYNC1H1,NARS1,WARS1,CCT4,PFKP,ACLY,G3BP1,DDX17,PI4KA,CCT2,VCP,AARS1,CCT6B,DDX39B | 0.25 | 0.31 | 0.0251 |
| GO:0016861 | Molecular Function | 3 | Intramolecular oxidoreductase activity, interconverting aldoses and ketoses | GPI,GNPDA1,GNPDA2 | 1.38 | 0.42 | 0.0275 |
| GO:0043394 | Molecular Function | 4 | Proteoglycan binding | NID1,CTSS,SDCBP,TNC | 1.06 | 0.39 | 0.0314 |
| GO:0005737 | Cellular Component | 253 | Cytoplasm | ERP29,FKBP9,NUDT21,API5,KIF5B,VPS35,PTGR1,ARF4,CKAP4,PA2G4,NAPA,HSPA9,NPEPPS,VASP,PCSK5,SLC39A14,MMP14,GDI2,PLBD2,CPE,AP2B1,PLIN3,VPS26A,PLOD2,YARS1,SGTA,PPP1CA,LGMN,RPL12,RPS4X,HDGF,PPP2CB,CCT8,MANF,SOD2,HEXB,THOP1,SERPINB9,SND1,VDAC1,RTRAF,DDB1,SMPD1,PLS3,HYOU1,RPL10A,SEC22B,COPG2,SEPHS1,KPNB1,PAFAH1B1,UBA1,IPO5,HNRNPH1,UGDH,ATIC,GANAB,G6PD,RRBP1,ALDH1A2,CORO1B,AP2A2,RPLP0,TSN,SPTBN1,OLA1,PPIA,VAT1,GOT2,CCT7,ERP44,QSOX1,PPP1CC,PRDX5,GCLC,ILF3,LRRC59,PSAT1,SEC31A,EIF2S1,HNRNPAB,TCP1,PSME2,GRN,KHSRP,GPI,HSPH1,ARPC5,GSTM3,CCT5,ENOPH1,GARS1,PAICS,ARCN1,DSTN,SARS1,TFG,EEF1A1,MXRA8,PSMA2,FERMT2,TGFBI,CCT3,PDCD6,RPL5,TARS1,HSPA4,PSMB1,DNPEP,RBM8A,ALOX12,FHL2,APEX1,PSMA6,INHBA,COPA,PLOD3,ENAH,CTSS,PCBP2,PSMD2,IMPA1,GNPDA1,HINT1,PLOD1,SNRPD3,MDH2,PRCP,SEPTIN6,PGM3,TWF1,HNRNPM,RBM3,RARS1,PSMC3,LOC100058290,LOXL2,MVP,DYNC1H1,EIF3E,LIPG,MYOF,SDCBP,DYNLL2,NIBAN2,NARS1,NUCB1,UCHL1,PGM2,SNX9,BSG,RNH1,CDC42,F11R,CLTC,NAMPT,RANBP1,PABPC4,NXN,FKBP10,RAB7A,WARS1,ADH5,LMAN2,SEPTIN9,DPYSL2,NDRG1,CCT4,PFKP,ACLY,RAB11A,SFPQ,STAM,SLC44A1,SCPEP1,SYNCRIP,G3BP1,VAPA,CAPZB,CRIP2,SEC23A,DPYSL3,ERO1A,AKR1B1,NANS,COPB1,PI4KA,TXNL1,FUS,HNRNPK,SEPTIN8,DBN1,AHNAK,SEPTIN11,AP1B1,VPS29,DCTN2,GLRX3,CCT2,LAP3,ATP6AP2,GDI1,ALDOA,COPB2,MINPP1,SERPINB8,GNPDA2,SEPTIN7,VCP,LASP1,COPG1,PDCD6IP,MAP4,PARVA,CALD1,IQGAP1,PXDN,IFI30,HNRNPD,GNS,NCSTN,PABPC1,ITGAV,ARPC2,FUBP1,CAT,MARCKS,SERPINB1,PSMD13,AARS1,PDLIM5,PSME1,PLAT,SCARB2,SRI,SPTAN1,TXNDC17,TALDO1,CCT6B,FLNC,DBNL,DPP3,PITPNB,CAPN2,PPP2R1A,PRMT1,PLEC,ENSECAP00000050120,HEXA,ARPC1B,FASN,FLNB,DDX39B | 0.19 | 0.8 | 4.28e-21 |
| GO:0005829 | Cellular Component | 107 | Cytosol | API5,KIF5B,VPS35,NPEPPS,MMP14,PLIN3,VPS26A,YARS1,SGTA,RPL12,RPS4X,PPP2CB,CCT8,SND1,PLS3,RPL10A,COPG2,KPNB1,HNRNPH1,UGDH,ATIC,G6PD,CORO1B,RPLP0,SPTBN1,CCT7,PRDX5,PSAT1,EIF2S1,TCP1,GPI,HSPH1,ARPC5,GSTM3,CCT5,GARS1,PAICS,SARS1,EEF1A1,FERMT2,CCT3,PDCD6,RPL5,DNPEP,ALOX12,PCBP2,SNRPD3,PGM3,TWF1,RARS1,LOC100058290,MVP,EIF3E,NIBAN2,NARS1,PGM2,SNX9,CLTC,RANBP1,PABPC4,NXN,RAB7A,WARS1,ADH5,DPYSL2,NDRG1,CCT4,PFKP,ACLY,SLC44A1,SCPEP1,SYNCRIP,G3BP1,SEC23A,DPYSL3,AKR1B1,NANS,COPB1,TXNL1,DBN1,AP1B1,VPS29,DCTN2,GLRX3,CCT2,ALDOA,COPB2,SERPINB8,VCP,COPG1,PARVA,IFI30,PABPC1,ITGAV,ARPC2,PSMD13,AARS1,SRI,TXNDC17,CCT6B,FLNC,DBNL,DPP3,CAPN2,HEXA,FASN,FLNB | 0.32 | 0.88 | 2.55e-11 |
| GO:0005622 | Cellular Component | 281 | Intracellular anatomical structure | ELOC,ERP29,FKBP9,NUDT21,HNRNPF,API5,KIF5B,VPS35,PTGR1,ARF4,CKAP4,PA2G4,HNRNPH3,NAPA,HSPA9,NPEPPS,VASP,PCSK5,LMNB1,SLC39A14,MMP14,ACTR2,GDI2,HNRNPA1,PLBD2,CPE,AP2B1,PLIN3,VPS26A,PLOD2,YARS1,SGTA,PPP1CA,LGMN,RPL12,TRIM28,RPS4X,HDGF,PPP2CB,DDX5,CRK,CCT8,MANF,SOD2,HEXB,THOP1,SERPINB9,SND1,VDAC1,RTRAF,HNRNPL,DDB1,SMPD1,PLS3,HYOU1,PSMD5,RPL10A,SEC22B,COPG2,SEPHS1,KPNB1,PAFAH1B1,UBA1,IPO5,HNRNPH1,UGDH,ATIC,GANAB,G6PD,RRBP1,ALDH1A2,CORO1B,AP2A2,RPLP0,TSN,SPTBN1,OLA1,PPIA,VAT1,GOT2,CCT7,ERP44,QSOX1,PPP1CC,PRDX5,GCLC,ILF3,LRRC59,CDKL3,PSAT1,SEC31A,FKBP3,EIF2S1,HNRNPAB,TCP1,PSME2,GRN,KHSRP,GPI,HSPH1,ARPC5,GSTM3,CCT5,PTBP1,ENOPH1,GARS1,PAICS,ARCN1,DSTN,SARS1,CNN3,TFG,EEF1A1,MXRA8,PSMA2,FERMT2,TGFBI,CCT3,PDCD6,RPL5,TARS1,HSPA4,PSMB1,DNPEP,RBM8A,ALOX12,FHL2,APEX1,PSMA6,INHBA,COPA,PLOD3,ENAH,CTSS,PCBP2,PSMD2,IMPA1,GNPDA1,HINT1,PLOD1,SNRPD3,MDH2,HNRNPA3,PRCP,SEPTIN6,PGM3,TWF1,HNRNPM,RBM3,RARS1,PUF60,PSMC3,LOC100058290,LOXL2,MVP,DYNC1H1,EIF3E,LIPG,MYOF,SDCBP,DYNLL2,NIBAN2,NARS1,NUCB1,UCHL1,PGM2,SNX9,BSG,RNH1,CNN2,CDC42,F11R,CLTC,NAMPT,RANBP1,PABPC4,NXN,FKBP10,RAB7A,WARS1,ADH5,LMAN2,U2AF2,SEPTIN9,DPYSL2,NDRG1,CCT4,PFKP,ACLY,RAB11A,SFPQ,STAM,SLC44A1,SCPEP1,SYNCRIP,G3BP1,VAPA,CAPZB,DDX17,CRIP2,SEC23A,DPYSL3,ERO1A,AKR1B1,NANS,COPB1,PI4KA,TXNL1,FUS,HNRNPK,SEPTIN8,DBN1,AHNAK,SEPTIN11,AP1B1,VPS29,DCTN2,GLRX3,ILF2,CCT2,LAP3,NONO,ATP6AP2,GDI1,ALDOA,COPB2,MINPP1,SERPINB8,GNPDA2,SEPTIN7,VCP,LASP1,COPG1,PDCD6IP,MAP4,PARVA,CALD1,IQGAP1,PXDN,IFI30,HNRNPD,GNS,NCSTN,PABPC1,ITGAV,ARPC2,HNRNPR,FUBP1,FOLR1,CAT,MARCKS,SERPINB1,PSMD13,AARS1,PDLIM5,PSME1,PLAT,SCARB2,SRI,SPTAN1,TXNDC17,TALDO1,CCT6B,FLNC,DBNL,DPP3,PITPNB,CAPN2,PPP2R1A,EWSR1,PRMT1,PLEC,ENSECAP00000050120,WDR43,HEXA,ARPC1B,FASN,FLNB,PSMD6,DDX39B,RNASE4 | 0.1 | 0.6 | 5.45e-11 |
| GO:0005576 | Cellular Component | 77 | Extracellular region | HTRA3,CD81,PLAU,ADAMTS7,PCSK5,MMP14,ECM1,FBLN2,LAMB1,PLBD2,CPE,LGMN,SERPING1,HDGF,COL5A2,MANF,SERPINB9,CXCL6,SMPD1,IGFBP7,IGFBP5,MMP9,STC1,CXCL8,EDIL3,DPT,ANGPTL2,CCN2,NID1,QSOX1,MXRA5,GRN,GPI,AREL1,GSTM3,GARS1,SERPINE1,FBN1,TGFBI,MMP1,PCOLCE,BMP1,INHBA,PLOD3,CTSS,PAPPA,LOXL1,LAMC1,CRLF1,LOC100058290,LOXL2,EMILIN1,LAMA2,LIPG,NUCB1,RNH1,NAMPT,LMAN2,HTRA1,SCPEP1,DPYSL3,ERO1A,HNRNPA2B1,ALDOA,SERPINB8,TNC,LAMA5,PXDN,IFI30,COL4A2,SERPINB1,PLAT,SRI,FSTL1,COL6A3,ADAMTS5,RNASE4 | 0.35 | 0.85 | 5.23e-09 |
| GO:0015629 | Cellular Component | 30 | Actin cytoskeleton | API5,ACTR2,CRK,PLS3,CORO1B,SPTBN1,ARPC5,DSTN,EEF1A1,TWF1,DYNLL2,SNX9,CNN2,SEPTIN9,CAPZB,DPYSL3,DBN1,SEPTIN11,DCTN2,ALDOA,PARVA,CALD1,IQGAP1,ARPC2,MARCKS,PDLIM5,SPTAN1,DBNL,ARPC1B,RNASE4 | 0.65 | 1.22 | 8.22e-09 |
| GO:0031012 | Cellular Component | 28 | Extracellular matrix | ADAMTS7,MMP14,ECM1,FBLN2,LAMB1,COL5A2,MMP9,ANGPTL2,CCN2,NID1,AREL1,FBN1,TGFBI,MMP1,PLOD3,LOXL1,LAMC1,LOXL2,EMILIN1,LAMA2,TNC,LAMA5,PXDN,COL4A2,COL6A3,ADAMTS5,RNASE4,HSPG2 | 0.68 | 1.24 | 1.02e-08 |
| GO:0032991 | Cellular Component | 134 | Protein-containing complex | ELOC,NUDT21,HNRNPF,KIF5B,VPS35,HNRNPH3,NAPA,PLAU,ACTR2,LAMB1,AP2B1,VPS26A,SGTA,PPP1CA,RPL12,TRIM28,RPS4X,HDGF,PPP2CB,DDX5,CRK,COL5A2,CCT8,SND1,VDAC1,RTRAF,HNRNPL,DDB1,IGFBP5,HYOU1,PSMD5,RPL10A,SEC22B,COPG2,KPNB1,PAFAH1B1,HNRNPH1,GANAB,AP2A2,RPLP0,TSN,SPTBN1,CCT7,PPP1CC,GCLC,ILF3,SEC31A,EIF2S1,HNRNPAB,TCP1,PSME2,HSPH1,ARPC5,CCT5,ARCN1,PSMA2,CCT3,PDCD6,RPL5,PSMB1,PCOLCE,RBM8A,PSMA6,INHBA,COPA,PSMD2,PLOD1,SNRPD3,SEPTIN6,CRLF1,HNRNPM,RARS1,PSMC3,EMILIN1,DYNC1H1,EIF3E,LIPG,DYNLL2,RNH1,F11R,CLTC,RANBP1,PABPC4,WARS1,U2AF2,SEPTIN9,CCT4,PFKP,SFPQ,STAM,SYNCRIP,G3BP1,CAPZB,DDX17,SEC23A,DPYSL3,COPB1,SEPTIN8,SEPTIN11,SGCA,AP1B1,VPS29,DCTN2,GLRX3,ILF2,CCT2,COPB2,TNC,SEPTIN7,LAMA5,VCP,COPG1,CALD1,IQGAP1,NCSTN,PABPC1,ITGAV,ARPC2,COL4A2,HNRNPR,CAT,PSMD13,PDLIM5,PSME1,PLAT,CCT6B,CAPN2,PPP2R1A,PRMT1,HEXA,ARPC1B,PSMD6,DDX39B,RNASE4 | 0.22 | 0.68 | 2.06e-08 |
| GO:0062023 | Cellular Component | 21 | Collagen-containing extracellular matrix | ECM1,FBLN2,LAMB1,COL5A2,ANGPTL2,NID1,FBN1,PLOD3,LOXL1,LAMC1,LOXL2,EMILIN1,LAMA2,TNC,LAMA5,PXDN,COL4A2,COL6A3,ADAMTS5,RNASE4,HSPG2 | 0.8 | 1.36 | 2.06e-08 |
| GO:0030662 | Cellular Component | 15 | Coated vesicle membrane | AP2B1,SEC22B,COPG2,AP2A2,SEC31A,ARCN1,PDCD6,COPA,CLTC,SEC23A,COPB1,ATP6AP2,COPB2,COPG1,DBNL | 0.99 | 1.54 | 3.09e-08 |
| GO:0005832 | Cellular Component | 8 | Chaperonin-containing T-complex | CCT8,CCT7,TCP1,CCT5,CCT3,CCT4,CCT2,CCT6B | 1.59 | 1.82 | 7.11e-08 |
| GO:0030117 | Cellular Component | 13 | Membrane coat | AP2B1,COPG2,AP2A2,SEC31A,ARCN1,PDCD6,COPA,CLTC,SEC23A,COPB1,AP1B1,COPB2,COPG1 | 0.98 | 1.32 | 6.13e-07 |
| GO:0030120 | Cellular Component | 11 | Vesicle coat | COPG2,AP2A2,SEC31A,ARCN1,PDCD6,COPA,CLTC,SEC23A,COPB1,COPB2,COPG1 | 1.11 | 1.4 | 6.13e-07 |
| GO:0005604 | Cellular Component | 12 | Basement membrane | LAMB1,NID1,FBN1,LAMC1,LOXL2,LAMA2,TNC,LAMA5,PXDN,COL4A2,RNASE4,HSPG2 | 1.01 | 1.32 | 8.27e-07 |
| GO:0005615 | Cellular Component | 54 | Extracellular space | CD81,PLAU,PCSK5,MMP14,ECM1,LAMB1,CPE,SERPING1,HDGF,COL5A2,MANF,SERPINB9,CXCL6,SMPD1,IGFBP7,IGFBP5,MMP9,STC1,CXCL8,ANGPTL2,QSOX1,GRN,GPI,GARS1,SERPINE1,FBN1,TGFBI,MMP1,PCOLCE,BMP1,INHBA,PLOD3,CTSS,PAPPA,LOXL1,CRLF1,LOXL2,EMILIN1,LIPG,NAMPT,LMAN2,DPYSL3,ERO1A,ALDOA,SERPINB8,TNC,PXDN,COL4A2,SERPINB1,PLAT,SRI,COL6A3,ADAMTS5,RNASE4 | 0.37 | 0.76 | 8.27e-07 |
| GO:1905369 | Cellular Component | 12 | Endopeptidase complex | PLAU,PSMD5,PSME2,PSMA2,PSMB1,PSMA6,PSMD2,PSMC3,PSMD13,PSME1,CAPN2,PSMD6 | 1.01 | 1.32 | 8.27e-07 |
| GO:0099080 | Cellular Component | 44 | Supramolecular complex | KIF5B,LMNB1,RPS4X,COL5A2,PLS3,KPNB1,PAFAH1B1,CORO1B,CCT7,PPP1CC,EIF2S1,TCP1,KHSRP,HSPH1,AREL1,FBN1,CCT3,FHL2,PSMA6,SEPTIN6,TWF1,DYNC1H1,DYNLL2,CLTC,PABPC4,SEPTIN9,NDRG1,CCT4,G3BP1,DPYSL3,DCTN2,GLRX3,CCT2,SEPTIN7,VCP,MAP4,CALD1,IQGAP1,PABPC1,COL4A2,SERPINB1,PDLIM5,SRI,PLEC | 0.42 | 0.8 | 1.05e-06 |
| GO:0030135 | Cellular Component | 17 | Coated vesicle | AP2B1,SEC22B,COPG2,AP2A2,SEC31A,ARCN1,PDCD6,COPA,SNX9,CLTC,LMAN2,SEC23A,COPB1,ATP6AP2,COPB2,COPG1,DBNL | 0.75 | 1.06 | 2.55e-06 |
| GO:0030659 | Cellular Component | 30 | Cytoplasmic vesicle membrane | VPS35,SLC39A14,CPE,AP2B1,VPS26A,SEC22B,COPG2,UBA1,AP2A2,SEC31A,ARCN1,PDCD6,COPA,SNX9,CLTC,RAB7A,NDRG1,RAB11A,STAM,SEC23A,COPB1,PI4KA,SEPTIN8,VPS29,ATP6AP2,COPB2,COPG1,SCARB2,SRI,DBNL | 0.5 | 0.83 | 4.76e-06 |
| GO:0000502 | Cellular Component | 10 | Proteasome complex | PSMD5,PSME2,PSMA2,PSMB1,PSMA6,PSMD2,PSMC3,PSMD13,PSME1,PSMD6 | 1.05 | 1.19 | 5.62e-06 |
| GO:0005938 | Cellular Component | 18 | Cell cortex | PAFAH1B1,SPTBN1,DSTN,EEF1A1,SEPTIN6,DYNC1H1,SNX9,SEPTIN9,CRIP2,SEPTIN8,SEPTIN11,GLRX3,SEPTIN7,LASP1,IQGAP1,MARCKS,SPTAN1,DBNL | 0.68 | 0.96 | 6.66e-06 |
| GO:0043229 | Cellular Component | 237 | Intracellular organelle | ELOC,ERP29,FKBP9,NUDT21,HNRNPF,API5,KIF5B,VPS35,ARF4,CKAP4,PA2G4,HNRNPH3,HSPA9,VASP,PCSK5,LMNB1,SLC39A14,MMP14,ACTR2,HNRNPA1,PLBD2,CPE,AP2B1,PLIN3,VPS26A,PLOD2,YARS1,SGTA,PPP1CA,LGMN,RPL12,TRIM28,RPS4X,HDGF,PPP2CB,DDX5,CRK,MANF,SOD2,HEXB,THOP1,SND1,VDAC1,RTRAF,HNRNPL,DDB1,SMPD1,PLS3,HYOU1,RPL10A,SEC22B,COPG2,SEPHS1,KPNB1,PAFAH1B1,UBA1,IPO5,HNRNPH1,UGDH,GANAB,G6PD,RRBP1,CORO1B,AP2A2,RPLP0,TSN,SPTBN1,PPIA,VAT1,GOT2,CCT7,ERP44,QSOX1,PPP1CC,PRDX5,ILF3,LRRC59,CDKL3,SEC31A,FKBP3,EIF2S1,HNRNPAB,TCP1,PSME2,GRN,KHSRP,GPI,HSPH1,ARPC5,PTBP1,ENOPH1,GARS1,ARCN1,DSTN,SARS1,CNN3,TFG,EEF1A1,MXRA8,PSMA2,FERMT2,TGFBI,CCT3,PDCD6,RPL5,PSMB1,RBM8A,FHL2,APEX1,PSMA6,COPA,PLOD3,ENAH,CTSS,PCBP2,PSMD2,HINT1,PLOD1,SNRPD3,MDH2,HNRNPA3,PRCP,SEPTIN6,TWF1,HNRNPM,RBM3,RARS1,PUF60,PSMC3,LOXL2,MVP,DYNC1H1,EIF3E,LIPG,MYOF,DYNLL2,NIBAN2,NUCB1,UCHL1,SNX9,BSG,RNH1,CNN2,CDC42,F11R,CLTC,NAMPT,RANBP1,PABPC4,NXN,FKBP10,RAB7A,WARS1,ADH5,LMAN2,U2AF2,SEPTIN9,NDRG1,CCT4,ACLY,RAB11A,SFPQ,STAM,SLC44A1,SYNCRIP,G3BP1,VAPA,CAPZB,DDX17,SEC23A,DPYSL3,ERO1A,COPB1,PI4KA,FUS,HNRNPK,SEPTIN8,DBN1,AHNAK,SEPTIN11,AP1B1,VPS29,DCTN2,GLRX3,ILF2,CCT2,NONO,ATP6AP2,GDI1,ALDOA,COPB2,MINPP1,SEPTIN7,VCP,LASP1,COPG1,PDCD6IP,MAP4,PARVA,CALD1,IQGAP1,PXDN,IFI30,HNRNPD,GNS,NCSTN,PABPC1,ARPC2,HNRNPR,FUBP1,FOLR1,CAT,MARCKS,SERPINB1,PSMD13,AARS1,PDLIM5,PSME1,PLAT,SCARB2,SRI,SPTAN1,FLNC,DBNL,PITPNB,CAPN2,EWSR1,PRMT1,PLEC,ENSECAP00000050120,WDR43,HEXA,ARPC1B,FASN,FLNB,DDX39B,RNASE4 | 0.09 | 0.49 | 6.66e-06 |
| GO:0031982 | Cellular Component | 55 | Vesicle | KIF5B,VPS35,CD81,SLC39A14,MMP14,CPE,AP2B1,VPS26A,LGMN,SND1,SMPD1,SEC22B,COPG2,UBA1,AP2A2,QSOX1,PRDX5,SEC31A,TCP1,GRN,GARS1,ARCN1,PDCD6,COPA,SEPTIN6,LIPG,MYOF,SNX9,F11R,CLTC,RAB7A,LMAN2,NDRG1,CCT4,RAB11A,STAM,SEC23A,COPB1,PI4KA,SEPTIN8,VPS29,DCTN2,ATP6AP2,ALDOA,COPB2,COPG1,PDCD6IP,NCSTN,SERPINB1,PLAT,SCARB2,SRI,DBNL,HEXA,FASN | 0.33 | 0.66 | 9.08e-06 |
| GO:0022624 | Cellular Component | 7 | Proteasome accessory complex | PSMD5,PSME2,PSMD2,PSMC3,PSMD13,PSME1,PSMD6 | 1.31 | 1.21 | 1.31e-05 |
| GO:0043226 | Cellular Component | 241 | Organelle | ELOC,ERP29,FKBP9,NUDT21,HNRNPF,API5,KIF5B,VPS35,ARF4,CKAP4,CD81,PA2G4,HNRNPH3,HSPA9,VASP,PCSK5,LMNB1,SLC39A14,MMP14,ACTR2,HNRNPA1,PLBD2,CPE,AP2B1,PLIN3,VPS26A,PLOD2,YARS1,SGTA,PPP1CA,LGMN,RPL12,TRIM28,RPS4X,HDGF,PPP2CB,DDX5,CRK,CCT8,MANF,SOD2,HEXB,THOP1,SND1,VDAC1,RTRAF,HNRNPL,DDB1,SMPD1,PLS3,HYOU1,RPL10A,SEC22B,COPG2,SEPHS1,KPNB1,PAFAH1B1,UBA1,IPO5,HNRNPH1,UGDH,GANAB,G6PD,RRBP1,CORO1B,AP2A2,RPLP0,TSN,SPTBN1,PPIA,VAT1,GOT2,CCT7,ERP44,QSOX1,PPP1CC,PRDX5,ILF3,LRRC59,CDKL3,SEC31A,FKBP3,EIF2S1,HNRNPAB,TCP1,PSME2,GRN,KHSRP,GPI,HSPH1,ARPC5,GSTM3,PTBP1,ENOPH1,GARS1,ARCN1,DSTN,SARS1,CNN3,TFG,EEF1A1,MXRA8,PSMA2,FERMT2,TGFBI,CCT3,PDCD6,RPL5,PSMB1,RBM8A,FHL2,APEX1,PSMA6,COPA,PLOD3,ENAH,CTSS,PCBP2,PSMD2,HINT1,PLOD1,SNRPD3,MDH2,HNRNPA3,PRCP,SEPTIN6,TWF1,HNRNPM,RBM3,RARS1,PUF60,PSMC3,LOXL2,MVP,DYNC1H1,EIF3E,LIPG,MYOF,DYNLL2,NIBAN2,NARS1,NUCB1,UCHL1,SNX9,BSG,RNH1,CNN2,CDC42,F11R,CLTC,NAMPT,RANBP1,PABPC4,NXN,FKBP10,RAB7A,WARS1,ADH5,LMAN2,U2AF2,SEPTIN9,NDRG1,CCT4,ACLY,RAB11A,SFPQ,STAM,SLC44A1,SYNCRIP,G3BP1,VAPA,CAPZB,DDX17,SEC23A,DPYSL3,ERO1A,COPB1,PI4KA,FUS,HNRNPK,SEPTIN8,DBN1,AHNAK,SEPTIN11,AP1B1,VPS29,DCTN2,GLRX3,ILF2,CCT2,NONO,ATP6AP2,GDI1,ALDOA,COPB2,MINPP1,SEPTIN7,VCP,LASP1,COPG1,PDCD6IP,MAP4,PARVA,CALD1,IQGAP1,PXDN,IFI30,HNRNPD,GNS,NCSTN,PABPC1,ARPC2,HNRNPR,FUBP1,FOLR1,CAT,MARCKS,SERPINB1,PSMD13,AARS1,PDLIM5,PSME1,PLAT,SCARB2,SRI,SPTAN1,FLNC,DBNL,PITPNB,CAPN2,EWSR1,PRMT1,PLEC,ENSECAP00000050120,WDR43,HEXA,ARPC1B,FASN,FLNB,DDX39B,RNASE4 | 0.09 | 0.47 | 1.46e-05 |
| GO:0110165 | Cellular Component | 328 | Cellular anatomical entity | ELOC,ERP29,FKBP9,HTRA3,NUDT21,HNRNPF,API5,KIF5B,VPS35,PTGR1,ARF4,CKAP4,CD81,PA2G4,HNRNPH3,NAPA,PLAU,HSPA9,NPEPPS,ADAMTS7,VASP,PCSK5,LMNB1,SLC39A14,MMP14,ECM1,ACTR2,GDI2,HNRNPA1,FBLN2,LAMB1,PLBD2,CPE,AP2B1,PLIN3,VPS26A,PLOD2,YARS1,SGTA,PPP1CA,LGMN,CSPG4,RPL12,TRIM28,RPS4X,SERPING1,HDGF,PPP2CB,DDX5,CRK,COL5A2,PTK7,CCT8,MANF,SOD2,HEXB,THOP1,SERPINB9,SND1,VDAC1,RTRAF,CXCL6,HNRNPL,DDB1,SMPD1,IGFBP7,IGFBP5,MMP9,PLS3,HYOU1,PSMD5,STC1,RPL10A,SEC22B,COPG2,SEPHS1,KPNB1,PAFAH1B1,UBA1,IPO5,HNRNPH1,UGDH,ATIC,GANAB,G6PD,RRBP1,ALDH1A2,CORO1B,AP2A2,CXCL8,EDIL3,RPLP0,TSN,DPT,SPTBN1,ANGPTL2,OLA1,PPIA,CCN2,NID1,VAT1,GOT2,CCT7,ERP44,QSOX1,PPP1CC,MXRA5,PRDX5,GCLC,ILF3,LRRC59,CDKL3,PSAT1,SEC31A,FKBP3,EIF2S1,HNRNPAB,TCP1,PSME2,GRN,KHSRP,GPI,HSPH1,ALCAM,ARPC5,AREL1,GSTM3,CCT5,PTBP1,ENOPH1,GARS1,SERPINE1,PAICS,ARCN1,DSTN,FBN1,SARS1,CNN3,TFG,EEF1A1,MXRA8,PSMA2,FERMT2,TGFBI,CCT3,PDCD6,RPL5,TARS1,MMP1,HSPA4,PSMB1,PCOLCE,DNPEP,RBM8A,ALOX12,FHL2,BMP1,APEX1,PSMA6,INHBA,COPA,PLOD3,ENAH,CTSS,PCBP2,PSMD2,PAPPA,IMPA1,GNPDA1,HINT1,PLOD1,SNRPD3,MDH2,HNRNPA3,PRCP,LOXL1,LAMC1,SEPTIN6,CRLF1,PGM3,TWF1,HNRNPM,RBM3,LOC100058329,RARS1,PUF60,PSMC3,LOC100058290,LOXL2,MVP,EMILIN1,DYNC1H1,LAMA2,EIF3E,LIPG,MYOF,SDCBP,DYNLL2,NIBAN2,NARS1,NUCB1,UCHL1,PGM2,SNX9,BSG,RNH1,CNN2,CDC42,F11R,CLTC,NAMPT,RANBP1,PABPC4,NXN,FKBP10,RAB7A,WARS1,ADH5,LMAN2,U2AF2,SEPTIN9,DPYSL2,NDRG1,CCT4,PFKP,ACLY,RAB11A,HTRA1,SFPQ,STAM,SLC44A1,SCPEP1,SYNCRIP,G3BP1,VAPA,CAPZB,DDX17,CRIP2,SEC23A,DPYSL3,ERO1A,AKR1B1,NANS,COPB1,PI4KA,TXNL1,FUS,HNRNPK,HNRNPA2B1,SEPTIN8,DBN1,AHNAK,SEPTIN11,SGCA,AP1B1,VPS29,DCTN2,GLRX3,ILF2,CCT2,LAP3,NONO,ATP6AP2,GDI1,ALDOA,COPB2,MINPP1,SERPINB8,TNC,GNPDA2,SEPTIN7,LAMA5,VCP,LASP1,COPG1,PDCD6IP,MAP4,PARVA,CALD1,IQGAP1,PXDN,IFI30,HNRNPD,GNS,NCSTN,PABPC1,ITGAV,ARPC2,COL4A2,HNRNPR,FUBP1,FOLR1,CAT,MARCKS,SERPINB1,PSMD13,AARS1,PDLIM5,PSME1,PLAT,SCARB2,SRI,SPTAN1,TXNDC17,TALDO1,CCT6B,FLNC,DBNL,DPP3,PITPNB,CAPN2,PPP2R1A,EWSR1,PRMT1,PLEC,ENSECAP00000050120,WDR43,HEXA,FSTL1,ARPC1B,COL6A3,FASN,FLNB,PSMD6,DDX39B,ADAMTS5,RNASE4,HSPG2 | 0.02 | 0.43 | 1.46e-05 |
| GO:0005856 | Cellular Component | 61 | Cytoskeleton | NUDT21,API5,KIF5B,VASP,LMNB1,MMP14,ACTR2,PPP2CB,CRK,RTRAF,PLS3,PAFAH1B1,G6PD,CORO1B,SPTBN1,CCT7,PPP1CC,TCP1,HSPH1,ARPC5,DSTN,CNN3,EEF1A1,CCT3,APEX1,ENAH,SEPTIN6,TWF1,MVP,DYNC1H1,DYNLL2,SNX9,CNN2,CDC42,CLTC,RANBP1,SEPTIN9,NDRG1,CCT4,CAPZB,DPYSL3,SEPTIN8,DBN1,SEPTIN11,DCTN2,CCT2,ALDOA,SEPTIN7,LASP1,MAP4,PARVA,CALD1,IQGAP1,ARPC2,MARCKS,PDLIM5,SPTAN1,DBNL,PLEC,ARPC1B,RNASE4 | 0.29 | 0.62 | 1.75e-05 |
| GO:0031410 | Cellular Component | 51 | Cytoplasmic vesicle | KIF5B,VPS35,SLC39A14,MMP14,CPE,AP2B1,VPS26A,LGMN,SND1,SMPD1,SEC22B,COPG2,UBA1,AP2A2,PRDX5,SEC31A,TCP1,GRN,GARS1,ARCN1,PDCD6,COPA,SEPTIN6,LIPG,MYOF,SNX9,F11R,CLTC,RAB7A,LMAN2,NDRG1,CCT4,RAB11A,STAM,SEC23A,COPB1,PI4KA,SEPTIN8,VPS29,ATP6AP2,COPB2,COPG1,PDCD6IP,NCSTN,SERPINB1,PLAT,SCARB2,SRI,DBNL,HEXA,FASN | 0.33 | 0.65 | 1.84e-05 |
| GO:0030126 | Cellular Component | 6 | COPI vesicle coat | COPG2,ARCN1,COPA,COPB1,COPB2,COPG1 | 1.41 | 1.17 | 2.51e-05 |
| GO:0043232 | Cellular Component | 101 | Intracellular non-membrane-bounded organelle | NUDT21,API5,KIF5B,CKAP4,PA2G4,HSPA9,VASP,LMNB1,MMP14,ACTR2,PLIN3,PPP1CA,RPL12,TRIM28,RPS4X,PPP2CB,DDX5,CRK,RTRAF,DDB1,SMPD1,PLS3,RPL10A,KPNB1,PAFAH1B1,UBA1,G6PD,CORO1B,RPLP0,SPTBN1,CCT7,PPP1CC,ILF3,LRRC59,EIF2S1,TCP1,KHSRP,HSPH1,ARPC5,DSTN,CNN3,EEF1A1,CCT3,RPL5,FHL2,APEX1,PSMA6,ENAH,PSMD2,SEPTIN6,TWF1,RARS1,LOXL2,MVP,DYNC1H1,DYNLL2,SNX9,CNN2,CDC42,CLTC,RANBP1,PABPC4,RAB7A,SEPTIN9,NDRG1,CCT4,SFPQ,G3BP1,CAPZB,DPYSL3,SEPTIN8,DBN1,SEPTIN11,DCTN2,GLRX3,ILF2,CCT2,ALDOA,SEPTIN7,VCP,LASP1,MAP4,PARVA,CALD1,IQGAP1,PABPC1,ARPC2,MARCKS,SERPINB1,PDLIM5,SRI,SPTAN1,FLNC,DBNL,CAPN2,EWSR1,PLEC,WDR43,ARPC1B,FLNB,RNASE4 | 0.2 | 0.55 | 2.51e-05 |
| GO:0030660 | Cellular Component | 8 | Golgi-associated vesicle membrane | COPG2,ARCN1,COPA,CLTC,COPB1,PI4KA,COPB2,COPG1 | 1.05 | 1.0 | 5.84e-05 |
| GO:0005764 | Cellular Component | 21 | Lysosome | VPS35,SLC39A14,PLBD2,VPS26A,LGMN,HEXB,SMPD1,UBA1,GRN,CTSS,PRCP,CLTC,RAB7A,ATP6AP2,IFI30,GNS,NCSTN,SERPINB1,SCARB2,CAPN2,HEXA | 0.54 | 0.74 | 7.24e-05 |
| GO:0099081 | Cellular Component | 31 | Supramolecular polymer | KIF5B,LMNB1,COL5A2,PLS3,PAFAH1B1,CORO1B,CCT7,TCP1,HSPH1,AREL1,FBN1,CCT3,FHL2,TWF1,DYNC1H1,DYNLL2,CLTC,SEPTIN9,NDRG1,CCT4,DPYSL3,DCTN2,GLRX3,CCT2,MAP4,CALD1,IQGAP1,COL4A2,PDLIM5,SRI,PLEC | 0.4 | 0.62 | 0.00015 |
| GO:1990904 | Cellular Component | 29 | Ribonucleoprotein complex | HNRNPF,HNRNPH3,RPL12,RPS4X,DDX5,SND1,HNRNPL,RPL10A,HNRNPH1,RPLP0,ILF3,EIF2S1,HNRNPAB,RPL5,RBM8A,PSMA6,SNRPD3,HNRNPM,EIF3E,PABPC4,U2AF2,SYNCRIP,G3BP1,DDX17,ILF2,IQGAP1,PABPC1,HNRNPR,DDX39B | 0.41 | 0.6 | 0.00024 |
| GO:0005940 | Cellular Component | 5 | Septin ring | SEPTIN6,SEPTIN9,SEPTIN8,SEPTIN11,SEPTIN7 | 1.36 | 0.91 | 0.00029 |
| GO:0031105 | Cellular Component | 5 | Septin complex | SEPTIN6,SEPTIN9,SEPTIN8,SEPTIN11,SEPTIN7 | 1.36 | 0.91 | 0.00029 |
| GO:0099512 | Cellular Component | 30 | Supramolecular fiber | KIF5B,LMNB1,COL5A2,PLS3,PAFAH1B1,CORO1B,CCT7,TCP1,HSPH1,AREL1,FBN1,CCT3,FHL2,TWF1,DYNC1H1,DYNLL2,CLTC,SEPTIN9,NDRG1,CCT4,DPYSL3,DCTN2,GLRX3,CCT2,MAP4,CALD1,IQGAP1,PDLIM5,SRI,PLEC | 0.39 | 0.58 | 0.00032 |
| GO:0002199 | Cellular Component | 4 | Zona pellucida receptor complex | TCP1,CCT3,CCT4,CCT2 | 1.63 | 0.92 | 0.00036 |
| GO:0098588 | Cellular Component | 40 | Bounding membrane of organelle | VPS35,PCSK5,SLC39A14,CPE,AP2B1,VPS26A,VDAC1,SMPD1,SEC22B,COPG2,UBA1,AP2A2,VAT1,QSOX1,SEC31A,GRN,GPI,ARCN1,MXRA8,PDCD6,COPA,PLOD1,CLTC,RAB7A,LMAN2,NDRG1,RAB11A,STAM,SEC23A,COPB1,PI4KA,SEPTIN8,VPS29,ATP6AP2,COPB2,COPG1,CAT,SCARB2,SRI,DBNL | 0.31 | 0.52 | 0.00052 |
| GO:0005838 | Cellular Component | 5 | Proteasome regulatory particle | PSMD5,PSMD2,PSMC3,PSMD13,PSMD6 | 1.22 | 0.78 | 0.00088 |
| GO:0032432 | Cellular Component | 7 | Actin filament bundle | PLS3,CORO1B,CNN2,SEPTIN9,SEPTIN11,MARCKS,PDLIM5 | 0.93 | 0.7 | 0.0011 |
| GO:0012505 | Cellular Component | 80 | Endomembrane system | ERP29,FKBP9,VPS35,ARF4,CKAP4,PCSK5,LMNB1,SLC39A14,MMP14,CPE,AP2B1,VPS26A,PLOD2,LGMN,MANF,SMPD1,HYOU1,SEC22B,COPG2,SEPHS1,KPNB1,PAFAH1B1,UBA1,GANAB,RRBP1,AP2A2,TSN,ERP44,QSOX1,LRRC59,SEC31A,TCP1,GRN,GARS1,ARCN1,TFG,TGFBI,PDCD6,APEX1,COPA,PLOD3,PLOD1,SEPTIN6,LOXL2,LIPG,NUCB1,UCHL1,SNX9,BSG,CLTC,RANBP1,FKBP10,RAB7A,LMAN2,NDRG1,RAB11A,STAM,VAPA,SEC23A,ERO1A,COPB1,SEPTIN8,AP1B1,VPS29,ATP6AP2,GDI1,COPB2,MINPP1,VCP,COPG1,PDCD6IP,PXDN,NCSTN,SERPINB1,PLAT,SRI,DBNL,CAPN2,HEXA,FASN | 0.19 | 0.43 | 0.0012 |
| GO:0005885 | Cellular Component | 4 | Arp2/3 protein complex | ACTR2,ARPC5,ARPC2,ARPC1B | 1.41 | 0.75 | 0.0014 |
| GO:0030864 | Cellular Component | 7 | Cortical actin cytoskeleton | SPTBN1,DSTN,EEF1A1,SNX9,IQGAP1,SPTAN1,DBNL | 0.84 | 0.59 | 0.0031 |
| GO:0030906 | Cellular Component | 3 | Retromer, cargo-selective complex | VPS35,VPS26A,VPS29 | 1.68 | 0.67 | 0.0033 |
| GO:0043227 | Cellular Component | 211 | Membrane-bounded organelle | ELOC,ERP29,FKBP9,NUDT21,HNRNPF,KIF5B,VPS35,ARF4,CKAP4,CD81,PA2G4,HNRNPH3,HSPA9,PCSK5,LMNB1,SLC39A14,MMP14,HNRNPA1,PLBD2,CPE,AP2B1,VPS26A,PLOD2,YARS1,SGTA,PPP1CA,LGMN,TRIM28,HDGF,PPP2CB,DDX5,CCT8,MANF,SOD2,HEXB,THOP1,SND1,VDAC1,RTRAF,HNRNPL,DDB1,SMPD1,HYOU1,SEC22B,COPG2,SEPHS1,KPNB1,PAFAH1B1,UBA1,IPO5,HNRNPH1,UGDH,GANAB,G6PD,RRBP1,AP2A2,RPLP0,TSN,SPTBN1,PPIA,VAT1,GOT2,ERP44,QSOX1,PPP1CC,PRDX5,ILF3,LRRC59,CDKL3,SEC31A,FKBP3,EIF2S1,HNRNPAB,TCP1,PSME2,GRN,KHSRP,GPI,HSPH1,ARPC5,GSTM3,PTBP1,ENOPH1,GARS1,ARCN1,SARS1,TFG,EEF1A1,MXRA8,PSMA2,FERMT2,TGFBI,PDCD6,RPL5,PSMB1,RBM8A,FHL2,APEX1,PSMA6,COPA,PLOD3,CTSS,PCBP2,PSMD2,HINT1,PLOD1,SNRPD3,MDH2,HNRNPA3,PRCP,SEPTIN6,HNRNPM,RBM3,RARS1,PUF60,PSMC3,LOXL2,MVP,EIF3E,LIPG,MYOF,DYNLL2,NIBAN2,NARS1,NUCB1,UCHL1,SNX9,BSG,RNH1,CDC42,F11R,CLTC,NAMPT,RANBP1,PABPC4,NXN,FKBP10,RAB7A,WARS1,ADH5,LMAN2,U2AF2,SEPTIN9,NDRG1,CCT4,ACLY,RAB11A,SFPQ,STAM,SLC44A1,SYNCRIP,G3BP1,VAPA,DDX17,SEC23A,ERO1A,COPB1,PI4KA,FUS,HNRNPK,SEPTIN8,AHNAK,AP1B1,VPS29,DCTN2,GLRX3,ILF2,NONO,ATP6AP2,GDI1,ALDOA,COPB2,MINPP1,SEPTIN7,VCP,COPG1,PDCD6IP,PARVA,IQGAP1,PXDN,IFI30,HNRNPD,GNS,NCSTN,PABPC1,ARPC2,HNRNPR,FUBP1,FOLR1,CAT,MARCKS,SERPINB1,PSMD13,AARS1,PSME1,PLAT,SCARB2,SRI,SPTAN1,DBNL,PITPNB,CAPN2,EWSR1,PRMT1,ENSECAP00000050120,WDR43,HEXA,ARPC1B,FASN,DDX39B,RNASE4 | 0.07 | 0.35 | 0.0036 |
| GO:0005783 | Cellular Component | 39 | Endoplasmic reticulum | ERP29,FKBP9,CKAP4,PLOD2,MANF,HYOU1,SEC22B,COPG2,KPNB1,UBA1,GANAB,RRBP1,TSN,ERP44,LRRC59,SEC31A,GRN,ARCN1,TFG,PDCD6,APEX1,PLOD3,PLOD1,LOXL2,UCHL1,BSG,FKBP10,LMAN2,VAPA,SEC23A,ERO1A,ATP6AP2,MINPP1,VCP,COPG1,PXDN,NCSTN,SRI,CAPN2 | 0.27 | 0.41 | 0.0043 |
| GO:0010494 | Cellular Component | 7 | Cytoplasmic stress granule | KPNB1,EIF2S1,KHSRP,PABPC4,G3BP1,VCP,PABPC1 | 0.8 | 0.55 | 0.0048 |
| GO:0042470 | Cellular Component | 7 | Melanosome | MMP14,SND1,SEC22B,CLTC,RAB7A,CCT4,FASN | 0.8 | 0.54 | 0.0051 |
| GO:0099513 | Cellular Component | 22 | Polymeric cytoskeletal fiber | KIF5B,LMNB1,PLS3,PAFAH1B1,CORO1B,CCT7,TCP1,HSPH1,CCT3,TWF1,DYNC1H1,DYNLL2,CLTC,SEPTIN9,NDRG1,CCT4,DPYSL3,DCTN2,CCT2,MAP4,IQGAP1,PDLIM5 | 0.37 | 0.42 | 0.0064 |
| GO:0031252 | Cellular Component | 14 | Cell leading edge | VASP,PAFAH1B1,CORO1B,ARPC5,EEF1A1,TWF1,CDC42,DPYSL3,ATP6AP2,PARVA,IQGAP1,PABPC1,ITGAV,DBNL | 0.49 | 0.45 | 0.0071 |
| GO:0005874 | Cellular Component | 16 | Microtubule | KIF5B,PAFAH1B1,CCT7,TCP1,HSPH1,CCT3,DYNC1H1,DYNLL2,CLTC,SEPTIN9,NDRG1,CCT4,DCTN2,CCT2,MAP4,IQGAP1 | 0.44 | 0.43 | 0.0081 |
| GO:0043231 | Cellular Component | 201 | Intracellular membrane-bounded organelle | ELOC,ERP29,FKBP9,NUDT21,HNRNPF,KIF5B,VPS35,ARF4,CKAP4,PA2G4,HNRNPH3,HSPA9,PCSK5,LMNB1,SLC39A14,MMP14,HNRNPA1,PLBD2,CPE,AP2B1,VPS26A,PLOD2,YARS1,SGTA,PPP1CA,LGMN,TRIM28,HDGF,PPP2CB,DDX5,MANF,SOD2,HEXB,THOP1,SND1,VDAC1,RTRAF,HNRNPL,DDB1,SMPD1,HYOU1,SEC22B,COPG2,SEPHS1,KPNB1,PAFAH1B1,UBA1,IPO5,HNRNPH1,UGDH,GANAB,G6PD,RRBP1,AP2A2,RPLP0,TSN,SPTBN1,PPIA,VAT1,GOT2,ERP44,QSOX1,PPP1CC,PRDX5,ILF3,LRRC59,CDKL3,SEC31A,FKBP3,EIF2S1,HNRNPAB,TCP1,PSME2,GRN,KHSRP,GPI,HSPH1,ARPC5,PTBP1,ENOPH1,GARS1,ARCN1,SARS1,TFG,EEF1A1,MXRA8,PSMA2,FERMT2,TGFBI,PDCD6,RPL5,PSMB1,RBM8A,FHL2,APEX1,PSMA6,COPA,PLOD3,CTSS,PCBP2,PSMD2,HINT1,PLOD1,SNRPD3,MDH2,HNRNPA3,PRCP,SEPTIN6,HNRNPM,RBM3,RARS1,PUF60,PSMC3,LOXL2,MVP,EIF3E,LIPG,MYOF,NIBAN2,NUCB1,UCHL1,SNX9,BSG,RNH1,F11R,CLTC,NAMPT,RANBP1,PABPC4,NXN,FKBP10,RAB7A,WARS1,ADH5,LMAN2,U2AF2,NDRG1,CCT4,ACLY,RAB11A,SFPQ,STAM,SLC44A1,SYNCRIP,G3BP1,VAPA,DDX17,SEC23A,ERO1A,COPB1,PI4KA,FUS,HNRNPK,SEPTIN8,AHNAK,AP1B1,VPS29,GLRX3,ILF2,NONO,ATP6AP2,GDI1,COPB2,MINPP1,VCP,COPG1,PDCD6IP,PARVA,IQGAP1,PXDN,IFI30,HNRNPD,GNS,NCSTN,PABPC1,ARPC2,HNRNPR,FUBP1,FOLR1,CAT,MARCKS,SERPINB1,PSMD13,AARS1,PSME1,PLAT,SCARB2,SRI,SPTAN1,DBNL,PITPNB,CAPN2,EWSR1,PRMT1,ENSECAP00000050120,WDR43,HEXA,ARPC1B,FASN,DDX39B,RNASE4 | 0.07 | 0.32 | 0.0081 |
| GO:0036464 | Cellular Component | 11 | Cytoplasmic ribonucleoprotein granule | RPS4X,KPNB1,EIF2S1,KHSRP,PSMA6,PABPC4,G3BP1,VCP,IQGAP1,PABPC1,SERPINB1 | 0.54 | 0.43 | 0.0111 |
| GO:0098637 | Cellular Component | 3 | Protein complex involved in cell-matrix adhesion | PLAU,EMILIN1,TNC | 1.38 | 0.51 | 0.0125 |
| GO:0005793 | Cellular Component | 7 | Endoplasmic reticulum-Golgi intermediate compartment | SEC22B,COPG2,ERP44,NUCB1,LMAN2,COPB1,COPG1 | 0.72 | 0.45 | 0.0130 |
| GO:0030027 | Cellular Component | 9 | Lamellipodium | VASP,CORO1B,ARPC5,CDC42,DPYSL3,PARVA,PABPC1,ITGAV,DBNL | 0.61 | 0.43 | 0.0130 |
| GO:0032153 | Cellular Component | 6 | Cell division site | PPP1CC,SEPTIN6,SEPTIN9,SEPTIN8,SEPTIN11,SEPTIN7 | 0.8 | 0.46 | 0.0130 |
| GO:0070971 | Cellular Component | 4 | Endoplasmic reticulum exit site | SEC31A,TFG,PDCD6,SEC23A | 1.06 | 0.48 | 0.0137 |
| GO:0098636 | Cellular Component | 5 | Protein complex involved in cell adhesion | PLAU,EMILIN1,SGCA,TNC,ITGAV | 0.89 | 0.46 | 0.0145 |
| GO:0031527 | Cellular Component | 3 | Filopodium membrane | VASP,SGCA,ITGAV | 1.28 | 0.46 | 0.0189 |
| GO:0001725 | Cellular Component | 5 | Stress fiber | CORO1B,CNN2,SEPTIN9,SEPTIN11,PDLIM5 | 0.85 | 0.42 | 0.0205 |
| GO:0012507 | Cellular Component | 4 | ER to Golgi transport vesicle membrane | SEC22B,SEC31A,PDCD6,SEC23A | 0.99 | 0.43 | 0.0218 |
| GO:0008540 | Cellular Component | 3 | Proteasome regulatory particle, base subcomplex | PSMD5,PSMD2,PSMC3 | 1.2 | 0.42 | 0.0268 |
| GO:0030054 | Cellular Component | 38 | Cell junction | NAPA,VASP,PTK7,VDAC1,PAFAH1B1,UBA1,SPTBN1,EIF2S1,MXRA8,FERMT2,SEPTIN6,TWF1,PUF60,SDCBP,DYNLL2,NIBAN2,CDC42,F11R,NAMPT,NDRG1,RAB11A,SEPTIN8,DBN1,SEPTIN11,SGCA,ATP6AP2,LASP1,PARVA,IQGAP1,IFI30,NCSTN,ITGAV,ARPC2,PDLIM5,PLAT,SPTAN1,DBNL,PLEC | 0.23 | 0.3 | 0.0268 |
| GO:0032311 | Cellular Component | 2 | angiogenin-PRI complex | RNH1,RNASE4 | 1.8 | 0.43 | 0.0268 |
| GO:0097180 | Cellular Component | 2 | Serine protease inhibitor complex | PLAU,PLAT | 1.8 | 0.43 | 0.0268 |
| GO:0031258 | Cellular Component | 3 | Lamellipodium membrane | VASP,CDC42,ITGAV | 1.17 | 0.4 | 0.0316 |
| GO:0030658 | Cellular Component | 8 | Transport vesicle membrane | CPE,SEC22B,SEC31A,PDCD6,CLTC,SEC23A,SEPTIN8,ATP6AP2 | 0.57 | 0.35 | 0.0322 |
| GO:0031090 | Cellular Component | 59 | Organelle membrane | VPS35,PCSK5,LMNB1,SLC39A14,CPE,AP2B1,VPS26A,PLOD2,YARS1,VDAC1,SMPD1,SEC22B,COPG2,SEPHS1,KPNB1,PAFAH1B1,UBA1,RRBP1,AP2A2,VAT1,ERP44,QSOX1,LRRC59,SEC31A,EIF2S1,GRN,GPI,ARCN1,MXRA8,PDCD6,COPA,PLOD3,PLOD1,PSMC3,NARS1,UCHL1,SNX9,BSG,CLTC,RAB7A,LMAN2,NDRG1,RAB11A,STAM,VAPA,SEC23A,ERO1A,COPB1,PI4KA,SEPTIN8,VPS29,ATP6AP2,COPB2,VCP,COPG1,CAT,SCARB2,SRI,DBNL | 0.17 | 0.28 | 0.0322 |
| GO:0030665 | Cellular Component | 5 | Clathrin-coated vesicle membrane | AP2B1,AP2A2,CLTC,ATP6AP2,DBNL | 0.78 | 0.36 | 0.0346 |
| GO:0030127 | Cellular Component | 3 | COPII vesicle coat | SEC31A,PDCD6,SEC23A | 1.14 | 0.38 | 0.0362 |
| GO:0008537 | Cellular Component | 2 | Proteasome activator complex | PSME2,PSME1 | 1.63 | 0.38 | 0.0391 |
| GO:0030134 | Cellular Component | 5 | COPII-coated ER to Golgi transport vesicle | SEC22B,SEC31A,PDCD6,LMAN2,SEC23A | 0.76 | 0.35 | 0.0391 |
| GO:0106002 | Cellular Component | 2 | mCRD-mediated mRNA stability complex | SYNCRIP,PABPC1 | 1.63 | 0.38 | 0.0391 |
| ecb04510 | KEGG Pathways | 18 | Focal adhesion | VASP,LAMB1,PPP1CA,CRK,PPP1CC,LAMC1,LAMA2,CDC42,SGCA,TNC,LAMA5,PARVA,ITGAV,COL4A2,FLNC,CAPN2,COL6A3,FLNB | 0.79 | 1.15 | 8.96e-07 |
| ecb00520 | KEGG Pathways | 9 | Amino sugar and nucleotide sugar metabolism | HEXB,UGDH,GPI,GNPDA1,PGM3,PGM2,NANS,GNPDA2,HEXA | 1.12 | 1.12 | 1.81e-05 |
| ecb03050 | KEGG Pathways | 8 | Proteasome | PSME2,PSMA2,PSMB1,PSMA6,PSMD2,PSMC3,PSMD13,PSME1 | 1.13 | 1.03 | 5.15e-05 |
| ecb00970 | KEGG Pathways | 8 | Aminoacyl-tRNA biosynthesis | YARS1,GARS1,SARS1,TARS1,RARS1,NARS1,WARS1,AARS1 | 1.09 | 1.0 | 6.41e-05 |
| ecb04141 | KEGG Pathways | 13 | Protein processing in endoplasmic reticulum | ERP29,CKAP4,HYOU1,GANAB,RRBP1,SEC31A,EIF2S1,HSPH1,LMAN2,SEC23A,ERO1A,VCP,CAPN2 | 0.72 | 0.79 | 0.00016 |
| ecb04512 | KEGG Pathways | 10 | ECM-receptor interaction | LAMB1,LAMC1,LAMA2,SGCA,TNC,LAMA5,ITGAV,COL4A2,COL6A3,HSPG2 | 0.87 | 0.85 | 0.00016 |
| ecb04144 | KEGG Pathways | 15 | Endocytosis | KIF5B,VPS35,ARF4,AP2B1,VPS26A,AP2A2,ARPC5,CDC42,CLTC,RAB7A,RAB11A,STAM,CAPZB,VPS29,ARPC2 | 0.64 | 0.73 | 0.00021 |
| ecb03015 | KEGG Pathways | 9 | mRNA surveillance pathway | NUDT21,PPP1CA,PPP2CB,PPP1CC,RBM8A,FUS,PABPC1,PPP2R1A,DDX39B | 0.83 | 0.74 | 0.00057 |
| ecb01100 | KEGG Pathways | 42 | Metabolic pathways | PLOD2,HEXB,SMPD1,SEPHS1,PAFAH1B1,UGDH,ATIC,GANAB,G6PD,ALDH1A2,GOT2,GCLC,PSAT1,GPI,GSTM3,ENOPH1,PAICS,ALOX12,PLOD3,IMPA1,GNPDA1,PLOD1,MDH2,PGM3,LOC100058329,LOC100058290,LIPG,PGM2,ADH5,ACLY,AKR1B1,NANS,PI4KA,LAP3,ALDOA,MINPP1,GNPDA2,GNS,CAT,TALDO1,HEXA,FASN | 0.3 | 0.49 | 0.00087 |
| ecb01200 | KEGG Pathways | 9 | Carbon metabolism | G6PD,GOT2,PSAT1,GPI,MDH2,ADH5,ALDOA,CAT,TALDO1 | 0.73 | 0.59 | 0.0025 |
| ecb05014 | KEGG Pathways | 16 | Amyotrophic lateral sclerosis | KIF5B,HNRNPA1,VDAC1,EIF2S1,PSMA2,PSMB1,PSMA6,PSMD2,HNRNPA3,PSMC3,FUS,HNRNPA2B1,DCTN2,VCP,CAT,PSMD13 | 0.5 | 0.52 | 0.0025 |
| ecb00030 | KEGG Pathways | 5 | Pentose phosphate pathway | G6PD,GPI,PGM2,ALDOA,TALDO1 | 1.09 | 0.64 | 0.0029 |
| ecb05100 | KEGG Pathways | 7 | Bacterial invasion of epithelial cells | CRK,ARPC5,SEPTIN6,CDC42,CLTC,SEPTIN8,ARPC2 | 0.84 | 0.6 | 0.0029 |
| ecb00480 | KEGG Pathways | 6 | Glutathione metabolism | G6PD,GCLC,GSTM3,LOC100058329,LOC100058290,LAP3 | 0.92 | 0.6 | 0.0034 |
| ecb03040 | KEGG Pathways | 9 | Spliceosome | HNRNPA1,DDX5,RBM8A,SNRPD3,HNRNPA3,PUF60,FUS,HNRNPK,DDX39B | 0.69 | 0.55 | 0.0034 |
| ecb04142 | KEGG Pathways | 9 | Lysosome | LGMN,HEXB,SMPD1,CTSS,CLTC,AP1B1,GNS,SCARB2,HEXA | 0.69 | 0.55 | 0.0034 |
| ecb05146 | KEGG Pathways | 8 | Amoebiasis | LAMB1,SERPINB9,CXCL8,LAMC1,LAMA2,RAB7A,LAMA5,COL4A2 | 0.72 | 0.53 | 0.0047 |
| ecb05205 | KEGG Pathways | 11 | Proteoglycans in cancer | PLAU,PPP1CA,DDX5,MMP9,PPP1CC,CDC42,IQGAP1,ITGAV,FLNC,FLNB,HSPG2 | 0.58 | 0.5 | 0.0047 |
| ecb05016 | KEGG Pathways | 13 | Huntington disease | KIF5B,AP2B1,SOD2,VDAC1,AP2A2,PSMA2,PSMB1,PSMA6,PSMD2,PSMC3,CLTC,DCTN2,PSMD13 | 0.5 | 0.46 | 0.0066 |
| ecb05132 | KEGG Pathways | 11 | Salmonella infection | CXCL8,ARPC5,DYNC1H1,DYNLL2,SNX9,CDC42,RAB7A,AHNAK,ARPC2,FLNC,FLNB | 0.56 | 0.47 | 0.0066 |
| ecb04612 | KEGG Pathways | 6 | Antigen processing and presentation | LGMN,PSME2,HSPA4,CTSS,IFI30,PSME1 | 0.8 | 0.49 | 0.0093 |
| ecb05222 | KEGG Pathways | 7 | Small cell lung cancer | LAMB1,LAMC1,LAMA2,SGCA,LAMA5,ITGAV,COL4A2 | 0.71 | 0.47 | 0.0103 |
| ecb05012 | KEGG Pathways | 11 | Parkinson disease | KIF5B,VDAC1,UBA1,EIF2S1,PSMA2,PSMB1,PSMA6,PSMD2,PSMC3,UCHL1,PSMD13 | 0.51 | 0.41 | 0.0127 |
| ecb00270 | KEGG Pathways | 5 | Cysteine and methionine metabolism | GOT2,GCLC,PSAT1,ENOPH1,MDH2 | 0.82 | 0.43 | 0.0184 |
| ecb03013 | KEGG Pathways | 8 | RNA transport | KPNB1,EIF2S1,EEF1A1,RBM8A,EIF3E,FUS,PABPC1,DDX39B | 0.55 | 0.34 | 0.0353 |
| ecb00010 | KEGG Pathways | 5 | Glycolysis / Gluconeogenesis | GPI,PGM2,ADH5,ALDOA,MINPP1 | 0.74 | 0.35 | 0.0368 |
| ecb00531 | KEGG Pathways | 3 | Glycosaminoglycan degradation | HEXB,GNS,HEXA | 1.03 | 0.34 | 0.0486 |
| ecb04810 | KEGG Pathways | 9 | Regulation of actin cytoskeleton | PPP1CA,CRK,PPP1CC,ARPC5,CDC42,SGCA,IQGAP1,ITGAV,ARPC2 | 0.48 | 0.3 | 0.0486 |
| ecb05165 | KEGG Pathways | 12 | Human papillomavirus infection | LAMB1,PPP2CB,LAMC1,LAMA2,CDC42,SGCA,TNC,LAMA5,ITGAV,COL4A2,PPP2R1A,COL6A3 | 0.4 | 0.29 | 0.0486 |
| MAP-6798695 | Reactome Pathways | 50 | Neutrophil degranulation | CKAP4,PA2G4,PLAU,ACTR2,GDI2,CCT8,HEXB,SERPINB9,MMP9,KPNB1,AP2A2,PPIA,VAT1,ERP44,QSOX1,GRN,GPI,ARPC5,CNN3,EEF1A1,PSMA2,MMP1,PSMB1,PRCP,PSMC3,MVP,DYNC1H1,SDCBP,PGM2,CNN2,RAB7A,ACLY,VAPA,COPB1,ILF2,CCT2,ATP6AP2,ALDOA,VCP,IQGAP1,GNS,NCSTN,ITGAV,FOLR1,CAT,SERPINB1,PSMD13,SPTAN1,DBNL,PSMD6 | 0.63 | 1.51 | 4.65e-14 |
| MAP-1474244 | Reactome Pathways | 34 | Extracellular matrix organization | MMP14,FBLN2,LAMB1,PLOD2,COL5A2,MMP9,NID1,AREL1,SERPINE1,FBN1,MMP1,PCOLCE,BMP1,PLOD3,CTSS,PLOD1,LOXL1,LAMC1,LOXL2,EMILIN1,LAMA2,BSG,F11R,HTRA1,SGCA,TNC,LAMA5,PXDN,ITGAV,COL4A2,CAPN2,COL6A3,ADAMTS5,HSPG2 | 0.79 | 1.81 | 1.80e-13 |
| MAP-168249 | Reactome Pathways | 68 | Innate Immune System | CKAP4,CD81,PA2G4,PLAU,ACTR2,GDI2,LGMN,SERPING1,PPP2CB,CRK,CCT8,HEXB,SERPINB9,MMP9,PSMD5,KPNB1,AP2A2,PPIA,VAT1,ERP44,QSOX1,PSME2,GRN,GPI,ARPC5,DSTN,CNN3,EEF1A1,PSMA2,MMP1,PSMB1,PSMA6,CTSS,PRCP,PSMC3,MVP,DYNC1H1,SDCBP,PGM2,RNH1,CNN2,CDC42,RAB7A,ACLY,SCPEP1,VAPA,COPB1,ILF2,CCT2,ATP6AP2,ALDOA,VCP,MAP4,IQGAP1,GNS,NCSTN,ITGAV,ARPC2,FOLR1,CAT,SERPINB1,PSMD13,PSME1,SPTAN1,DBNL,PPP2R1A,ARPC1B,PSMD6 | 0.47 | 1.14 | 1.73e-12 |
| MAP-392499 | Reactome Pathways | 94 | Metabolism of proteins | ELOC,FKBP9,ARF4,CKAP4,NAPA,ADAMTS7,LAMB1,CPE,YARS1,RPL12,RPS4X,CCT8,VDAC1,DDB1,IGFBP7,IGFBP5,PSMD5,RPL10A,SEC22B,COPG2,UBA1,GANAB,RRBP1,EDIL3,RPLP0,SPTBN1,CCT7,QSOX1,SEC31A,EIF2S1,TCP1,PSME2,CCT5,GARS1,ARCN1,FBN1,SARS1,TFG,EEF1A1,MXRA8,PSMA2,TGFBI,CCT3,RPL5,TARS1,PSMB1,PSMA6,INHBA,COPA,PAPPA,LAMC1,PGM3,RARS1,PSMC3,DYNC1H1,EIF3E,DYNLL2,NARS1,NUCB1,UCHL1,RNH1,PABPC4,FKBP10,WARS1,LMAN2,CCT4,RAB11A,STAM,CAPZB,DDX17,SEC23A,NANS,COPB1,DCTN2,CCT2,LAP3,ATP6AP2,COPB2,TNC,VCP,COPG1,MAP4,PABPC1,FOLR1,PSMD13,AARS1,PSME1,SPTAN1,CCT6B,DPP3,FSTL1,PSMD6,ADAMTS5,HSPG2 | 0.36 | 0.95 | 4.72e-12 |
| MAP-390471 | Reactome Pathways | 10 | Association of TriC/CCT with target proteins during biosynthesis | FKBP9,CCT8,CCT7,TCP1,CCT5,CCT3,FKBP10,CCT4,CCT2,CCT6B | 1.55 | 2.14 | 2.34e-09 |
| MAP-168256 | Reactome Pathways | 86 | Immune System | ELOC,KIF5B,CKAP4,CD81,PA2G4,PLAU,NPEPPS,ACTR2,GDI2,AP2B1,LGMN,SERPING1,PPP2CB,CRK,PTK7,CCT8,HEXB,SERPINB9,MMP9,PLS3,PSMD5,KPNB1,UBA1,AP2A2,PPIA,VAT1,ERP44,QSOX1,SEC31A,PSME2,GRN,GPI,ARPC5,DSTN,CNN3,EEF1A1,PSMA2,MMP1,PSMB1,PSMA6,CTSS,PRCP,CRLF1,PSMC3,MVP,DYNC1H1,SDCBP,DYNLL2,PGM2,RNH1,CNN2,CDC42,CLTC,RAB7A,ACLY,SCPEP1,VAPA,CAPZB,SEC23A,COPB1,AP1B1,DCTN2,ILF2,CCT2,ATP6AP2,ALDOA,VCP,MAP4,IQGAP1,IFI30,HNRNPD,GNS,NCSTN,ITGAV,ARPC2,FOLR1,CAT,SERPINB1,PSMD13,PSME1,SPTAN1,DBNL,PPP2R1A,ARPC1B,FLNB,PSMD6 | 0.31 | 0.76 | 3.76e-08 |
| MAP-199977 | Reactome Pathways | 19 | ER to Golgi Anterograde Transport | ARF4,NAPA,SEC22B,COPG2,SPTBN1,SEC31A,ARCN1,TFG,COPA,DYNC1H1,DYNLL2,LMAN2,CAPZB,SEC23A,COPB1,DCTN2,COPB2,COPG1,SPTAN1 | 0.82 | 1.31 | 8.61e-08 |
| MAP-450408 | Reactome Pathways | 12 | AUF1 (hnRNP D0) binds and destabilizes mRNA | PSMD5,HNRNPAB,PSME2,PSMA2,PSMB1,PSMA6,PSMC3,PABPC4,PABPC1,PSMD13,PSME1,PSMD6 | 1.14 | 1.58 | 8.94e-08 |
| MAP-8953854 | Reactome Pathways | 43 | Metabolism of RNA | NUDT21,HNRNPF,HNRNPH3,HNRNPA1,RPL12,RPS4X,DDX5,RTRAF,HNRNPL,PSMD5,RPL10A,HNRNPH1,RPLP0,HNRNPAB,PSME2,KHSRP,PTBP1,PSMA2,RPL5,PSMB1,RBM8A,PSMA6,PCBP2,SNRPD3,HNRNPA3,HNRNPM,RBM3,PUF60,PSMC3,PABPC4,U2AF2,SYNCRIP,FUS,HNRNPK,HNRNPA2B1,PABPC1,HNRNPR,FUBP1,PSMD13,PSME1,WDR43,PSMD6,DDX39B | 0.46 | 0.89 | 1.94e-07 |
| MAP-199991 | Reactome Pathways | 37 | Membrane Trafficking | KIF5B,ARF4,NAPA,ACTR2,GDI2,AP2B1,PLIN3,SEC22B,COPG2,PAFAH1B1,AP2A2,SPTBN1,SEC31A,ARPC5,ARCN1,TFG,COPA,DYNC1H1,DYNLL2,SNX9,CLTC,RAB7A,LMAN2,RAB11A,STAM,CAPZB,SEC23A,COPB1,AP1B1,DCTN2,GDI1,COPB2,COPG1,GNS,ARPC2,SCARB2,SPTAN1 | 0.49 | 0.88 | 6.75e-07 |
| MAP-450531 | Reactome Pathways | 14 | Regulation of mRNA stability by proteins that bind AU-rich elements | PSMD5,HNRNPAB,PSME2,KHSRP,PSMA2,PSMB1,PSMA6,PSMC3,PABPC4,PABPC1,FUBP1,PSMD13,PSME1,PSMD6 | 0.93 | 1.28 | 6.75e-07 |
| MAP-9675108 | Reactome Pathways | 24 | Nervous system development | ACTR2,LAMB1,AP2B1,MMP9,AP2A2,SPTBN1,ALCAM,ARPC5,DSTN,ENAH,LAMC1,LAMA2,SDCBP,CDC42,CLTC,DPYSL2,DPYSL3,AHNAK,NCSTN,ARPC2,PDLIM5,SPTAN1,ENSECAP00000050120,ARPC1B | 0.63 | 1.01 | 9.14e-07 |
| MAP-948021 | Reactome Pathways | 19 | Transport to the Golgi and subsequent modification | ARF4,NAPA,SEC22B,COPG2,SPTBN1,SEC31A,ARCN1,TFG,COPA,DYNC1H1,DYNLL2,LMAN2,CAPZB,SEC23A,COPB1,DCTN2,COPB2,COPG1,SPTAN1 | 0.72 | 1.07 | 1.55e-06 |
| MAP-72163 | Reactome Pathways | 21 | mRNA Splicing - Major Pathway | HNRNPF,HNRNPH3,HNRNPA1,DDX5,HNRNPL,HNRNPH1,HNRNPAB,PTBP1,RBM8A,PCBP2,SNRPD3,HNRNPA3,HNRNPM,RBM3,PUF60,U2AF2,SYNCRIP,FUS,HNRNPK,HNRNPA2B1,HNRNPR | 0.67 | 1.02 | 1.69e-06 |
| MAP-6807878 | Reactome Pathways | 14 | COPI-mediated anterograde transport | ARF4,NAPA,COPG2,SPTBN1,ARCN1,COPA,DYNC1H1,DYNLL2,CAPZB,COPB1,DCTN2,COPB2,COPG1,SPTAN1 | 0.88 | 1.17 | 2.00e-06 |
| MAP-72172 | Reactome Pathways | 21 | mRNA Splicing | HNRNPF,HNRNPH3,HNRNPA1,DDX5,HNRNPL,HNRNPH1,HNRNPAB,PTBP1,RBM8A,PCBP2,SNRPD3,HNRNPA3,HNRNPM,RBM3,PUF60,U2AF2,SYNCRIP,FUS,HNRNPK,HNRNPA2B1,HNRNPR | 0.66 | 0.98 | 2.59e-06 |
| MAP-1474228 | Reactome Pathways | 15 | Degradation of the extracellular matrix | MMP14,LAMB1,COL5A2,MMP9,NID1,FBN1,MMP1,CTSS,LAMC1,BSG,HTRA1,COL4A2,CAPN2,ADAMTS5,HSPG2 | 0.81 | 1.08 | 3.75e-06 |
| MAP-5653656 | Reactome Pathways | 38 | Vesicle-mediated transport | KIF5B,ARF4,NAPA,ACTR2,GDI2,AP2B1,PLIN3,HYOU1,SEC22B,COPG2,PAFAH1B1,AP2A2,SPTBN1,SEC31A,ARPC5,ARCN1,TFG,COPA,DYNC1H1,DYNLL2,SNX9,CLTC,RAB7A,LMAN2,RAB11A,STAM,CAPZB,SEC23A,COPB1,AP1B1,DCTN2,GDI1,COPB2,COPG1,GNS,ARPC2,SCARB2,SPTAN1 | 0.44 | 0.78 | 3.78e-06 |
| MAP-379716 | Reactome Pathways | 8 | Cytosolic tRNA aminoacylation | YARS1,GARS1,SARS1,TARS1,RARS1,NARS1,WARS1,AARS1 | 1.28 | 1.31 | 4.33e-06 |
| MAP-4086400 | Reactome Pathways | 12 | PCP/CE pathway | AP2B1,PSMD5,AP2A2,PSME2,PSMA2,PSMB1,PSMA6,PSMC3,CLTC,PSMD13,PSME1,PSMD6 | 0.92 | 1.13 | 5.55e-06 |
| MAP-446203 | Reactome Pathways | 23 | Asparagine N-linked glycosylation | ARF4,NAPA,SEC22B,COPG2,GANAB,SPTBN1,SEC31A,ARCN1,TFG,COPA,PGM3,DYNC1H1,DYNLL2,LMAN2,CAPZB,SEC23A,NANS,COPB1,DCTN2,COPB2,VCP,COPG1,SPTAN1 | 0.59 | 0.88 | 6.99e-06 |
| MAP-390466 | Reactome Pathways | 10 | Chaperonin-mediated protein folding | FKBP9,CCT8,CCT7,TCP1,CCT5,CCT3,FKBP10,CCT4,CCT2,CCT6B | 1.03 | 1.16 | 7.86e-06 |
| MAP-8856688 | Reactome Pathways | 15 | Golgi-to-ER retrograde transport | KIF5B,ARF4,NAPA,SEC22B,COPG2,PAFAH1B1,ARCN1,COPA,DYNC1H1,DYNLL2,CAPZB,COPB1,DCTN2,COPB2,COPG1 | 0.77 | 0.99 | 9.62e-06 |
| MAP-72203 | Reactome Pathways | 23 | Processing of Capped Intron-Containing Pre-mRNA | NUDT21,HNRNPF,HNRNPH3,HNRNPA1,DDX5,HNRNPL,HNRNPH1,HNRNPAB,PTBP1,RBM8A,PCBP2,SNRPD3,HNRNPA3,HNRNPM,RBM3,PUF60,U2AF2,SYNCRIP,FUS,HNRNPK,HNRNPA2B1,HNRNPR,DDX39B | 0.57 | 0.85 | 1.04e-05 |
| MAP-195253 | Reactome Pathways | 11 | Degradation of beta-catenin by the destruction complex | PPP2CB,PSMD5,PSME2,PSMA2,PSMB1,PSMA6,PSMC3,PSMD13,PSME1,PPP2R1A,PSMD6 | 0.94 | 1.09 | 1.08e-05 |
| MAP-2132295 | Reactome Pathways | 15 | MHC class II antigen presentation | KIF5B,AP2B1,LGMN,AP2A2,SEC31A,CTSS,DYNC1H1,DYNLL2,CLTC,RAB7A,CAPZB,SEC23A,AP1B1,DCTN2,IFI30 | 0.76 | 0.98 | 1.14e-05 |
| MAP-391251 | Reactome Pathways | 10 | Protein folding | FKBP9,CCT8,CCT7,TCP1,CCT5,CCT3,FKBP10,CCT4,CCT2,CCT6B | 1.01 | 1.11 | 1.14e-05 |
| MAP-1234176 | Reactome Pathways | 10 | Oxygen-dependent proline hydroxylation of Hypoxia-inducible Factor Alpha | ELOC,PSMD5,PSME2,PSMA2,PSMB1,PSMA6,PSMC3,PSMD13,PSME1,PSMD6 | 1.0 | 1.11 | 1.22e-05 |
| MAP-1236978 | Reactome Pathways | 9 | Cross-presentation of soluble exogenous antigens (endosomes) | PSMD5,PSME2,PSMA2,PSMB1,PSMA6,PSMC3,PSMD13,PSME1,PSMD6 | 1.06 | 1.12 | 1.36e-05 |
| MAP-349425 | Reactome Pathways | 9 | Autodegradation of the E3 ubiquitin ligase COP1 | PSMD5,PSME2,PSMA2,PSMB1,PSMA6,PSMC3,PSMD13,PSME1,PSMD6 | 1.06 | 1.12 | 1.36e-05 |
| MAP-350562 | Reactome Pathways | 9 | Regulation of ornithine decarboxylase (ODC) | PSMD5,PSME2,PSMA2,PSMB1,PSMA6,PSMC3,PSMD13,PSME1,PSMD6 | 1.07 | 1.13 | 1.36e-05 |
| MAP-381426 | Reactome Pathways | 14 | Regulation of Insulin-like Growth Factor (IGF) transport and uptake by Insulin-like Growth Factor Binding Proteins (IGFBPs) | CKAP4,LAMB1,IGFBP7,IGFBP5,RRBP1,EDIL3,QSOX1,FBN1,MXRA8,PAPPA,LAMC1,NUCB1,TNC,FSTL1 | 0.78 | 0.98 | 1.36e-05 |
| MAP-422475 | Reactome Pathways | 21 | Axon guidance | ACTR2,LAMB1,AP2B1,MMP9,AP2A2,SPTBN1,ALCAM,ARPC5,DSTN,ENAH,LAMC1,SDCBP,CDC42,CLTC,DPYSL2,DPYSL3,NCSTN,ARPC2,PDLIM5,SPTAN1,ARPC1B | 0.6 | 0.86 | 1.36e-05 |
| MAP-5687128 | Reactome Pathways | 11 | MAPK6/MAPK4 signaling | PSMD5,PSME2,PSMA2,PSMB1,PSMA6,PSMC3,CDC42,SEPTIN7,PSMD13,PSME1,PSMD6 | 0.92 | 1.06 | 1.36e-05 |
| MAP-69601 | Reactome Pathways | 9 | Ubiquitin Mediated Degradation of Phosphorylated Cdc25A | PSMD5,PSME2,PSMA2,PSMB1,PSMA6,PSMC3,PSMD13,PSME1,PSMD6 | 1.07 | 1.13 | 1.36e-05 |
| MAP-69610 | Reactome Pathways | 9 | p53-Independent DNA Damage Response | PSMD5,PSME2,PSMA2,PSMB1,PSMA6,PSMC3,PSMD13,PSME1,PSMD6 | 1.07 | 1.13 | 1.36e-05 |
| MAP-69613 | Reactome Pathways | 9 | p53-Independent G1/S DNA damage checkpoint | PSMD5,PSME2,PSMA2,PSMB1,PSMA6,PSMC3,PSMD13,PSME1,PSMD6 | 1.07 | 1.13 | 1.36e-05 |
| MAP-75815 | Reactome Pathways | 9 | Ubiquitin-dependent degradation of Cyclin D | PSMD5,PSME2,PSMA2,PSMB1,PSMA6,PSMC3,PSMD13,PSME1,PSMD6 | 1.07 | 1.13 | 1.36e-05 |
| MAP-4641257 | Reactome Pathways | 9 | Degradation of AXIN | PSMD5,PSME2,PSMA2,PSMB1,PSMA6,PSMC3,PSMD13,PSME1,PSMD6 | 1.04 | 1.1 | 1.63e-05 |
| MAP-597592 | Reactome Pathways | 61 | Post-translational protein modification | ELOC,ARF4,CKAP4,NAPA,ADAMTS7,LAMB1,VDAC1,DDB1,IGFBP7,IGFBP5,PSMD5,SEC22B,COPG2,UBA1,GANAB,RRBP1,EDIL3,SPTBN1,QSOX1,SEC31A,PSME2,ARCN1,FBN1,TFG,EEF1A1,MXRA8,PSMA2,PSMB1,PSMA6,COPA,LAMC1,PGM3,PSMC3,DYNC1H1,DYNLL2,NUCB1,UCHL1,RNH1,LMAN2,RAB11A,STAM,CAPZB,DDX17,SEC23A,NANS,COPB1,DCTN2,LAP3,COPB2,TNC,VCP,COPG1,MAP4,FOLR1,PSMD13,PSME1,SPTAN1,DPP3,FSTL1,PSMD6,ADAMTS5 | 0.29 | 0.62 | 1.63e-05 |
| MAP-8852276 | Reactome Pathways | 9 | The role of GTSE1 in G2/M progression after G2 checkpoint | PSMD5,PSME2,PSMA2,PSMB1,PSMA6,PSMC3,PSMD13,PSME1,PSMD6 | 1.04 | 1.1 | 1.63e-05 |
| MAP-9762114 | Reactome Pathways | 9 | GSK3B and BTRC:CUL1-mediated-degradation of NFE2L2 | PSMD5,PSME2,PSMA2,PSMB1,PSMA6,PSMC3,PSMD13,PSME1,PSMD6 | 1.04 | 1.1 | 1.63e-05 |
| MAP-1474290 | Reactome Pathways | 12 | Collagen formation | PLOD2,COL5A2,PCOLCE,BMP1,PLOD3,PLOD1,LOXL1,LOXL2,LAMA5,PXDN,COL4A2,COL6A3 | 0.84 | 1.0 | 1.74e-05 |
| MAP-4641258 | Reactome Pathways | 9 | Degradation of DVL | PSMD5,PSME2,PSMA2,PSMB1,PSMA6,PSMC3,PSMD13,PSME1,PSMD6 | 1.03 | 1.09 | 1.74e-05 |
| MAP-5607761 | Reactome Pathways | 9 | Dectin-1 mediated noncanonical NF-kB signaling | PSMD5,PSME2,PSMA2,PSMB1,PSMA6,PSMC3,PSMD13,PSME1,PSMD6 | 1.03 | 1.09 | 1.74e-05 |
| MAP-5676590 | Reactome Pathways | 9 | NIK-->noncanonical NF-kB signaling | PSMD5,PSME2,PSMA2,PSMB1,PSMA6,PSMC3,PSMD13,PSME1,PSMD6 | 1.03 | 1.09 | 1.74e-05 |
| MAP-9755511 | Reactome Pathways | 11 | KEAP1-NFE2L2 pathway | PSMD5,PSME2,PSMA2,PSMB1,PSMA6,PSMC3,VCP,PSMD13,PSME1,DPP3,PSMD6 | 0.89 | 1.02 | 1.85e-05 |
| MAP-5358346 | Reactome Pathways | 10 | Hedgehog ligand biogenesis | PSMD5,PSME2,PSMA2,PSMB1,PSMA6,PSMC3,VCP,PSMD13,PSME1,PSMD6 | 0.95 | 1.05 | 1.90e-05 |
| MAP-8854050 | Reactome Pathways | 9 | FBXL7 down-regulates AURKA during mitotic entry and in early mitosis | PSMD5,PSME2,PSMA2,PSMB1,PSMA6,PSMC3,PSMD13,PSME1,PSMD6 | 1.02 | 1.07 | 2.02e-05 |
| MAP-1234174 | Reactome Pathways | 10 | Cellular response to hypoxia | ELOC,PSMD5,PSME2,PSMA2,PSMB1,PSMA6,PSMC3,PSMD13,PSME1,PSMD6 | 0.94 | 1.04 | 2.04e-05 |
| MAP-5610780 | Reactome Pathways | 9 | Degradation of GLI1 by the proteasome | PSMD5,PSME2,PSMA2,PSMB1,PSMA6,PSMC3,PSMD13,PSME1,PSMD6 | 1.01 | 1.06 | 2.21e-05 |
| MAP-69541 | Reactome Pathways | 9 | Stabilization of p53 | PSMD5,PSME2,PSMA2,PSMB1,PSMA6,PSMC3,PSMD13,PSME1,PSMD6 | 1.01 | 1.06 | 2.21e-05 |
| MAP-8957275 | Reactome Pathways | 13 | Post-translational protein phosphorylation | CKAP4,LAMB1,IGFBP7,IGFBP5,RRBP1,EDIL3,QSOX1,FBN1,MXRA8,LAMC1,NUCB1,TNC,FSTL1 | 0.78 | 0.95 | 2.21e-05 |
| MAP-1169091 | Reactome Pathways | 9 | Activation of NF-kappaB in B cells | PSMD5,PSME2,PSMA2,PSMB1,PSMA6,PSMC3,PSMD13,PSME1,PSMD6 | 1.0 | 1.05 | 2.40e-05 |
| MAP-5610785 | Reactome Pathways | 9 | GLI3 is processed to GLI3R by the proteasome | PSMD5,PSME2,PSMA2,PSMB1,PSMA6,PSMC3,PSMD13,PSME1,PSMD6 | 1.0 | 1.05 | 2.40e-05 |
| MAP-8941858 | Reactome Pathways | 9 | Regulation of RUNX3 expression and activity | PSMD5,PSME2,PSMA2,PSMB1,PSMA6,PSMC3,PSMD13,PSME1,PSMD6 | 1.0 | 1.05 | 2.40e-05 |
| MAP-9711123 | Reactome Pathways | 15 | Cellular response to chemical stress | SOD2,PSMD5,PRDX5,PSME2,PSMA2,PSMB1,PSMA6,PSMC3,ERO1A,VCP,CAT,PSMD13,PSME1,DPP3,PSMD6 | 0.7 | 0.89 | 2.53e-05 |
| MAP-8939902 | Reactome Pathways | 9 | Regulation of RUNX2 expression and activity | PSMD5,PSME2,PSMA2,PSMB1,PSMA6,PSMC3,PSMD13,PSME1,PSMD6 | 1.0 | 1.05 | 2.54e-05 |
| MAP-187577 | Reactome Pathways | 9 | SCF(Skp2)-mediated degradation of p27/p21 | PSMD5,PSME2,PSMA2,PSMB1,PSMA6,PSMC3,PSMD13,PSME1,PSMD6 | 0.99 | 1.03 | 2.84e-05 |
| MAP-9759194 | Reactome Pathways | 9 | Nuclear events mediated by NFE2L2 | PSMD5,PSME2,PSMA2,PSMB1,PSMA6,PSMC3,PSMD13,PSME1,PSMD6 | 0.99 | 1.03 | 2.84e-05 |
| MAP-379724 | Reactome Pathways | 8 | tRNA Aminoacylation | YARS1,GARS1,SARS1,TARS1,RARS1,NARS1,WARS1,AARS1 | 1.07 | 1.06 | 2.95e-05 |
| MAP-9716542 | Reactome Pathways | 34 | Signaling by Rho GTPases, Miro GTPases and RHOBTB3 | KIF5B,CKAP4,LMNB1,ACTR2,PLIN3,PPP2CB,PAFAH1B1,RRBP1,CCT7,PPP1CC,ARPC5,DSTN,FERMT2,HINT1,TWF1,RBM3,DYNC1H1,DYNLL2,CDC42,CLTC,RAB7A,STAM,CAPZB,TXNL1,DBN1,CCT2,LAP3,VCP,MAP4,IQGAP1,ARPC2,SPTAN1,PPP2R1A,ARPC1B | 0.41 | 0.69 | 2.95e-05 |
| MAP-2022090 | Reactome Pathways | 8 | Assembly of collagen fibrils and other multimeric structures | COL5A2,PCOLCE,BMP1,LOXL1,LOXL2,LAMA5,PXDN,COL4A2 | 1.06 | 1.05 | 3.33e-05 |
| MAP-4608870 | Reactome Pathways | 9 | Asymmetric localization of PCP proteins | PSMD5,PSME2,PSMA2,PSMB1,PSMA6,PSMC3,PSMD13,PSME1,PSMD6 | 0.97 | 1.01 | 3.37e-05 |
| MAP-2243919 | Reactome Pathways | 6 | Crosslinking of collagen fibrils | PCOLCE,BMP1,LOXL1,LOXL2,PXDN,COL4A2 | 1.33 | 1.12 | 3.59e-05 |
| MAP-3000157 | Reactome Pathways | 6 | Laminin interactions | LAMB1,LAMC1,LAMA2,LAMA5,COL4A2,HSPG2 | 1.33 | 1.12 | 3.59e-05 |
| MAP-2467813 | Reactome Pathways | 15 | Separation of Sister Chromatids | PPP2CB,PSMD5,PAFAH1B1,PPP1CC,PSME2,PSMA2,PSMB1,PSMA6,PSMC3,DYNC1H1,DYNLL2,PSMD13,PSME1,PPP2R1A,PSMD6 | 0.68 | 0.86 | 3.64e-05 |
| MAP-6814122 | Reactome Pathways | 8 | Cooperation of PDCL (PhLP1) and TRiC/CCT in G-protein beta folding | CCT8,CCT7,TCP1,CCT5,CCT3,CCT4,CCT2,CCT6B | 1.05 | 1.04 | 3.64e-05 |
| MAP-8939236 | Reactome Pathways | 9 | RUNX1 regulates transcription of genes involved in differentiation of HSCs | PSMD5,PSME2,PSMA2,PSMB1,PSMA6,PSMC3,PSMD13,PSME1,PSMD6 | 0.97 | 1.0 | 3.64e-05 |
| MAP-351202 | Reactome Pathways | 9 | Metabolism of polyamines | PSMD5,PSME2,PSMA2,PSMB1,PSMA6,PSMC3,PSMD13,PSME1,PSMD6 | 0.96 | 1.0 | 3.92e-05 |
| MAP-1236975 | Reactome Pathways | 10 | Antigen processing-Cross presentation | PSMD5,PSME2,PSMA2,PSMB1,PSMA6,PSMC3,ITGAV,PSMD13,PSME1,PSMD6 | 0.88 | 0.94 | 5.08e-05 |
| MAP-194315 | Reactome Pathways | 33 | Signaling by Rho GTPases | KIF5B,CKAP4,LMNB1,ACTR2,PPP2CB,PAFAH1B1,RRBP1,CCT7,PPP1CC,ARPC5,DSTN,FERMT2,HINT1,TWF1,RBM3,DYNC1H1,DYNLL2,CDC42,CLTC,RAB7A,STAM,CAPZB,TXNL1,DBN1,CCT2,LAP3,VCP,MAP4,IQGAP1,ARPC2,SPTAN1,PPP2R1A,ARPC1B | 0.4 | 0.66 | 5.08e-05 |
| MAP-174084 | Reactome Pathways | 9 | Autodegradation of Cdh1 by Cdh1:APC/C | PSMD5,PSME2,PSMA2,PSMB1,PSMA6,PSMC3,PSMD13,PSME1,PSMD6 | 0.94 | 0.96 | 5.33e-05 |
| MAP-69202 | Reactome Pathways | 9 | Cyclin E associated events during G1/S transition | PSMD5,PSME2,PSMA2,PSMB1,PSMA6,PSMC3,PSMD13,PSME1,PSMD6 | 0.93 | 0.95 | 5.88e-05 |
| MAP-8878159 | Reactome Pathways | 10 | Transcriptional regulation by RUNX3 | PSMD5,CCN2,PSME2,PSMA2,PSMB1,PSMA6,PSMC3,PSMD13,PSME1,PSMD6 | 0.87 | 0.92 | 5.88e-05 |
| MAP-69275 | Reactome Pathways | 15 | G2/M Transition | PPP2CB,PSMD5,PAFAH1B1,PSME2,PSMA2,PSMB1,PSMA6,PSMC3,DYNC1H1,DCTN2,LAP3,PSMD13,PSME1,PPP2R1A,PSMD6 | 0.65 | 0.81 | 6.01e-05 |
| MAP-68882 | Reactome Pathways | 17 | Mitotic Anaphase | LMNB1,PPP2CB,PSMD5,KPNB1,PAFAH1B1,PPP1CC,PSME2,PSMA2,PSMB1,PSMA6,PSMC3,DYNC1H1,DYNLL2,PSMD13,PSME1,PPP2R1A,PSMD6 | 0.6 | 0.78 | 6.04e-05 |
| MAP-2555396 | Reactome Pathways | 17 | Mitotic Metaphase and Anaphase | LMNB1,PPP2CB,PSMD5,KPNB1,PAFAH1B1,PPP1CC,PSME2,PSMA2,PSMB1,PSMA6,PSMC3,DYNC1H1,DYNLL2,PSMD13,PSME1,PPP2R1A,PSMD6 | 0.6 | 0.78 | 6.24e-05 |
| MAP-3000178 | Reactome Pathways | 8 | ECM proteoglycans | LAMB1,COL5A2,SERPINE1,LAMC1,LAMA2,TNC,ITGAV,HSPG2 | 1.01 | 0.98 | 6.24e-05 |
| MAP-453274 | Reactome Pathways | 15 | Mitotic G2-G2/M phases | PPP2CB,PSMD5,PAFAH1B1,PSME2,PSMA2,PSMB1,PSMA6,PSMC3,DYNC1H1,DCTN2,LAP3,PSMD13,PSME1,PPP2R1A,PSMD6 | 0.65 | 0.81 | 6.24e-05 |
| MAP-6811442 | Reactome Pathways | 16 | Intra-Golgi and retrograde Golgi-to-ER traffic | KIF5B,ARF4,NAPA,PLIN3,SEC22B,COPG2,PAFAH1B1,ARCN1,COPA,DYNC1H1,DYNLL2,CAPZB,COPB1,DCTN2,COPB2,COPG1 | 0.62 | 0.79 | 6.24e-05 |
| MAP-69563 | Reactome Pathways | 9 | p53-Dependent G1 DNA Damage Response | PSMD5,PSME2,PSMA2,PSMB1,PSMA6,PSMC3,PSMD13,PSME1,PSMD6 | 0.93 | 0.94 | 6.24e-05 |
| MAP-69580 | Reactome Pathways | 9 | p53-Dependent G1/S DNA damage checkpoint | PSMD5,PSME2,PSMA2,PSMB1,PSMA6,PSMC3,PSMD13,PSME1,PSMD6 | 0.93 | 0.94 | 6.24e-05 |
| MAP-216083 | Reactome Pathways | 9 | Integrin cell surface interactions | COL5A2,FBN1,BSG,F11R,SGCA,TNC,ITGAV,COL4A2,HSPG2 | 0.92 | 0.94 | 6.46e-05 |
| MAP-69615 | Reactome Pathways | 9 | G1/S DNA Damage Checkpoints | PSMD5,PSME2,PSMA2,PSMB1,PSMA6,PSMC3,PSMD13,PSME1,PSMD6 | 0.92 | 0.94 | 6.46e-05 |
| MAP-69656 | Reactome Pathways | 9 | Cyclin A:Cdk2-associated events at S phase entry | PSMD5,PSME2,PSMA2,PSMB1,PSMA6,PSMC3,PSMD13,PSME1,PSMD6 | 0.92 | 0.94 | 6.46e-05 |
| MAP-69206 | Reactome Pathways | 11 | G1/S Transition | PPP2CB,PSMD5,PSME2,PSMA2,PSMB1,PSMA6,PSMC3,PSMD13,PSME1,PPP2R1A,PSMD6 | 0.8 | 0.88 | 6.49e-05 |
| MAP-174154 | Reactome Pathways | 9 | APC/C:Cdc20 mediated degradation of Securin | PSMD5,PSME2,PSMA2,PSMB1,PSMA6,PSMC3,PSMD13,PSME1,PSMD6 | 0.91 | 0.93 | 6.86e-05 |
| MAP-3858494 | Reactome Pathways | 12 | Beta-catenin independent WNT signaling | AP2B1,PSMD5,AP2A2,PSME2,PSMA2,PSMB1,PSMA6,PSMC3,CLTC,PSMD13,PSME1,PSMD6 | 0.75 | 0.86 | 6.87e-05 |
| MAP-68949 | Reactome Pathways | 9 | Orc1 removal from chromatin | PSMD5,PSME2,PSMA2,PSMB1,PSMA6,PSMC3,PSMD13,PSME1,PSMD6 | 0.9 | 0.91 | 8.28e-05 |
| MAP-5689603 | Reactome Pathways | 10 | UCH proteinases | PSMD5,PSME2,PSMA2,PSMB1,PSMA6,PSMC3,UCHL1,PSMD13,PSME1,PSMD6 | 0.84 | 0.88 | 8.30e-05 |
| MAP-1168372 | Reactome Pathways | 9 | Downstream signaling events of B Cell Receptor (BCR) | PSMD5,PSME2,PSMA2,PSMB1,PSMA6,PSMC3,PSMD13,PSME1,PSMD6 | 0.9 | 0.9 | 8.98e-05 |
| MAP-8948751 | Reactome Pathways | 9 | Regulation of PTEN stability and activity | PSMD5,PSME2,PSMA2,PSMB1,PSMA6,PSMC3,PSMD13,PSME1,PSMD6 | 0.9 | 0.9 | 8.98e-05 |
| MAP-174184 | Reactome Pathways | 9 | Cdc20:Phospho-APC/C mediated degradation of Cyclin A | PSMD5,PSME2,PSMA2,PSMB1,PSMA6,PSMC3,PSMD13,PSME1,PSMD6 | 0.89 | 0.9 | 9.72e-05 |
| MAP-69017 | Reactome Pathways | 9 | CDK-mediated phosphorylation and removal of Cdc6 | PSMD5,PSME2,PSMA2,PSMB1,PSMA6,PSMC3,PSMD13,PSME1,PSMD6 | 0.89 | 0.9 | 9.72e-05 |
| MAP-176409 | Reactome Pathways | 9 | APC/C:Cdc20 mediated degradation of mitotic proteins | PSMD5,PSME2,PSMA2,PSMB1,PSMA6,PSMC3,PSMD13,PSME1,PSMD6 | 0.88 | 0.88 | 0.00011 |
| MAP-179419 | Reactome Pathways | 9 | APC:Cdc20 mediated degradation of cell cycle proteins prior to satisfation of the cell cycle checkpoint | PSMD5,PSME2,PSMA2,PSMB1,PSMA6,PSMC3,PSMD13,PSME1,PSMD6 | 0.88 | 0.88 | 0.00011 |
| MAP-176814 | Reactome Pathways | 9 | Activation of APC/C and APC/C:Cdc20 mediated degradation of mitotic proteins | PSMD5,PSME2,PSMA2,PSMB1,PSMA6,PSMC3,PSMD13,PSME1,PSMD6 | 0.87 | 0.87 | 0.00013 |
| MAP-8878166 | Reactome Pathways | 9 | Transcriptional regulation by RUNX2 | PSMD5,PSME2,PSMA2,PSMB1,PSMA6,PSMC3,PSMD13,PSME1,PSMD6 | 0.87 | 0.86 | 0.00014 |
| MAP-174178 | Reactome Pathways | 9 | APC/C:Cdh1 mediated degradation of Cdc20 and other APC/C:Cdh1 targeted proteins in late mitosis/early G1 | PSMD5,PSME2,PSMA2,PSMB1,PSMA6,PSMC3,PSMD13,PSME1,PSMD6 | 0.86 | 0.85 | 0.00015 |
| MAP-3000171 | Reactome Pathways | 7 | Non-integrin membrane-ECM interactions | LAMB1,COL5A2,LAMC1,LAMA2,ITGAV,COL4A2,HSPG2 | 1.03 | 0.9 | 0.00016 |
| MAP-382556 | Reactome Pathways | 11 | ABC-family proteins mediated transport | PSMD5,EIF2S1,PSME2,PSMA2,PSMB1,PSMA6,PSMC3,VCP,PSMD13,PSME1,PSMD6 | 0.74 | 0.79 | 0.00017 |
| MAP-5621481 | Reactome Pathways | 10 | C-type lectin receptors (CLRs) | PSMD5,PSME2,PSMA2,PSMB1,PSMA6,PSMC3,MAP4,PSMD13,PSME1,PSMD6 | 0.78 | 0.79 | 0.00021 |
| MAP-5607764 | Reactome Pathways | 9 | CLEC7A (Dectin-1) signaling | PSMD5,PSME2,PSMA2,PSMB1,PSMA6,PSMC3,PSMD13,PSME1,PSMD6 | 0.83 | 0.8 | 0.00025 |
| MAP-5632684 | Reactome Pathways | 9 | Hedgehog on state | PSMD5,PSME2,PSMA2,PSMB1,PSMA6,PSMC3,PSMD13,PSME1,PSMD6 | 0.83 | 0.8 | 0.00025 |
| MAP-75205 | Reactome Pathways | 5 | Dissolution of Fibrin Clot | PLAU,SERPINB9,SERPINE1,SERPINB8,PLAT | 1.27 | 0.9 | 0.00029 |
| MAP-1566948 | Reactome Pathways | 7 | Elastic fibre formation | FBLN2,AREL1,FBN1,LOXL1,LOXL2,EMILIN1,ITGAV | 0.97 | 0.82 | 0.00033 |
| MAP-9619665 | Reactome Pathways | 5 | EGR2 and SOX10-mediated initiation of Schwann cell myelination | LAMB1,LAMC1,LAMA2,AHNAK,ENSECAP00000050120 | 1.25 | 0.87 | 0.00036 |
| MAP-2682334 | Reactome Pathways | 9 | EPH-Ephrin signaling | ACTR2,MMP9,ARPC5,DSTN,SDCBP,CDC42,NCSTN,ARPC2,ARPC1B | 0.8 | 0.75 | 0.00039 |
| MAP-174143 | Reactome Pathways | 9 | APC/C-mediated degradation of cell cycle proteins | PSMD5,PSME2,PSMA2,PSMB1,PSMA6,PSMC3,PSMD13,PSME1,PSMD6 | 0.79 | 0.75 | 0.00041 |
| MAP-453276 | Reactome Pathways | 9 | Regulation of mitotic cell cycle | PSMD5,PSME2,PSMA2,PSMB1,PSMA6,PSMC3,PSMD13,PSME1,PSMD6 | 0.79 | 0.75 | 0.00041 |
| MAP-162582 | Reactome Pathways | 75 | Signal Transduction | KIF5B,VPS35,CKAP4,PCSK5,LMNB1,ACTR2,LAMB1,AP2B1,PLIN3,VPS26A,PPP1CA,PPP2CB,DDX5,CRK,COL5A2,CXCL6,MMP9,PSMD5,PAFAH1B1,RRBP1,ALDH1A2,AP2A2,CXCL8,SPTBN1,CCT7,PPP1CC,PSME2,ARPC5,AREL1,PTBP1,DSTN,FBN1,PSMA2,FERMT2,PSMB1,PSMA6,PSMD2,HINT1,LAMC1,TWF1,HNRNPM,RBM3,PSMC3,DYNC1H1,LAMA2,DYNLL2,CDC42,F11R,CLTC,RAB7A,SFPQ,STAM,CAPZB,TXNL1,DBN1,VPS29,CCT2,LAP3,NONO,SEPTIN7,VCP,MAP4,IQGAP1,NCSTN,ITGAV,ARPC2,PSMD13,PSME1,PLAT,SPTAN1,PPP2R1A,PRMT1,FSTL1,ARPC1B,PSMD6 | 0.2 | 0.47 | 0.00049 |
| MAP-453279 | Reactome Pathways | 11 | Mitotic G1 phase and G1/S transition | PPP2CB,PSMD5,PSME2,PSMA2,PSMB1,PSMA6,PSMC3,PSMD13,PSME1,PPP2R1A,PSMD6 | 0.68 | 0.69 | 0.00051 |
| MAP-5668541 | Reactome Pathways | 9 | TNFR2 non-canonical NF-kB pathway | PSMD5,PSME2,PSMA2,PSMB1,PSMA6,PSMC3,PSMD13,PSME1,PSMD6 | 0.78 | 0.72 | 0.00054 |
| MAP-69052 | Reactome Pathways | 9 | Switching of origins to a post-replicative state | PSMD5,PSME2,PSMA2,PSMB1,PSMA6,PSMC3,PSMD13,PSME1,PSMD6 | 0.78 | 0.72 | 0.00054 |
| MAP-6811434 | Reactome Pathways | 10 | COPI-dependent Golgi-to-ER retrograde traffic | KIF5B,ARF4,NAPA,SEC22B,COPG2,ARCN1,COPA,COPB1,COPB2,COPG1 | 0.72 | 0.7 | 0.00058 |
| MAP-195721 | Reactome Pathways | 17 | Signaling by WNT | VPS35,AP2B1,VPS26A,PPP2CB,PSMD5,AP2A2,PSME2,PSMA2,PSMB1,PSMA6,PSMC3,CLTC,VPS29,PSMD13,PSME1,PPP2R1A,PSMD6 | 0.5 | 0.6 | 0.00072 |
| MAP-8953897 | Reactome Pathways | 28 | Cellular responses to stimuli | ELOC,HSPA9,HDGF,SOD2,HYOU1,PSMD5,PRDX5,EIF2S1,PSME2,HSPH1,EEF1A1,PSMA2,HSPA4,PSMB1,PSMA6,PSMC3,DYNC1H1,DYNLL2,CAPZB,CRIP2,ERO1A,DCTN2,VCP,CAT,PSMD13,PSME1,DPP3,PSMD6 | 0.36 | 0.52 | 0.00086 |
| MAP-9013420 | Reactome Pathways | 6 | RHOU GTPase cycle | CDC42,CLTC,STAM,TXNL1,IQGAP1,SPTAN1 | 0.99 | 0.74 | 0.00090 |
| MAP-71291 | Reactome Pathways | 16 | Metabolism of amino acids and derivatives | PSMD5,SEPHS1,GOT2,PSAT1,PSME2,ENOPH1,SARS1,PSMA2,PSMB1,PSMA6,RARS1,PSMC3,SLC44A1,PSMD13,PSME1,PSMD6 | 0.51 | 0.59 | 0.00091 |
| MAP-9706574 | Reactome Pathways | 6 | RHOBTB GTPase Cycle | CCT7,TWF1,RBM3,TXNL1,DBN1,CCT2 | 0.97 | 0.71 | 0.0011 |
| MAP-72766 | Reactome Pathways | 18 | Translation | YARS1,RPL12,RPS4X,RPL10A,RPLP0,EIF2S1,GARS1,SARS1,EEF1A1,RPL5,TARS1,RARS1,EIF3E,NARS1,PABPC4,WARS1,PABPC1,AARS1 | 0.46 | 0.55 | 0.0012 |
| MAP-2262752 | Reactome Pathways | 27 | Cellular responses to stress | ELOC,HSPA9,HDGF,SOD2,HYOU1,PSMD5,PRDX5,EIF2S1,PSME2,HSPH1,EEF1A1,PSMA2,HSPA4,PSMB1,PSMA6,PSMC3,DYNC1H1,DYNLL2,CAPZB,ERO1A,DCTN2,VCP,CAT,PSMD13,PSME1,DPP3,PSMD6 | 0.36 | 0.5 | 0.0013 |
| MAP-70326 | Reactome Pathways | 7 | Glucose metabolism | GOT2,GPI,GNPDA1,MDH2,PFKP,ALDOA,GNPDA2 | 0.83 | 0.64 | 0.0018 |
| MAP-9013424 | Reactome Pathways | 6 | RHOV GTPase cycle | CDC42,CLTC,TXNL1,LAP3,IQGAP1,SPTAN1 | 0.93 | 0.66 | 0.0018 |
| MAP-6807070 | Reactome Pathways | 9 | PTEN Regulation | PSMD5,PSME2,PSMA2,PSMB1,PSMA6,PSMC3,PSMD13,PSME1,PSMD6 | 0.69 | 0.6 | 0.0019 |
| MAP-71387 | Reactome Pathways | 15 | Metabolism of carbohydrates | CSPG4,HEXB,G6PD,GOT2,GPI,GNPDA1,MDH2,PGM2,PFKP,ALDOA,GNPDA2,GNS,TALDO1,HEXA,HSPG2 | 0.5 | 0.54 | 0.0019 |
| MAP-68867 | Reactome Pathways | 10 | Assembly of the pre-replicative complex | PSMD5,KPNB1,PSME2,PSMA2,PSMB1,PSMA6,PSMC3,PSMD13,PSME1,PSMD6 | 0.64 | 0.58 | 0.0020 |
| MAP-3928662 | Reactome Pathways | 6 | EPHB-mediated forward signaling | ACTR2,ARPC5,DSTN,CDC42,ARPC2,ARPC1B | 0.91 | 0.64 | 0.0021 |
| MAP-5610787 | Reactome Pathways | 9 | Hedgehog off state | PSMD5,PSME2,PSMA2,PSMB1,PSMA6,PSMC3,PSMD13,PSME1,PSMD6 | 0.68 | 0.59 | 0.0022 |
| MAP-5683057 | Reactome Pathways | 17 | MAPK family signaling cascades | PPP2CB,PSMD5,SPTBN1,PPP1CC,PSME2,PSMA2,PSMB1,PSMA6,PSMC3,CDC42,SEPTIN7,IQGAP1,PSMD13,PSME1,SPTAN1,PPP2R1A,PSMD6 | 0.45 | 0.51 | 0.0024 |
| MAP-8875878 | Reactome Pathways | 5 | MET promotes cell motility | LAMB1,CRK,COL5A2,LAMC1,LAMA2 | 1.01 | 0.63 | 0.0028 |
| MAP-202424 | Reactome Pathways | 9 | Downstream TCR signaling | PSMD5,PSME2,PSMA2,PSMB1,PSMA6,PSMC3,PSMD13,PSME1,PSMD6 | 0.67 | 0.56 | 0.0029 |
| MAP-9020702 | Reactome Pathways | 9 | Interleukin-1 signaling | PSMD5,PSME2,PSMA2,PSMB1,PSMA6,PSMC3,PSMD13,PSME1,PSMD6 | 0.67 | 0.56 | 0.0029 |
| MAP-1650814 | Reactome Pathways | 8 | Collagen biosynthesis and modifying enzymes | PLOD2,COL5A2,PCOLCE,BMP1,PLOD3,PLOD1,COL4A2,COL6A3 | 0.71 | 0.56 | 0.0032 |
| MAP-69239 | Reactome Pathways | 9 | Synthesis of DNA | PSMD5,PSME2,PSMA2,PSMB1,PSMA6,PSMC3,PSMD13,PSME1,PSMD6 | 0.65 | 0.54 | 0.0034 |
| MAP-68886 | Reactome Pathways | 19 | M Phase | LMNB1,PPP2CB,PSMD5,KPNB1,PAFAH1B1,PPP1CC,PSME2,PSMA2,PSMB1,PSMA6,PSMC3,DYNC1H1,DYNLL2,DCTN2,LAP3,PSMD13,PSME1,PPP2R1A,PSMD6 | 0.4 | 0.46 | 0.0039 |
| MAP-9013422 | Reactome Pathways | 5 | RHOBTB1 GTPase cycle | CCT7,RBM3,TXNL1,DBN1,CCT2 | 0.97 | 0.6 | 0.0039 |
| MAP-373760 | Reactome Pathways | 6 | L1CAM interactions | LAMB1,AP2B1,AP2A2,ALCAM,LAMC1,CLTC | 0.84 | 0.57 | 0.0043 |
| MAP-69002 | Reactome Pathways | 10 | DNA Replication Pre-Initiation | PSMD5,KPNB1,PSME2,PSMA2,PSMB1,PSMA6,PSMC3,PSMD13,PSME1,PSMD6 | 0.6 | 0.51 | 0.0043 |
| MAP-9012999 | Reactome Pathways | 21 | RHO GTPase cycle | CKAP4,LMNB1,RRBP1,CCT7,FERMT2,HINT1,TWF1,RBM3,CDC42,CLTC,RAB7A,STAM,CAPZB,TXNL1,DBN1,CCT2,LAP3,VCP,MAP4,IQGAP1,SPTAN1 | 0.37 | 0.45 | 0.0043 |
| MAP-5358351 | Reactome Pathways | 10 | Signaling by Hedgehog | PSMD5,PSME2,PSMA2,PSMB1,PSMA6,PSMC3,VCP,PSMD13,PSME1,PSMD6 | 0.59 | 0.51 | 0.0046 |
| MAP-8874081 | Reactome Pathways | 4 | MET activates PTK2 signaling | LAMB1,COL5A2,LAMC1,LAMA2 | 1.13 | 0.6 | 0.0046 |
| MAP-1280218 | Reactome Pathways | 34 | Adaptive Immune System | ELOC,KIF5B,CD81,NPEPPS,AP2B1,LGMN,PPP2CB,PTK7,PSMD5,UBA1,AP2A2,SEC31A,PSME2,PSMA2,PSMB1,PSMA6,CTSS,PSMC3,DYNC1H1,DYNLL2,CDC42,CLTC,RAB7A,SCPEP1,CAPZB,SEC23A,AP1B1,DCTN2,IFI30,ITGAV,PSMD13,PSME1,PPP2R1A,PSMD6 | 0.27 | 0.4 | 0.0056 |
| MAP-1266738 | Reactome Pathways | 24 | Developmental Biology | ACTR2,LAMB1,AP2B1,MMP9,AP2A2,SPTBN1,ALCAM,ARPC5,DSTN,ENAH,LAMC1,LAMA2,SDCBP,CDC42,CLTC,DPYSL2,DPYSL3,AHNAK,NCSTN,ARPC2,PDLIM5,SPTAN1,ENSECAP00000050120,ARPC1B | 0.33 | 0.41 | 0.0061 |
| MAP-1430728 | Reactome Pathways | 57 | Metabolism | PTGR1,PLBD2,PLIN3,CSPG4,HEXB,VDAC1,SMPD1,PSMD5,SEPHS1,KPNB1,UGDH,ATIC,G6PD,GOT2,GCLC,PSAT1,PSME2,GPI,GSTM3,ENOPH1,PAICS,SARS1,PSMA2,PSMB1,ALOX12,FHL2,PSMA6,IMPA1,GNPDA1,MDH2,LOC100058329,RARS1,PSMC3,LOC100058290,PGM2,BSG,NAMPT,ADH5,PFKP,ACLY,SLC44A1,AKR1B1,PI4KA,ALDOA,MINPP1,GNPDA2,GNS,FOLR1,MARCKS,PSMD13,PSME1,TALDO1,PITPNB,HEXA,FASN,PSMD6,HSPG2 | 0.19 | 0.37 | 0.0063 |
| MAP-195258 | Reactome Pathways | 15 | RHO GTPase Effectors | KIF5B,ACTR2,PPP2CB,PAFAH1B1,RRBP1,PPP1CC,ARPC5,DSTN,DYNC1H1,DYNLL2,CDC42,IQGAP1,ARPC2,PPP2R1A,ARPC1B | 0.44 | 0.44 | 0.0067 |
| MAP-2129379 | Reactome Pathways | 5 | Molecules associated with elastic fibres | FBLN2,AREL1,FBN1,EMILIN1,ITGAV | 0.9 | 0.53 | 0.0072 |
| MAP-202403 | Reactome Pathways | 9 | TCR signaling | PSMD5,PSME2,PSMA2,PSMB1,PSMA6,PSMC3,PSMD13,PSME1,PSMD6 | 0.59 | 0.46 | 0.0083 |
| MAP-449147 | Reactome Pathways | 16 | Signaling by Interleukins | PPP2CB,CRK,PLS3,PSMD5,PSME2,DSTN,PSMA2,PSMB1,PSMA6,CRLF1,PSMC3,HNRNPD,PSMD13,PSME1,PPP2R1A,PSMD6 | 0.41 | 0.42 | 0.0083 |
| MAP-70171 | Reactome Pathways | 5 | Glycolysis | GPI,GNPDA1,PFKP,ALDOA,GNPDA2 | 0.88 | 0.51 | 0.0086 |
| MAP-446652 | Reactome Pathways | 9 | Interleukin-1 family signaling | PSMD5,PSME2,PSMA2,PSMB1,PSMA6,PSMC3,PSMD13,PSME1,PSMD6 | 0.58 | 0.44 | 0.0102 |
| MAP-5663213 | Reactome Pathways | 5 | RHO GTPases Activate WASPs and WAVEs | ACTR2,ARPC5,CDC42,ARPC2,ARPC1B | 0.86 | 0.49 | 0.0103 |
| MAP-9006934 | Reactome Pathways | 19 | Signaling by Receptor Tyrosine Kinases | PCSK5,LAMB1,AP2B1,PPP2CB,CRK,COL5A2,MMP9,AP2A2,PTBP1,LAMC1,HNRNPM,LAMA2,CDC42,CLTC,STAM,NCSTN,ITGAV,PLAT,PPP2R1A | 0.36 | 0.39 | 0.0104 |
| MAP-5689880 | Reactome Pathways | 11 | Ub-specific processing proteases | VDAC1,PSMD5,PSME2,PSMA2,PSMB1,PSMA6,PSMC3,LAP3,PSMD13,PSME1,PSMD6 | 0.5 | 0.42 | 0.0111 |
| MAP-5658442 | Reactome Pathways | 4 | Regulation of RAS by GAPs | PSMD5,PSMB1,PSME1,PSMD6 | 0.99 | 0.49 | 0.0121 |
| MAP-69242 | Reactome Pathways | 9 | S Phase | PSMD5,PSME2,PSMA2,PSMB1,PSMA6,PSMC3,PSMD13,PSME1,PSMD6 | 0.56 | 0.43 | 0.0123 |
| MAP-177504 | Reactome Pathways | 3 | Retrograde neurotrophin signalling | AP2B1,AP2A2,CLTC | 1.24 | 0.5 | 0.0126 |
| MAP-5140745 | Reactome Pathways | 3 | WNT5A-dependent internalization of FZD2, FZD5 and ROR2 | AP2B1,AP2A2,CLTC | 1.24 | 0.5 | 0.0126 |
| MAP-446353 | Reactome Pathways | 4 | Cell-extracellular matrix interactions | VASP,FERMT2,PARVA,FLNC | 0.98 | 0.47 | 0.0134 |
| MAP-69306 | Reactome Pathways | 10 | DNA Replication | PSMD5,KPNB1,PSME2,PSMA2,PSMB1,PSMA6,PSMC3,PSMD13,PSME1,PSMD6 | 0.52 | 0.41 | 0.0139 |
| MAP-204005 | Reactome Pathways | 6 | COPII-mediated vesicle transport | NAPA,SEC22B,SEC31A,TFG,LMAN2,SEC23A | 0.7 | 0.42 | 0.0177 |
| MAP-5099900 | Reactome Pathways | 3 | WNT5A-dependent internalization of FZD4 | AP2B1,AP2A2,CLTC | 1.17 | 0.46 | 0.0183 |
| MAP-6806834 | Reactome Pathways | 6 | Signaling by MET | LAMB1,CRK,COL5A2,LAMC1,LAMA2,STAM | 0.69 | 0.41 | 0.0198 |
| MAP-8878171 | Reactome Pathways | 9 | Transcriptional regulation by RUNX1 | PSMD5,PSME2,PSMA2,PSMB1,PSMA6,PSMC3,PSMD13,PSME1,PSMD6 | 0.52 | 0.38 | 0.0212 |
| MAP-156590 | Reactome Pathways | 4 | Glutathione conjugation | GCLC,GSTM3,LOC100058329,LOC100058290 | 0.9 | 0.41 | 0.0227 |
| MAP-1442490 | Reactome Pathways | 5 | Collagen degradation | MMP14,COL5A2,MMP9,MMP1,COL4A2 | 0.76 | 0.4 | 0.0236 |
| MAP-1257604 | Reactome Pathways | 11 | PIP3 activates AKT signaling | PPP2CB,PSMD5,PSME2,PSMA2,PSMB1,PSMA6,PSMC3,PSMD13,PSME1,PPP2R1A,PSMD6 | 0.45 | 0.35 | 0.0244 |
| MAP-9696264 | Reactome Pathways | 5 | RND3 GTPase cycle | CKAP4,RRBP1,RBM3,TXNL1,MAP4 | 0.76 | 0.39 | 0.0250 |
| MAP-8951664 | Reactome Pathways | 13 | Neddylation | ELOC,DDB1,PSMD5,PSME2,PSMA2,PSMB1,PSMA6,PSMC3,VCP,PSMD13,PSME1,DPP3,PSMD6 | 0.4 | 0.34 | 0.0262 |
| MAP-1500931 | Reactome Pathways | 8 | Cell-Cell communication | VASP,FERMT2,F11R,LAMA5,PARVA,IQGAP1,FLNC,RNASE4 | 0.55 | 0.36 | 0.0263 |
| MAP-9696270 | Reactome Pathways | 5 | RND2 GTPase cycle | CKAP4,RRBP1,RBM3,TXNL1,MAP4 | 0.74 | 0.38 | 0.0284 |
| MAP-2024101 | Reactome Pathways | 3 | CS/DS degradation | CSPG4,HEXB,HEXA | 1.08 | 0.4 | 0.0288 |
| MAP-447115 | Reactome Pathways | 4 | Interleukin-12 family signaling | PLS3,DSTN,CRLF1,HNRNPD | 0.86 | 0.39 | 0.0292 |
| MAP-446728 | Reactome Pathways | 7 | Cell junction organization | VASP,FERMT2,F11R,LAMA5,PARVA,FLNC,RNASE4 | 0.59 | 0.36 | 0.0293 |
| MAP-2871837 | Reactome Pathways | 9 | FCERI mediated NF-kB activation | PSMD5,PSME2,PSMA2,PSMB1,PSMA6,PSMC3,PSMD13,PSME1,PSMD6 | 0.5 | 0.34 | 0.0296 |
| MAP-6811436 | Reactome Pathways | 5 | COPI-independent Golgi-to-ER retrograde traffic | PAFAH1B1,DYNC1H1,DYNLL2,CAPZB,DCTN2 | 0.73 | 0.37 | 0.0296 |
| MAP-8856828 | Reactome Pathways | 9 | Clathrin-mediated endocytosis | ACTR2,AP2B1,AP2A2,ARPC5,SNX9,CLTC,STAM,ARPC2,SCARB2 | 0.49 | 0.34 | 0.0303 |
| MAP-1280215 | Reactome Pathways | 19 | Cytokine Signaling in Immune system | ELOC,PPP2CB,CRK,PLS3,PSMD5,KPNB1,PSME2,DSTN,PSMA2,PSMB1,PSMA6,CRLF1,PSMC3,HNRNPD,PSMD13,PSME1,PPP2R1A,FLNB,PSMD6 | 0.31 | 0.3 | 0.0350 |
| MAP-9013405 | Reactome Pathways | 5 | RHOD GTPase cycle | LMNB1,HINT1,RAB7A,CAPZB,DBN1 | 0.71 | 0.35 | 0.0354 |
| MAP-8866427 | Reactome Pathways | 3 | VLDLR internalisation and degradation | AP2B1,AP2A2,CLTC | 1.03 | 0.37 | 0.0370 |
| MAP-156827 | Reactome Pathways | 9 | L13a-mediated translational silencing of Ceruloplasmin expression | RPL12,RPS4X,RPL10A,RPLP0,EIF2S1,RPL5,EIF3E,PABPC4,PABPC1 | 0.48 | 0.32 | 0.0387 |
| MAP-201681 | Reactome Pathways | 10 | TCF dependent signaling in response to WNT | PSMD5,PSME2,PSMA2,PSMB1,PSMA6,PSMC3,PSMD13,PSME1,PPP2R1A,PSMD6 | 0.44 | 0.32 | 0.0387 |
| MAP-174824 | Reactome Pathways | 6 | Plasma lipoprotein assembly, remodeling, and clearance | PCSK5,AP2B1,AP2A2,BMP1,LIPG,CLTC | 0.61 | 0.33 | 0.0402 |
| MAP-5663205 | Reactome Pathways | 6 | Infectious disease | IPO5,GANAB,CORO1B,RANBP1,G3BP1,HSPG2 | 0.61 | 0.33 | 0.0402 |
| MAP-1643685 | Reactome Pathways | 8 | Disease | SND1,IPO5,GANAB,CORO1B,BSG,RANBP1,G3BP1,HSPG2 | 0.51 | 0.32 | 0.0412 |
| MAP-166520 | Reactome Pathways | 7 | Signaling by NTRKs | PCSK5,AP2B1,PPP2CB,CRK,AP2A2,CLTC,PPP2R1A | 0.55 | 0.32 | 0.0440 |
| MAP-71336 | Reactome Pathways | 3 | Pentose phosphate pathway | G6PD,PGM2,TALDO1 | 0.98 | 0.35 | 0.0463 |
| MAP-141424 | Reactome Pathways | 6 | Amplification of signal from the kinetochores | PPP2CB,PAFAH1B1,PPP1CC,DYNC1H1,DYNLL2,PPP2R1A | 0.59 | 0.31 | 0.0497 |
| MAP-141444 | Reactome Pathways | 6 | Amplification of signal from unattached kinetochores via a MAD2 inhibitory signal | PPP2CB,PAFAH1B1,PPP1CC,DYNC1H1,DYNLL2,PPP2R1A | 0.59 | 0.31 | 0.0497 |
